# An engineered aldolase enables the biocatalytic synthesis of 2′-functionalized nucleoside analogues

**DOI:** 10.1038/s44160-024-00671-w

**Published:** 2024-11-05

**Authors:** Matthew Willmott, William Finnigan, William R. Birmingham, Sasha R. Derrington, Rachel S. Heath, Christian Schnepel, Martin A. Hayes, Peter D. Smith, Francesco Falcioni, Nicholas J. Turner

**Affiliations:** 1 Department of Chemistry, University of Manchester, Manchester Institute of Biotechnology, Manchester, UK; 2 Compound Synthesis and Management, Discovery Sciences, BioPharmaceuticals, R&D, AstraZeneca, Gothenburg, Sweden; 3 Early Chemical Development, Pharmaceutical Sciences, R&D, AstraZeneca, Macclesfield, UK; 4 Early Chemical Development, Pharmaceutical Sciences, R&D, AstraZeneca, Cambridge, UK

**Keywords:** Synthetic chemistry methodology, Biocatalysis

## Abstract

Nucleosides functionalized at the 2′-position play a crucial role in therapeutics, serving as both small-molecule drugs and modifications in therapeutic oligonucleotides. However, the synthesis of these molecules often presents substantial synthetic challenges. Here we present an approach to the synthesis of 2′-functionalized nucleosides based on enzymes from the purine nucleoside salvage pathway. Initially, active-site variants of deoxyribose-5-phosphate aldolase were generated for the highly stereoselective synthesis of d-ribose-5-phosphate analogues with a broad range of functional groups at the 2-position. Thereafter, these 2-modified pentose phosphates were converted into 2′-modified purine analogues by construction of one-pot multienzyme cascade reactions, leading to the synthesis of guanosine (2′-OH) and adenosine (2′-OH, 2′-Me, 2′-F) analogues. This cascade allows for the control of the 2′-functional group alongside 2-stereochemistry. Our findings demonstrate the capability of these biocatalytic cascades to efficiently generate 2′-functionalized nucleosides, starting from simple starting materials.

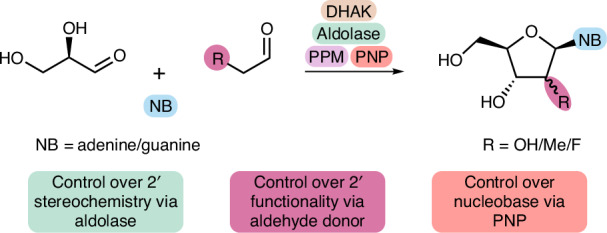

## Abstract (web summary)

The chemical synthesis of nucleoside analogues with modifications at the 2-position often requires multiple steps and the extensive use of protecting groups. Now, biocatalytic cascades are reported for the synthesis of 2-functionalized sugars and 2′-functionalized nucleosides, using enzymes derived from those of the purine nucleoside salvage pathway.

## Main

**Fig. 1:**
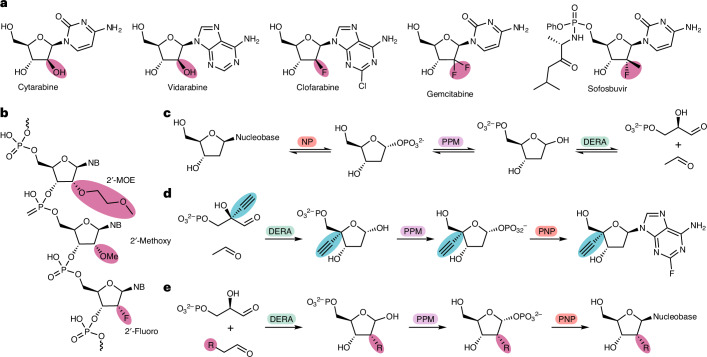
Overview of previous studies and this work. **a**, Examples of 2′-modified nucleoside and nucleotide analogues. **b**, Examples of common 2′-modifications made to therapeutic oligonucleotides. **c**, Nucleoside salvage pathway enzymes involved in the breakdown and recycling of nucleosides. **d**, Previously published biocatalytic synthesis of islatravir^8^ using engineered variants of salvage pathway enzymes. **e**, This work: proposed synthesis of 2′-functionalized nucleosides using non-natural aldol donors resulting in modifications (R group) at the 2′-position. NB, nucleobase.

Nucleoside analogues are an important class of pharmaceutical agent showing activity towards a range of biological targets^1^. For therapeutic applications nucleosides are typically modified at a variety of different positions including the ribose sugar, the phosphate backbone or the nucleobase, depending on the desired pharmacological properties^2^. Particularly important and synthetically challenging is the 2′-modification of the ribose sugar, which is utilized both in nucleoside analogue drugs (Fig. 1a) and in nucleoside building blocks of therapeutic oligonucleotides (Fig. 1b). Therapeutic oligonucleotides are an important new modality that facilitate a precision medicine approach for patients^3,4^. Modifications to the 2′-position of the nucleoside, for example, 2′-methoxy, 2′-methoxyethoxy (MOE) and 2′-fluoro, are all commonly deployed substitutions in therapeutic oligonucleotides (Fig. 1b) which impart both resistance to nucleases and improvements in RNA binding affinity.

The preparation of 2′-modified nucleosides often requires multistep synthesis, with extensive protection/deprotection sequences, or generates complex mixtures of regioisomers, making the synthesis of these molecules challenging. We considered a biocatalytic route to access 2′-modified nucleosides because enzymes are often able to carry out complex transformations in an atom-efficient manner, under relatively mild conditions and without the use of protecting groups. Moreover, because different biocatalysts often function under similar temperature and pH in aqueous solution, they can be combined into multienzyme cascades allowing for two or more reactions to be carried out in one pot without the need to isolate intermediates. In many cases this approach can lead to a reduction in waste from intermediate/product isolation, and allows reactions to be coupled together, thereby overcoming potential thermodynamic barriers of individual reactions^5^. These advantages have resulted in biocatalysis becoming increasingly deployed in the synthesis of active pharmaceutical ingredients^6^.

Recent reports have shown that enzymes from the nucleoside salvage pathway^7^ (Fig. 1c) provide a useful platform for the generation of functionalized nucleosides. In the retrosynthetic direction, this pathway is responsible for the degradation of 2′-deoxynucleosides. Starting with a nucleoside phosphorylase (NP) the nucleobase is removed, resulting in the generation of a C1 phosphate sugar (Fig. 1c). In the following step, phosphopentomutase (PPM) catalyses transfer of the phosphate group from the 1-position to the 5-position; finally, deoxyribose-5-phosphate aldolase (DERA) breaks down the sugar into acetaldehyde and d-glyceraldehyde-3-phosphate (d-G3P) via a retroaldol reaction. Because all three enzymes catalyse reversible reactions, they can be combined in the synthetic direction to generate nucleosides. An excellent example of this approach is the synthesis of the antiviral nucleoside analogue islatravir^8^ (Fig. 1d). There are additional examples of enzymes on this pathway being used to generate nucleoside analogues such as dideoxyinosine^9^ and molnupiravir^10^. There are also many examples of other biocatalysts being used in the synthesis of nucleosides, nucleotide analogues^11,12^ and oligonucleotides^13^.

To exploit this pathway for the synthesis of 2′-modified nucleosides, all enzymes in the pathway would need to possess sufficient promiscuity to tolerate a wide range of 2′-modifications. The initial challenge posed by this synthetic cascade is the limited substrate scope of DERA. While PPM and purine nucleoside phosphorylase (PNP) enzymes have been shown to tolerate a small number of 2′-modifications^14,15^, thus far the approach has been limited to 2′-deoxynucleosides when starting from DERA, with acetaldehyde as the donor. Engineering DERA would allow the synthesis of 2-modified d-ribose-5-phosphate (R5P) analogues, starting from d-G3P and a suitable aldehyde donor. The products can then be further modified with the remaining enzymes in the pathway to generate 2′-functionalized nucleosides (Fig. 1e).

Aldolases tend to have more limited substrate scope for the donor rather than the acceptor, and consequently they are often classified based on their donor selectivity^16^ with the acetaldehyde-dependent enzyme DERA being a prime example^17^. DERA catalyses the reversible aldol condensation of acetaldehyde and d-G3P and is typically only able to accept a small range of donor analogues, such as propanal or glycolaldehyde, often coming at the cost of reduced activity^18^. A recent report demonstrated that screening a diverse panel of DERAs resulted in activity toward six different aldehydes^19^. Recent work has also involved engineering DERA to expand the activity of the wild-type enzyme, resulting in variants able to catalyse the Michael addition of nitromethane to α,β-unsaturated ketones^20^.

In this work, we demonstrate that mutations in the active site of *Escherichia coli* DERA (*Ec*DERA) result in a considerably broadened donor substrate scope, allowing for the synthesis of a wide range of 2-modified-5-phosphate sugars. We have used these DERA variants to develop a cascade for the synthesis of 2-functionalized d-ribose- and l-lyxose-5-phosphate analogues with a range of functional groups including fluoro, MOE and benzyloxy (OBn). Further addition of PPM and PNP results in a one-pot synthesis of adenosine analogues functionalized at the 2′-position with –OH, –araOH, –Me and –F.

## Results and discussion

### Broadening the donor substrate scope of DERA

**Fig. 2:**
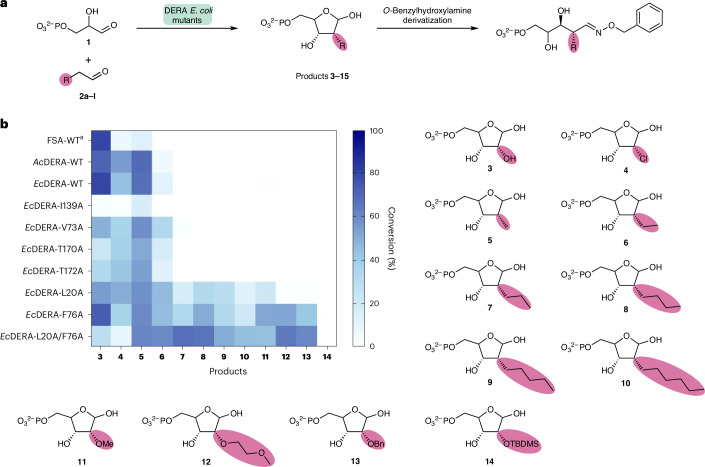
Screening of DERA variants. **a**, Assay of DERA variants towards a range of aldehyde donors using dl-G3P as acceptor. Conversion was monitored by HPLC using UV at 220 nm after derivatization with *O*-benzylhydroxylamine. **b**, Heatmap showing the average conversions of triplicate repeats for each of the aldolase variants with a range of aldehyde donors generating products (**3**–**14**). The stereochemistry of these products is assigned based on the mechanism of DERA. The stereochemistry was later confirmed for a subset of 2′-functionalized analogues (Fig. 6). ^a^For FSA the opposite stereochemistry at C2 is generated to the stereochemistry shown. Source data

Previous work suggested that the restriction in the donor substrate scope of DERA was possibly due to steric constraints in the active site^17^. Moreover, the substrate scope of fructose-6-phosphate aldolase (FSA) was greatly expanded by a ‘minimalist protein engineering’ approach that increased space in the active site^21^. We initially decided to focus on a similar strategy, generating mutations in the active site of DERA with the intention of enlarging the binding pocket for the donor substrate. The previously reported crystal structure of *Ec*DERA [PDB:1JCL]^18^, which contains the linear aldol product bound to the catalytic lysine residue, was used to identify six active-site residues in close proximity to the 2-position of the final product. These residues were mutated to alanine, and the resulting variants screened for activity against a broad panel of aldehyde donor substrates (Fig. 2).

In addition to the *Ec*DERA variants, wild-type DERA from *Arthrobacter chlorophenolicus* (*Ac*DERA), which was recently shown to have the broadest donor substrate scope of a panel of DERAs^19^, and wild-type FSA from *E. coli* (*Ec*FSA) were also screened. The panel of aldehyde donors included halogen (Cl)-substituted, two functional groups found in therapeutic oligonucleotides (OMe and MOE) and two typical protecting groups used in nucleoside chemistry (OBn and *O*-*tert*-butyldimethylsilyl (OTBDMS)). These aldehydes were all screened with dl-G3P (**1**) as the acceptor.

The *Ec*DERA-L20A and *Ec*DERA-F76A variants showed notable improvement over both wild-type *Ec*DERA and *Ec*FSA. Between both variants, conversions ranging from 22% to 73% were obtained towards all substrates screened with the exception of OTBDMS which showed no activity towards any of the enzyme variants. For the majority of substrates, *Ec*DERA-F76A gave higher conversion than *Ec*DERA-L20A. The double alanine mutation at these two positions *Ec*DERA-L20A/F76A showed increased conversion with larger aldehyde substrates (MOE, OBn, heptanal), at the cost of decreased conversion with a selection of other functional groups (OH, Cl, OMe).

The equilibrium of this aldolase reaction for the wild-type substrate (acetaldehyde) has been shown to favour the formation of the aldol addition product deoxyribose-5-phosphate^22^. In addition to this, calculation of the equilibrium constants for a model reaction (synthesis of product **3**) via eQuilibrator^23^ gave an equilibrium constant *K* = 5.4 × 10^2^. The equilibrium for the remaining products **3**–**14** similarly favours the synthetic direction.

In practice, however, these reactions did not proceed to the expected thermodynamic equilibrium values. A time-course study (Supplementary Information, section 6.3.2) showed that the optimal reaction length was 4 h, with longer reaction times resulting in a decrease in product formation. This was probably due to the chemical degradation of the G3P starting material^24^, combined with the reversible nature of the reaction, shifting the equilibrium away from the desired product. In the future, this could be mitigated by engineering the enzymes in the cascade to function at lower pH, where G3P is more stable.

### Computational rationalization of substrate scope

To rationalize the increase in activity of the three active variants, models were generated in silico and compared to that of the *E. coli* wild-type DERA. The binding pocket volumes for all four enzymes were analysed using fpocket^25^ and compared. As predicted, mutation of the selected residues to alanine resulted in an increase in the volume of the active site for all variants. The wild-type enzyme has a binding pocket volume of 317 Å^3^; this increases to 416 Å^3^ for L20A, 447 Å^3^ for F76A and 529 Å^3^ for L20A/F76A. These increases in binding pocket size seem to correlate with the trend of activity observed: larger donor molecules showed little to no activity towards the wild-type enzyme, but show better activity with the larger binding pockets of the variants. Conversely, smaller donor molecules, which were already active towards the wild type, did not benefit from the increased active site volume.

**Fig. 3:**
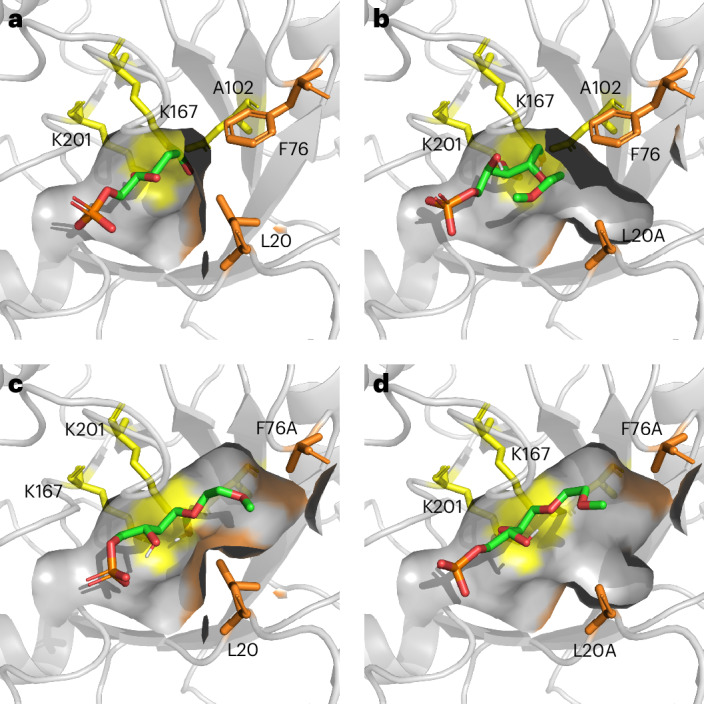
Docked products and active site volumes. **a**–**d**, The binding pockets for DERA and the three variants: wild-type *E. coli* crystal structure from 1JCL (**a**); L20A variant (**b**); F76A variant (**c**); L20A/F76A variant (**d**), with active site volumes of 317 Å^3^, 416 Å^3^, 447 Å^3^ and 529 Å^3^, respectively. Each of the variants are shown with the 2-MOE substrate covalently docked into the active site. Catalytic residues are shown in yellow, residues targeted for mutation are shown in orange.

To determine whether the generated products are able to fit inside the active site and occupy a reasonable binding mode a representative product functionalized with 2-MOE was covalently docked into the three variants using Autodock^26^ and compared to the wild-type ligand in the crystal structure (Fig. 3). For all three variants the 2-MOE-functionalized products were able to occupy a binding mode similar to that of the wild-type product (2-deoxy) and in all cases the larger functional group added at the 2-position was able to occupy the newly generated binding pockets. Further study, in particular molecular dynamics, may help to better understand the effect of these mutations on the conformation of the active site and the increased donor substrate scope.

### Synthesis of 2-modified pentose-5-phosphates

**Fig. 4:**
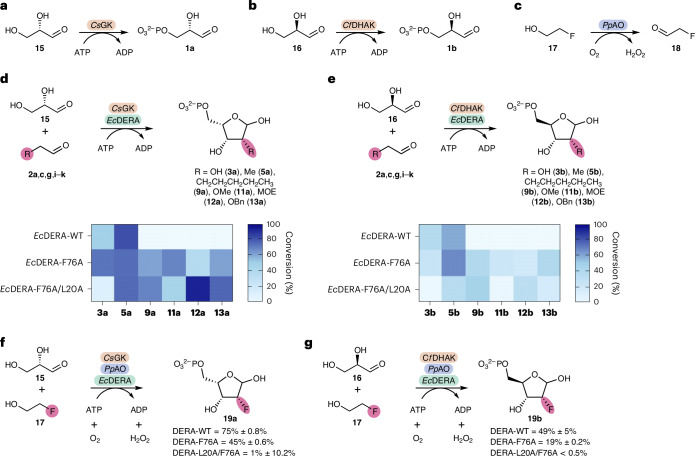
Generation of 2-functionalized stereopure pentose-5-phosphates via multistep cascades. **a**, Generation of l-G3P (**1a**) from l-glyceraldehyde (**15**) via glycerokinase from *Cellumonas* sp. (*Cs*GK). **b**, Generation of d-G3P (**16**) from d-glyceraldehyde (**1b**) via *Cf*DHAK. **c**, Generation of fluoroacetaldehyde from fluoroethanol via alcohol oxidase from *P. pastoris* (*Pp*AO). **d**, Synthesis of l-lyxose-5-phosphate analogues via two-step biocatalytic cascades using *Cs*GK and *Ec*DERA variants. **e**, Synthesis of d-ribose-5-phosphate analogues via two-step biocatalytic cascades using *Cf* DHAK and *Ec*DERA variants. Heatmaps show average conversions for triplicate repeats. **f**, Synthesis of 2-*F*-l-lyxose-5-phosphate via one-pot biocatalytic cascade using *Pp*AO, *Cs*GK and wild-type (WT) *Ec*DERA. Values represent average conversions ± s.d. **g**, Synthesis of 2-*F*-d-ribose-5-phosphate via one-pot biocatalytic cascade using *Pp*AO, *Cf*DHAK and wild-type *Ec*DERA. Values represent average conversions ± s.d. Source data

Glyceraldehyde-3-phosphate is both expensive and relatively unstable, degrading to methyl glyoxal and free phosphate at neutral pH^24^. One way to offset these problems is to generate d- or l-G3P from d- or l-glyceraldehyde, respectively, in situ using a kinase, an approach that has been employed previously when using FSA^27,28^. l-G3P (**1a**) can be generated from l-glyeraldehyde (**15**) using glycerokinase from *Cellumonas* sp.^29^ (Fig. 4a), and d-G3P (**1b**) can be generated from d-glyceraldehyde (**16**) using dihydroxyacetone kinase from *Citrobacter freundii*^30^ (*Cf*DHAK, Fig. 4b). These kinase enzymes were therefore combined into two-step cascades with the active *Ec*DERA variants.

For both reactions, enzyme loadings were optimized (Supplementary Information, section 8.2.1) and the cascades were screened towards a selection of substrates from the initial panel (Fig. 4d,e). In general, a similar pattern of activity for the wild type and the two variants was seen compared to the initial screen. However, for both cascades, when starting from enantiomerically pure glyceraldehyde, only a single product peak was observed in the HPLC traces (compared to two peaks for dl-G3P) which suggested the formation of a single product diastereomer at the 2-position as desired (Supplementary Information, section 11.15.1).

**Fig. 5:**
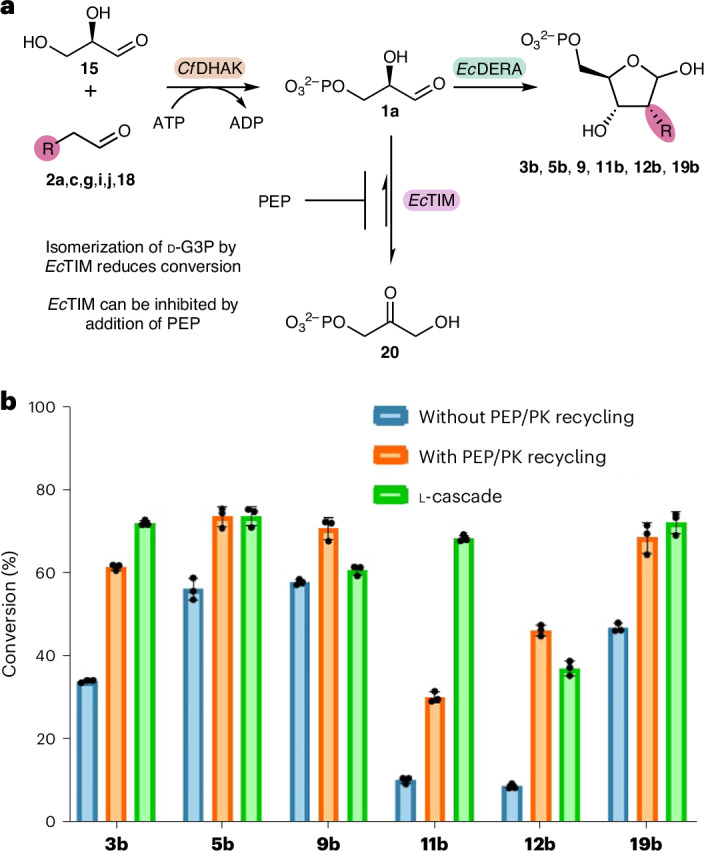
Inhibition of TIM. **a**, The isomerization of d-G3P (**1a**) into DHAP (**19**) via *Ec*TIM and potential inhibition via PEP. **b**, Mean conversion of triplicate repeat biotransformations, for a range of d-ribose-5-phosphate analogues with and without the addition of the PK-recycling system alongside conversions for the l-cascade. Error bars show s.d.; individual data points for triplicate repeat are also shown. Source data

Interestingly, when comparing the two reactions, the cascade starting from l-glyceraldehyde performed notably better than with d-glyceraldehyde. One possible explanation for this difference is the presence of native *E. coli* enzymes coexpressed with the *Ec*DERA variants. For example, d-G3P is a substrate for triose phosphate isomerase (TIM), a highly active enzyme that is rate-limited only by diffusion of G3P into the active site^31^. Even small quantities of this enzyme present from *E. coli* remaining after purification are likely to be sufficient to isomerize a notable proportion of d-G3P into dihydroxyacetone phosphate (DHAP, **20**) thus lowering conversions (Fig. 5a). Because l-G3P is not a substrate for this isomerase, the cascade starting from l-glyceraldehyde can be assumed to show the optimal conversions for the reaction in the absence of enzymatic side reactions; however, both cascades are still affected by the chemical degradation of G3P.

Despite this side reaction, the d-ribose-5-phosphate analogues were all generated with conversions ranging from 22% to 66% for all the substrates screened.

### Synthesis of 2-fluoro pentose-5-phosphates

One modification of particular interest for therapeutic oligonucleotides is 2ʹ-fluoro, which is used in several currently approved therapeutic oligonucleotides (lumasiran, inclisiran, etc.)^32^. Synthesis of the 2-fluoro-modified R5P requires fluoroacetaldehyde **18** as the donor substrate. In view of the difficulty of handling aldehyde **18** we decided instead to generate it in situ using an oxidase. Initial work showed that fluoroacetaldehyde **18** could be generated from fluoroethanol **17** using wild-type methanol alcohol oxidase from *Pichia Pastoris* (*Pp*AO) (Fig. 4c). This system was then combined with both kinase enzymes, *Cs*GK or *Cf*DHAK, and screened with the wild-type *Ec*DERA alongside the two best-performing variants.

Combining the oxidase, kinase and aldolase enabled the synthesis of fluorinated products **19a** and **19b** (Fig. 4f,g). In contrast to the previous aldehyde substrates, with fluoroacetaldehyde **17** the wild-type enzyme gave higher conversion than both the F76A and L20A/F76A variants, presumably due to the small size of the fluorine substituent. The HPLC traces for this cascade (Supplementary Information, sections 9.2.1 and 9.3.1) contained multiple peaks, suggesting the presence of a mixture of diastereomers at the 2-position (**19a** d.r., 12:88; **19b** d.r., 50:50). Carrying out a time-course analysis for the synthesis of product **19b** (Supplementary Information, section 9.2.4) demonstrated little difference in diastereomeric ratio at 1 h compared to 18 h, suggesting that this lack of diastereoselectivity is not due to thermodynamic effects, as can be observed with threonine-dependent aldolases^33^.

### Inhibition of TIM activity by phosphoenolpyruvate

As highlighted above, the isomerization of d-G3P **1a** to DHAP **20** catalysed by TIM presented a problem for the cascade to generate d-ribose-5-phosphate analogues (Fig. 5a). Phosphoenolpyruvate (PEP) has been previously shown to inhibit TIM^34^ and can also be used as a phosphate donor substrate for pyruvate kinase (PK) to implement an ATP-recycling system. We reasoned that addition of PEP to the cascade could serve to both recycle ATP for the kinase step and to increase overall conversion to product by inhibiting the formation of DHAP via TIM.

Reactions were screened using the F76A variant, both with and without the PEP/PK-recycling system present (Fig. 5b). PEP (10 mM, 2 equiv.) was added to ensure efficient inhibition of TIM throughout the reaction while still enabling recycling of ATP. Addition of the PEP/PK-recycling system resulted in an increase in conversion for all donor substrates screened. For the majority of donors, conversions to the d-R5P analogues in the presence of the PEP/PK-recycling system matched or exceeded the conversions to equivalent l-L5P products.

Addition of the PEP/PK-recycling system enables not only the use of catalytic amounts of ATP but also reduces the effects of TIM on the cascade, enabling the generation of d-R5P analogues in higher conversions.

### Semipreparative-scale synthesis of pentose-5-phosphates

Following the development of successful analytical-scale reactions, a number of key substrates were then carried through on a semipreparative scale using *Ec*DERA-F76A with a substrate loading of 20 mM glyceraldehyde.

**Fig. 6:**
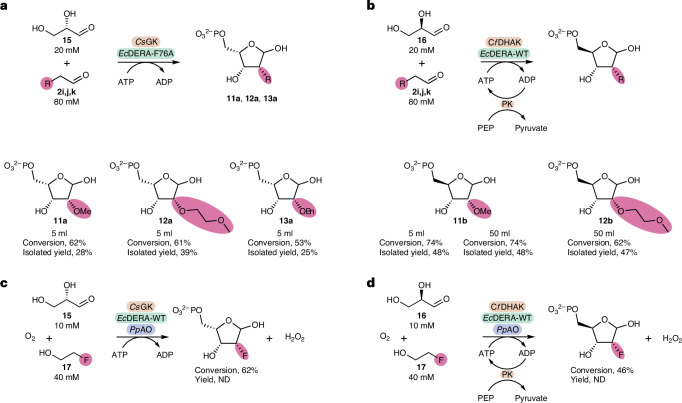
Semipreparative-scale syntheses of 2-functionalized pentose-5-phosphates. **a**, Semipreparative-scale synthesis of l-lyxose-5-phosphate analogues via a two-step cascade with *Cs*GK and *Ec*DERA-F76A. **b**, Semipreparative-scale synthesis of d-ribose-5-phosphate analogues via a two-step cascade with *Cf*DHAK and *Ec*DERA-F76A. **c**, Semipreparative-scale synthesis of 2-*F*-l-lyxose-5-phosphate via *Cs*GK, methanol alcohol oxidase (*Pp*AO) and wild-type *Ec*DERA. **d**, Semipreparative-scale synthesis of 2-*F*-d-ribose-5-phosphate via *Pp*AO, *Cf*DHAK and wild-type *Ec*DERA. Products were purified by anion-exchange chromatography and isolated as ammonium salts.

For the synthesis of l-lyxose-5-phosphates **11a**–**13a** (Fig. 6a) the ATP-recycling system was omitted because the PEP-based inhibition of TIM was not needed for l-G3P. Reactions were instead carried out at 20 mM substrate loading with stoichiometric amounts of ATP. The initial substrate chosen to analyse was the 2-OMe, due to its diagnostic singlet peak in ^1^H NMR. Conditions for the scale-up reaction of l-lyxose-5-phosphates were optimized via design of experiment (DOE) (Supplementary Information, section 11.1.1). The most important factors were found to be both aldolase and donor substrate concentrations.

For the synthesis of d-ribose-5-phosphates **11b** and **12b** (Fig. 6b) reactions were carried out at 20 mM substrate loading with the PEP/PK-recycling system. The reaction conditions were again optimized by DOE (Supplementary Information, section 11.1.2). Reaction products from both cascades were purified by anion-exchange chromatography.

For the synthesis of l-lyxose-5-phosphate analogues, 2-OMe and MOE products **11a** and **12a** were generated in good conversions (62% and 61%, respectively) and isolated in reasonable yields (28% and 39%, respectively). For the benzylated product **13a**, conversion of 53% was obtained alongside an isolated yield of 25%.

For the d-cascade, 2-OMe and 2-MOE products **11b** and **12b** were obtained in high analytical yields (74% and 62%, respectively) and good isolated yields (48% and 47%, respectively). Interestingly for **11b**, the scale-up analytical yield was higher than any of the previous optimization reactions carried out in 200 µl volumes. When compared to the l-lyxose-5-phosphate products, d-ribose-5-phosphate analogues contained a small amount of pyruvate, a by-product of the recycling system, alongside small amounts of unknown impurities. The synthesis of the 2-OMe analogue **11b** was further scaled up to a 50 ml reaction volume. This reaction showed a conversion of 71% and an isolated yield of 62% (168 mg). At this larger scale the product was isolated alongside an unknown phosphorylated impurity. Nonetheless, an almost identical conversion was obtained between both the 5 ml and 50 ml scale reactions, demonstrating the potential scalability of this cascade.

Preparative-scale syntheses of the 2-F products were also carried out (Fig. 6c,d). For these reactions several alterations had to be made to the reaction conditions. To overcome issues with oxygen limitation posed by the oxidase, the concentration of substrate was decreased to 10 mM and the reaction was carried out as a series of smaller-volume biotransformations (10 × 500 µl). For 2-*F*-l-lyxose-5-phosphate **19a** a conversion of 62% was obtained, whereas the 2-*F*-d-ribose-5-phosphate **19b** was generated with a conversion of 46%.

Mass spectrometry electrospray ionization (ESI) analysis confirmed the presence of desired [M–H]^−^ ions in both products. While HPLC analysis of the 2-F products seemed to show a mixture of two diastereomers, NMR spectra showed a more complex mixture of products. Both ^1^H and ^19^F NMR analysis appeared to show multiple unknown peaks in addition to the expected four diastereomers (Supplementary Information, sections 11.2.5 and 11.3.4).

The stereochemistry of the isolated products was determined by comparison to previous work and by the use of ^1^H–^1^H nuclear Overhauser effect NMR spectroscopy (Supplementary Information, section 11.5.2). Products were assigned the 2*R*,3*R* stereochemistry, in agreement with previous work^19^.

### Biocatalytic synthesis of 2′-functionalized nucleosides

**Fig. 7:**
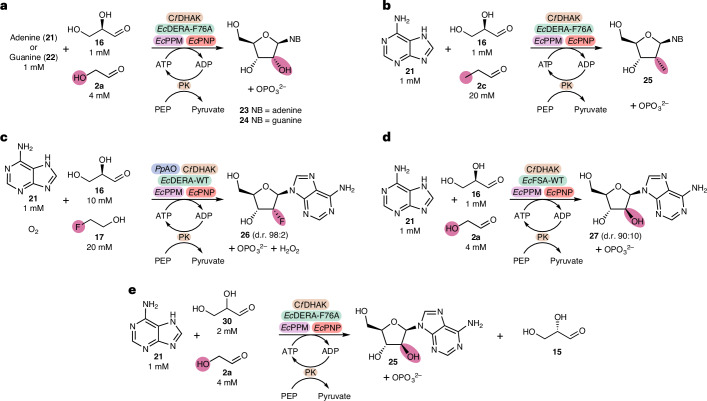
Synthesis of 2′-modified purine analogues via one-pot multienzyme cascades. **a**, Synthesis of adenosine/guanosine via a four-step cascade. **b**, Synthesis of 2′-Me adenosine via a four-step cascade. **c**, Synthesis of 2ʹ-fluoro adenosine via a five-step biocatalytic cascade. Fluoroacetaldehyde is generated in situ via the oxidase *Pp*AO. **d**, Replacement of the aldolase DERA with d-fructose-6-phosphate aldolase (wild-type *Ec*FSA) allows for the synthesis of the 2ʹ-arabinosyl analogue vidarabine. **e**, Synthesis of stereopure adenosine starting from dl-glyceraldehyde.

Finally, to demonstrate the application of this cascade to generate nucleosides functionalized at the 2′-position the aldolase step was combined with phosphopentomutase (wild-type *Ec*PPM) and purine nucleoside phosphorylase (wild-type *Ec*PNP) to synthesize guanosine and adenosine analogues in a one-pot reaction (Fig. 7).

Initial studies showed that the five-enzyme cascade (Fig. 7a) was able to generate the ribonucleoside adenosine **25** in good conversions (Table 1). Furthermore, as wild-type *Cf*DHAK is enantiospecific, d-glyceraldehyde can also be substituted with 2 equiv. dl-glyceraldehyde (Fig. 7e), removing the need to start from enantiopure starting material.

**Table 1:** Biocatalytic synthesis of 2′-functionalized nucleoside via one-pot cascades


Entry	2′-Modification	Nucleobase	Sugar equivalents^a^	Aldolase loading (variant)	PPM loading (mol%)	PNP loading (mol%)	Conversion^b^ (%)
1	OH	Adenine	1×	7 mol% (*Ec*DERA-F76A)	0.2	0.4	61 ± 1
2^c^	OH	Adenine	2×	7 mol% (*Ec*DERA-F76A)	0.2	0.4	64 ± 1
3	OH	Adenine	10×	7 mol% (*Ec*DERA-F76A)	0.2	0.4	94 ± 1
4	Me	Adenine	1×	7 mol% (*Ec*DERA-WT)	2	8	ND
5	Me	Adenine	10×	7 mol% (*Ec*DERA-WT)	2	8	17 ± 1
6	Me	Adenine	10×	7 mol% (*Ec*DERA-F76A)	2	8	38 ± 1
7	Me	Adenine	10×	7 mol% (*Ec*DERA-F76A)	2	16	65 ± 8
8	F	Adenine	1×	7 mol% (*Ec*DERA-WT)	2	8	ND
9	F	Adenine	10×	7 mol% (*Ec*DERA-WT)	2	8	6 ± 1
10	F	Adenine	10×	7 mol% (*Ec*DERA-WT)	2	16	20 ± 1
11^d^	F	Adenine	10×	7 mol% (*Ec*DERA-WT)	2	16	28 ± 1
12	OH	Guanine	1×	7 mol% (*Ec*DERA-F76A)	0.2	0.4	24 ± 1
13	AraOH	Adenine	1×	4 mol% (*Ec*FSA-WT)	0.2	0.4	32 ± 1

^a^Sugar equivalents as determined by the equivalents of d-glyceraldehyde relative to nucleobase.

^b^Average conversions ± s.d.

^c^Reaction carried out with 2 mM dl-glyceraldehyde as opposed to 1 mM d-glyceraldehyde, conversion reported with respect to limiting nucleobase.

^d^Reaction from entry 10 repeated, replacing wild-type PNP with the best-performing variant from ref. ^37^. ND, not determined.

Calculation of the overall thermodynamic equilibrium for the synthesis of adenosine gave a value of *K* = 4.0 × 10^2^ (Supplementary Information, section 14.1) suggesting the cascade should favour nucleoside formation. Previous work has shown that equilibrium constants of phosphorolysis are largely unchanged for 4′-functionalized nucleoside analogues^35^. It is likely, therefore, that the 2′-functionalized nucleoside analogues generated here show similarly favourable equilibrium constants to the synthesis of adenosine.

In practice, the experimental conversion did not reach the expected equilibrium values. A time-course analysis showed an initial increase in conversion over time followed by a decrease in conversion at longer reaction times (Supplementary Information, section 14.2). In the same manner as the aldolase reaction, this was assumed to be due to the degradation of G3P shifting reaction equilibrium away from the product. Addition of 10 equiv. glyceraldehyde appeared to mitigate these effects, showing both higher conversions and no decrease in conversion over time.

While the 2′-substrate scope of wild-type PPM and PNP is likely to be restricted, it was found that in addition to 2′-OH, 2′-Me **27** and 2′-F **28** substitutions were accepted, by both enzymes, albeit with lower conversions (Table 1). For both of these analogues, a 10× excess of the d-glyceraldehyde starting material was required to give reasonable conversions. Alongside mitigating the effects of G3P degradation, the requirement of a 10_×_ excess to observe conversion is presumably due to the lower activity of the wild-type *Ec*PNP towards these non-natural substrates.

DOE was used to determine the effects of enzyme loading on conversion for the three main enzymes (DERA, PPM and PNP) (Supplementary Information, section 13). This revealed that the concentration of PNP was by far the most limiting of the three enzymes. PNPs typically show Michaelis constant (*K*_m_) values in the high micromolar to low millimolar range^36^. Therefore, under the conditions at which the cascade is being carried out, PNP is probably kinetically limited, operating well below the *K*_m_. By increasing the concentration of PNP in these reactions, an increase in conversions for both 2′-Me (from 38% to 65%) and 2′-F (from 6% to 20%) was observed (Table 1). Addition of a recently published PNP variant, which was engineered to give a small increase in activity to 2′-F adenosine^37^, resulted in a further increase in conversion to 28% (Table 1). The lower conversions seen for the fluorinated analogues are in part due to around 20% conversion to the by-product to 2′-F inosine, via adenosine deaminase (Supplementary Information, section 12.3.3). This issue is compounded by the relatively large amounts of protein required and highlights the need for further engineering, particularly of the PNP enzyme.

In addition to adenosine, guanosine was also synthesized using the same conditions as the adenosine reaction (Table 1). Conversion to guanosine was notably lower than for adenosine. The equilibrium constant for the overall synthesis of guanosine is lower than that of adenosine (1.4 × 10^2^), due to guanosine having a higher equilibrium constant for phosphorolysis^35^, and the reaction is therefore less favourable in the synthetic direction. In addition to this, the nucleobase substrate guanine shows much poorer solubility than adenine.

As the stereochemistry of the 2′-position is set by the aldolase used, changing to a different aldolase allows for control of this stereochemistry. Thus, replacement of DERA by wild-type *Ec*FSA^38^ resulted in the synthesis of the nucleoside analogue vidarabine (2′-araOH adenosine, **29**) (Table 1). Vidarabine was generated alongside a small amount of adenosine, in a diastereomeric ratio of 90:10.

Finally, to characterize the 2′-OH, 2′-araOH, 2′-Me and 2′-F adenosine analogues by ^1^H NMR 5 ml scale reactions were carried out and the products were purified by semipreparative HPLC. For adenosine, vidarabine and the 2′-Me adenosine analogues the ^1^H NMR spectra confirmed the products were isolated as a single diastereomer at the 2′-postion. For 2′-F adenosine **28**, despite the aldolase step generating a mixture of 2′-F diastereomers, the 2′-F adenosine product was synthesized in a diasteromeric ratio of 98:2 (Supplementary Information, section 15.4.3) favouring the desired ‘down’ stereochemistry of the fluorine. These isomers were unable to be separated by semipreparative HPLC and were isolated together. This observation suggests that, while the aldolase exhibits poor stereochemical control, one or both of the final two enzymes in the cascade are stereoselective for the desired fluorine diastereomer. Alongside the desired 2′-F adenosine we were also able to isolate the 2′-F inosine side product (Supplementary Information, section 14.5), thereby demonstrating the potential to generate 2′-functionalized inosine analogues by addition of a deaminase enzyme to the cascade.

## Conclusions

By targeting the active site of wild-type *Ec*DERA, simple mutations (F76A and L20A) have considerably expanded the donor substrate scope, enabling the synthesis of a diverse range of d-ribose-5-phosphate and l-lyxose-5-phosphate analogues in two- or three-step cascades. Semipreparative-scale biotransformations successfully produced 2-OMe, 2-MOE, 2-OBn and 2-F analogues with reasonable conversions and yields of 5.8–15.2 mg. A further 50 ml scale-up reaction yielded over 150 mg of the 2-OMe analogue **11b**, highlighting the scalability of this cascade. Furthermore, with the exception of the fluorinated products, all analogues were synthesized as single diastereomers at the 2-position, demonstrating the excellent stereoselectivity of DERA.

Combination of these engineered aldolase-based cascades with wild-type *Ec*PPM and *Ec*PNP enabled the one-pot synthesis of ribonucleosides, including adenosine and guanosine, alongside the 2′-Me and 2′-F adenosine analogues. This demonstrates the ability to generate 2′-functionalized analogues in a one-pot cascade from simple starting materials. The modularity of this cascade was further demonstrated by substituting *Ec*DERA-F76A with wild-type *Ec*FSA, which imparted stereochemical control at the 2′-position, thereby facilitating the synthesis of the arabinosyl nucleoside analogue vidarabine.

Like many biocatalytic routes to nucleoside analogues^39^, this cascade currently suffers from relatively large amounts of waste water and low substrate loadings. Despite these shortcomings, the cascade provides a completely protection-group-free synthesis of 2ʹ-functionalized nucleoside analogues, which represents a notable improvement over traditional chemical methods. Further improvements in process development should help to improve both the yield and the E-factor of this cascade. This is exemplified by recent developments to the synthesis of islatravir and molnupiravir^40^, which demonstrate the ability of nucleoside salvage pathway enzymes to generate these analogues at high substrate loadings on a multikilogramme scale.

Although PPM and PNP have been shown to tolerate a small range of 2′-functionalization such as 2′-NH_2_, araOH and 2′-F, cascades utilizing DERA have thus far been limited to the synthesis of 2′-deoxynucleosides. This work shows that a much broader range of 2′-functionalization is possible via engineering of the aldolase, and that installation of a range of functionality is possible by simply changing the aldehyde donor used. We consider this to be the first step towards the repurposing of this pathway towards generating a much broader range of nucleoside analogues. Further engineering of PPM and PNP, in particular for increased substrate scope at the 2ʹ-position, will hopefully increase both the scope and yield of nucleoside analogues via this approach. Further diversification of the nucleoside analogue may also be possible, either via the nucleoside phosphorylases themselves, or by combining this cascade with other enzymes able to carry out transglycosylation reactions^41^.

## Methods

### Aldolase reactions

A reaction mixture was made up containing dl-G3P (5.5 mM; final reaction concentration, 5 mM), HEPES buffer (111 mM; final reaction concentration, 100 mM) and aldolase (2.2 mg ml^−1^; final reaction concentration, 2 mg ml^−1^). To a 96-well plate 10 μl of donor substrate (200 mM; final reaction concentration, 20 mM) was added. For substrates insoluble in water, donor solutions were made up in dimethylsulfoxide, giving a final concentration of 10%. Reactions were initiated by addition of 90 μl of the reaction mixture to 10 μl of donor substrate; plates were then sealed and incubated at 30 °C, shaking at 900 rpm. After 4 h the reactions were quenched and derivatized with 100 μl of *O*-benzylhydroxylamine (100 mM) in methanol for 1 h. Reactions were then filtered through 96-well 0.45 Å filter plates and analysed by ultraperformance liquid chromatography (UPLC) with UV detection at 220 nm. Product masses were confirmed by UPLC–mass spectrometry.

### Kinase-aldolase cascade screening

To a 96-well plate, 10 µl of donor substrate (200 mM; final reaction concentration, 20 mM) was added. For substrates insoluble in water, donor solutions were made up in dimethylsulfoxide, giving a final concentration of 10%. A reaction mixture was made up containing d- or l-glyceraldehyde (6.25 mM; final reaction concentration, 5 mM), ATP (9.4 mM; final reaction concentration, 7.5 mM, 1.5 equiv.), MgCl_2_ (9.4 mM; final reaction concentration, 7.5 mM, 1.5 equiv.), HEPES buffer pH 7.5 (125 mM; final reaction concentration, 100 mM), aldolase (1.25 mg ml^−1^; final reaction concentration, 1 mg ml^−1^). Then, 80 µl of the reaction mixture was added to the plates and the reactions were initiated with 10 µl of DHAK or GK (1 mg ml^−1^; final reaction concentration, 0.1 mg ml^−1^). The plates were then sealed and incubated at 30 °C, shaking at 900 rpm. After 4 h the reactions were quenched and derivatized with 100 µl of *O*-benzylhydroxylamine (100 mM) in methanol for 1 h. Reactions were then filtered through 96-well 0.45 Å filter plates to remove precipitated protein and analysed by UPLC-UV at 220 nm. Product masses were confirmed by UPLC–mass spectrometry, which gave identical mass spectra to the products from the initial screen.

### Kinase–oxidase–aldolase cascade

A 2× reaction mixture was made up containing d- or l-glyceraldehyde (10 mM,), fluoroethanol (40 mM), ATP (15 mM), MgCl_2_ (15 mM) in HEPES buffer (0.1 M, pH 7.5). A 2× enzyme mixture was made up containing DHAK or GK (0.2 mg ml^−1^), DERA (2 mg ml^−1^) and *Pp*AO (1 mg ml^−1^) in HEPES buffer (0.1 M, pH 7.5). The reaction was initiated by addition of 100 μl reaction mix to 100 μl enzyme mix giving final reaction concentrations half those stated. Samples were incubated for 4 h at 30 °C, shaking at 750 rpm. After 4 h samples were derivatized with *O*-benzylhydroxylamine, filtered and analysed by HPLC.

### Semipreparative-scale synthesis of 2-functionalized pentose-5-phosphates

To a 15 ml Falcon tube, d- or l-glyceraldehyde (20 mM final concentration), aldehyde donor (80 mM final concentration) and MgCl_2_ (20 mM) were added. HEPES buffer (pH 8.2) and MilliQ water were added to give a final buffer concentration of 50 mM and a final reaction volume of 5 ml. The reaction was initiated by addition of DERA-F76A (final concentration, 4 mg ml^−1^), DHAK (final concentration, 0.1 mg ml^−1^). The reaction mixture was left in a shaking incubator at 30 °C, 200 rpm, for 18 h. After this time the enzyme was removed using a Vivaspin 10 kDa molecular weight cut-off filter. The phosphorylated products were then purified using anion-exchange chromatography in the same manner as previous products.

For d-ribose-5-phosphates, final concentrations of 1 mol% ATP, 40 mM PEP and 10 U ml^−1^ PK were used. For l-lyxose-5-phosphates, 1.5 equiv. ATP was used without a recycling system.

The phosphorylated products were purified using anion-exchange chromatography. The reaction mixtures were loaded directly onto a 5 ml Bio-Rad High Q anion-exchange column. The column was washed with 20 ml of water followed by elution with 10 ml of 200 mM ammonium bicarbonate and 10 ml of 400 mM ammonium bicarbonate. Fractions (1 ml) fractions were collected and analysed for product presence by ESI mass spectrometry. Fractions containing product mass by ESI were pooled and freeze-dried to give the ammonium salts of the sugar phosphate products as white solids.

### Semipreparative-scale synthesis of 2-fluoro-pentose-5-phosphate

To a 15 ml Falcon tube, d- or l-glyceraldehyde (10 mM), fluoroethanol (40 mM) and MgCl_2_ (10 mM) were added. HEPES buffer (pH 8.2) and MilliQ water were added to give a final buffer concentration of 50 mM and a final reaction volume of 5 ml. The reaction was initiated by addition of wild-type DERA (4 mg ml^−1^), glycerokinase (0.1 mg ml^−1^) and *Pp*AO (0.5 mg ml^−1^). The reaction mixture was split into 10 × 500 μl reactions. These were incubated in a thermoshaker at 30 °C, 750 rpm, for 18 h. After this time fractions were combined, and the enzyme was removed using a 10 kDa molecular weight cut-off filter.

For 2-*F*-d-ribose-5-phosphate, 1 mol% ATP, 20 mM PEP and 10 U ml^−1^ PK were added. 2-*F*-l-Lyxose-5-phosphate was generated using 1.5 equiv. ATP and no recycling system.

The phosphorylated products were purified using anion-exchange chromatography in the same manner as previous products.

### Biocatalytic synthesis of nucleosides

A 2× reaction mixture was made up containing d-glyceraldehyde (2 mM), aldehyde donor (8 mM), ATP (10 mol%), PEP (4 mM), adenine (2 mM) MgCl_2_ (2 mM), MnCl_2_ (2 mM), glucose bisphosphate (0.02 mM) and HEPES buffer (50 mM, pH 8). A 2× enzyme mixture was made up containing DHAK (0.2 mg ml^−1^), DERA (4 mg ml^−1^), PPM (0.2 mg ml^−1^), PNP (0.2 mg ml^−1^) and PK (20 U ml^−1^). To initiate the reaction, 100 µl of enzyme mix was added to 100 µl of reaction mixture, giving final reaction concentrations half those stated above. The reactions were left shaking in an orbital incubator at 30 °C, 200 rpm, for 4 h. The reactions were quenched by additions of equal volumes of methanol. Samples were filtered and then analysed directly by HPLC.

For the biocatalytic synthesis of guanosine, 1 mM of adenine was replaced with 1 mM of guanine.

For the biocatalytic synthesis of vidarabine, 2 mg ml^−1^ *Ec*DERA-F76A was replaced with 1 mg ml^−1^ wild-type *Ec*FSA

For the biocatalytic synthesis of 2ʹ-Me adenosine, 10 mM of glyceraldehyde, 20 mM PEP and 20 mM propanal donor was used.

For the biocatalytic synthesis of 2ʹ-F adenosine, 10 mM glyceraldehyde, 20 mM PEP, 20 mM fluoroethanol and 0.5 mg ml^−1^ PpAO were used.

### Reporting summary

Further information on research design is available in the Nature Portfolio Reporting Summary linked to this article.

## Supplementary information

Supplementary Information Supplementary Figs. 1–141, Tables 1–37, extended material details and methods, HPLC data, LC–MS data and NMR data.

Reporting Summary

## Source data

Source Data Fig. 2 Heatmap Raw Data

Source Data Fig. 4 Heatmap Raw Data

Source Data Fig. 5 Bar Chart Raw Data

## Data Availability

The data supporting this research are available within the Article and its Supplementary Information. Plasmid maps, docked structures and NMR files are available on figshare via 10.6084/m9.figshare.26520880 (ref. ^42^). Additional data can be obtained from the corresponding author upon reasonable request.
