# Supplementary material for: An engineered aldolase enables the biocatalytic synthesis of 2′-functionalized nucleoside analogues
Source: Nat Synth. 2024 Nov 5;4(2):156–66. doi: 10.1038/s44160-024-00671-w (PMC11821518; doi:10.1038/s44160-024-00671-w)
Supplement: Supplementary file 1 — Supplementary Figs. 1–141, Tables 1–37, extended material details and methods, HPLC data, LC–MS data and NMR data. [file 44160_2024_671_MOESM1_ESM.pdf]

nature synthesis

Supplementary information https://doi.org/10.1038/s44160-024-00671-w

An engineered aldolase enables the biocatalytic synthesis of 2′-functionalized nucleoside analogues

In the format provided by the authors and unedited

Table of Contents

1. Materials 5
2. General Information 5
3. Molecular Biology Methods 6
   - 3.1 Cloning Wild Type Genes 6
   - 3.2 Mutagenesis 6
   - 3.3 Protein Expression and Purification 7
   - 3.4 Protein/Primer Sequences Used 9
4. Synthesis of Aldehyde Donors from Diacetals 12
5. HPLC Analysis of Compounds 14
   - 5.1 HPLC Analysis of Sugar Phosphates 14
   - 5.2 HPLC Analysis of Nucleosides 17
6. Initial Mutant Screen 20
   - 6.1 Example HPLC Trace 20
   - 6.2 Substrate Screen 22
     - 6.2.1 Glycolaldehyde donor (product 3) 23
     - 6.2.2 Chloroacetaldehyde donor (product 4) 24
     - 6.2.3 Propanal donor (product 5) 25
     - 6.2.4 Butanal donor (product 6) 26
     - 6.2.5 Pentanal donor (product 7) 27
     - 6.2.6 Hexanal donor (product 8) 28
     - 6.2.7 Heptanal donor (product 9) 29
     - 6.2.8 Octanal donor (product 10) 30
     - 6.2.9 Methoxyacetaldehyde donor (product 11) 31
     - 6.2.10 Methoxy(ethoxy) acetaldehyde donor (product 12) 32
     - 6.2.11 Benzyoxyacetaldehyde donor (product 13) 33
     - 6.2.12 O-TBDMS acetaldehyde donor (product 14) 34
   - 6.3 Aldolase Reaction Equilibrium 35

6.3.1 Equilibrium Constant ... 35
6.3.2 Time Course ... 35
7. Computational Methods ... 36
7.1 FPocket ... 36
7.2 Covalent Docking ... 39
8. Kinase + Aldolase Cascades ... 41
8.1 L-Kinase-Aldolase Cascade ... 41
8.1.1 Substrate Scope Screening ... 42
8.1.2 Example HPLC Trace ... 42
8.1.3 Tables of Results ... 44
8.2 D-Kinase-Aldolase Cascade ... 45
8.2.1 Optimising Enzyme Concentrations ... 45
8.2.2 Substrate Scope Screening ... 46
8.2.3 Example HPLC Traces ... 47
8.2.4 Results Tables ... 49
8.2.5 Phosphorylation of Glycolaldehyde Donor ... 50
9. Kinase + Oxidase + Aldolase Cascades ... 51
9.1 Oxidation of fluoroethanol ... 51
9.2 L-Kinase-Oxidase-Aldolase Cascade ... 52
9.2.1 Biotransformation Protocol ... 52
9.2.2 Example HPLC ... 52
9.2.3 Mass Spectra ... 53
9.2.4 Results Tables ... 54
9.2.5 Time Course ... 54
9.3 D-Kinase-Oxidase-Aldolase Cascade ... 54
9.3.1 Biotransformation Protocol ... 54
9.3.2 Example HPLC ... 55
9.3.3 Mass Spectra ... 55
9.3.4 Results Tables ... 56

10. PEP/PK Recycling System ... 56
10.1 PEP inhibition of TPI ... 56
10.2 Substrate Screening ... 58
11. Semi-Preparative Scale Synthesis of Pentose-5-Phosphates ... 59
11.1 DOE ... 59
11.1.1 L-Cascade ... 59
11.1.2 D-Cascade ... 62
11.2 Semi-Preparative Scale Syntheses of L-Lyxose-5-Phosphate Analogues ... 66
11.2.1 General Procedure for the Synthesis of L-Lyxose-5-Phosphate Analogues ... 66
11.2.2 2-deoxy-2-methoxy-L-lyxose-5-phosphate (**11a**) ... 67
11.2.3 2-deoxy-2-methyoxy(ethoxy)-L-lyxose-5-phosphate (**12a**) ... 72
11.2.4 2-deoxy-2-benzoxyl-L-Lyxose-5-phosphate (**13a**) ... 77
11.2.5 2-Deoxy-2-fluoro-L-lyxose-5-phosphate (**19a**) ... 82
11.3 Semi-preparative Scale Syntheses of D-Ribose-5-Phosphate Analogues ... 88
11.3.1 General Procedure for the Synthesis of D-Ribose-5-Phosphate Analogues ... 88
11.3.2 2-deoxy-2-methoxy-D-ribose-5-phosphate (**11b**) ... 89
11.3.3 2-deoxy-2-methoxy(ethoxy)-D-ribose-5-phosphate (**12b**) ... 93
11.3.4 2-deoxy-2-fluoro-D-ribose-5-phosphate (19b) ... 98
11.3.5 Scale Up Reaction Conversions ... 104
11.4 50 ml scale synthesis of 2-deoxy-2-methoxy-D-ribose-5-phosphate (**11b**) ... 104
11.5 Assignment of Stereochemistry ... 108
11.5.1 Analytical HPLC ... 108
11.5.2 NOESY NMR ... 109
12. Full Cascade Reactions ... 111
12.1 Adenosine (**23**) ... 111
12.1.1 Biotransformation Protocol ... 111
12.1.2 Example HPLCs ... 111
12.1.3 Mass Spectra ... 112
12.1.4 Table of Conversions ... 112

12.2 2'-Me Adenosine (**25**) 113
12.2.1 Biotransformation Protocol 113
12.2.2 Example HPLCs 113
12.2.3 Mass Spec 114
12.2.4 Table of Conversions 114
12.3 2'-F Adenosine (**26**) 115
12.3.1 Biotransformation Protocol 115
12.3.2 Example HPLCs 115
12.3.3 Mass Spec 116
12.3.4 Table of Conversions 117
12.4 Guanosine (**24**) 118
12.4.1 Biotransformation Protocol 118
12.4.2 Calculation of Conversion 118
12.4.3 Example HPLCs 119
12.4.4 Mass Spectra 120
12.5 Vidarabine (**27**) 121
12.5.1 Biotransformation Protocol 121
12.5.2 Example HPLC 122
12.5.3 Stereoselectivity 122
12.5.4 Mass Spectra 123
13. Enzyme Loading DOE 123
13.1 Table of Values 124
13.2 Results 124
14. Equilibrium 125
14.1 Calculated Equilibrium Constants 125
14.2 Time Course 127
15. Semi-Preparative Scale Synthesis of Nucleoside Products 128
15.1 HPLC Method 128
15.2 Adenosine (**23**) 129

15.2.1 Biotransformation ... 129

15.2.2 NMR ... 129

15.3 2'-Me Adenosine (**25**) ... 131

15.3.1 Biotransformation ... 131

15.3.2 NMR ... 132

15.4 2'-F Adenosine (**26**) ... 134

15.4.1 Biotransformation ... 134

15.4.2 NMR ... 134

15.4.3 Stereoselectivity ... 137

15.5 2'-F-inosine (side product) ... 138

15.6 Vidarabine (**27**) ... 139

15.6.1 Biotransformation ... 139

15.6.2 NMR ... 140

16. Uncropped SDS-PAGE gel ... 142

1. Materials

Synthetic genes were purchased from GeneArt. Oligonucleotide primers were purchased from IDT. NiNTA resin was purchased from Qiagen. PD10 desalting columns packed with Sephadex G-25 resin were purchased from Cytiva. Culture and media components were purchased from Formedium. Aldehydes and diacetals were all purchased as commercial standards at purities of >97%. Unless specified otherwise, all chemicals were purchased from Sigma-Aldrich.

2. General Information

HPLC Analysis. HPLC Analysis was carried out using an Agilent 1290 Infinity II System. Individual Methods and representative HPLC chromatograms for an example substrate have been provided where relevant.

Mass Spec Analysis. Mass spectroscopy was carried out using an Agilent 6135b single quadrupole mass spectrometer.

NMR Analysis. ^{1}H,^{13}C,^{19}F and ^{31}P analysis was carried out on a Bruker 400 Ultrasheild, a Bruker 500 Ultrashield, and a Bruker 800 Ultrashield.

Repeats. All reactions were carried out in triplicate and measurements were taken from three distinct samples.

Data Availability. NMR files, wild type plasmid maps, and docked model structures are available online on a figshare data repository (DOI = 10.6084/m9.figshare.26520880).

3. Molecular Biology Methods

3.1 Cloning Wild Type Genes

Wild-Type gene sequences were either purchased from GeneArt and cloned into pET28b vectors using NEB Hifi DNA assembly, or purchased from Twist precloned into pET28a. Empty pET28b was linearised using NdeI and XhoI restriction enzymes. To a PCR tube 1 µg of empty pET28b, 5 µl cutsmart buffer, 1 µL of NdeI, 1 µL of Xho1 and finally nuclease-free water to make up the volume to 50 µL was added. This was incubated at 37 °C for 1 hour before the addition of 1 µL CIP phosphatase and incubation for a further hour. Cut vector was run on a 1% agarose gel and extracted using a Gel Extraction Kit (Qiagen), following the manufacturer's instructions.

To a PCR tube was added 1 µL of cut pET28b (25 ng/µL), 2 µL of insert (150 ng/µL) and 3 µL of NEB Builder. The reaction was incubated at 50 °C for 1 hour. 2 µL of this reaction mixture was transformed into NEB DH5α cells, grown overnight in 10 mls of LB media, and miniprepped using a miniprep kit (Qiagen) according to the manufacturer's instructions. Following this they were sequenced (Eurofins TubSeqService).

3.2 Mutagenesis

Single- and double-point mutations were made following the quick-change mutagenesis protocol using Pfu polymerase. Briefly, primers were designed with the target mutation in the centre of the sequence and 10-15 bases of correct sequence on either side, a GC content >50% (where possible) and terminating on one or more C or G bases. To a PCR tube was added 40.5 µL H_2O , 5 µL 10x pfu reaction buffer, 1 µL of forward primer and 1 µL of reverse primer (10 µM), 0.5 µL of template DNA (100 ng/µl) and 1 µL of dNTP mix and 1 µL Pfu polymerase. The PCR was carried out according to the following cycle, for each mutation the annealing temperature was based on the theoretical melting temperature (calculated via SnapGene).

Cycle step	Temp (°C)	Time	Cycles
Initial Denaturation	95	30s	1
Denaturation<br>Annealing<br>Extension	95<br>various<br>68	30s<br>1 minute<br>1 min/kb template	18 cycles
Final Extension	68<br>4	10 mins<br>(hold)	1

After PCR reactions were carried out, 1 µL of DpnI was added and the reactions were incubated for a further 2 hours. Products were then purified using a PCR purification kit (Qiagen), following the manufacturer's instructions, transformed into DH5α cells and miniprepped for sequencing.

3.3 Protein Expression and Purification

A plasmid containing the target gene was transformed into BL21(DE3) *E. coli* competent cells (New England Biolabs) according to the manufacturers protocol and plated onto LB agar containing kanamycin at concentration 50 µg/ml. A single colony was then incubated overnight in 5 ml LB media at 37 °C, shaking at 170 rpm. Overnight inoculum was added to 600 ml of LB media containing kanamycin at concentration 50 µg/ml and incubated at 37 °C for approximately 3 hours. When OD_{600} reached 0.6, protein production was induced with IPTG to 0.2 mM final concentration and incubated overnight at 20 °C. The inoculum from these flasks was centrifuged for 20 mins at 2831 x g and the supernatant was discarded. The cell pellet was then resuspended in 50 mM HEPES, 5 mM imidazole, 250 mM NaCl, pH 7.5 (10 ml buffer per 1g of cell pellet) and the cells were lysed by sonication (20s on/ 20s off/ 20 cycles). The cell debris was separated by centrifuging at 39191 x g for 20 minutes. The cell debris was discarded, and the cell lysate was kept on ice. Proteins were purified by immobilised metal ion affinity chromatography using His Tag columns (GE healthcare HisTrap). Cell lysate was washed through column twice to ensure maximum binding. The column was then washed with 5 column volumes of 30 mM imidazole, 50mM HEPES pH 7.5 buffer. Protein was eluted using 250 mM imidazole 50 mM HEPES pH 7.5. The protein was eluted into 4 column volumes and desalted to remove imidazole using PD10 desalting columns (GE Healthcare). Fractions from the lysate, flow through, wash and elution were ran on a 4-20% SDS-PAGE gel (Bio-Rad). The concentration of protein was calculated by nanodrop spectrophotometer, using the theoretical molar extinction coefficient (ε) and MW of the protein. The purified protein was flash frozen using liquid nitrogen and stored at -80 °C. Proteins were

typically stored in 50 mM HEPES buffer at pH 7-8 (depending on the pH of the desired reaction), at concentrations of 10-20 mg/ml.

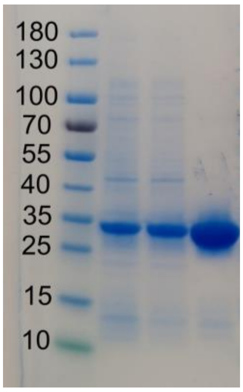

Figure 1: Example purification for DERA, ladder (Thermo PageRuler™ , 26616, masses shown in kDa), CFE (1/10 dilution), wash fraction (1/2 dilution), purified protein (1/5 dilution). Uncropped gel shown in Figure 141.

3.4 Protein/Primer Sequences Used

Table 1: Amino acid sequences of proteins and purification tags for proteins used in this work. UniProt ID numbers have been included for Wild-Type sequence. Mutations from the WT sequence are highlighted in red. Molecular weight and theoretical extinction coefficients used to calculate protein concentration have been included.

Name	Amino Acid Sequence	Purification Tag	UniProt ID	Molecular Weight (kDA)	Molar Extinction Coefficient (M^{-1} cm^{-1})
FSA_{WT}	MELYLDTSDVVAVTALSRIFPLAGVTTNPSIIA AGKKPLEVVLPQLHEAMGGQGRLFAQVMAT TAEGMVNDARKLRSIIADIVVKVPVTAEGLAAI KMLKAEGIPTLGTAVYGAAQGLLSALAGAEY VAPYVNRIDAQGGSGIQTVTDLHQLLKMHAP QAKVLAASFKTPRQALDCLLAGCESITLPLDV AQQMISYPAVDAAVAKFEQDWLGAFGRTSI*	N-Term 6xHis	P78055	23.0	13075
$EcDERA_{WT}$	MTDLKASSLRALKLMDLTTLNDDDTDEKVIAL CHQAKTPVGNTAAICIYPRFIPIARKTLKEQGT PEIRIATVTNFPHGNDDIDIALAETRAAIAYGA DEVDVVFPYRALMAGNEQVGFDLVKACKEA CAAANVLLKVIIETGELKDEALIRKASEISIKAG ADFIKTSTGKVAVNATPESARIMMEVIRDMG VEKTVGFKPAGGVRTAEDAQKYLAIADELFG ADWADARHYRFGASSLLASLLKALGHGDGK SASSY*	N-Term 6xHis	P0A6L0	27.8	14690
$AcDERA_{WT}$	MSNEATPSVQPAAAAARNIASYIDHTLLKPEA SEADVLKACAEAAEYGFKSVCVNPVWVKTV KKALKGSGVLTCSVVGFPLGATPTDVKTFEA RGAVLDGADEVDMVINIAAARADDKGALTED IASVAEAVHASGAILKVIIETSLLSDSQKVLAC EAAVAAGADFVKTSTGFNGGGATAEDVALM RRTVGPELGVKASGGVRSLADAQAMIAAGA TRIGASSGIAIVKGEQGSSAY*	N-Term 6xHis	B8HFF3	23.9	10220
$EcDERA_{L20A}$	MTDLKASSLRALKLMDLTT**A**NDDDTDEKVIA LCHQAKTPVGNTAAICIYPRFIPIARKTLKEQG TPEIRIATVTNFPHGNDDIDIALAETRAAIAYG ADEVDVVFPYRALMAGNEQVGFDLVKACKE ACAAANVLLKVIIETGELKDEALIRKASEISIKA GADFIKTSTGKVAVNATPESARIMMEVIRDM GVEKTVGFKPAGGVRTAEDAQKYLAIADELF GADWADARHYRFGASSLLASLLKALGHGDG KSASSY*	N-Term 6xHis	P0A6L0	27.8	14690

EcDERA$_{V73A}$	MTDLKASSLRALKLMDLTTLNDDDTDEKVIAL<br>CHQAKTPVGNTAAICIYPRFIPIARKTLKEQGT<br>PEIRIAT**A**TNFPHGNDDIDIALAETRAAIAYGA<br>DEVDVVFPYRALMAGNEQVGFDLVKACKEA<br>CAAANVLLKVIIETGELKDEALIRKASEISIKAG<br>ADFIKTSTGKVAVNATPESARIMMEVIRDMG<br>VEKTVGFKPAGGVRTAEDAQKYLAIADELFG<br>ADWADARHYRFGASSLLASLLKALGHGDGK<br>SASSY*	N-Term<br>6xHis	P0A6L0	27.8	14690
EcDERA$_{F76A}$	MTDLKASSLRALKLMDLTTLNDDDTDEKVIAL<br>CHQAKTPVGNTAAICIYPRFIPIARKTLKEQGT<br>PEIRIATVTN**A**PHGNDDIDIALAETRAAIAYGA<br>DEVDVVFPYRALMAGNEQVGFDLVKACKEA<br>CAAANVLLKVIIETGELKDEALIRKASEISIKAG<br>ADFIKTSTGKVAVNATPESARIMMEVIRDMG<br>VEKTVGFKPAGGVRTAEDAQKYLAIADELFG<br>ADWADARHYRFGASSLLASLLKALGHGDGK<br>SASSY*	N-Term<br>6xHis	P0A6L0	27.8	14690
EcDERA$_{I139A}$	MTDLKASSLRALKLMDLTTLNDDDTDEKVIAL<br>CHQAKTPVGNTAAICIYPRFIPIARKTLKEQGT<br>PEIRIATVTNFPHGNDDIDIALAETRAAIAYGA<br>DEVDVVFPYRALMAGNEQVGFDLVKACKEA<br>CAAANVLLKV**A**IETGELKDEALIRKASEISIKA<br>GADFIKTSTGKVAVNATPESARIMMEVIRDM<br>GVEKTVGFKPAGGVRTAEDAQKYLAIADELF<br>GADWADARHYRFGASSLLASLLKALGHGDG<br>KSASSY*	N-Term<br>6xHis	P0A6L0	27.8	14690
EcDERA$_{T170A}$	MTDLKASSLRALKLMDLTTLNDDDTDEKVIAL<br>CHQAKTPVGNTAAICIYPRFIPIARKTLKEQGT<br>PEIRIATVTNFPHGNDDIDIALAETRAAIAYGA<br>DEVDVVFPYRALMAGNEQVGFDLVKACKEA<br>CAAANVLLKVIIETGELKDEALIRKASEISIKAG<br>ADFIK**A**STGKVAVNATPESARIMMEVIRDMG<br>VEKTVGFKPAGGVRTAEDAQKYLAIADELFG<br>ADWADARHYRFGASSLLASLLKALGHGDGK<br>SASSY*	N-Term<br>6xHis	P0A6L0	27.8	14690
EcDERA$_{K172A}$	MTDLKASSLRALKLMDLTTLNDDDTDEKVIAL<br>CHQAKTPVGNTAAICIYPRFIPIARKTLKEQGT<br>PEIRIATVTNFPHGNDDIDIALAETRAAIAYGA<br>DEVDVVFPYRALMAGNEQVGFDLVKACKEA<br>CAAANVLLKVIIETGELKDEALIRKASEISIKAG<br>ADFIKTS**A**GKVAVNATPESARIMMEVIRDMG<br>VEKTVGFKPAGGVRTAEDAQKYLAIADELFG	N-Term<br>6xHis	P0A6L0	27.8	14690

	ADWADARHYRFGASSLLASLLKALGHGDGK SASSY*				
EcDERA$_{L20A/F76A}$	MTDLKASSLRALKLMDLTTANDDDTDEKVIA LCHQAKTPVGNTAAICIYPRFIPIARKTLKEQG TPEIRIATVTNAPHGNDDIDIALAETRAAIAYG ADEVDVVFPYRALMAGNEQVGFDLVKACKE ACAAANVLLKVIIETGELKDEALIRKASEISIKA GADFIKTSTGKVAVNATPESARIMMEVIRDM GVEKTVGFKPAGGVRTAEDAQKYLAIADELF GADWADARHYRFGASSLLASLLKALGHGDG KSASSY*	N-Term 6xHis	P0A6L0	27.8	14690
CfDHAK$_{WT}$	MSQFFFNQRTHLVSDVIDGAIIASPWNNLARL ESDPAIRIVVRRDLNKNNVAVISGGGSGHEP AHVGFIGKGMLTAAVCGDVFASPSVDAVLTA IQAVTGEAGCLLIVKNYTGDRLNFGLAAEKAR RLGYNVEMLIVGDDISLPDNKHPRGIAGTILV HKIAGYFAERGYNLATVLREAQYAASNTFSL GVALSSCHLPQETDAAPRHHPGHAELGMGI HGEPGASVIDTQNSAQVVNLMVDKLLAALPE TGRLAVMINNLGGVSVAEMAIITRELASSPLH SRIDWLIGPASLVTALDMKGFSLTAIVLEESIE KALLTEVETSNWPTPVPPREITCVVSSHASA RVEFQPSANALVAGIVELVTATLSDLETHLNA LDAKVGDGDTGSTFAAAAREIASLLHRQQLP LNNLATLFALIGERLTVVMGGSSGVLMSIFFT AAGQKLEQGANVVEALNTGLAQMKFYGGAD EGDRTMIDALQPALTSLLAQPKNLQAAFDAA QAGAERTCLSSKANAGRASYLSSESLLGNM DPGAQRLAMVFKALAESELG*	C-Term 6xHis	P45510	57.9	27180
PPM$_{WT}$	MKRAFIMVLDSFGIGATEDAERFGDVGADTL GHIAEACAKGEADNGRKGPLNLPNLTRLGLA KAHEGSTGFIPAGMDGNAEVIGAYAWAHEM SSGKDTPSGHWEIAGVPVLFEWGYFSDHEN SFPQELLDKLVERANLPGYLGNCHSSGTVIL DQLGEEHMKTGKPIFYTSADSVFQIACHEET FGLDKLYELCEIAREELTNGGYNIGRVIARPFI GDKAGNFQRTGNRHDLAVEPPAPTVLQKLV DEKHGQVVSVGKIADIYANCGITKKVKATGLD ALFDATIKEMKEAGDNTIVFTNFVDFDSSWG HRRDVAGYAAGLELFDRRLPELMSLLRDDDI LILTADHGCDPTWTGTDHTREHIPVLVYGPK VKPGSLGHRETFADIGQTLAKYFGTSDMEYG KAMF*	N-Term 6xHis	B1XFJ3	44.4	44265

PNP_{WT}	MATPHINAEMGDFADVVLMPGDPLRAKYIAE TFLEDAREVNNVRGMLGFTGTYKGRKISVM GHGMGIPSCSIYTKELITDFGVKKIIRVGSCG AVLPHVKLRDVVIGMGACTDSKVNRIRFKDH DFAAIADFDMVRNAVDAAKALGIDARVGNLF SADLFYSPDGEMFDVMEKYGILGVEMEAAGI YGVAAEFGAKALTICTVSDHIRTHEQTTAAER QTTFNDMIKIALESVLLGDKE*	N-Term 6xHis	P0ABP8	25.9	9190

Table 2: Forwards (for) and reverse (rev) primers used to generate aldolase mutants by quick change mutagenesis.

Name	Primer For (5′-3′)	Primer Rev (5′-3′)
Ec$DERA_{L20A}$	GATGGACCTGACCACCG CGAATGACGACGACACC	GGTGTCGTCGTCATTCGC GGTGGTCAGGTCCATC
Ec$DERA_{V73A}$	CCGTATCGCTACGGCGA CCAACTTCCCACACG	CGTGTGGGAAGTTGGTCG CCGTAGCGATACGG
Ec$DERA_{F76A}$	CGCTACGGTAACCAACG CGCCACACGGTAACGAC G	CGTCGTTACCGTGTGGCG CGTTGGTTACCGTAGCG
Ec$DERA_{I139A}$	TGTACTGCTGAAAGTGG CGATCGAAACCGGC	GCCGGTTTCGATCGCCAC TTTCAGCAGTACA
Ec$DERA_{T170A}$	GCGGACTTCATCAAAGC GTCTACCGGTAAAGTGG CTG	CAGCCACTTTACCGGTAG ACGCTTTGATGAAGTCCG C
Ec$DERA_{K172A}$	CTTCATCAAAACCTCTGC GGGTAAAGTGGCTGTGA ACG	CGTTCACAGCCACTTTACC CGCAGAGGTTTTGATGAA G
Ec$DERA_{L20A/F76A}$	GATGGACCTGACCACCG CGAATGACGACGACACC	CGTCGTTACCGTGTGGCG CGTTGGTTACCGTAGCG

4. Synthesis of Aldehyde Donors from Diacetals

Some aldehyde donors were only available as diacetal derivatives (methoxy acetaldehyde and methoxy(ethoxy)acetaldehyde). These were converted to the free aldehydes by hydrolysis with Dowex Acidic Resin.

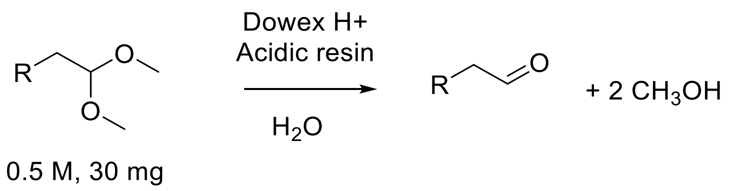

Methoxy Acetaldehyde

To a 1.5 µL Eppendorf, 500 µL of a 0.5 M solution of methoxyacetaldehyde dimethyl acetal (Tokyo Chemical Industry, 30 mg, 0.25 mmoles) was made up in water. To this, 30 mg of Dowex Acidic Resin H+ form was added. The reaction was left shaking at 750 rpm, 70 °C for 30 minutes. The reaction was then filtered, giving a mixture of aldehyde donor and methanol. This reaction mixture was used directly in the biotransformations, without any further purification.

^{1}H NMR (400 MHz): 3.26 (s, methanol biproduct) 3.32 (s, 3H, CH3) 3.35 (d, J= 5.1 Hz, 2H, CH2) 5.07 (t, J= 5.1 Hz, 1H, hydrate of aldehyde proton)

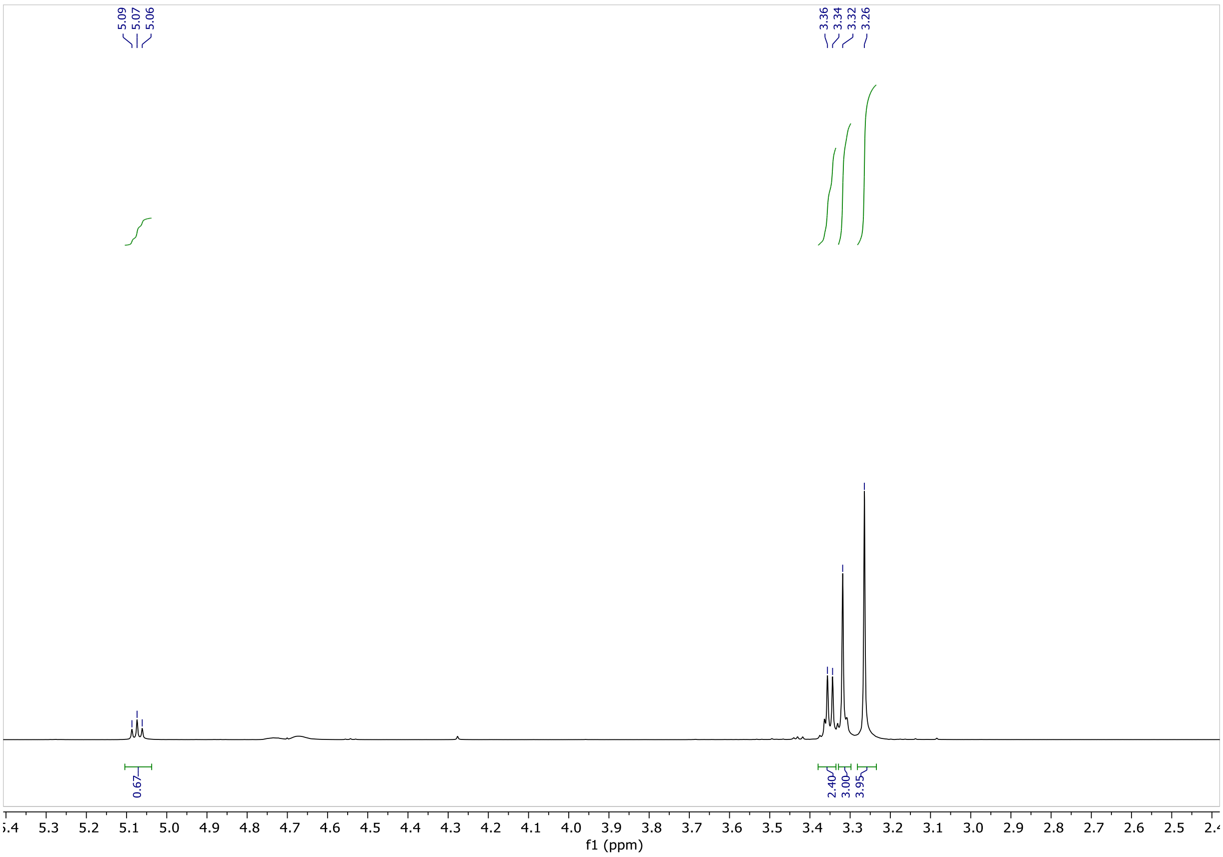

Figure 2: ^{1}H NMR of methoxyacetaldehyde deprotection reaction mixture.

Methoxy (Ethoxy) Acetaldehyde

To a 1.5 µL Eppendorf, 500 µL of a 0.5 M solution of Methoxy(ethoxy) acetaldehyde dimethyl acetal (Tokyo Chemical Industry, 41 mg, 0.25 mmoles) was made up in water. To this, 40 mg of Dowex Acidic Resin H+ form was added. The reaction was left shaking at 750 rpm, 70 °C for 30 minutes. The reaction was then filtered, giving a mixture of aldehyde donor and methanol. This reaction mixture was used directly in the biotransformations, without any further purification.

^{1}H NMR (400 MHz): 3.26 (s, methanol biproduct) 3.30 (s, 3H, CH3) 3.42 (d, J = 4.91 Hz, 2H, CH2, 3.55 (m, 2H, CH2) 3.63 (m, 2H, 2H) 5.08 (t, J = 4.91 Hz, 1H, aldehyde proton hydrate)

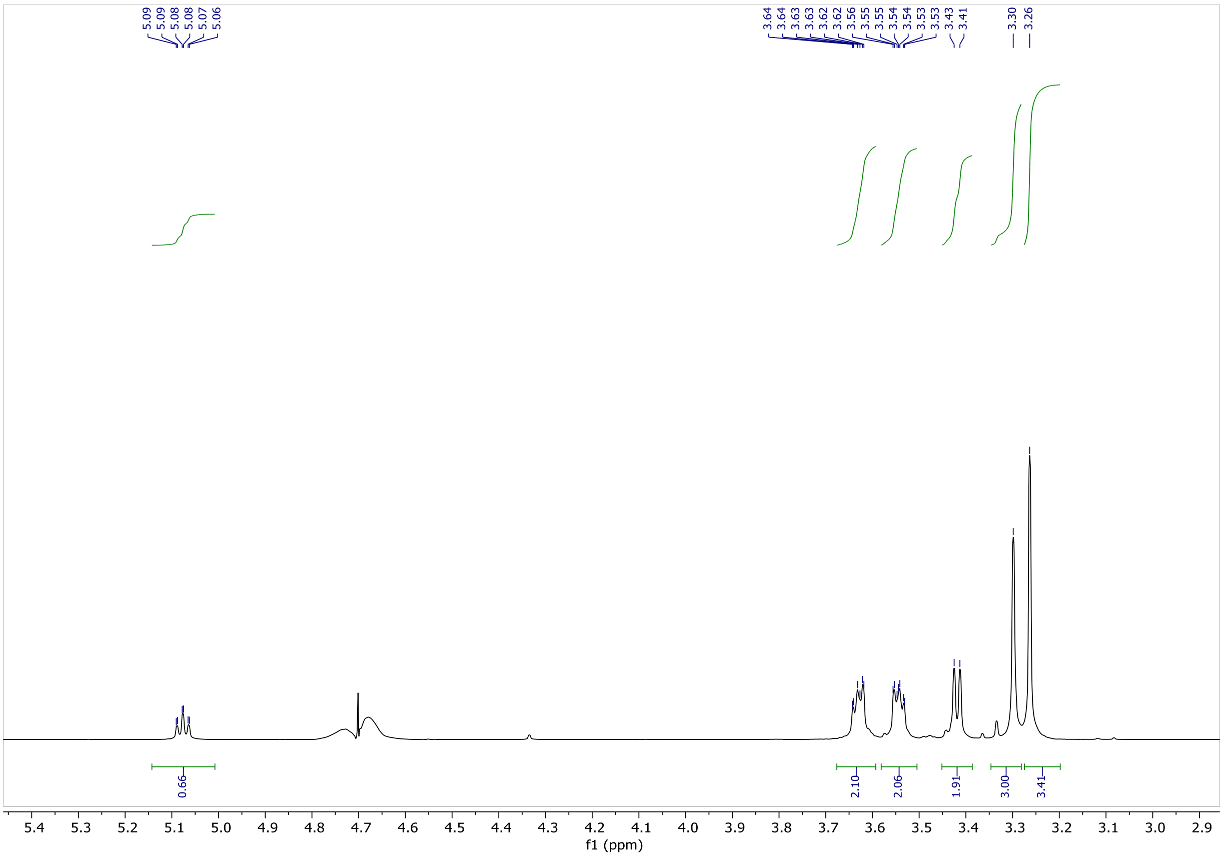

Figure 3: ¹H NMR of methoxy(ethoxy) acetaldehyde reaction mixture

5. HPLC Analysis of Compounds

5.1 HPLC Analysis of Sugar Phosphates

Calculation of Product Formation

As glyceraldehyde-3-phosphate is unstable under the conditions of the enzymatic reaction, starting material consumption is an unsuitable measure of reaction progress. In addition to this functionalised pentose-5-phosphates are unavailable commercially. Therefore, to

measure reaction progress, product formations were worked out by comparison to D-ribose-5-phosphate (the only commercially available product) via calibration curve. For simplicity these have been referred to as “conversion” throughout the main text. This method relies on the assumption that all products derivatised with *O*-benzylhydroxylamine would give a similar response factor. This assumption is true for all substrates, with the exception of benzoylacetaldehyde, where the additional benzyl group will cause an increase in absorbance compared to the D-Ribose-5-Phosphate standard. For these products, concentrations were instead calculated using a calibration curve of the purified product after a preparative scale synthesis.

Derivatisation Protocol

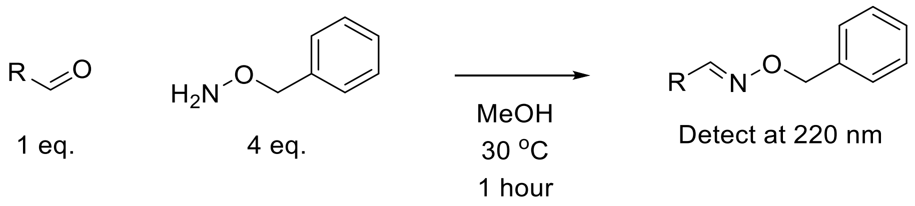

Samples were derivatized with a 4x excess of *O*-benzylhydroxylamine.HCl (based on the total concentration of aldehyde donor and acceptor added). Typically, an equal volume of 100 mM *O*-benzylhydroxylamine solution in MeOH was added directly to biotransformation reaction mixtures. The reactions were left shaking at 30 °C for 1 hour, filtered and analysed directly by HPLC method 1. For reactions at higher substrate loading, to prevent overloading of the detector, samples were diluted down to 5 mM concentration and then derivatised. For the majority of products only the E isomer of the imine was observed, smaller donor aldehydes appeared as two peaks corresponding to a mixture of E/Z isomers after derivatisation.

HPLC Method 1

Buffer A: 0.1% Difluoroacetic acid in Water

Buffer B: 0.1% Difluoroacetic acid in Acetonitrile

Column: Agilent Poroshell 120 C18

Injection = 5 µL

Method:

Time (mins)	Buffer A (%)	Buffer B (%)
0	95	5

5.00	50	50
5.05	5	95
5.3	5	95
5.4	95	5
6	95	5

Table 3: D-ribose-5-phosphate calibration curve

Concentration (mM)	Peak Area at 220 nm		
1	1132	1140	1129
2.5	2783	2828	2788
5	5414	5398	5396
7.5	8055	7933	8020
10	10352	10381	10341

Table 4: 2-deoxy-2-benzoxyl-D-ribose-5-phosphate calibration curve

Concentration (mM)	Peak Area at 220 nm		
0.66	1277	1279	1274
1.65	3161	3049	3141
3.33	5704	5716	5723
4.95	8271	8268	8270
6.6	10565	10542	10616

Conversions were calculated using the following formula:

$$\left(\frac{A/C}{S}\right) \times 100\%$$

Where A = peak area from product in HPLC at 220 nm

C = slope of calibration curve, typically using Ribose-5-Phosphate as a product standard

S = concentration of limiting reagent, typically the aldehyde acceptor at 5 mM concentration.

Representative HPLCs traces for example substrates have been included for each of the cascades. Retention times may shift between different sets of reactions – this is a result of changing HPLC guard columns.

5.2 HPLC Analysis of Nucleosides

HPLC analysis of nucleosides was carried out by HPLC, using peak areas of starting materials and products. To calculate conversions, it was assumed the ratio absorbance for the nucleoside and nucleobase at 254 nm was 1:1. “Conversions” were defined as the area of desired nucleoside product divided by the total area of nucleobase containing compounds. This included both the limiting nucleobase but also any nucleoside side-products formed from product degradation (such as the breakdown of adenosine analogues into inosine analogues by adenosine deaminase). Conversions were calculated using the following formula:

$$Conversion = \frac{An}{(An + Ab\ +\ As)} \times 100\%$$

Where:

An = area of nucleoside product

Ab = area of nucleobase starting material

As = area of nucleoside side products

To separate the nucleosides and the nucleobases three additional HPLC Methods were used:

HPLC Method 2

Used for analysis of: Adenosine, 2-Me Adenosine reactions

Mobile Phase A: 95% Water 5% Acetonitrile 10 mM Ammonium Formate pH 4

Mobile Phase B: 5% Water 95% Acetonitrile 10 mM Ammonium Formate pH 4

Column = Agilent Poroshell 120 C18

Injection = 5 μL

Method: isocratic A 100% (5 mins)]

HPLC Method 3:

Used for Analysis of 2-F-Adenosine Reactions

Mobile Phase A = 20 mM Ammonium Formate pH 6

Mobile Phase B = Acetonitrile

Column = Atlantis T3

Injection = 2 µL

Method:

Time (mins)	A%	B%
0	97	3
2	97	3
3	50	50
3.5	50	50
4	97	3
5	97	3

HPLC Method 4:

Used for Analysis of Guanosine reactions

Mobile Phase A = 20 mM Ammonium Formate pH 6

Mobile Phase B = Acetonitrile

Column = Atlantis T3

Injection = 2 µL

Method:

Time	A%	B%
0	95	5
5	5	95

5.1	95	5
6	95	5

HPLC Method 5

Used for analysis of Vidarabine Reactions

Mobile Phase A = 20 mM Ammonium Formate pH 3

Mobile Phase B = Acetonitrile

Column = Atlantis T3

Injection = 2 μL

Method:

Time	A%	B%
0	95	5
5	5	95
5.1	95	5
6	95	5

6. Initial Mutant Screen

6.1 Example HPLC Trace

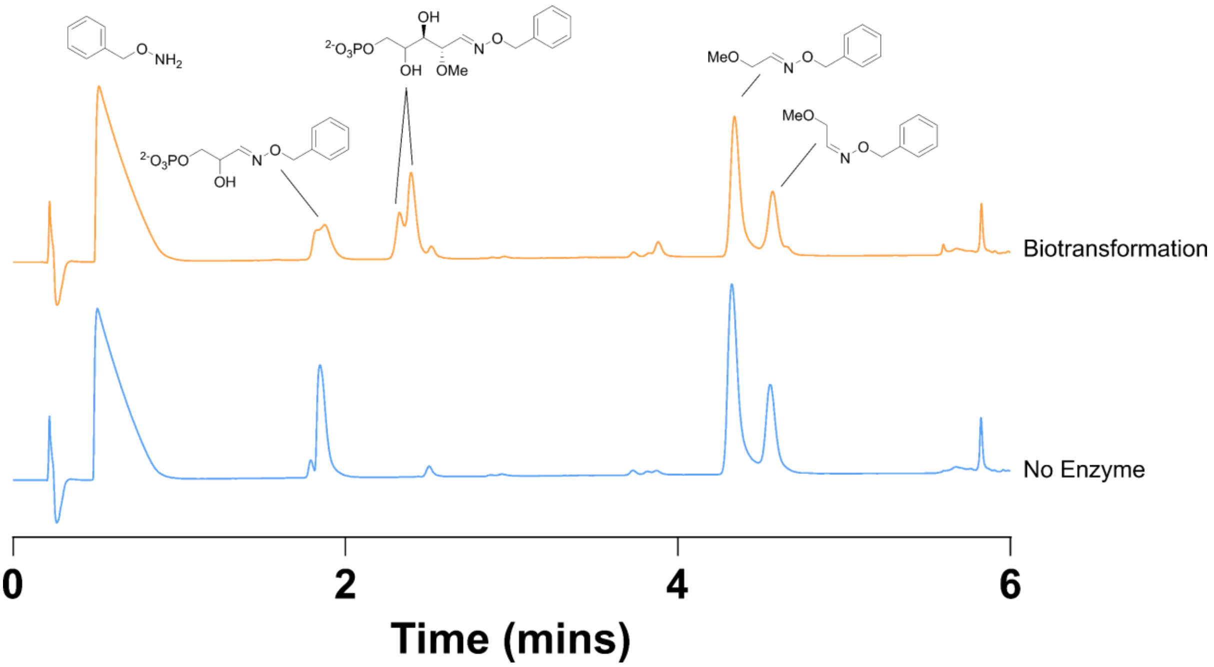

Figure 4: Example HPLC trace for methoxyacetaldehyde biotransformation, and no enzyme control. Derivatised structures of shown for each peak.

Table 5: RP-HPLC retention times for DERA biotransformations with DL-G3P. Due to slight shifts between the different cascades after changing guard columns, retention times have been normalised using G3P as an internal standard.

Underivatised Structure	Derivatised structure	Compound Number	Retention Time
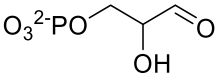	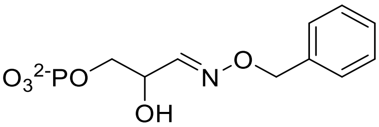	**1**	1.84
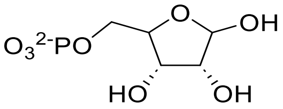	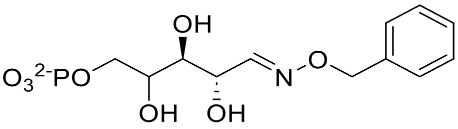	**3**	1.48
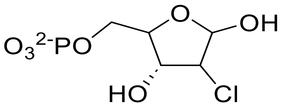	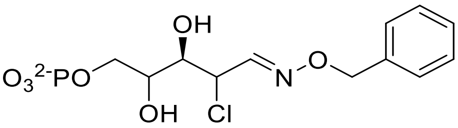	**4**	2.55, 2.66, 2.73, 2.80
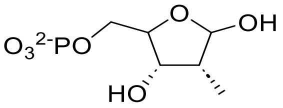	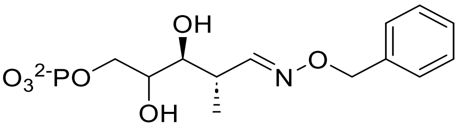	**5**	2.38, 2.45
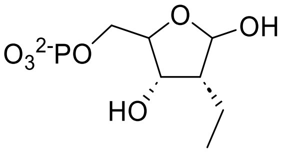	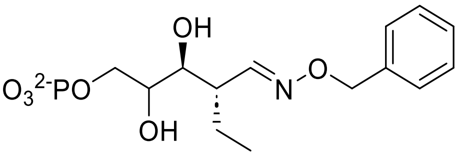	**6**	2.79, 2.90
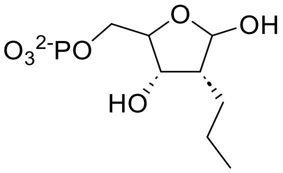	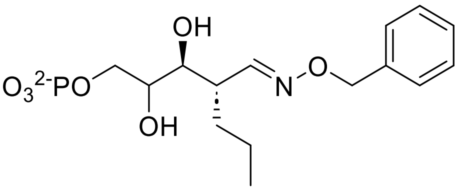	**7**	3.29, 3.39
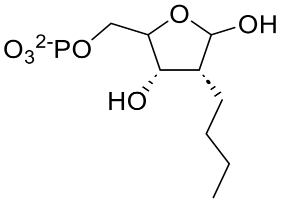	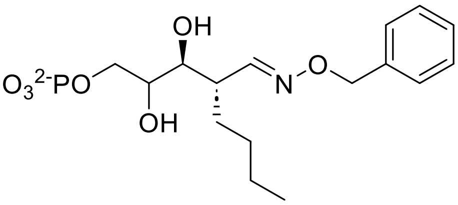	**8**	3.76, 3.86
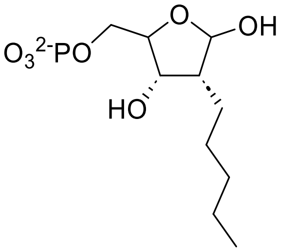	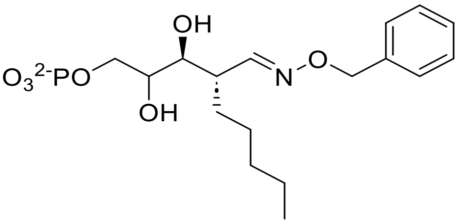	**9**	4.22, 4.32

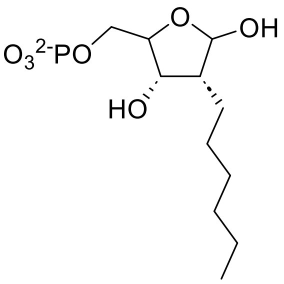	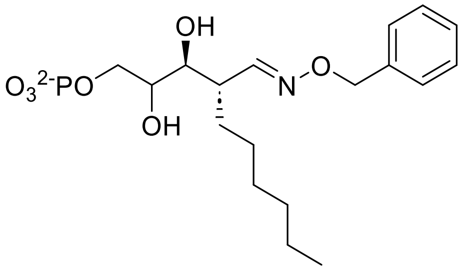	**10**	4.66, 4.75
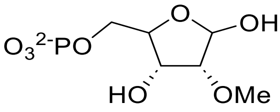	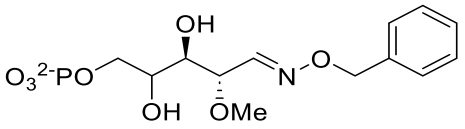	**11**	2.32, 2.40
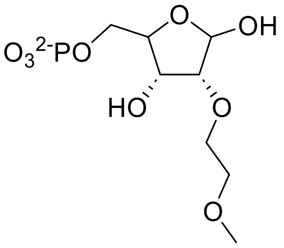	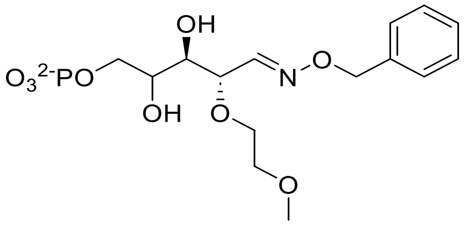	**12**	2.68, 2.70
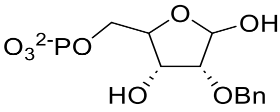	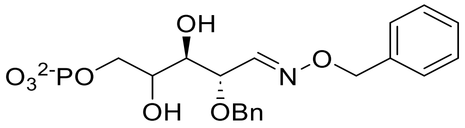	**13**	3.95, 4.00

6.2 Substrate Screen

A panel of aldehyde donors was screened towards the DERA variants alongside aldolase Wild-Types (*Ec*DERA, *Ac*DERA, *Ec*FSA). For each of the aldehyde donors a mass spectrum of the derivatised product alongside a table of HPLC product areas has been included.

6.2.1 Glycolaldehyde donor (product 3)

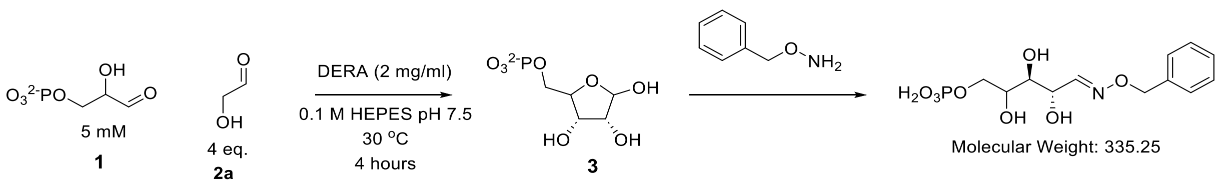

Table 6: Peak areas for glycolaldehyde donor reactions

Enzyme	Peak Area	Product Formation
FSA WT	4213.7 ± 27.9	80.0 ± 0.7
DERA AC		
DERA WT	4175.7 ± 97.1	79 ± 2.2
DERA L20A	2863 ± 44.7	54 ± 1.0
DERA V73A	2611 ± 94.1	50 ± 2.2
DERA F76A	3864.7 ± 43.1	73 ± 1.1
DERA I139A	200.33 ± 4.01	4 ± 0.1
DERA T170A	1240.3 ± 42.9	24 ± 1
DERA K172A	1731.3 ± 24.1	34 ± 0.6
DERA L20A/F76A	1488 ± 12.8	28 ± 0.3

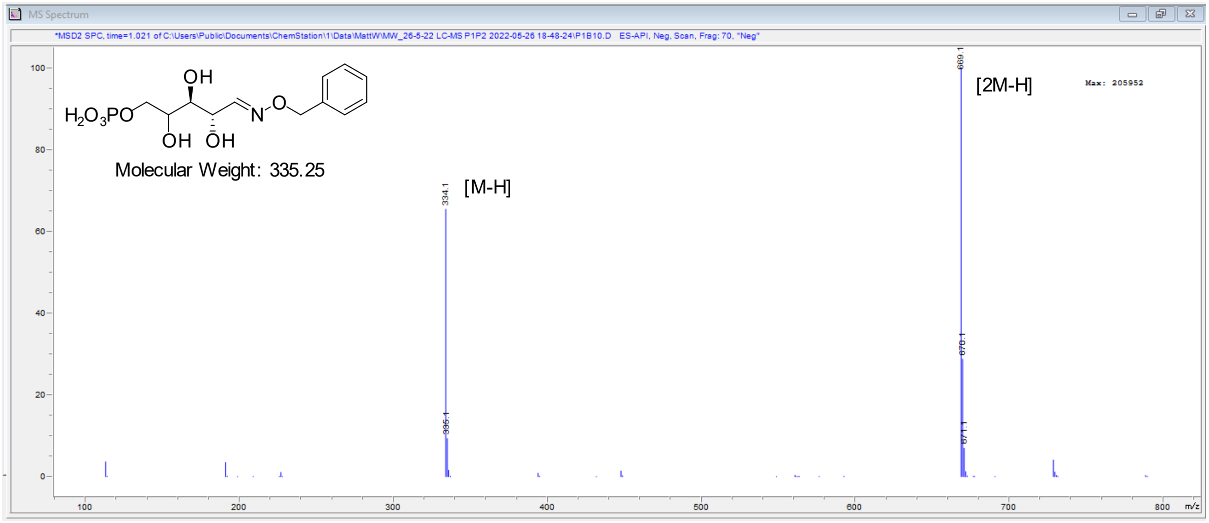

*Figure 5: ESI- mass spectrum of derivatised 2-OH product (**3**), [M-H] = 334.1*

6.2.2 Chloroacetaldehyde donor (product 4)

Reaction Scheme:

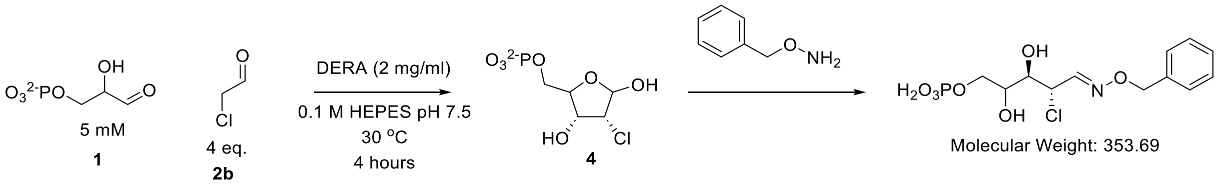

Table 7: Peak areas for chloroacetaldehyde donor reactions

Enzyme	Peak Area	Product Formation
FSA WT	451 ± 2.62	9 ± 0.1
DERA AC WT		
DERA EC WT	2317 ± 107	45 ± 2.5
DERA L20A	2613 ± 32.0	50 ± 0.8
DERA V73A	2092 ± 102	37 ± 2.4
DERA F76A	1973 ± 34.8	38 ± 0.8
DERA I139A	594 ± 209.6	6 ± 4.8
DERA T170A	2045 ± 32.9	39 ± 0.8
DERA K172A	2246 ± 64.2	43 ± 1.5
DERA L20A/F76A	524 ± 2.05	10 ± 0.1

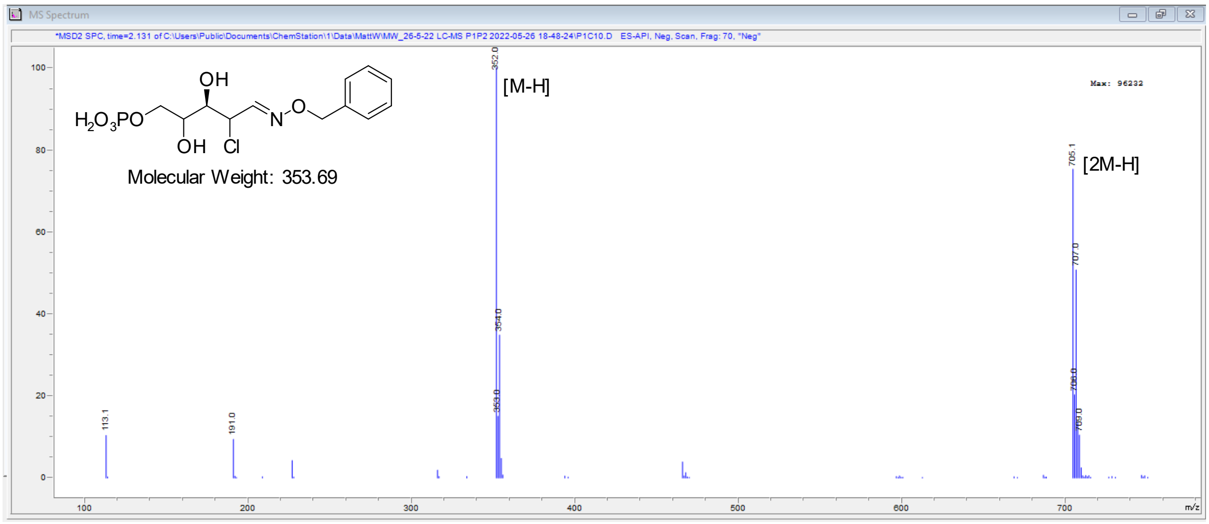

*Figure 6: ESI- mass spec of derivatised 2-Cl product (**4**), [M-H] = 352.0*

6.2.3 Propanal donor (product 5)

Reaction Scheme

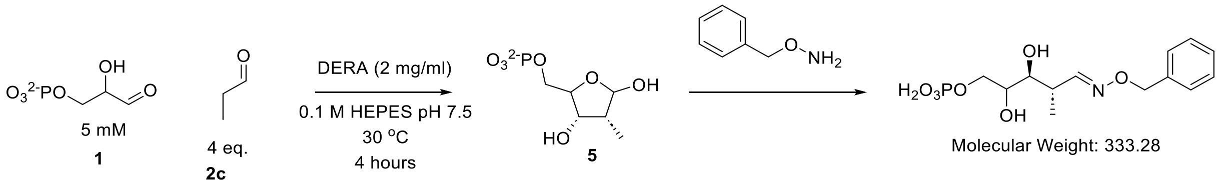

Table 8: Peak areas for propanal donor reactions

Enzyme	Peak Area	Product Formation
FSA WT	749.3 ± 22.2	14 ± 0.5
DERA AC WT		69 ± 1.0
DERA WT	3542 ± 94.4	67 ± 2.2
DERA L20A	3063 ± 27.9	58 ± 0.4
DERA V73A	3121 ± 46.8	59 ± 1.1
DERA F76A	3095 ± 169	59 ± 3.9
DERA I139A	916.7 ± 14.5	17.4 ± 0.4
DERA T170A	2710 ± 22.7	51 ± 0.5
DERA K172A	2853 ± 56.4	54 ± 1.3
DERA L20A/F76A	3224 ± 7.95	61 ± 0.2

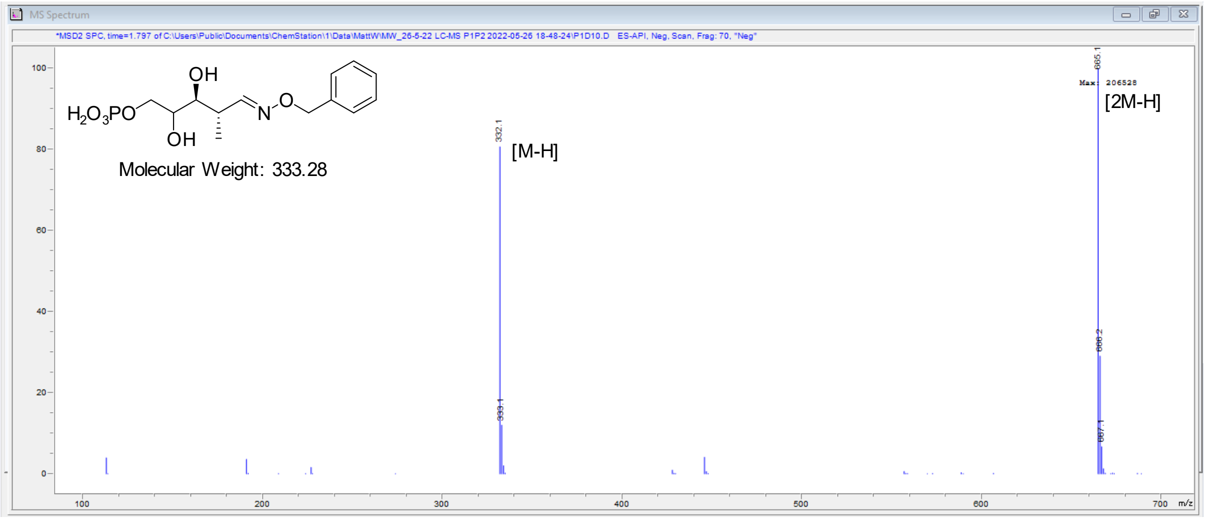

*Figure 7: ESI- mass spectrum of derivatised 2-Me product (**5**), [M-H] = 332.1*

6.2.4 Butanal donor (product 6)

Reaction Scheme

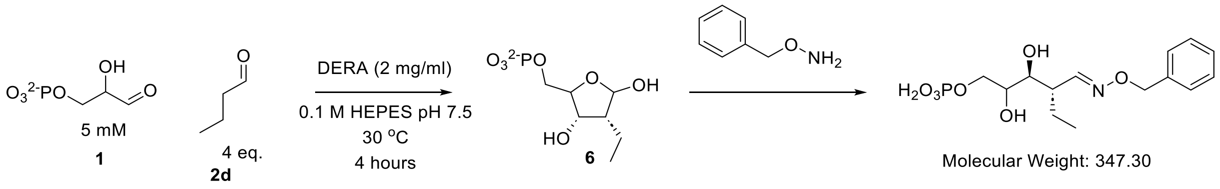

Table 9: Peak areas for butanal donor reactions

Enzyme	Peak Area	Product Formation
FSA WT	0	0
		7 ± 0.1
DERA WT	633.7 ± 92.3	12 ± 2.2
DERA L20A	2422 ± 3.30	46 ± 0.1
DERA V73A	1880 ± 168	36 ± 3.8
DERA F76A	2272 ± 192	43 ± 4.4
DERA I139A	0	0
DERA T170A	930.3 ± 9.88	18 ± 0.2
DERA K172A	744.7 ± 30.8	14 ± 0.7
DERA L20A/F76A	3191 ± 51.7	61 ± 1.2

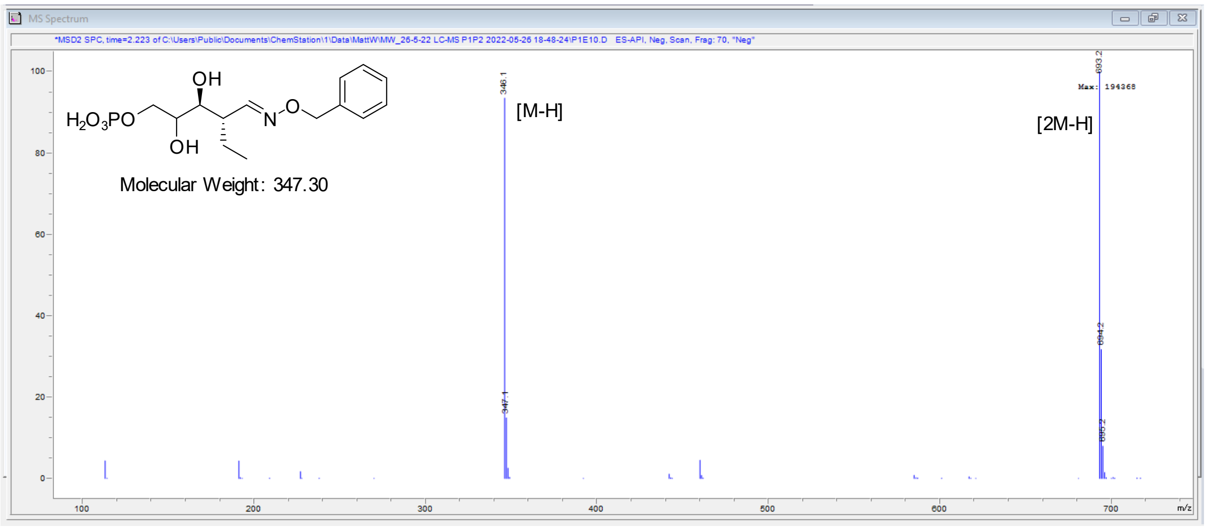

*Figure 8: ESI- mass spectrum of derivatised 2-Et product (**6**), [M-H] = 346.1*

6.2.5 Pentanal donor (product 7)

Reaction Scheme

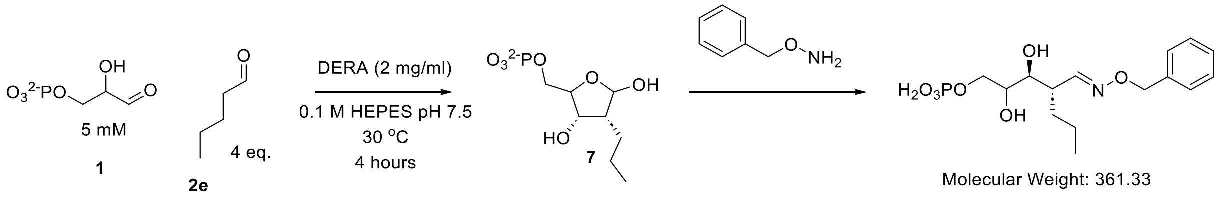

Table 10: peak areas for pentanal donor reactions

Enzyme	Peak Area	Mean Conversion
FSA WT	0	0
DERA WT	0	0
DERA L20A	854.3 ± 92.3	16 ± 2.1
DERA V73A	157 ± 4.24	3 ± 0.1
DERA F76A	1918 ± 259	36 ± 6
DERA I139A	0	0
DERA T170A	0	0
DERA K172A	0	0
DERA L20A/F76A	3563 ± 33.5	68 ± 0.8

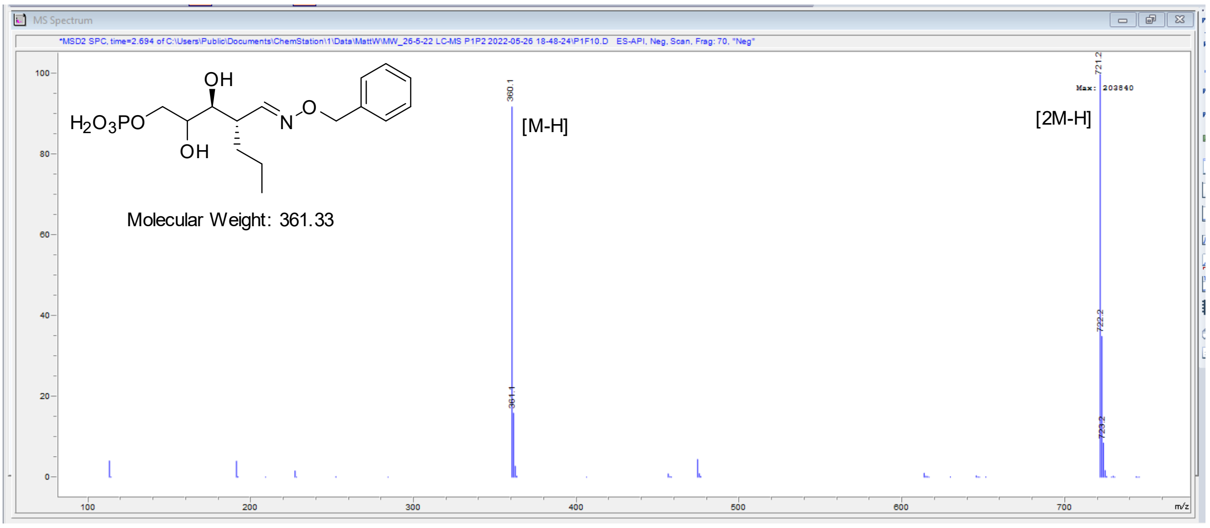

*Figure S9: ESI- mass spectrum of derivatised 2-iPr product (**7**), [M-H] = 360.1*

6.2.6 Hexanal donor (product 8)

Reaction Scheme

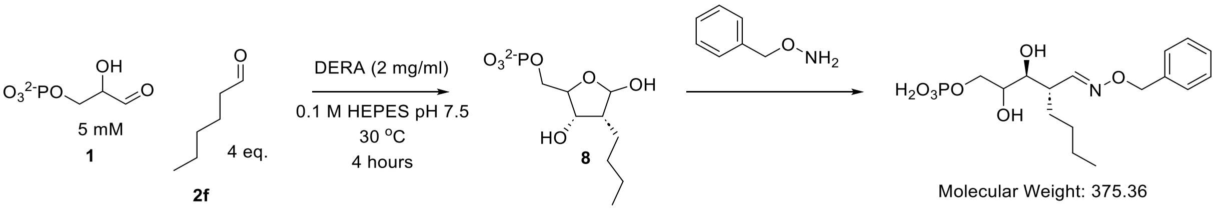

Table 11: peak areas for hexanal donor reactions

Enzyme	Peak Area	Mean Conversion
FSA WT	0	0
DERA WT	0	0
DERA L20A	1741 ± 5.8	33 ± 0.2
DERA V73A	0	0
DERA F76A	2551 ± 159	48 ± 3.7
DERA I139A	0	0
DERA T170A	0	0
DERA K172A	0	0
DERA L20A/F76A	3478 ± 33	66 ± 0.8

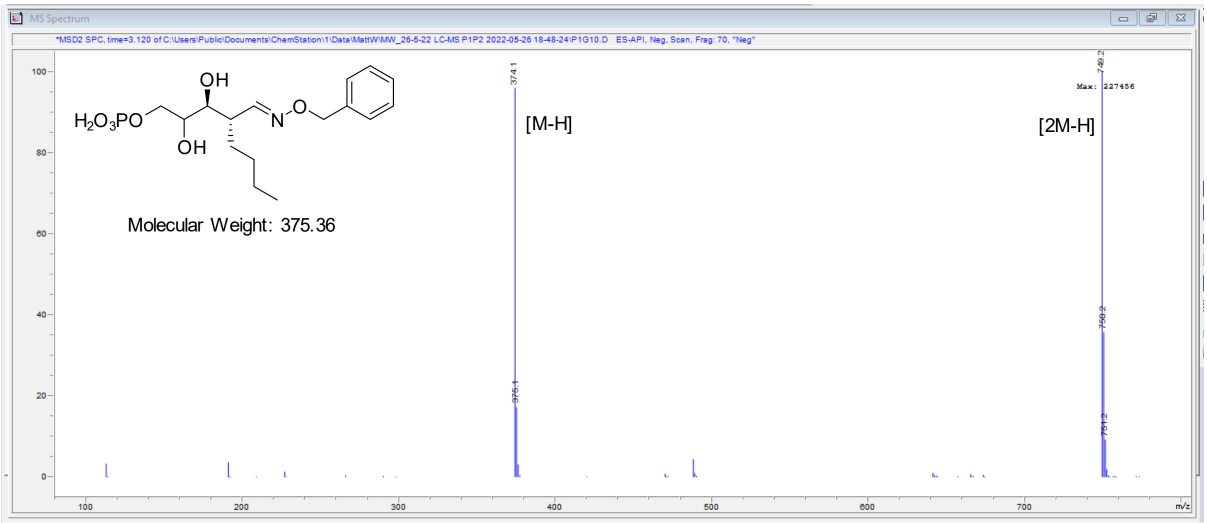

*Figure 10: ESI- mass spectrum of derivatised 2-Bu product (**8**), [M-H] = 374.1*

6.2.7 Heptanal donor (product 9)

Reaction Scheme

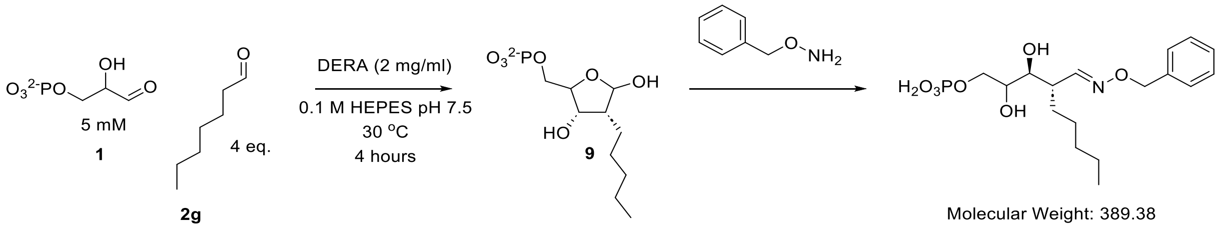

Table 12: peak areas for heptanal donor reactions

Enzyme	Peak Area	Mean Conversion
FSA WT	0	0
DERA WT	0	0
DERA L20A	1531 ± 71.5	28.0 ± 1.7
DERA V73A	0	0
DERA F76A	1623 ± 77.6	33 ± 1.8
DERA I139A	0	0
DERA T170A	0	0
DERA K172A	0	0
DERA L20A/F76A	2644 ± 55.0	49 ± 1.3

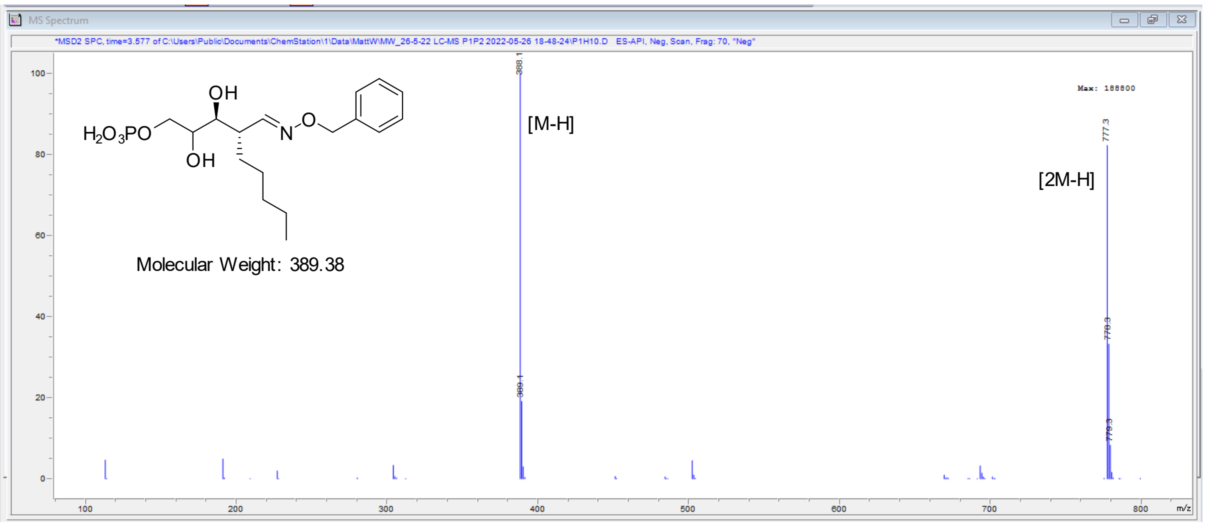

*Figure 11: ESI- mass spectrum of derivatised 2-pentyl product (**9**), [M-H] = 388.1*

6.2.8 Octanal donor (product 10)

Reaction Scheme

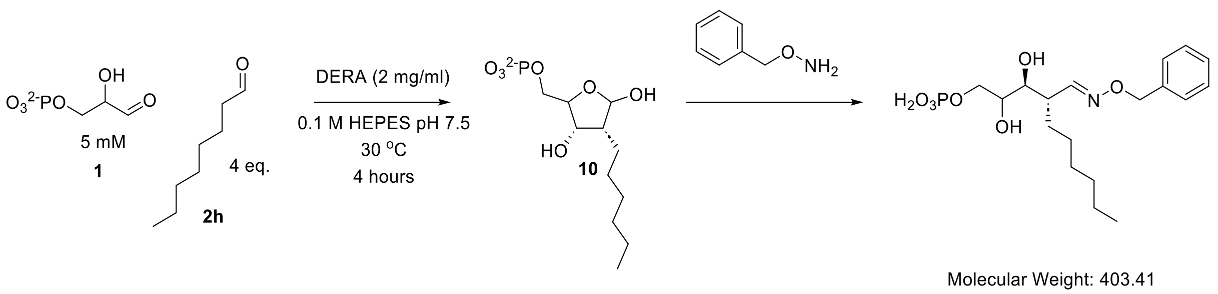

Table 13: peak areas for octanal donor reactions

Enzyme	Peak Area	Mean Conversion
FSA WT	0	0
DERA WT	0	0
DERA L20A	724 ± 34.6	14 ± 0.7
DERA V73A	0	0
DERA F76A	1088 ± 64.3	21 ± 1.5
DERA I139A	0	0
DERA T170A	0	0
DERA K172A	0	0
DERA L20A/F76A	2886 ± 81.6	45 ± 1.9

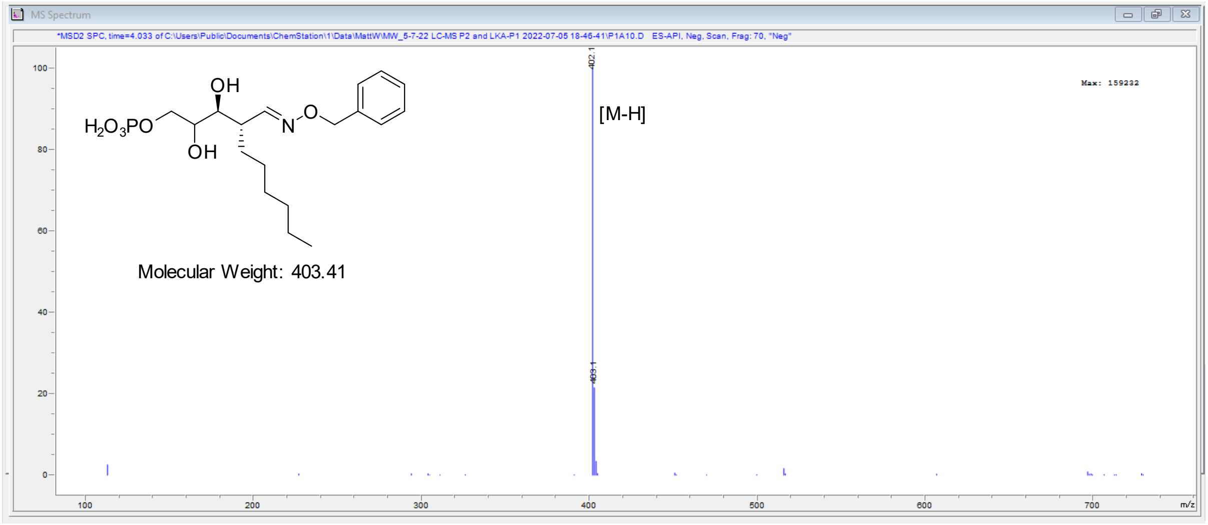

*Figure 12: ESI- mass spectrum of derivatised 2-hexyl product (**10**), [M-H] = 402.1*

6.2.9 Methoxyacetaldehyde donor (product 11)

Reaction Scheme

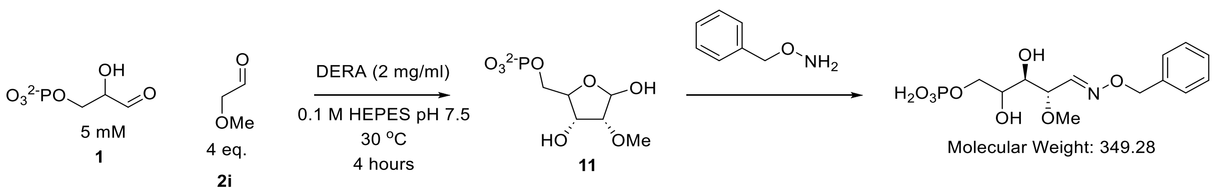

Table 14: peak areas for methoxyacetaldehyde donor reactions

Enzyme	Peak Area	Mean Conversion
FSA WT	0	0
DERA WT	0	0
DERA L20A	1139 ± 55.4	22 ± 1.3
DERA V73A	0	0
DERA F76A	2705 ± 146.5	51 ± 3.3
DERA I139A	0	0
DERA T170A	0	0
DERA K172A	0	0
DERA L20A/F76A	2335 ± 13.6	44 ± 0.3

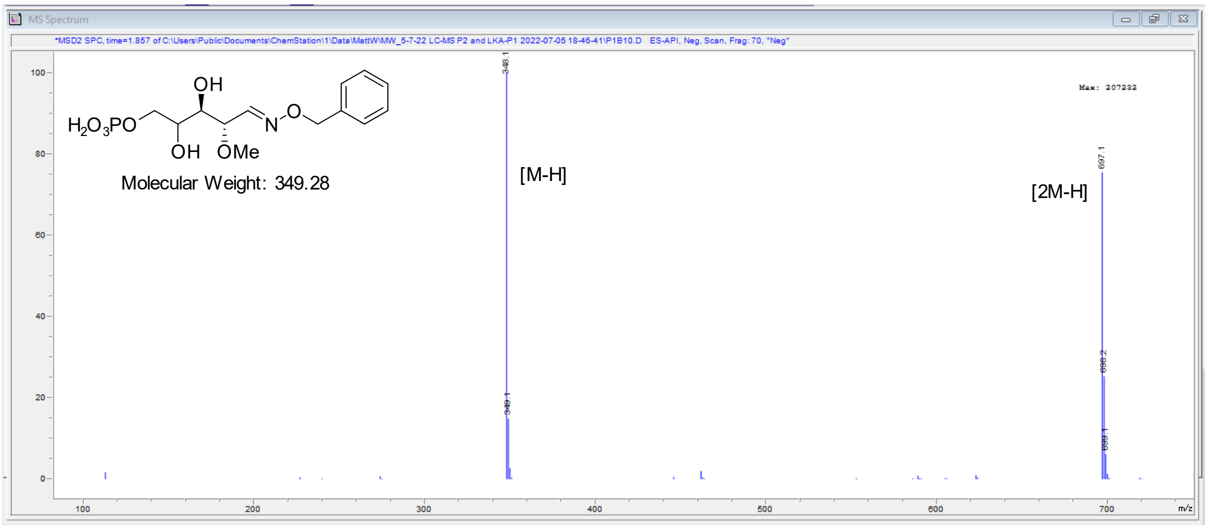

*Figure 13: ESI- mass spectrum of derivatised 2-OMe product (**11**), [M-H] = 348.1*

6.2.10 Methoxy(ethoxy) acetaldehyde donor (product 12)

Reaction Scheme

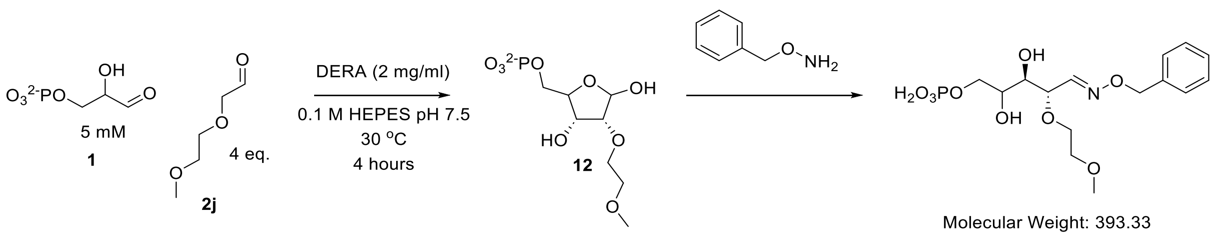

Table 15: peak areas for methoxy(ethoxy) acetaldehyde donor reactions

Enzyme	Peak Area	Mean Conversion
FSA WT	0	0
DERA WT	0	0
DERA L20A	132.7 ± 4.1	3 ± 0.1
DERA V73A	0	0
DERA F76A	2715 ± 95.4	52 ± 2.2
DERA I139A	0	0
DERA T170A	0	0
DERA K172A	0	0
DERA L20A/F76A	3733 ± 158.5	66 ± 4.9

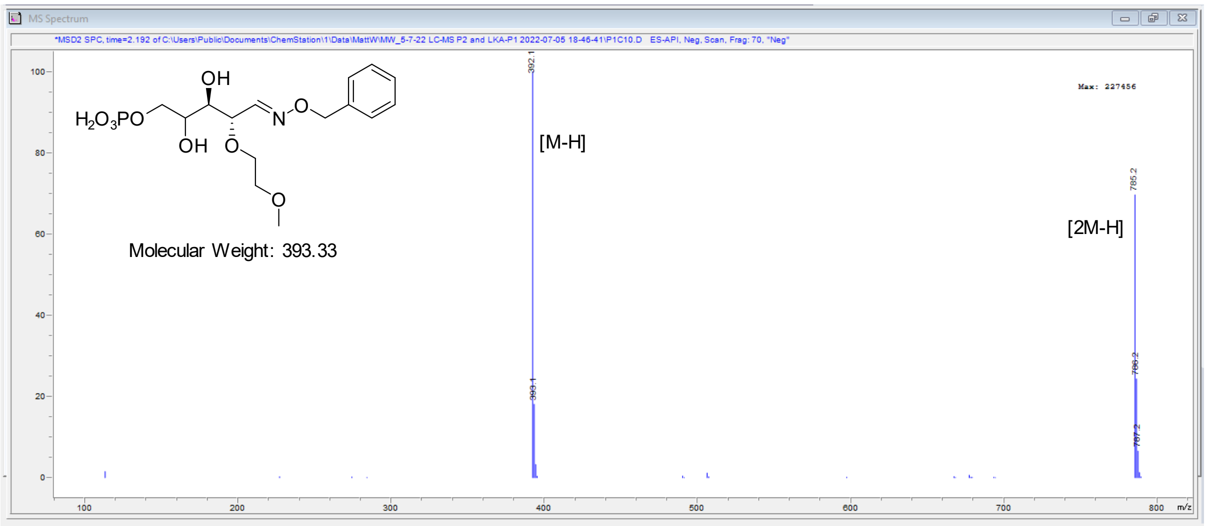

*Figure 14: ESI - mass spectrum of derivatised 2-MOE product (**12**), [M-H] = 392.1*

6.2.11 Benzyoxyacetaldehyde donor (product 13)

Reaction Scheme

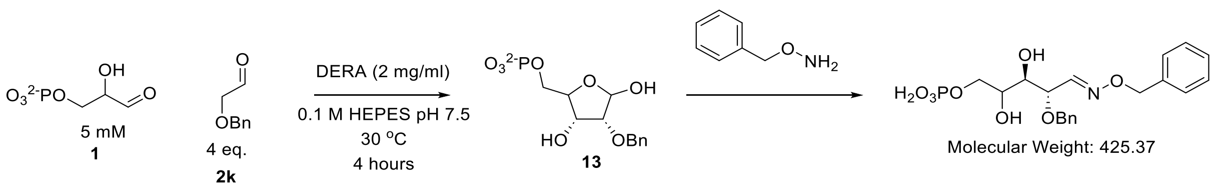

Table 16: peak areas for benzoxylacetaldehyde donor reactions

Enzyme	Peak Area	Mean Conversion
FSA WT	0	0
DERA WT	0	0
DERA L20A	166 ± 4.9	±
DERA V73A	0	0
DERA F76A	3420 ± 161	±
DERA I139A	0	0
DERA T170A	0	0
DERA K172A	0	0
DERA L20A/F76A	4991 ± 81.7	±

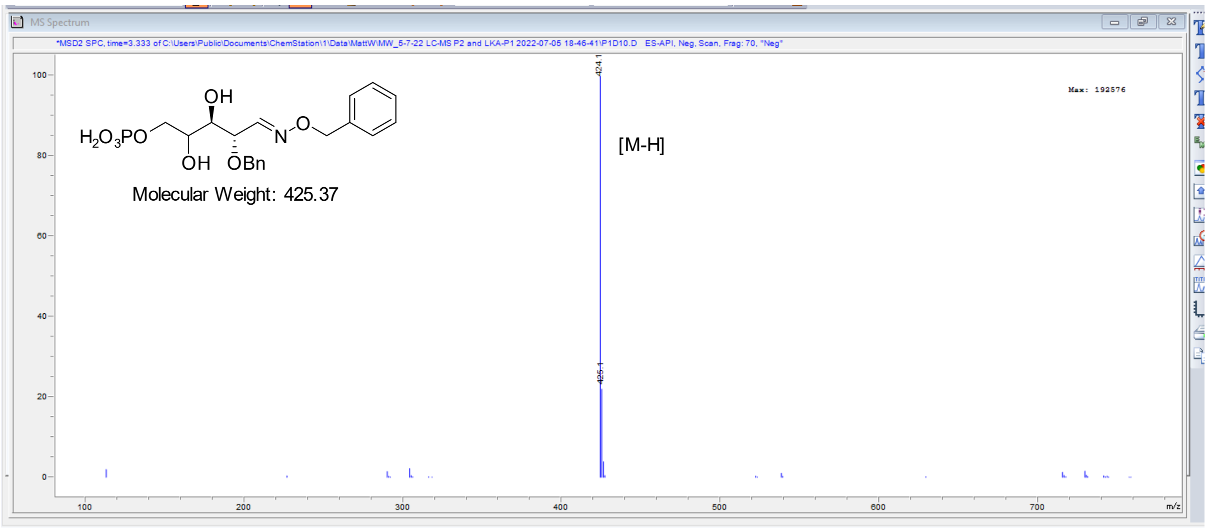

*Figure 15: ESI - mass spectrum of derivatised 2-OBn product (**13**), [M-H] = 424.1*

6.2.12 O-TBDMS acetaldehyde donor (product 14)

Reaction Scheme:

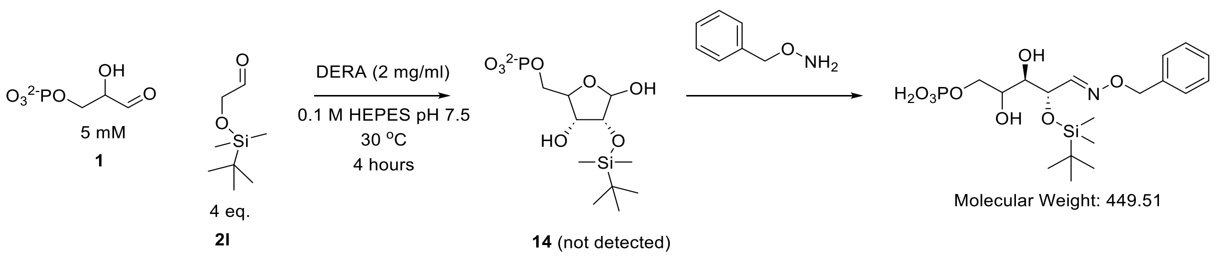

No distinguishable difference between the biotransformation and the enzyme-free control was observed for any of the variants screened.

No product masses for the expected product were detected by LC-MS.

6.3 Aldolase Reaction Equilibrium

6.3.1 Equilibrium Constant

To calculate the equilibrium constant for the aldolase reaction the eQuilibrator webserver was used[1]. While the majority of products for this reaction were not present in the biological databases that equilibrator uses, the synthesis of D-ribose-5-phosphate (product **3**) was used as a model system. Calculation of this equilibrium constant gave a value of 5.4 x 10^2 suggesting the reaction should significantly favour product formation. While the equilibrium constants for the other reactants will differ from this number, they are likely to similarly favour nucleoside formation.

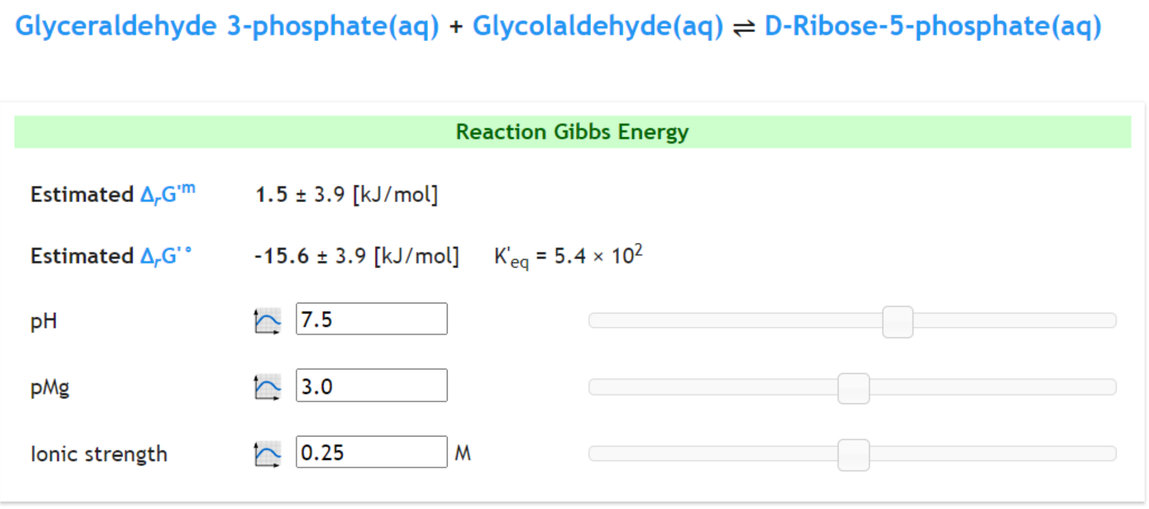

Figure 16: Screenshot taken from https://equilibrator.weizmann.ac.il/ (14/6/24) showing the calculated equilibrium constant for this model reaction.

6.3.2 Time Course

To determine whether longer reaction times would generate conversions closer to the calculated thermodynamic equilibrium of the reaction, a time course using heptanal as a model substrate, generating product **9**, was carried out. This time course was ran over 5 hours with a further time point taken after overnight reaction (18 hours). An increase in conversion was seen over the first 4 hours, with conversion starting to fall after 5 hours. After 18 hours the conversion was significantly reduced. This can be attributed to the irreversible chemical breakdown of glyceraldehyde-3-phosphate into methylglyoxal and free phosphate[2], shifting the equilibrium away from the desired products. This breakdown of glyceraldehyde-3-phosphate can also be observed in the enzyme-free control.

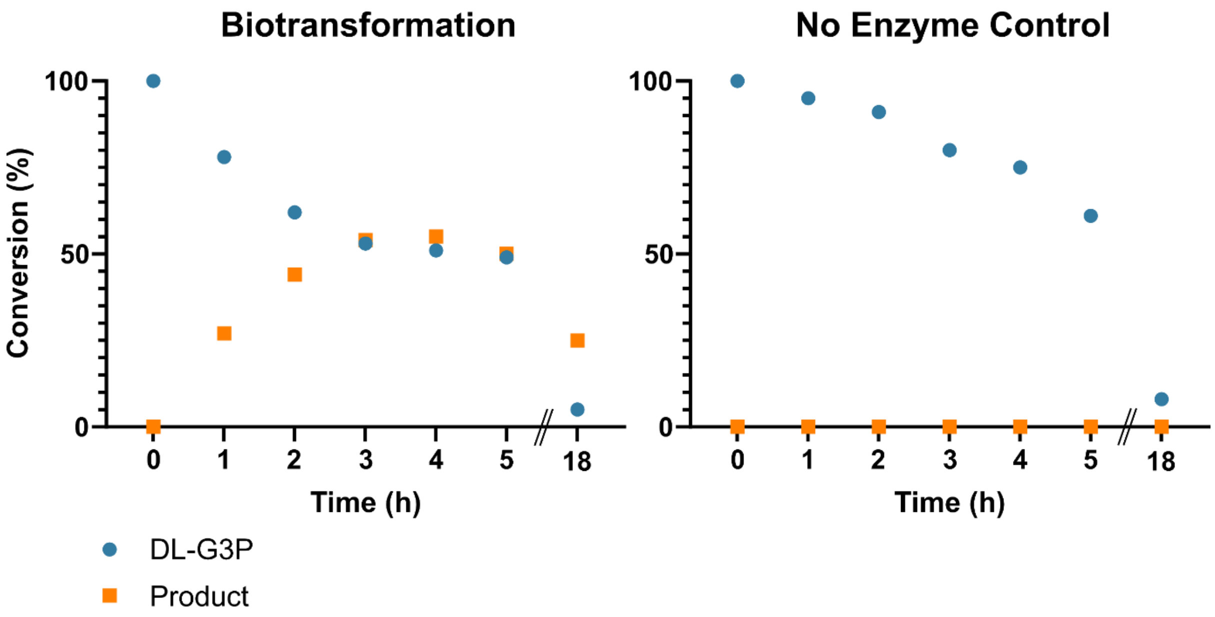

Figure 17: Time course following the generation of product X via DERA. An enzyme-free control has also been included as a comparison.

7. Computational Methods

For each of the three active mutants, models were generated by making *in silico* mutations to the wild type PDB, these models were then energy minimised using Yasara. The models were analysed by fpocket to determine the volume of the active-site and a representative 2-functionalised analogue (methoxyethoxy) was covalently docked into the active-site using Yasara.

7.1 FPocket

The substrate was removed, and binding pockets and volumes were calculated using fpocket 2.0[3]. For each enzyme the binding pocket corresponding to the active-site was selected by visual inspection and the volume recorded. Default settings were used. Active-site pockets and their volumes are shown below.

WT

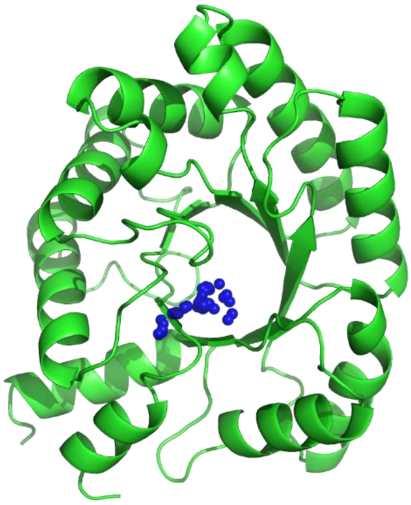

Figure 18: WT binding pocket

L20A

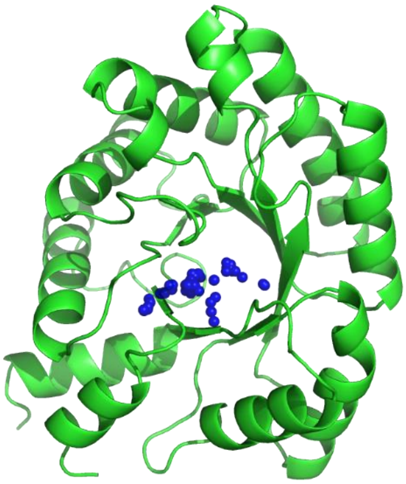

Figure 19: L20A binding pocket

F76A

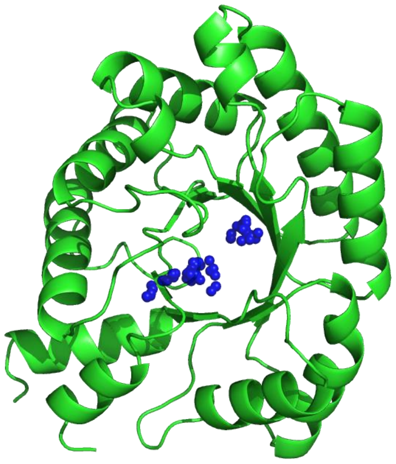

Figure 20: F76A binding pocket

L20A/F76A

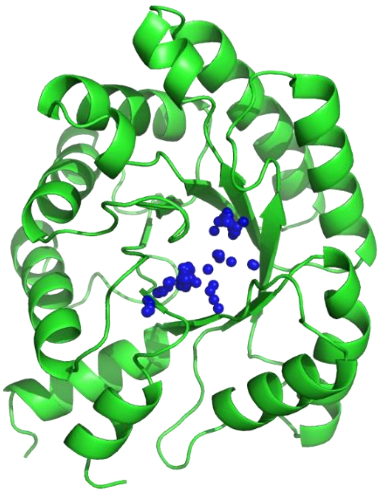

Figure 21: L20A/F76A binding pocket

Pocket Volumes:

Table 17: binding pocket volumes calculated by fpocket

Enzyme	Pocket Volume (A3)
Wild Type	317
L20A	416
F76A	447
L20A/F76A	529

7.2 Covalent Docking

Linear aldol products, with the MOE functional group at the 2' position (product **12**), were covalently docked into the active-site of the 3 mutants using AutoDock[4] via the program YASARA. For each of these docking runs, 50 poses were generated and the best poses were selected by comparison to the binding mode of the WT, and by binding energy. The three best docked poses and the WT crystal structure are shown in the main text and also below:

WT (pdb = 1JCL)

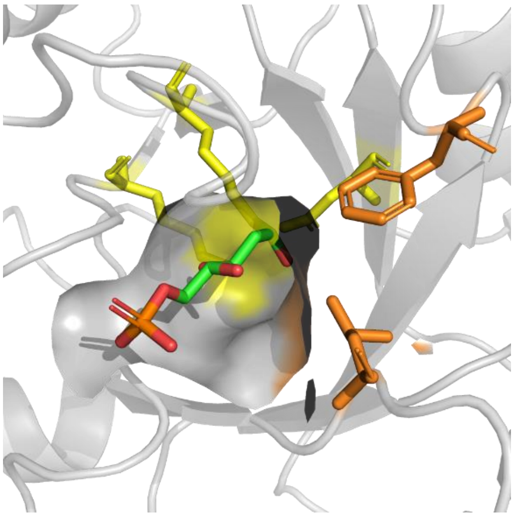

Figure 22: WT crystal structure 1JCL from[5]

DERA L20A

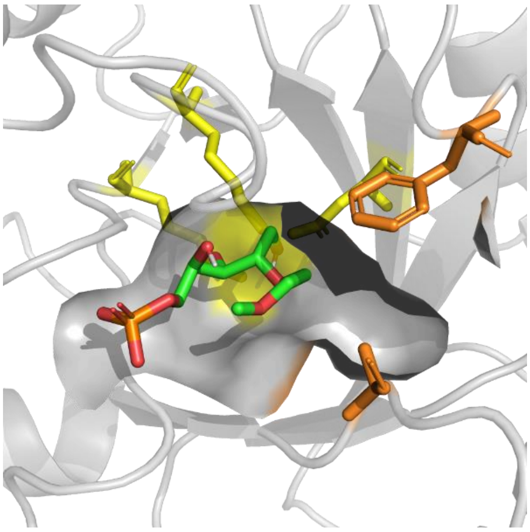

Figure 23: 2'MOE product docked into L20A mutant

DERA F76A

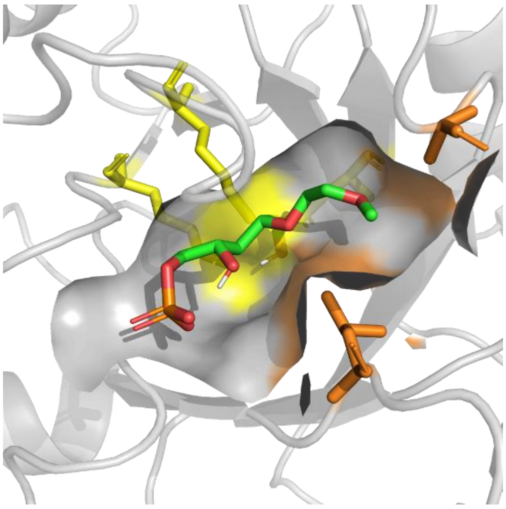

Figure 24: 2' MOE docked into F76A mutant

DERA L20A/F76A

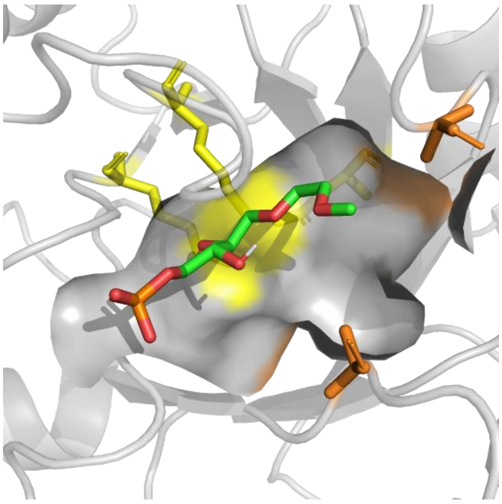

Figure 25: 2'MOE product docked into L20A/F76A mutant

Table 18: binding energies for each of the selected docking poses

Variant	Binding Energy (kcal/mol)
L20A	-9.06
F76A	-8.07
L20A/F76A	-7.11

8. Kinase + Aldolase Cascades

8.1 L-Kinase-Aldolase Cascade

To generate products with defined stereochemistry at the 4-position (L-lyxose-5-phophate analogues), the aldolase reaction was combined with a kinase (*Cs*GK) starting from L-glyceraldehyde (**16**) to generate the L-G3P acceptor (**1a**) *in situ*.

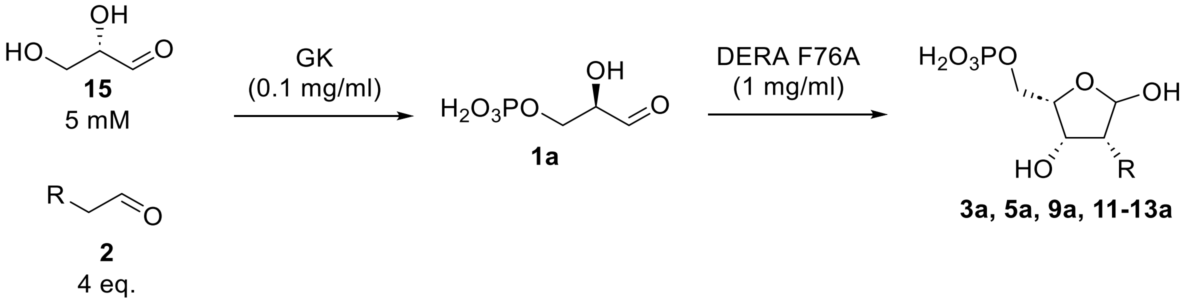

8.1.1 Substrate Scope Screening

To a 96 well plate 10 µL of donor substrate (200 mM, final reaction concentration 20 mM) was added. For substrates insoluble in water donor solutions were made up in DMSO giving a final concentration of 10%. A reaction mixture was made up containing L-glyceraldehyde (6.25 mM, final reaction concentration 5 mM), ATP (9.4 mM, final reaction concentration 7.5 mM, 1.5 eq.), $MgCl_2$ (9.4 mM, final reaction concentration 7.5 mM, 1.5 eq.), HEPES buffer pH 7.5 (125 mM, final reaction concentration 100 mM), aldolase (1.25 mg/ml, final reaction concentration 1 mg/ml). 80 µL of the reaction mixture was added to the plates and the reactions were initiated with 10 µL of glycerokinase (1 mg/ml, final reaction concentration 0.1 mg/ml). The plates were sealed and incubated at 30 °C shaking at 900 rpm. After four hours the reactions were quenched and derivatised with 100 µL of *O*-benzylhydroxylamine (100 mM) in methanol for one hour. Reactions were then filtered through 96 well 0.45 A filter plates to remove precipitated protein and analysed by UPLC-UV at 220 nm. Product masses were confirmed by UPLC-MS giving identical mass spectra to the products from the initial screen.

8.1.2 Example HPLC Trace

Representative HPLC Traces for DERA F76A reaction, with the methoxyacetaldehyde donor (generating product **11a**) have been included alongside, Aldolase free, kinase free and enzyme-free control reactions.

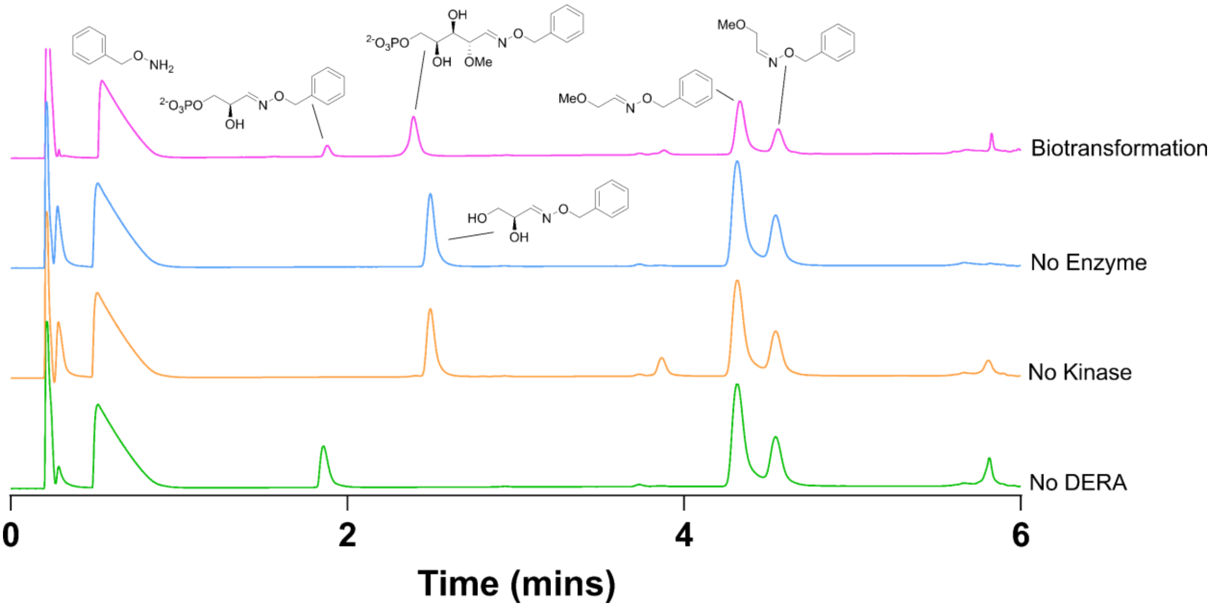

Figure 26: Example HPLC traces for methoxy biotransformation from GK, DERA cascade

Table 19: RP-HPLC retention times for products of GK, DERA cascade

Underivatised Structure	Derivatised structure	Compound Number	Retention Time
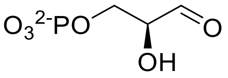	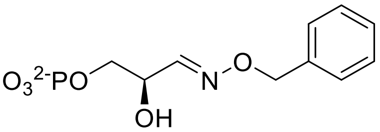	**1a**	1.84
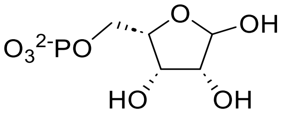	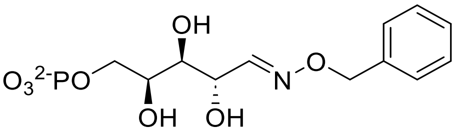	**3a**	1.48
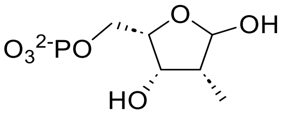	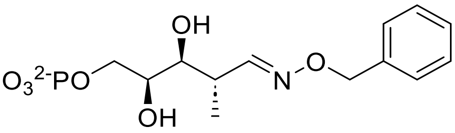	**5a**	2.38
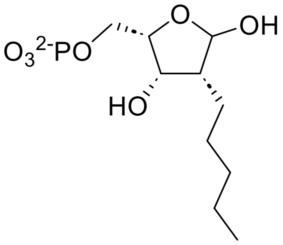	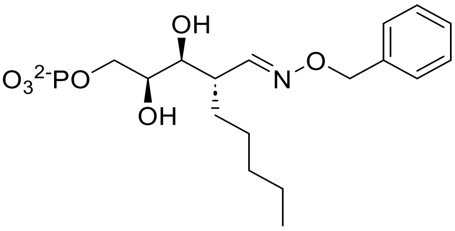	**9a**	4.22
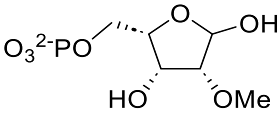	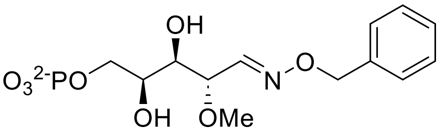	**11a**	2.40
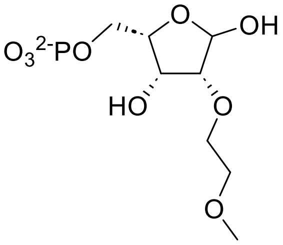	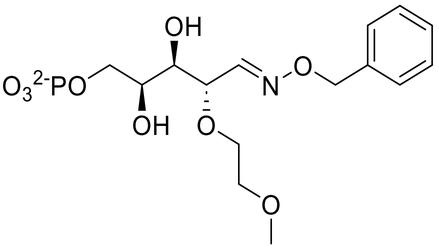	**12a**	2.70
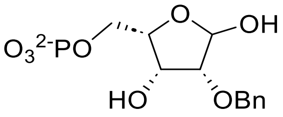	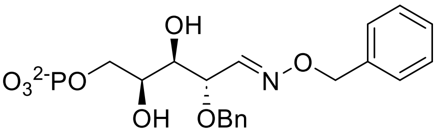	**13a**	4.00

8.1.3 Tables of Results

Table 20: peak areas for E. coli WT DERA

Donor Substrate	Peak Area	Product Formation
Glycolaldehyde	2621 ± 5	50 ± 0.1
Propanal	4256 ± 17	81 ± 0.4
Heptanal	0	0
Methoxyacetaldehyde	0	0
Methoxy(ethoxy) acetaldehyde	0	0
O-Benzyl acetaldehyde	0	0

Table 21: peak areas for F76A DERA

Donor Substrate	Peak Area	Product Formation
Glycolaldehyde	3790 ± 27	72 ± 0.6
Propanal	3878 ± 100	74 ± 2.3
Heptanal	3198 ± 51	61 ± 1.2
Methoxyacetaldehyde	3599 ± 33	68 ± 0.8
Methoxy(ethoxy) acetaldehyde	1962 ± 74	37 ± 1.7
O-Benzyl acetaldehyde	5037 ± 56	61 ± 0.8

Table 22: peak areas for L20A/F76A DERA

Donor Substrate	Peak Area	Product Formation
Glycolaldehyde	571 ± 16	11 ± 0.4
Propanal	3917 ± 119	75 ± 2.8
Heptanal	3612 ± 153	69 ± 3.6
Methoxyacetaldehyde	2449 ± 86	46 ± 2.0
Methoxy(ethoxy) acetaldehyde	4830 ± 172	91 ± 4.0
O-Benzyl acetaldehyde	6223 ± 47	75 ± 0.7

8.2 D-Kinase-Aldolase Cascade

To generate products with defined stereochemistry at the 4-position (D-ribose-5-phophate analogues), the aldolase reaction was combined with a kinase (*Cf*DHAK) starting from D-glyceraldehyde (**16**) to generate the D-G3P (**1b**) acceptor *in situ*.

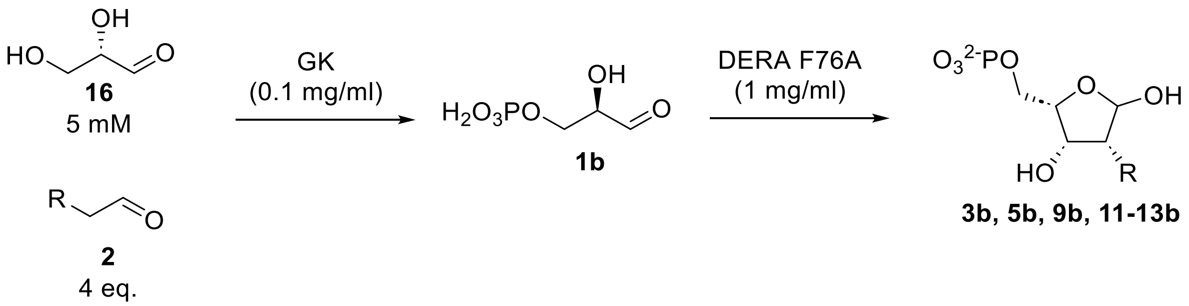

8.2.1 Optimising Enzyme Concentrations

As the amount of starting material isomerised to DHAP by triose phosphate isomerase will depend strongly on the amount of enzyme in solution, alongside the rates of the two reactions, the concentrations of DHAK and DERA were varied with a model substrate (heptanal) to determine optimal conditions for the initial assay. All other conditions were kept the same as previous experiments. Interestingly, lowering the concentration of both the kinase and the aldolase gave slightly increased product formations. This is likely due to reducing the amounts of highly active contaminant TIM still present after purification, reducing the isomerisation of D-G3P into DHAP. For the actual substrate screening 0.05 mg/ml of kinase was used instead of 0.01 mg/ml to ensure full consumption of the D-glyceraldehyde starting material.

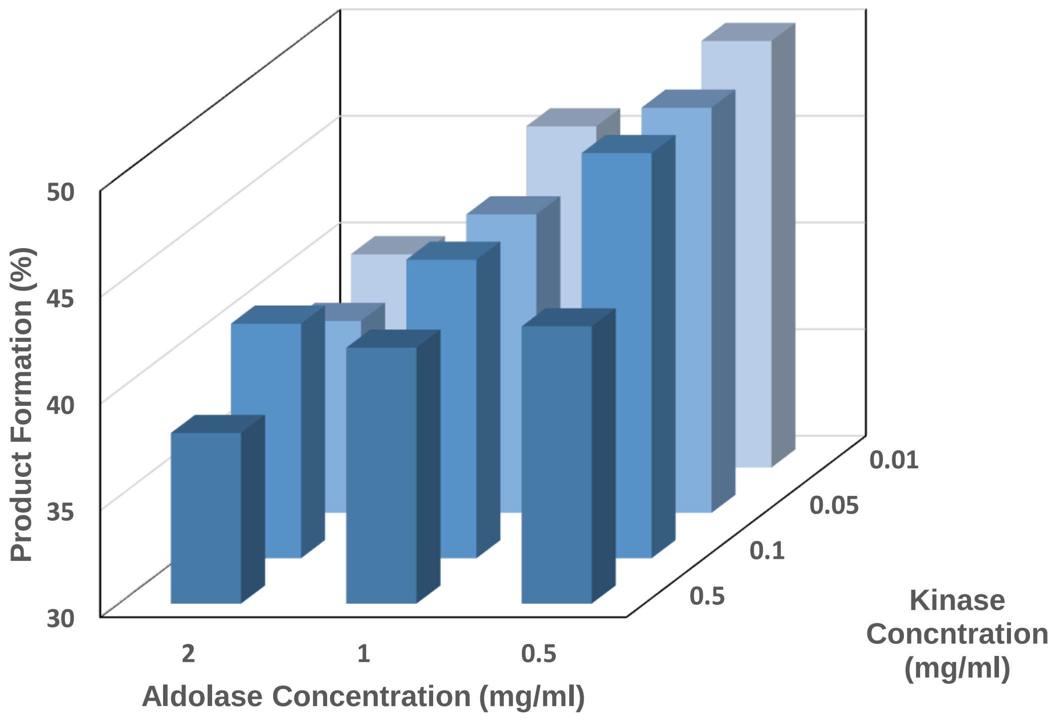

Figure 27: Enzyme loading optimisation for DHAK, DERA cascade

8.2.2 Substrate Scope Screening

To a 96 well plate 10 µL of donor substrate (200 mM, final reaction concentration 20 mM.) was added. For substrates insoluble in water donor solutions were made up in DMSO giving a final reaction concentration of 10%. A reaction mixture was made up containing D-glyceraldehyde (6.25 mM, final reaction concentration 5 mM), ATP (9.4 mM, final reaction concentration 7.5 mM, 1.5 eq.), $MgCl_2$ (9.4 mM, final reaction concentration 7.5 mM, 1.5 eq.), HEPES buffer pH 7.5 (125 mM, final reaction concentration 100 mM), aldolase (0.63 mg/ml, final reaction concentration 0.5 mg/ml). 80 µL of the reaction mixture was added to the plates and the reactions were initiated with 10 µL of dihydroxyacetone kinase (0.5 mg/ml, final reaction concentration 0.05 mg/ml). The plates were sealed and incubated at 30 °C shaking at 900 rpm. After four hours the reactions were quenched with 100 µL of *O*-benzylhydroxylamine (100 mM) in methanol for one hour. Reactions were then filtered through 96 well 0.45 Å filter plates to remove precipitated protein and analysed by UPLC-UV at 220 nm. Product masses were confirmed by UPLC-MS using the DERA F76A mutant, peaks at the same retention time in other mutants were assumed to be the same product.

8.2.3 Example HPLC Traces

As before, example HPLC traces have been included for the methoxyacetaldehyde donor, generating product **11b**. A biotransformation with F76A, a control without aldolase, a control without kinase and a completely enzyme-free control have been included. All substrates were analysed using HPLC Method 1. Products were confirmed by LC-MS analysis and gave identical mass spectra to those previously included.

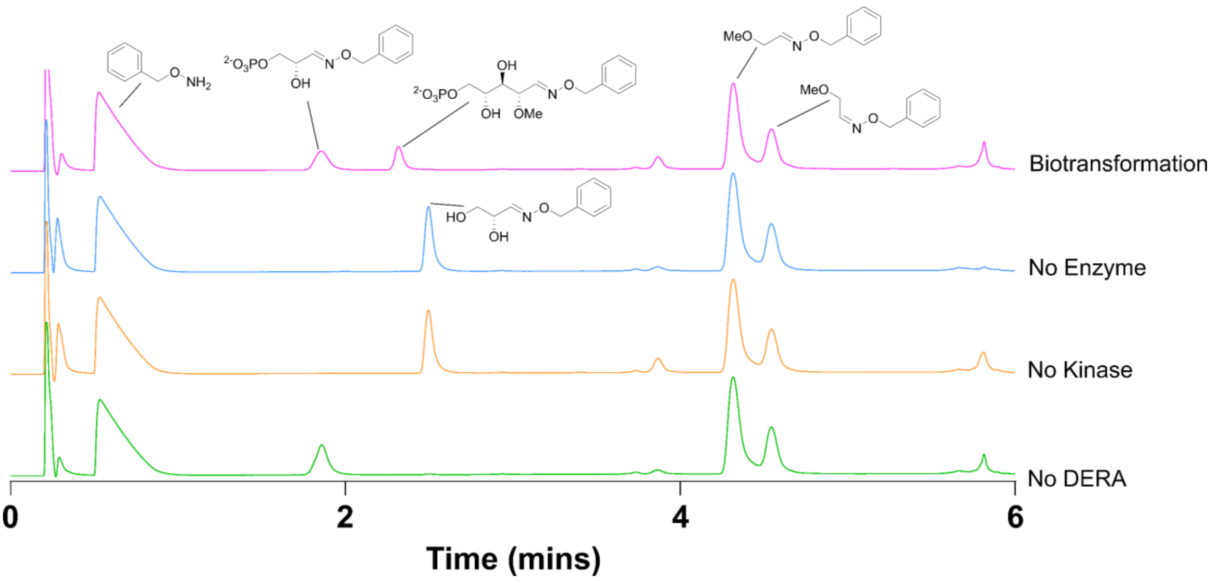

Figure 28: Example HPLC traces for methoxy product from DHAK, DERA cascade

Table 23: RP-HPLC retention times for products of DHAK, DERA cascade

Underivatised Structure	Derivatised structure	Compound Number	Retention Time
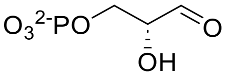	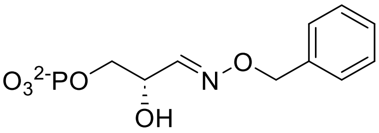	**1b**	1.84
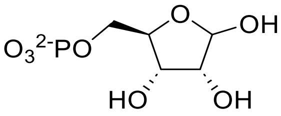	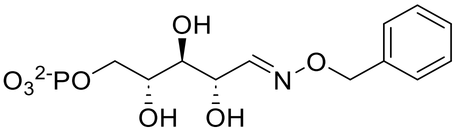	**3b**	1.52
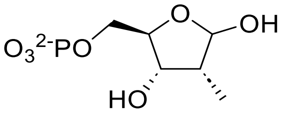	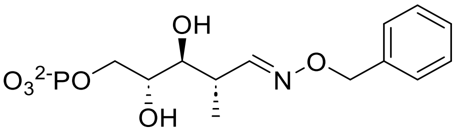	**5b**	2.46
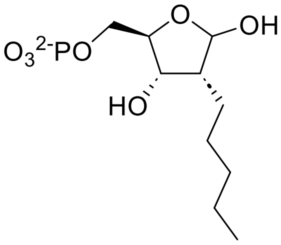	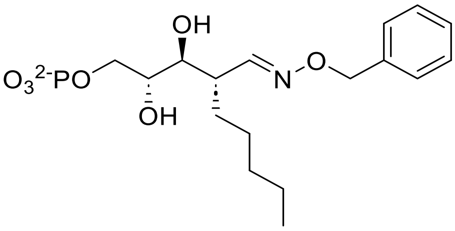	**9b**	4.32
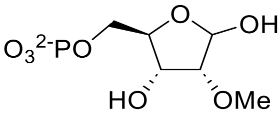	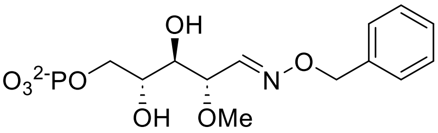	**11b**	2.34
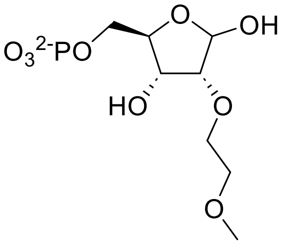	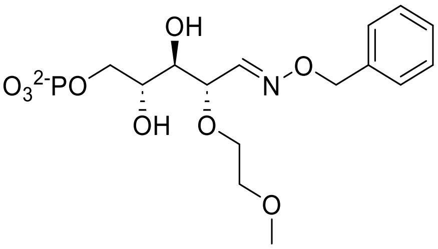	**12b**	2.68
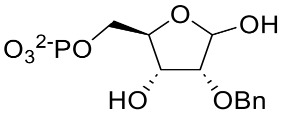	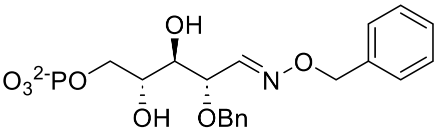	**13b**	3.98

8.2.4 Results Tables

For the enzymes screened tables of peak areas and conversions have been included

Table 24: peak areas for WT DERA

Donor Substrate	Peak Area	Conversion(%)
Glycolaldehyde	1995 ± 27	38 ± 1.7
Propanal	3106 ± 16	58 ± 0.4
Heptanal	0	0
Methoxyacetaldehyde	0	0
Methoxy(ethoxy) acetaldehyde	0	0
O-Benzyl acetaldehyde	0	0

Table 25:peak areas for F76A DERA

Donor Substrate	Peak Area	Conversion (%)
Glycolaldehyde	1544 ± 44	25 ± 1.2
Propanal	3534 ± 20	67 ± 0.5
Heptanal	2101 ± 218	40 ± 5
Methoxyacetaldehyde	1198 ± 17	23 ± 0.4
Methoxy(ethoxy) acetaldehyde	1014 ± 19	19 ± 0.4
O-Benzyl acetaldehyde	4267 ± 3	40 ± 0.1

Table 26: peak areas for 20A/F76A DERA

Donor Substrate	Peak Area	Conversion(%)
Glycolaldehyde	194 ± 12	2 ± 0.1
Propanal	1856 ± 82	35 ± 2
Heptanal	2473 ± 146	47 ± 4
Methoxyacetaldehyde	169 ± 5	3 ± 0.1
Methoxy(ethoxy) acetaldehyde	1014 ± 19	33 ± 0.9
O-Benzyl acetaldehyde	4268 ± 3	17 ± 2.8

8.2.5 Phosphorylation of Glycolaldehyde Donor

One-pot kinase-aldolase reaction with glycolaldehyde yielded the product in reasonable conversions, however, also yielded glycolaldehyde phosphate as a major impurity. This product was formed by the direct phosphorylation of the glycolaldehyde donor by DHAK.

To prevent the formation of glycolaldehyde phosphate, the reaction was carried out sequentially. Phosphorylation of D-glyceraldehyde (**16**) was carried out for 2 hours, following this, the kinase was removed with a 10k MWCO filter and the reaction mixture was then used directly in the aldolase biotransformation for 3 hours. Carrying out the reaction in this manner completely removed the contaminant peak. When carrying out the reactions sequentially a slight decrease in conversion was observed, likely caused by the additional time for degradation of D-G3P (**1b**). Conversions for the sequential reaction rather than the one-pot reaction were included in the D-G3P (**1b**) substrate screening.

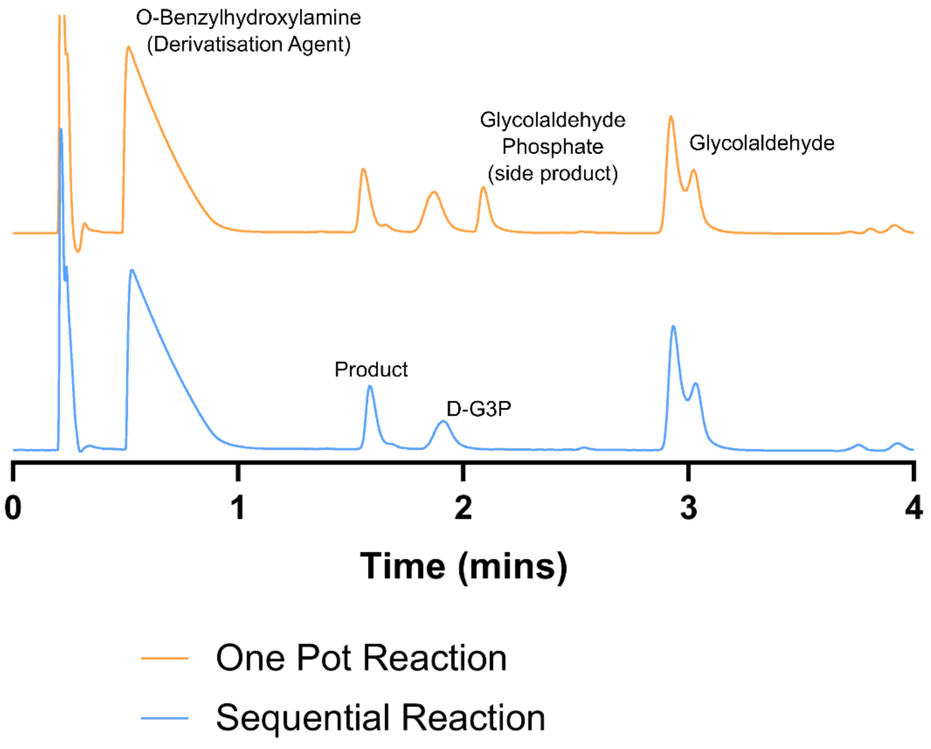

Figure 29: HPLC traces for both sequential and one pot biotransformations

Table 27: peak areas for one-pot vs sequential biotransformations with glycolaldehyde donor

Name	Peak Areas	Conversion
WT - Sequential	2006 ± 73	38 ± 1.4
F76A - Sequential	1354 ± 52	26 ± 1.0
L20A/F76A - Sequential	92 ± 93	38 ± 0.5
WT – One Pot	1995 ± 27	37 ± 0.8
F76A – One Pot	1544 ± 44	29 ± 0.8
L20A/F76A – One Pot	194 ± 12	4 ± 0.2

9. Kinase + Oxidase + Aldolase Cascades

9.1 Oxidation of fluoroethanol

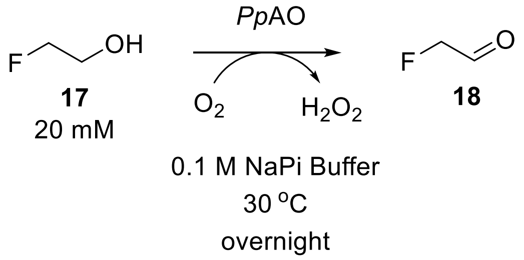

To a 2 ml Eppendorf was added fluoroethanol (30 mM final concentration), phosphate buffer (100 mM, pH 7.5), and milliQ water to give a final volume of 500 µL. The reaction was initiated by the addition of methanol alcohol oxidase from *Pichia pastoris* (0.5 mg/ml final concentration). The reaction was incubated in an orbital incubator at 180 rpm and 30 °C for 18 hours (overnight). Enzyme was removed from the reaction by centrifugation through a 500 µL 10k MWCO vivaspin and then analysed directly by ^{19}F NMR.

Analysis of the ^{19}F NMR shows a conversion of 74% to the aldehyde **18** and 20% of the overoxidation product fluoroacetic acid. While the overoxidation is likely due to the longer reaction times, the product fluoroacetic acid is highly toxic and therefore these reactions should be treated with care.

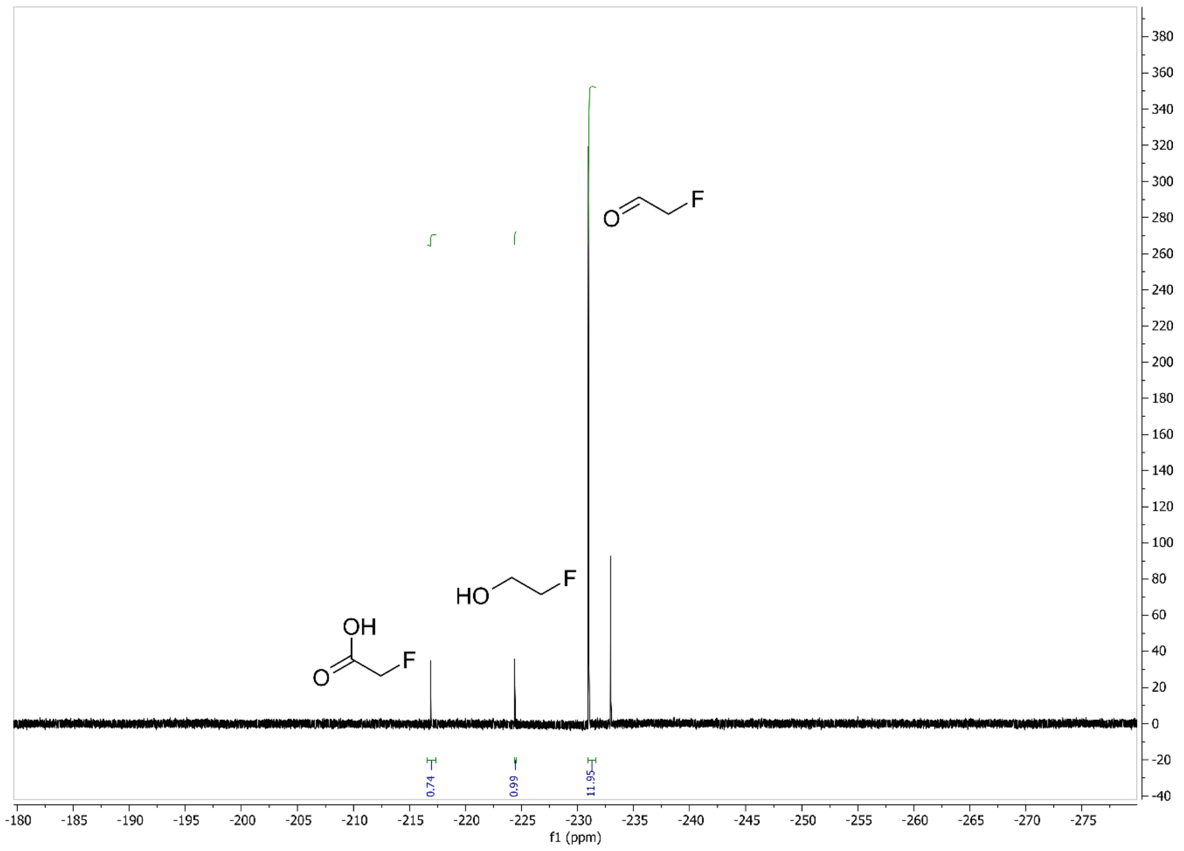

Figure 30: ^{19}F NMR of fluoroethanol oxidation crude reaction mix

9.2 L-Kinase-Oxidase-Aldolase Cascade

9.2.1 Biotransformation Protocol

A 2x reaction mixture was made up containing L-glyceraldehyde (10 mM,), fluoroethanol (40 mM), ATP (15 mM), $MgCl_2$ (15 mM) in HEPES Buffer (0.1 M, pH 7.5). A 2x enzyme mixture was made up containing GK (0.2 mg/ml), DERA (2 mg/ml), and *Pp*AO (1 mg/ml) in HEPES buffer (0.1 M, pH 7.5). The reaction was initiated by addition of 100 µL reaction mix to 100 µL enzyme mix giving final reaction concentrations half those stated. Samples were incubated for 4 hours at 30 °C 750 rpm. After 4 hours samples were derivatised with *O*-benzylhydroxylamine, filtered, and analysed by HPLC.

9.2.2 Example HPLC

An example HPLC trace of a biotransformation alongside an aldolase-free, kinase-free, oxidase free and an enzyme-free control reaction have been included. Unlike for previous HPLC traces, for the 2-F reactions, multiple peaks containing product mass were observed, these are assumed to be diastereomers at C2.

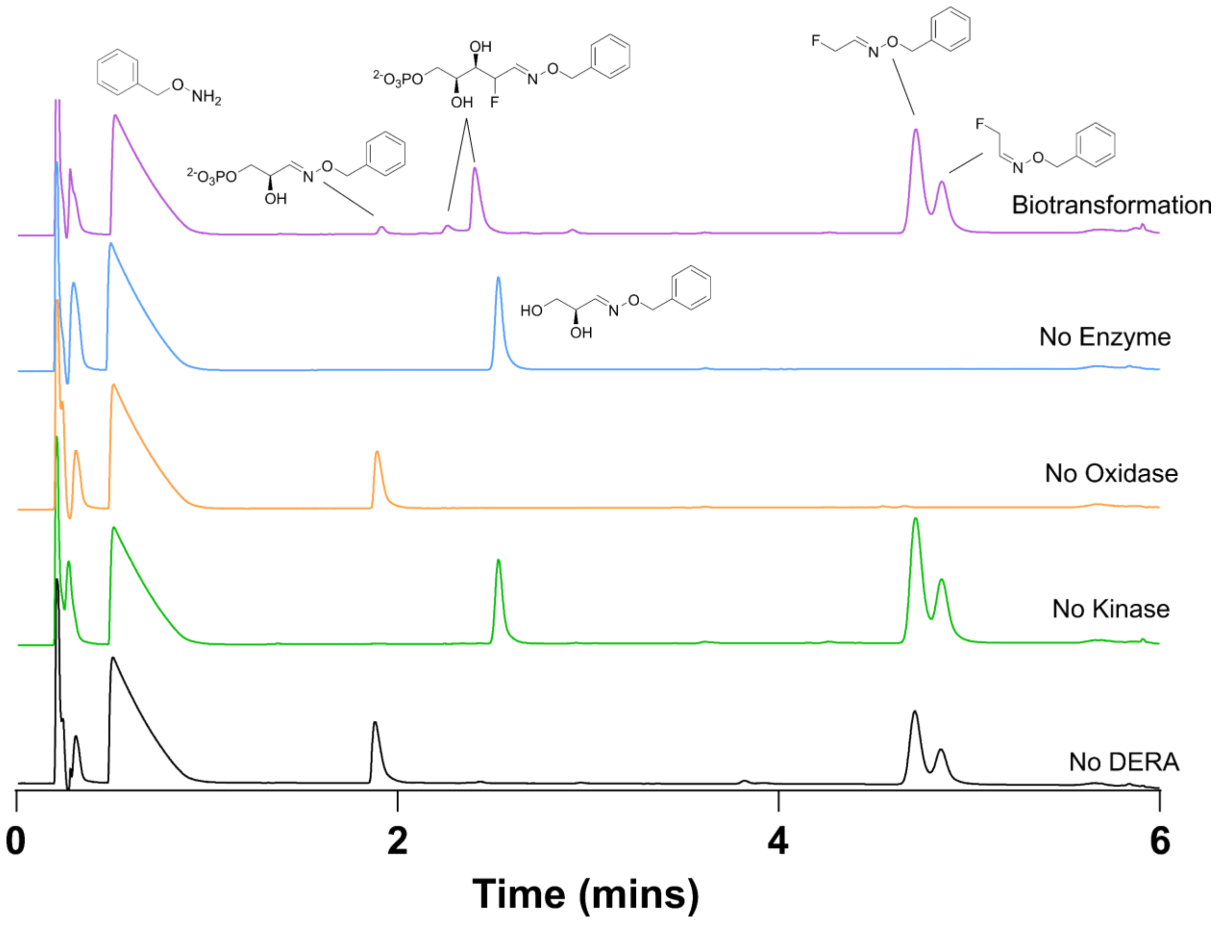

Figure 31: Example HPLC traces for 2'F product from PpAO, GK, DERA cascade

9.2.3 Mass Spectra

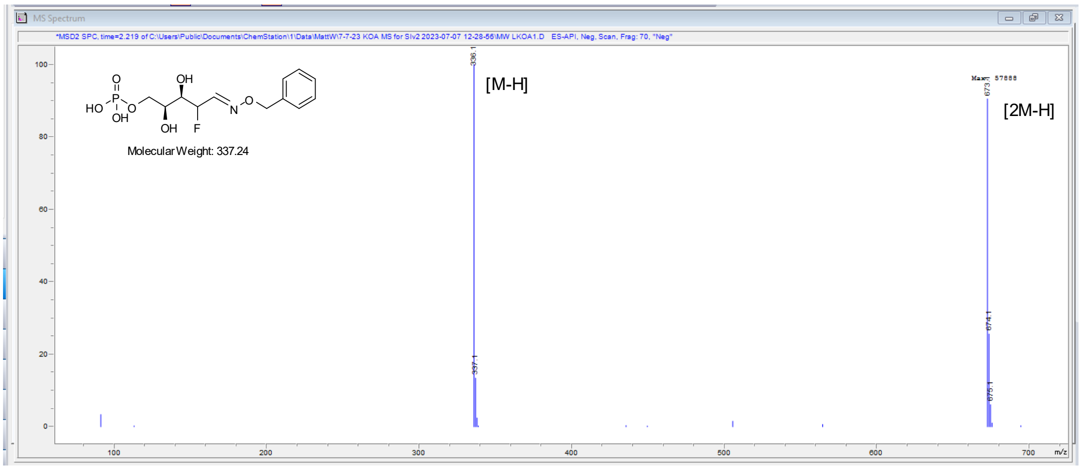

*Figure 32: ESI- mass spectrum for derivatised 2'-F product (**19a**), [M-H] = 336.1*

9.2.4 Results Tables

Table 28: peak areas for L kinase oxidase aldolase cascade

Variant	Peak Area	Conversion
*Ec*DERA$_{WT}$	3955 ± 33	75 ± 0.6
*Ec*DERA$_{F76A}$	2360 ± 27	45 ± 0.5
*Ec*DERA$_{L20A/F76A}$	74 ± 6	1 ± 0.1

9.2.5 Time Course

To determine if the lack of stereochemical control for the fluorinated product was due to thermodynamic effects, as can be seen for threonine aldolases[6], the ratio of the two peaks was measured over the course of the four-hour reaction and then again after 18 hours. For the first four hours no change in the two peaks was observed, and after 18 hours only a small change in ratio from 88:12 to 84:16 was observed.

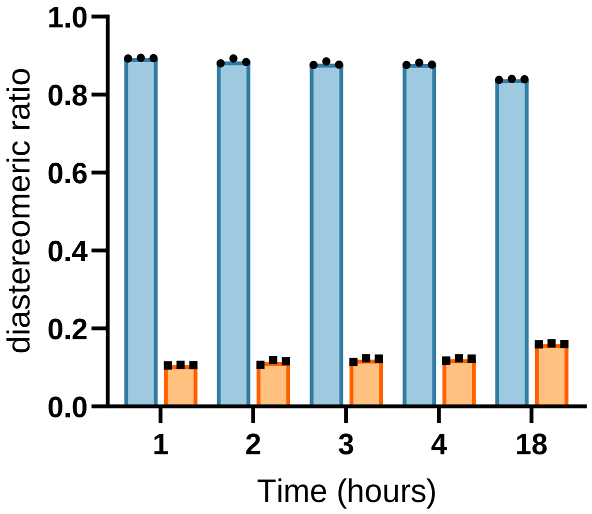

*Figure 33: The ratio of the two diastereomers observed for the fluorinated product (**19a**) over time.* Triplicate repeats for three separate biotransformations are shown over the bars. *A negative control gave no conversion and therefore does not have a diastereomeric ratio, as such it has not been included.*

9.3 D-Kinase-Oxidase-Aldolase Cascade

9.3.1 Biotransformation Protocol

A 2x Reaction mixture was made up containing D-glyceraldehyde (10 mM), fluoroethanol (40 mM), ATP (15 mM), $MgCl_2$ (15 mM) in HEPES Buffer (0.1 M pH 7.5). A 2x enzyme mixture

was made up containing DHAK (0.2 mg/ml), DERA (2 mg/ml), and PpAO (1 mg/ml) in HEPES buffer (0.1 M). The reaction was initiated by addition of 100 µL Reaction Mix to 100 µL enzyme mix giving final reaction concentrations half those stated. Samples were incubated for 4 hours at 30 °C 750 rpm. After 4 hours samples were derivatised with *O*-benzylhydroxylamine, filtered and analysed by HPLC.

9.3.2 Example HPLC

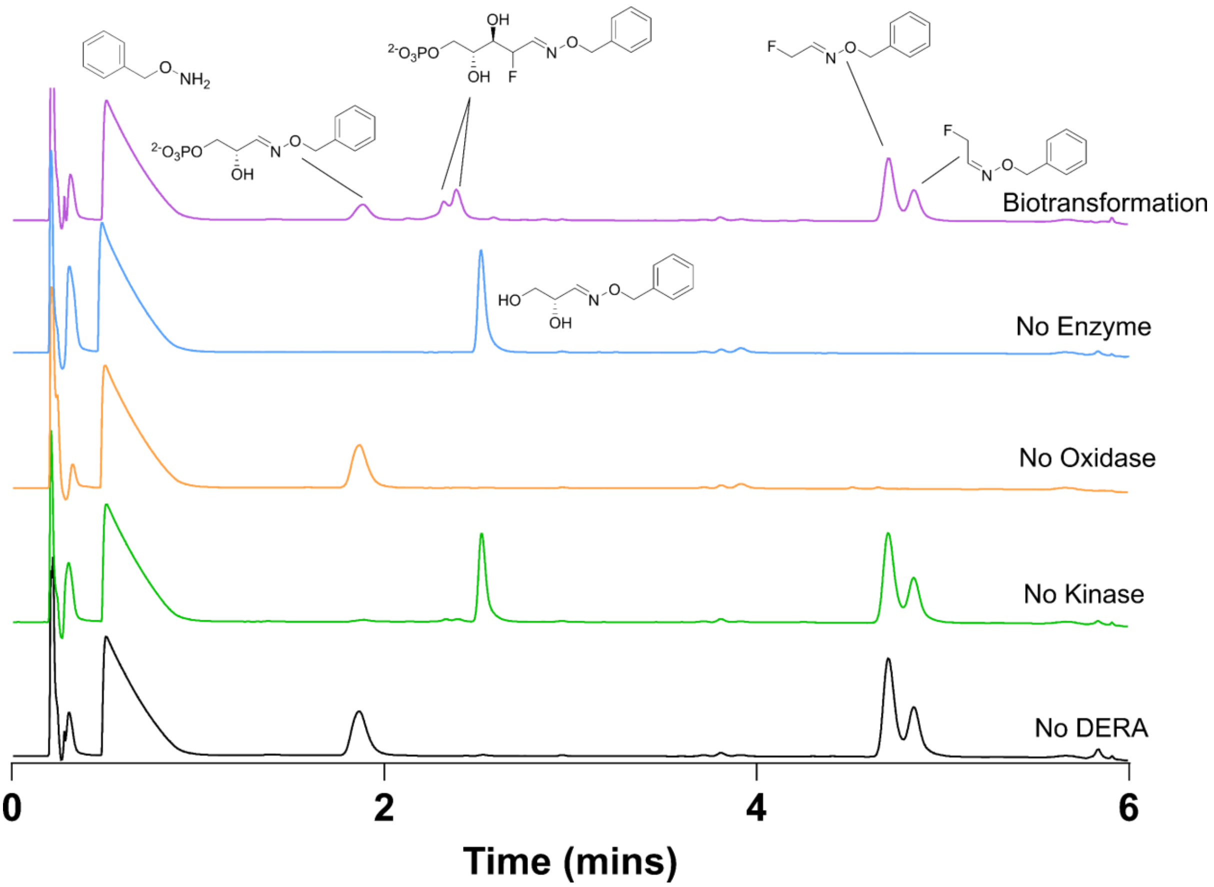

Figure 34: Example HPLC traces for 2-F product from PpAO, DHAK, DERA cascade

9.3.3 Mass Spectra

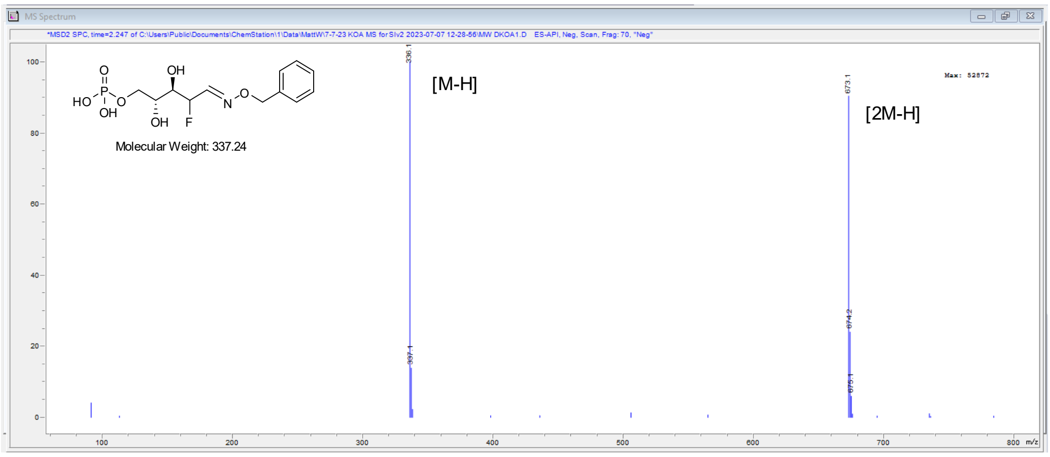

Figure 35: ESI- mass spectrum of derivatised 2-F product (19b), [M-H] = 336.1

9.3.4 Results Tables

Table 29: peak areas for D kinase oxidase aldolase cascade

Variant	Peak Area	Conversion
*Ec*DERA$_{WT}$	2602 ± 294	49 ± 5.6
*Ec*DERA$_{F76A}$	1012 ± 12	19 ± 0.2
*Ec*DERA$_{L20A/F76A}$	19 ± 1.6	<0.5%

10. PEP/PK Recycling System

10.1 PEP inhibition of TPI

Initial experiments were carried out adding PEP and PK to the biotransformations, using heptanal as a donor substrate. This showed that addition of 10 mM PEP alongside the PK recycling system increased conversion from 50% to 65%, while decreasing the amount of DHAP in the reaction. As 10 mM PEP allowed the reaction to reach and even slightly exceed the conversion for the L-cascade, the remainder of substrates were screened using these conditions.

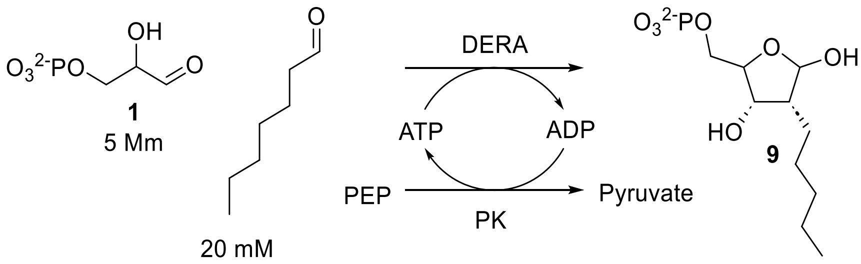

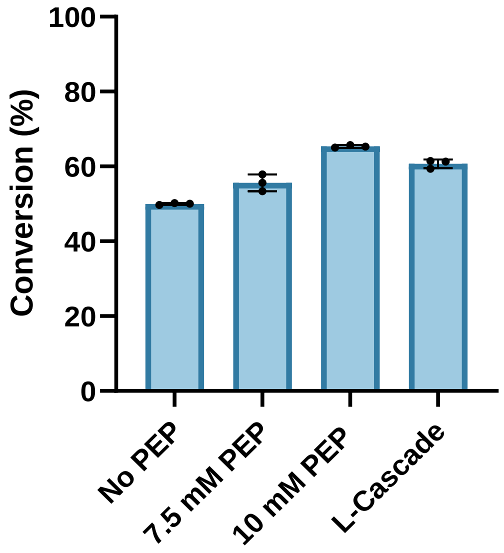

Figure 36: Mean conversion of triplicate biotransformations to product at different PEP concentrations, L-cascade is also included as a control for conversion in the absence of TIM activity. Data points for individual reactions are shown, error bars show standard deviation of triplicate repeats.

Table 30: peak areas for addition of PEP/PK recycling

Name	Area	Conversion
No PEP	2643 ± 10	50 ± 0.2
7.5 mM PEP	2931 ± 96	56 ± 1.8
10 mM PEP	3444 ± 16	66 ± 0.3

10.2 Substrate Screening

A 2x reaction mixture was made up containing D-glyceraldehyde (10 mM), aldehyde donor (40 mM), ATP (10 mol %), $MgCl_2$ (10 mM), PEP (20 mM), in HEPES buffer (0.1 M pH 7.5). A 2x enzyme mixture was made up containing DHAK (0.2 mg/ml), $DERA_{F76A}$ (2 mg/ml), and pyruvate kinase (20 U/ml) in HEPES buffer (0.1 M, pH 7.5). Reaction was initiated by addition of 100 µL reaction Mix to 100 µL enzyme mixture giving final reaction concentrations half those stated. Samples were incubated for 4 hours at 30 °C 750 rpm. After 4 hours samples were derivatised with *O*-benzylhydroxylamine, filtered and analysed by HPLC.

For reactions without PEP/PK 1.5 eq. of ATP was added and no PEP or PK was added.

For 2-F reactions the enzyme mixture also contained 0.5 mg/ml final concentration of *Pp*AO, and $DERA_{WT}$ was used instead of the F76A mutant.

Table 31: peak areas for PEP/PK substrate scope screening

Reaction	Peak Area	Conversion
Propanal (with PEP/PK)	3870 ± 104	73% ± 2.0
Propanal (without PEP/PK)	2954 ± 112	56% ± 2.1
Heptanal (with PEP/PK)	3720 ± 111	71% ± 2.1
Heptanal (without PEP/PK)	3047 ± 28	58% ± 0.5
Methoxyacetaldehyde (with PEP/PK)	1596 ± 54	30% ± 2.1
Methoxyacetaldehyde (without PEP/PK)	551 ± 34	10% ± 0.7
Methoxy(ethoxy) acetaldehyde (with PEP/PK)	2438 ± 57	46% ± 1
Methoxy(ethoxy) acetaldehyde (without PEP/PK)	470 ± 26	9% ± 0.5

Fluoroacetaldehyde (with PEP/PK)	2471 ± 47	47% ± 0.9
Fluoroacetaldehyde (without PEP/PK)	3782 ± 107	72% ± 2.0

11. Semi-Preparative Scale Synthesis of Pentose-5-Phosphates

11.1 DOE

Design of experiments was used to optimise the conditions of the scale up reactions for both the L- and D- cascade scale-up reactions. As a model reaction for the DOE, the methoxyacetaldehyde substrate was chosen, optimised conditions for this substrate were applied to all other substrates for semi-preparative scale reactions. DOE was carried out using JMP Pro 17.

11.1.1 L-Cascade

For the L-kinase-aldolase cascade, to determine optimal reaction parameters design of experiment (DOE) was carried out to fit a response surface considering aldolase concentration, kinase concentration and donor concentration as factors. Both the donor dimerization side product and 2-methoxy-L-lyxose-5-phosphate product **12a** formation were used as responses.

Table 32: L-kinase-aldolase DOE reactions

Donor Concentration (mM)	Aldolase Concentration (mg/ml)	Kinase Concentration (mg/ml)	Product Peak Area	Donor Dimer Peak Area
40	1	0.05	1169	207
40	1	0.5	1030	161
40	3	0.275	2868	361
40	3	0.275	2812	342
40	5	0.05	4000	818
40	5	0.5	3942	668
120	1	0.275	1770	672
120	1	0.275	1529	587
120	3	0.05	4046	2515
120	3	0.275	3948	2879
120	3	0.275	4066	3013
120	3	0.275	4250	3073
120	3	0.5	4260	2858
120	3	0.5	4056	2913
120	5	0.275	4270	6165
200	1	0.05	1059	906
200	1	0.5	1285	1002
200	3	0.275	3904	4573
200	5	0.05	4847	9517
200	5	0.5	4822	12668

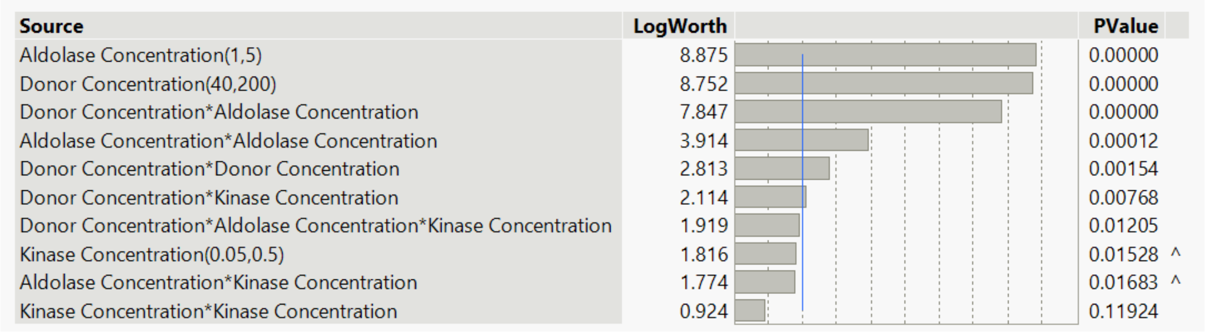

Figure 37: DOE model factors and their significance. P values were calculated using two-sided, sequential T tests, no adjustments were made for multiple comparisons.

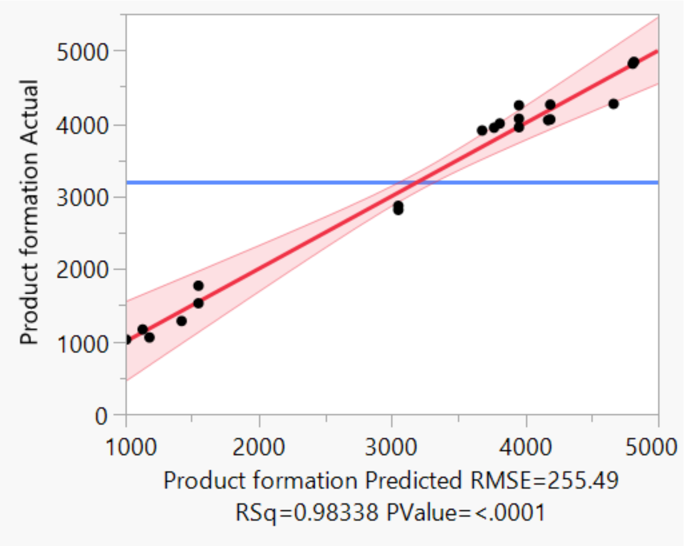

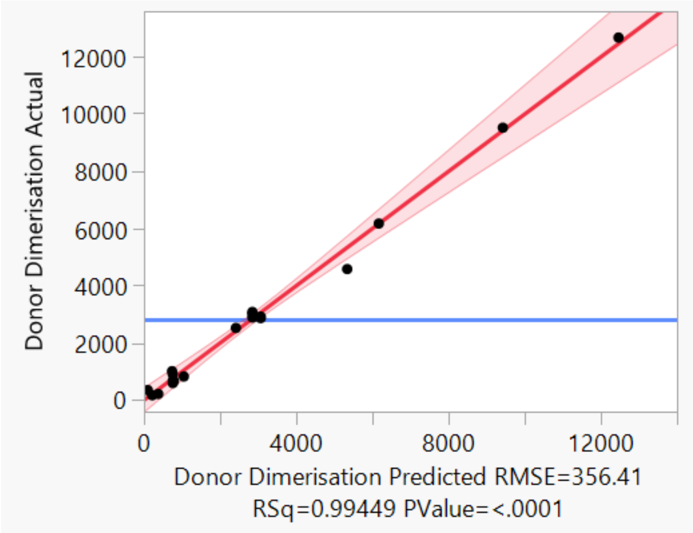

Figure 38: Actual vs Predicted peak areas for product formation and donor dimerization. Upper and lower bands represent 95% confidence intervals. P value was calculated using a one-sided F test. No adjustments were made for multiple comparisons

Both donor concentration and aldolase concentration seemed to have a significant effect on product formation and donor dimerization. In contrast, the kinase concentration does not appear to affect ether property within the concentrations screened, suggesting the kinase reaction is significantly faster than the aldolase step. Both donor concentration and aldolase concentration appear to increase product formation up to a point before levelling off. However, both aldolase and donor concentration, unsurprisingly, increased the amount of donor dimerization in addition to product formation. To determine the optimal conditions for the reaction product equal weighting was given to both maximising product formation and

minimising donor dimerization. However, as the products for this reaction were subsequently purified by anion exchange chromatography an alternative would be to merely consider product formation as the donor dimerization side product is subsequently removed by purification. While further rounds of DOE optimisation may help to better characterise the system, the optimal conditions from one round gave what the authors deemed to be reasonable conversions. These conditions were therefore taken an applied to the semi-preparative synthesis of L-Lyxose-5-phosphate analogues **11a, 12,** and **13a**.

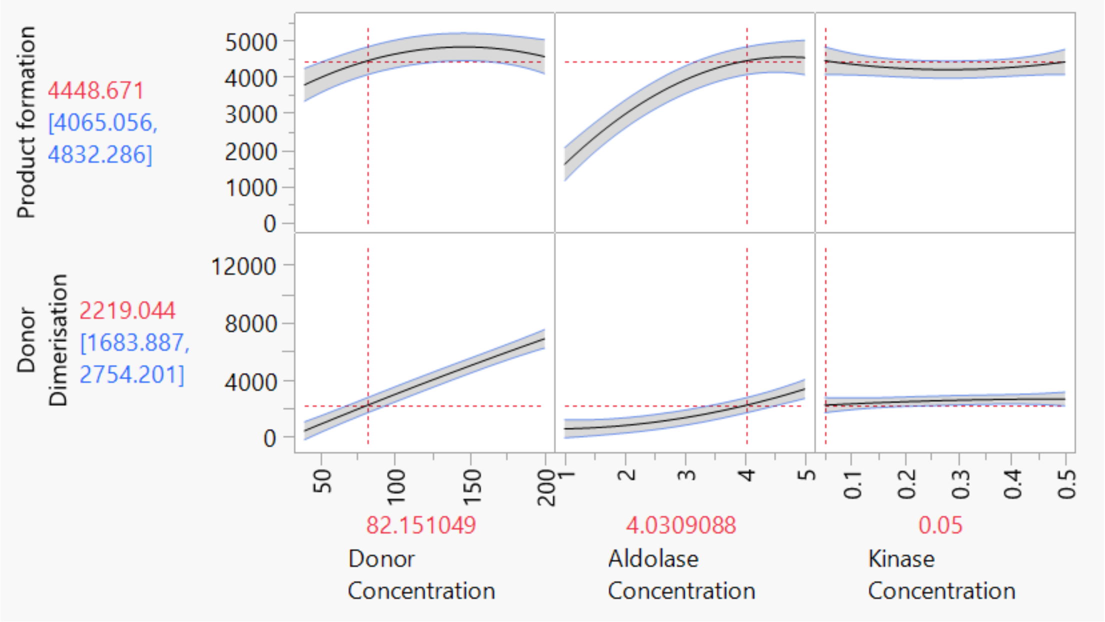

Figure 39: DOE Model effects of individual factors, upper and lower bounds represent 95% confidence intervals.

11.1.2 D-Cascade

Addition of the ATP recycling system to the D-Kinase-Aldolase cascade gave many more factors to consider for optimisation. The factors chosen to screen were: aldolase concentration, kinase concentration, pyruvate kinase concentration, donor concentration, PEP concentration, magnesium concentration, ATP loading (mol %), buffer pH and temperature. As the number of experiments required to characterise a full response surface quickly becomes unwieldy, instead a model was built using definitive screening design. Using 25 experimental runs a model was fitted using stepwise regression focused on the minimisation of AICc. For this model only product formation of 2-methoxy-D-ribose-5-phosphate (**11b**) was

considered due to overlap of the pyruvate biproduct peak from the ATP recycling and the donor dimer.

Table 33: D-Kinase-Aldolase DOE reactions

	DERA (mg/ml)	Buffer pH	Temp (°C)	Donor Concentration (mM)	PEP Concentration (mM)	ATP (mol%)	Buffer Concentration (mM)	Pyruvate Kinase Activity (U/ml)	DHAK Concentration (mg/ml)	Magnesium Concentration (mg/ml)	Product Peak Area
1	0.5	6.8	20	20	100	10	150	1	0.5	12.5	94
2	0.5	6.8	20	200	100	10	50	20	0.3	5	439
3	0.5	6.8	37	20	16	1	150	20	0.5	5	66
4	0.5	6.8	37	110	16	10	50	1	0.1	20	99
5	0.5	6.8	37	200	100	1	150	10.5	0.1	20	84
6	0.5	7.5	20	200	16	1	50	20	0.5	20	406
7	0.5	8.2	20	20	16	10	150	20	0.1	20	258
8	0.5	8.2	20	200	58	1	150	1	0.1	5	199
9	0.5	8.2	28.5	20	100	1	50	1	0.5	20	60
10	0.5	8.2	37	20	100	5.5	50	20	0.1	5	23
11	0.5	8.2	37	200	16	10	100	1	0.5	5	173
12	1.75	6.8	20	20	16	1	50	1	0.1	5	514
13	1.75	7.5	28.5	110	58	5.5	100	10.5	0.3	12.5	2319
14	1.75	8.2	37	200	100	10	150	20	0.5	20	915
15	3	6.8	20	20	100	1	100	20	0.1	20	124
16	3	6.8	20	200	16	5.5	150	1	0.5	20	1942
17	3	6.8	28.5	200	16	10	150	20	0.1	5	2075
18	3	6.8	37	20	58	10	50	20	0.5	20	746
19	3	6.8	37	200	100	1	50	1	0.5	5	492
20	3	7.5	37	20	100	10	150	1	0.1	5	340
21	3	8.2	20	20	16	10	50	10.5	0.5	5	1059
22	3	8.2	20	110	100	1	150	20	0.5	5	1712
23	3	8.2	20	200	100	10	50	1	0.1	20	2558
24	3	8.2	37	20	16	1	150	1	0.3	20	447
25	3	8.2	37	200	16	1	50	20	0.1	12.5	949
V1	3	8.2	30	200	58	6.7	50	10.5	0.1	20	3401
V2	3	8.2	30	200	16	6.7	50	10.5	0.1	20	2646
V3	3	8.2	30	200	58	1	50	10.5	0.1	20	3313
V4	3	8.2	30	110	58	10	50	10.5	0.1	20	2885

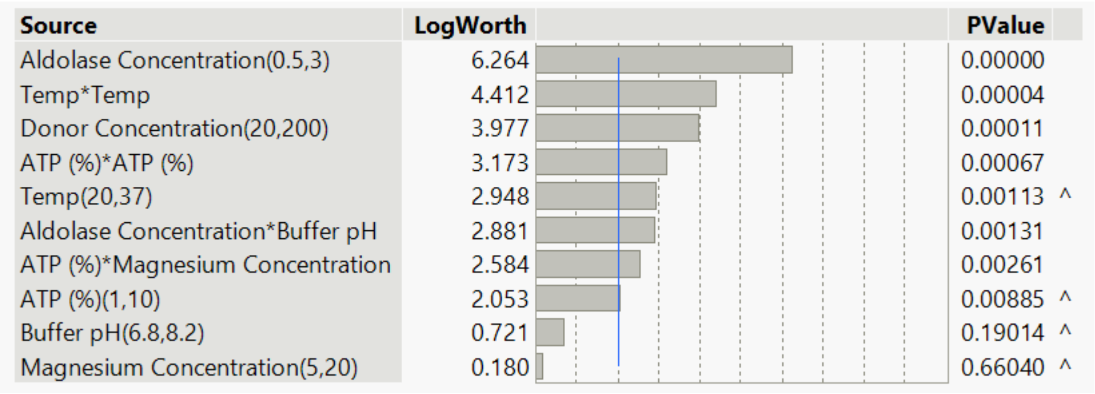

Figure 40: DOE Model Factors and their significance. P values were calculated using two-sided, sequential T tests, no adjustments were made for multiple comparisons.

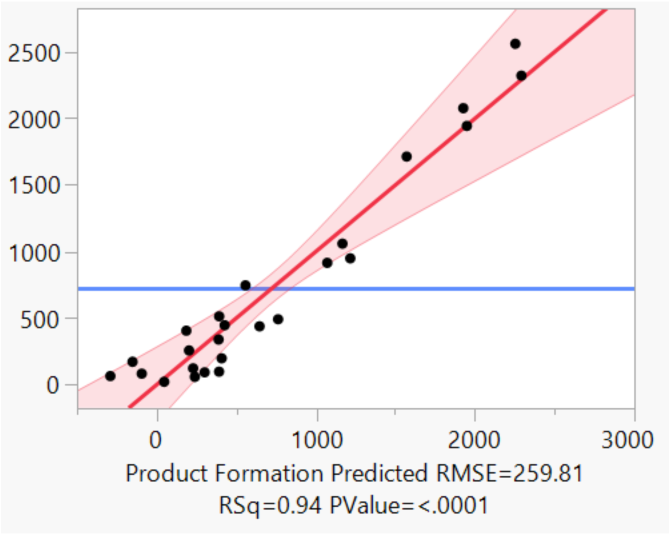

Figure 41: Predicted product formation vs actual product formation, upper and lower bands represent 95% confidence intervals. P value was calculated using a one-sided F test. Adjustments were not made for multiple comparisons.

As before, an increase in both aldolase concentration and donor concentration gave an increased amount of product formed. Increase in buffer pH up to 8.2 also gave an increase in the amount of product formation, a pH of 8.2 was chosen as the maximum due to the pH range of HEPES buffer. Temperature increased conversion up to a maximum of around 27 °C. ATP mol% also appeared to increase the product conversion up to around 6 mol% before levelling off.

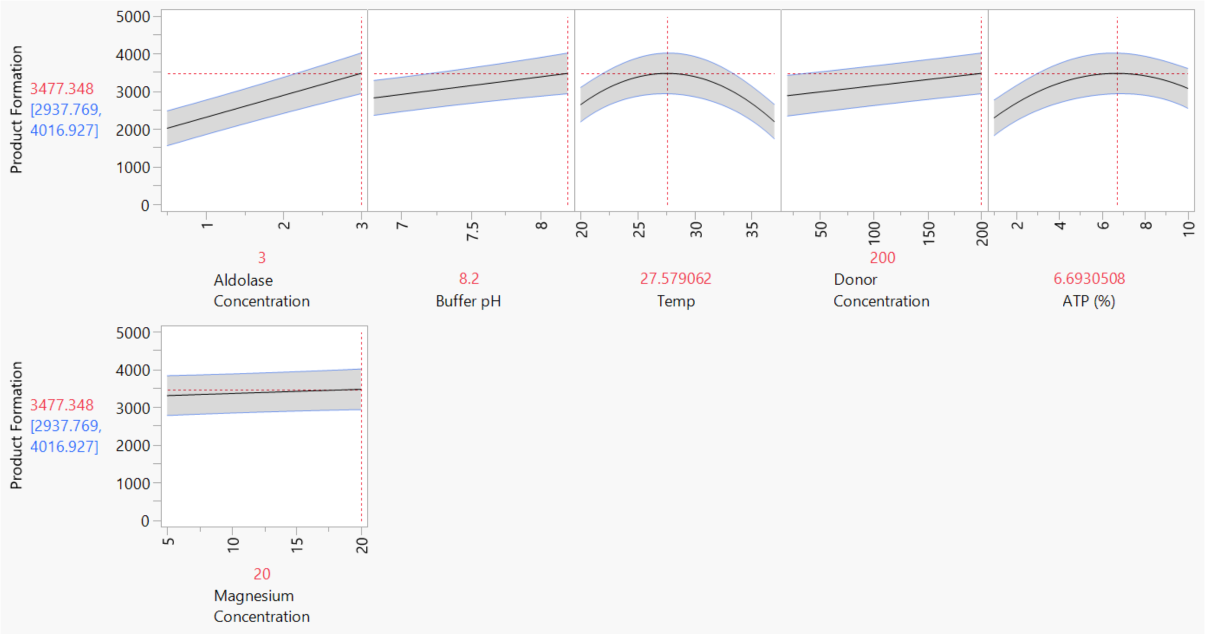

Figure 42: DOE model effects of individual factors, upper and lower bands represent 95% confidence intervals.

To validate the model four additional experiments were carried out based on the maximum desirability.

1) A validation experiment based on the conditions which maximised product formation.
2) As PEP had been shown to inhibit the TIM reaction however the model did not appear to suggest this factor was significant, one validation experiment using minimal PEP was compared to the optimal conditions.
3) The model suggested that ATP mol% had a significant effect on the reaction. This was surprising considering ATP is being recycled by PK and therefore two ATP mol% loadings were chosen.
4) Finally, one validation testing the middle vs the highest amount of donor concentration was considered.

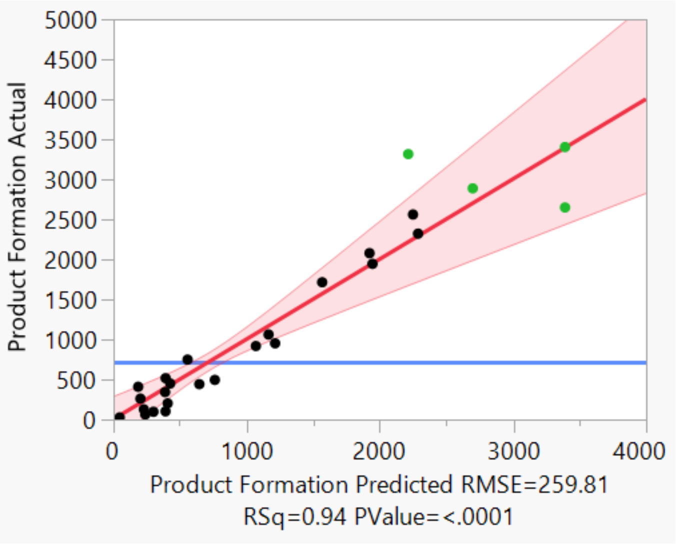

Figure 43: predicted vs actual product formation, green points represent validation experiments, upper and lower bounds represent the 95% confidence intervals. P value was calculated using a one-sided F test. Adjustments were not made for multiple comparisons

Three of the validations (optimal conditions (1), PEP (2), donor concentration (4)) all extrapolated well, giving reasonable results that fit within the bounds of the model.

The validation experiment considering ATP mol% (3) falls outside the bounds of the model, giving a higher conversion than the model predicts. This appears to suggest that ATP mol% is not a significant factor, despite the model's predictions. This is unsurprising as ATP is being recycled in the reaction by PK. As before, while further rounds of DOE might generate a better fitting model, the optimal conditions from this round were deemed enough to achieve a reasonable conversion, therefore, these conditions were taken and used for the semi-preparative synthesis of the D-Ribose-5-Phosphate Analogues **11b** and **12b**.

11.2 Semi-Preparative Scale Syntheses of L-Lyxose-5-Phosphate Analogues

11.2.1 General Procedure for the Synthesis of L-Lyxose-5-Phosphate Analogues

To a 15 ml falcon tube, L-glyceraldehyde (20 mM), aldehyde donor (80 mM), ATP (30 mM), and $MgCl_2$ (30 mM) were added. HEPES buffer (pH 8.2), and water were added so as to give a final buffer concentration of 50 mM and a final reaction volume of 5 ml. The reaction was initiated by addition of $DERA_{F76A}$ (4 mg/ml final concentration), and glycerokinase (0.1 mg/ml

final concentration). The reaction mixture was left shaking in an orbital incubator at 30 °C 200 RPM for 18 hours. After this time, the enzyme was removed using a 10k MWCO filter. The phosphorylated products were then purified using anion exchange chromatography as described below:

The reaction mixtures were loaded directly onto a 5 ml Biorad highQ anion exchange column. The column was washed with 20 ml of water followed by elution with 10 ml of 200 mM ammonium bicarbonate and 10 ml of 400 mM ammonium bicarbonate. 1 ml fractions were collected and analysed for product presence by mass spectrometry (ESI-). Fractions containing product mass by ESI- were pooled and freeze dried to give the diammonium salts of the sugar phosphates products as a white solid.

11.2.2 2-deoxy-2-methoxy-L-lyxose-5-phosphate (**11a**)

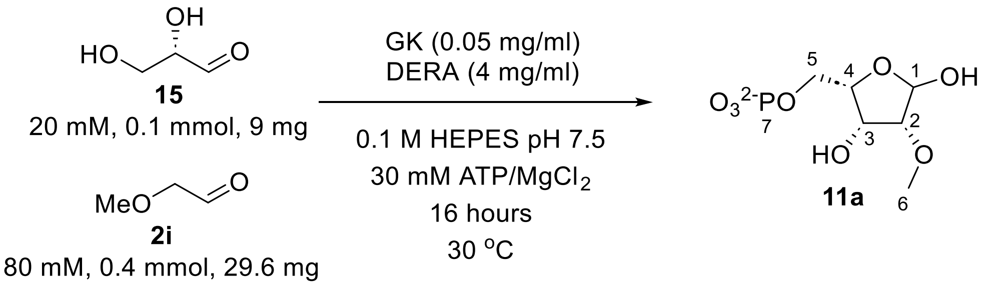

Title compound **11a** was prepared accord to the general procedure using L-glyceraldehyde **15** (20 mM, 0.1 mmol, 9 mg), methoxyacetaldehyde **2i** (80 mM, 0.4 mmol, 29.6 mg), ATP (30 mM) and $MgCl_2$ (30 mM). 7.7 mg of product was isolated as a white solid, representing a 28% isolated yield.

Major anomer:

^{1}H NMR (500 MHz) 3.48 (s,3H, H6), 3.86 (dd, J = 5.2, 4.3 Hz, 1H, H2), 3.91 (m, 1H, H5),4.06 (m, 1H, H5), 4.43 (ddd, J = 7.2, 5.3, 2.8 Hz, 1H, H4), 4.51 (dd, J= 4.4, 2.9 Hz, 1H, H3), 5.36 (d, J = 5.2 Hz, 1H, H1).

^{13}C NMR (125 MHz) 57.72 (C6), 63.19 (d, J = 4.9 Hz, C5), 69.04 (C3), 79.38 (d, J = 7.9, C4), 86.20 (C2), 99.44 (C1).

^{31}P (400 MHz) 2.94 (s, P5).

Minor anomer:

^{1}H NMR (500 MHz) 3.51 (s, 3H, H6), 3.93 (m, 1H, H2), 4.0 (m, 1H, H5), 4.16 (m, 1H, H5), 4.18 (m, 1H, H4, overlapping with H5 minor), 4.50 (dd, J = 4.6, 3.6 Hz, 1H, H3), 5.38 (d, J = 4.8 Hz, 1H, H1).

^{13}C (125 MHz) 57.48 (C6), 64.06 (d, J = 5.0 Hz, C5), 67.93 (C3), 79.43 (d, J = 7.8 Hz, C4), 80.07 (C2), 94.61 (C1).

^{31}P (400 MHz) 2.97 (s, P5).

Known impurities have been annotated on the NMR spectra. Peaks not annotated correspond to unknown impurities.

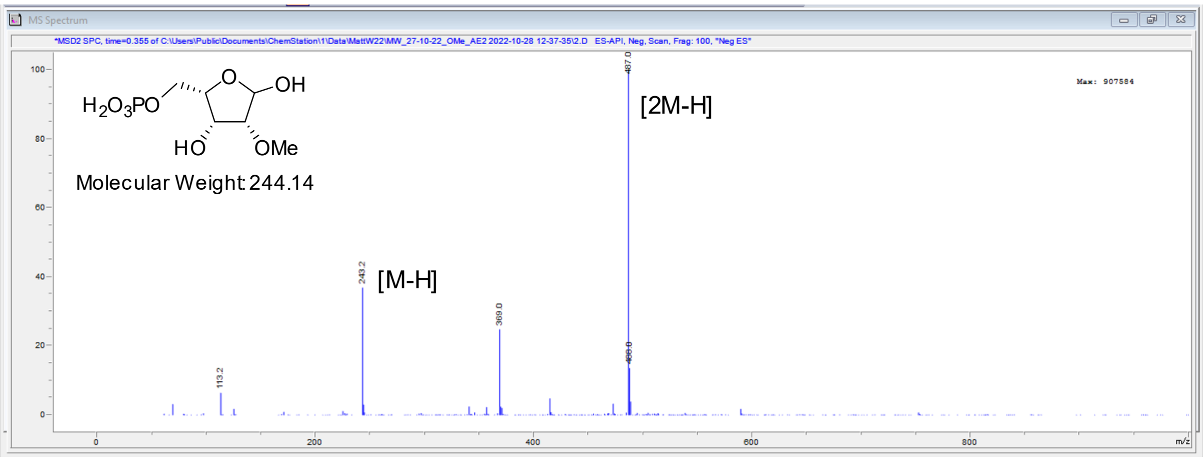

*Figure 44: ESI- Mass spectrum 2-deoxy-2-methoxy-L-lyxose-5-phosphate (**11a**), [M-H] = 243.2*

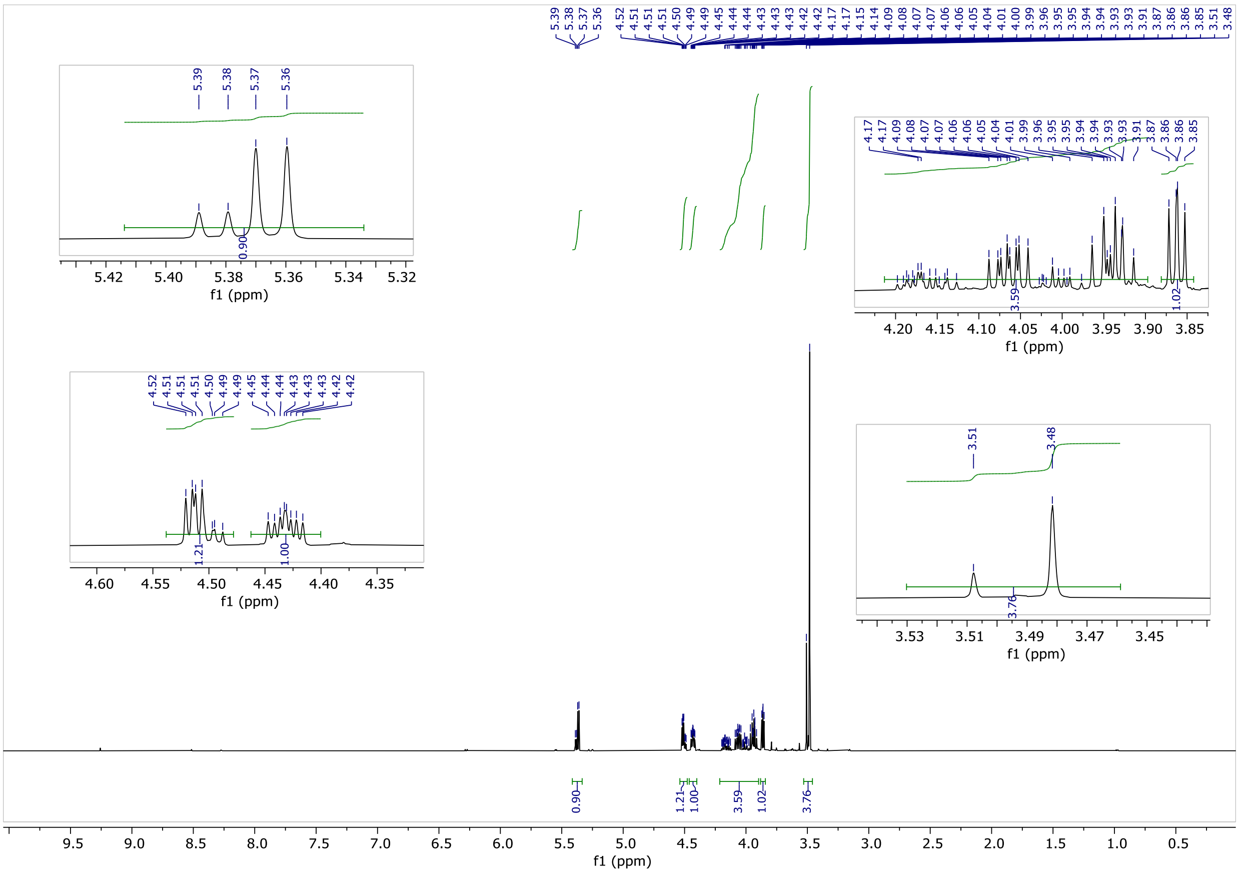

Figure 45: ^{1}H NMR (500 MHz) of 2-deoxy-2-methoxy-L-lyxose-5-phosphate **11a**

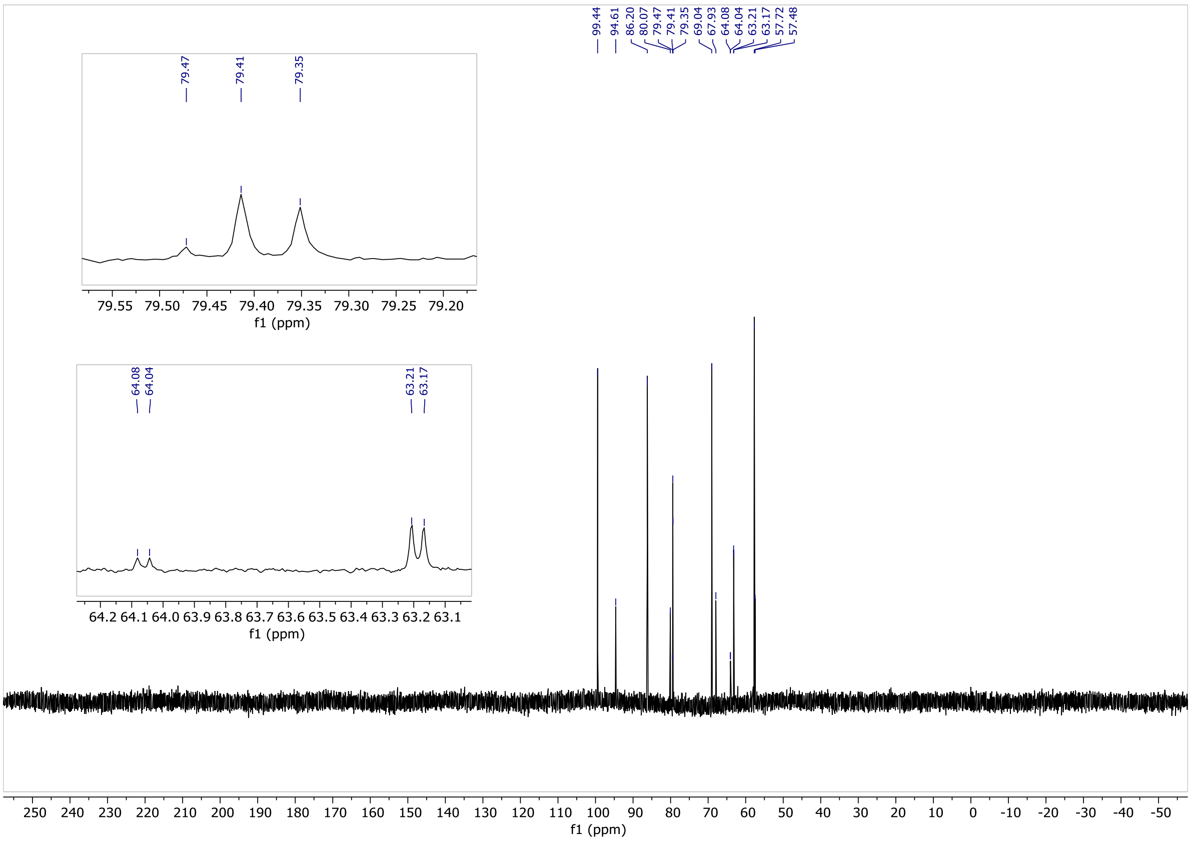

Figure 46: ^{13}C NMR (125 MHz) of 2-deoxy-2-methoxy-L-lyxose-5-phosphate **11a**

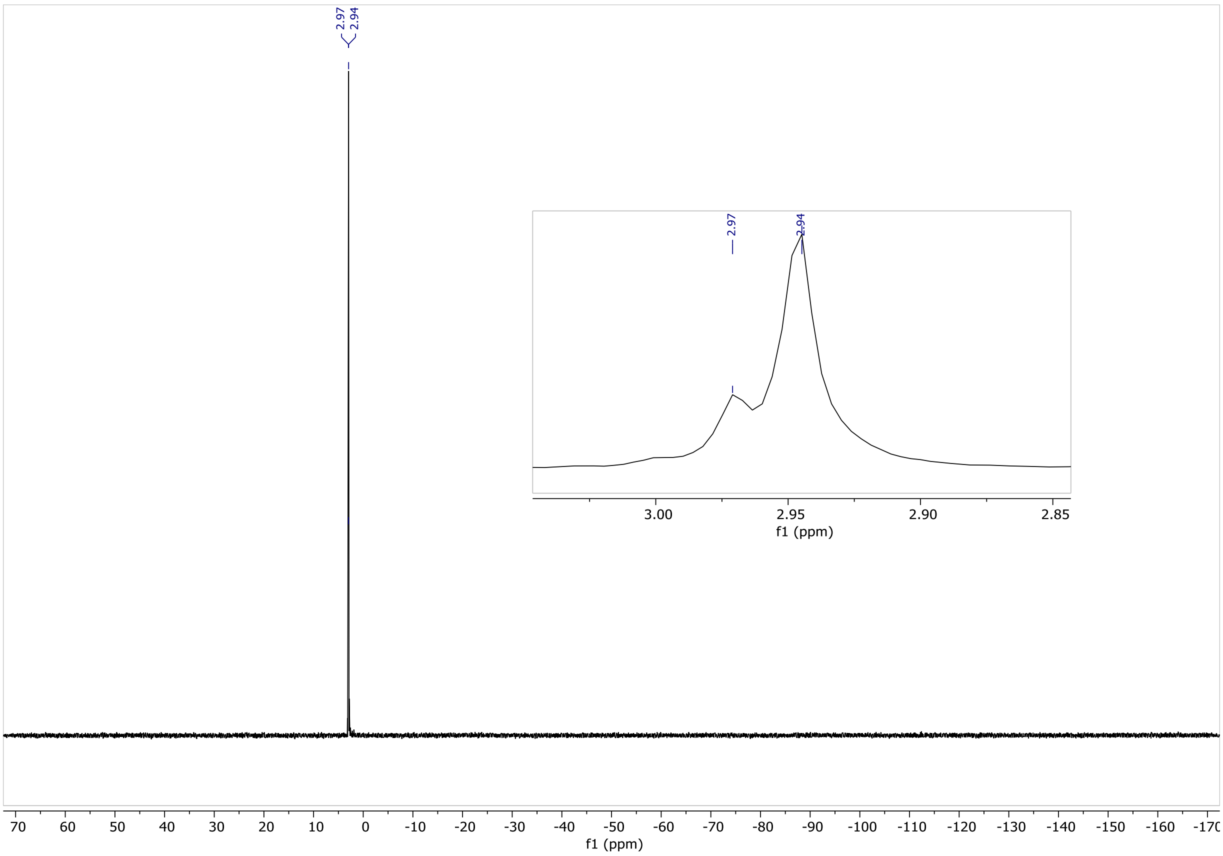

Figure 47: ^{31}P NMR (400 MHz) of 2-deoxy-2-methoxy-L-lyxose-5-phosphate ***11a***

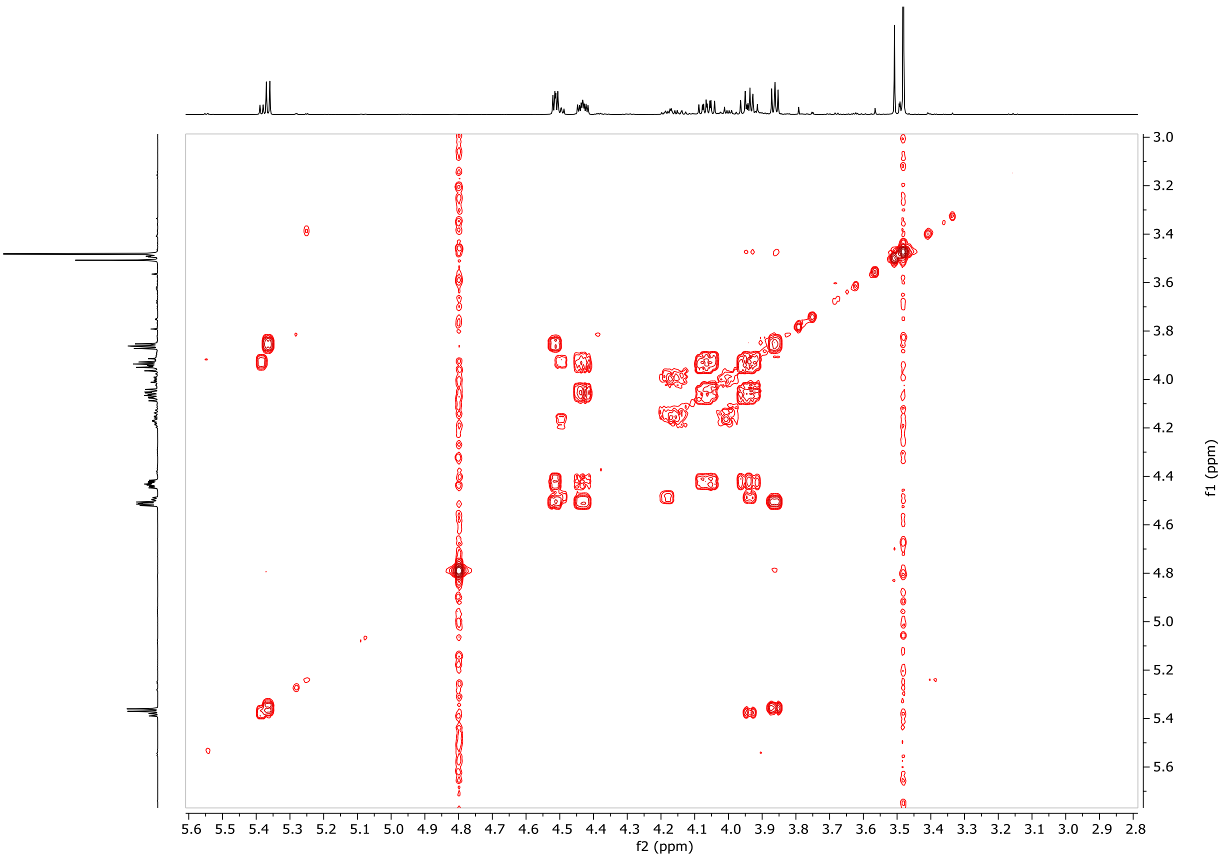

Figure 48: ^{1}H-^{1}H COSY NMR (500 MHz) of 2-deoxy-2-methoxy-L-lyxose-5-phosphate ***11a***

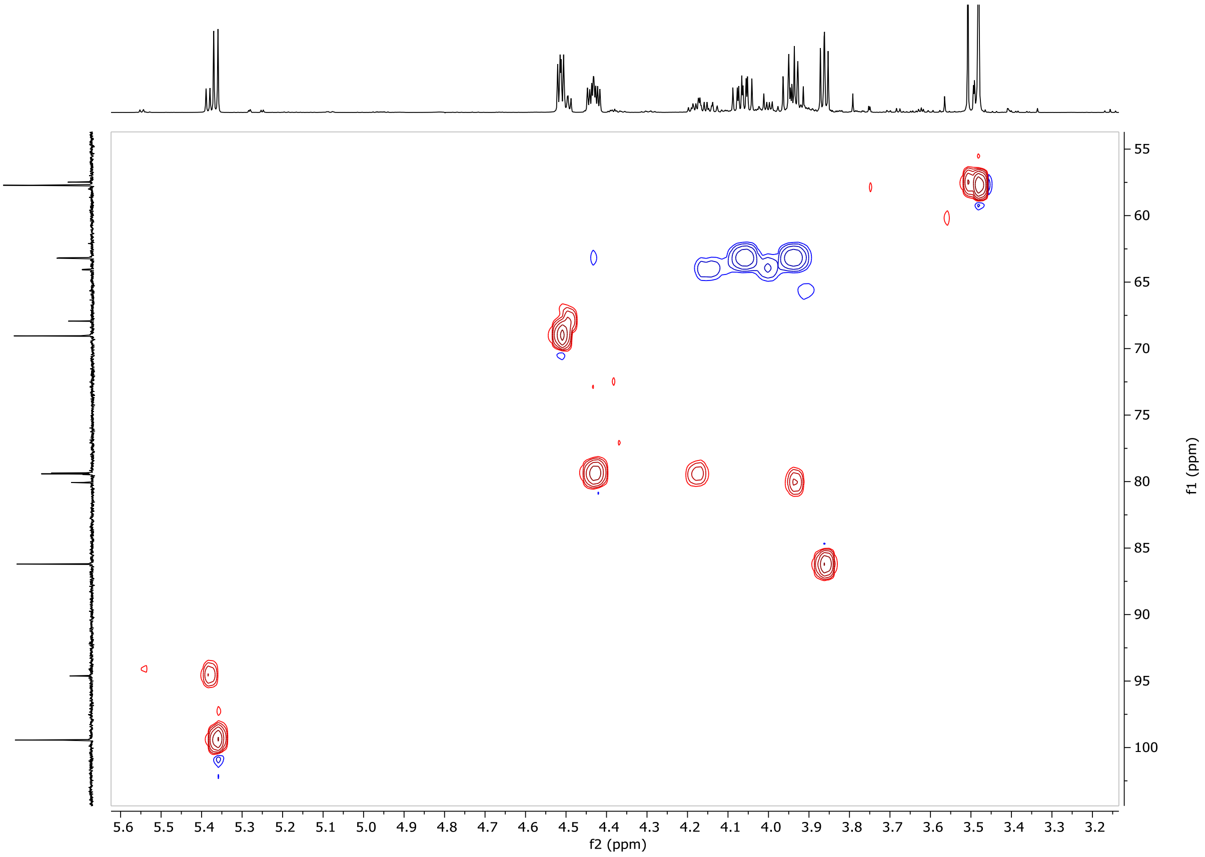

Figure 49: ^{1}H-^{13}C HSQC NMR (500 MHz) of 2-deoxy-2-methoxy-L-lyxose-5-phosphate ***11a***

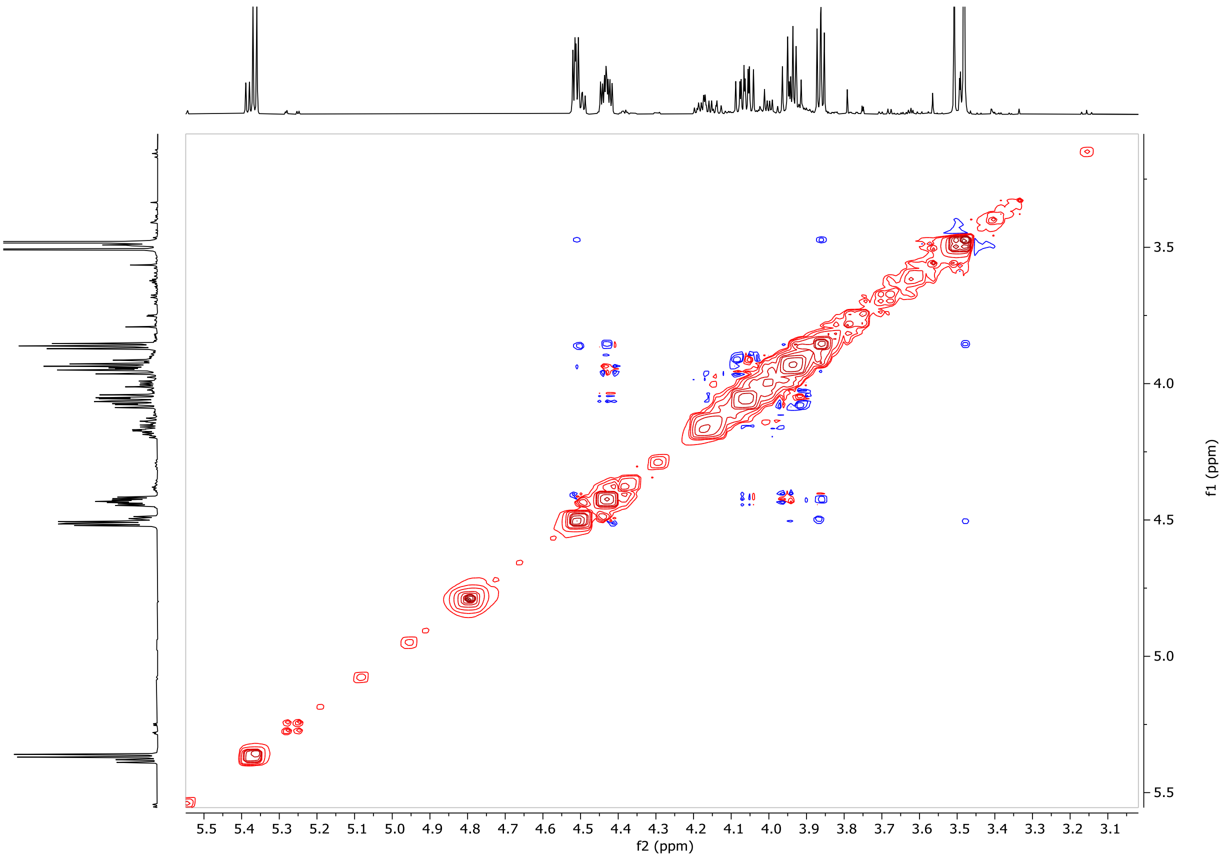

*Figure 50: ^{1}H-^{1}H NOESY (500 MHz) NMR of 2-deoxy-2-methoxy-L-lyxose-5-phosphate **11a***

11.2.3 2-deoxy-2-methyoxy(ethoxy)-L-lyxose-5-phosphate (12a)

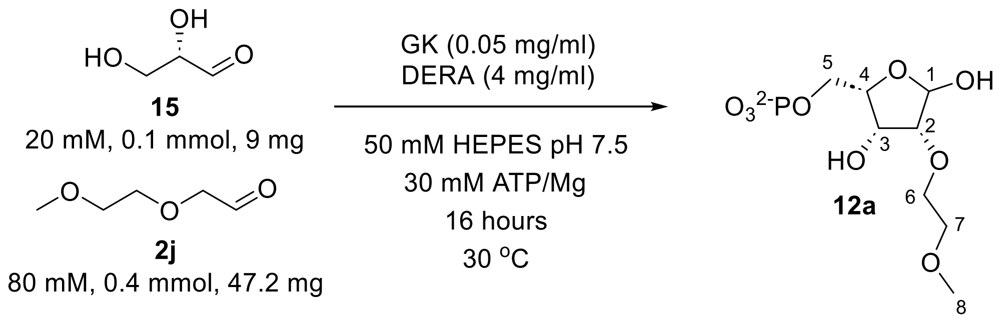

Title compound **12a** was prepared accord to the general procedure using L-glyceraldehyde **15** (20 mM, 0.1 mmol, 9 mg), methoxy(ethoxy)acetaldehyde **2j** (80 mM, 0.4 mmol, 47.2 mg), ATP (30 mM) and $MgCl_2$ (30 mM). 12.9 mg of product was isolated as a white solid, representing a 39% isolated yield.

Major Anomer:

^{1}H NMR (500 MHz) 3.40 (s,3H, H8), 3.67 (m, 2H, H7), 3.80 (m, 2H, H6), 3.95 (m, 1H, H5), 3.97 (m, 1H, H2, overlapping with H5), 4.06 (m, 1H, H5), 4.42 (ddd, J = 7.8, 5.1, 2.9 Hz, 1H, H4), 4.47 (dd, J = 4.3, 2.9 Hz, 1H, H3), 5.38 (d, J = 5.0 Hz, 1H, H1).

^{13}C NMR (125 MHz) 58.02 (H8), 63.26 (d, J= 4.8 Hz, H5), 69.03 (H6), 69.52 (H3), 70.97 (H7), 79.34 (d, J = 7.9 Hz, H4), 85.13 (H2), 99.56 (H1).

^{31}P (400 MHz) 2.94 (s, P5).

Minor Anomer:

^{1}H NMR (500 MHz) 3.40 (s,3H, H8), 3.67 (m, 2H, H7, overlapping with major anomer), 3.80 (m, 2H, H6, overlapping with major anomer), 4.00 (m, 1H, H5), 4.04 (m, 1H, H2), 4.15 (m, 1H, H5), 4.18 (m, 1H, H4, overlapping with H5), 4.46 (m, 1H, H3 overlapping with major anomer)), 5.38 (d, J = 4.4 Hz, 1H, H1 overlapping with major anomer H1).

^{13}C NMR (125 MHz) 58.02 (H8) 64.12 (d, J = 5.0 Hz, H5) 68.37 (H3) 68.74 (H6) 71.03 (H7) 78.97 (H2) 79.38 (d, J = 7.8 Hz, H4) 94.76 (H1).

^{31}P (400 MHz) 2.97 (s, P5).

Known impurities have been annotated on the NMR spectra. Peaks not annotated correspond to unknown impurities.

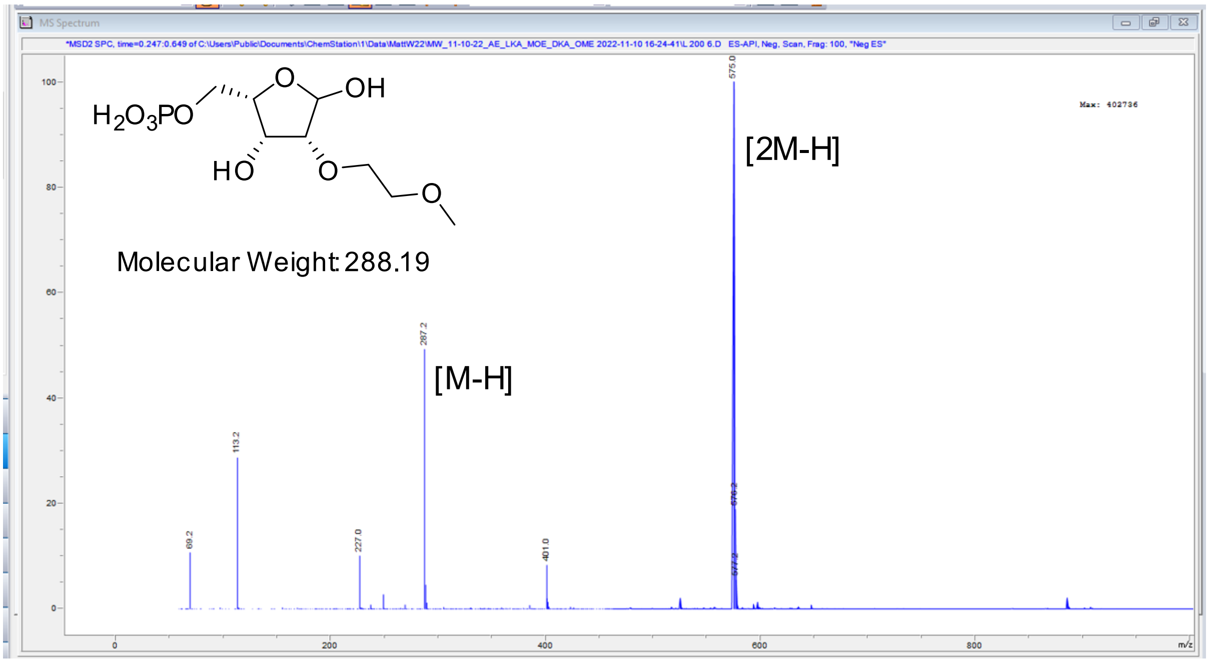

*Figure 51: ESI- Mass spectrum of 2-deoxy-2-methoxy(ethoxy)-L-lyxose-5-phosphate (**12a**), [M-H] = 287.2*

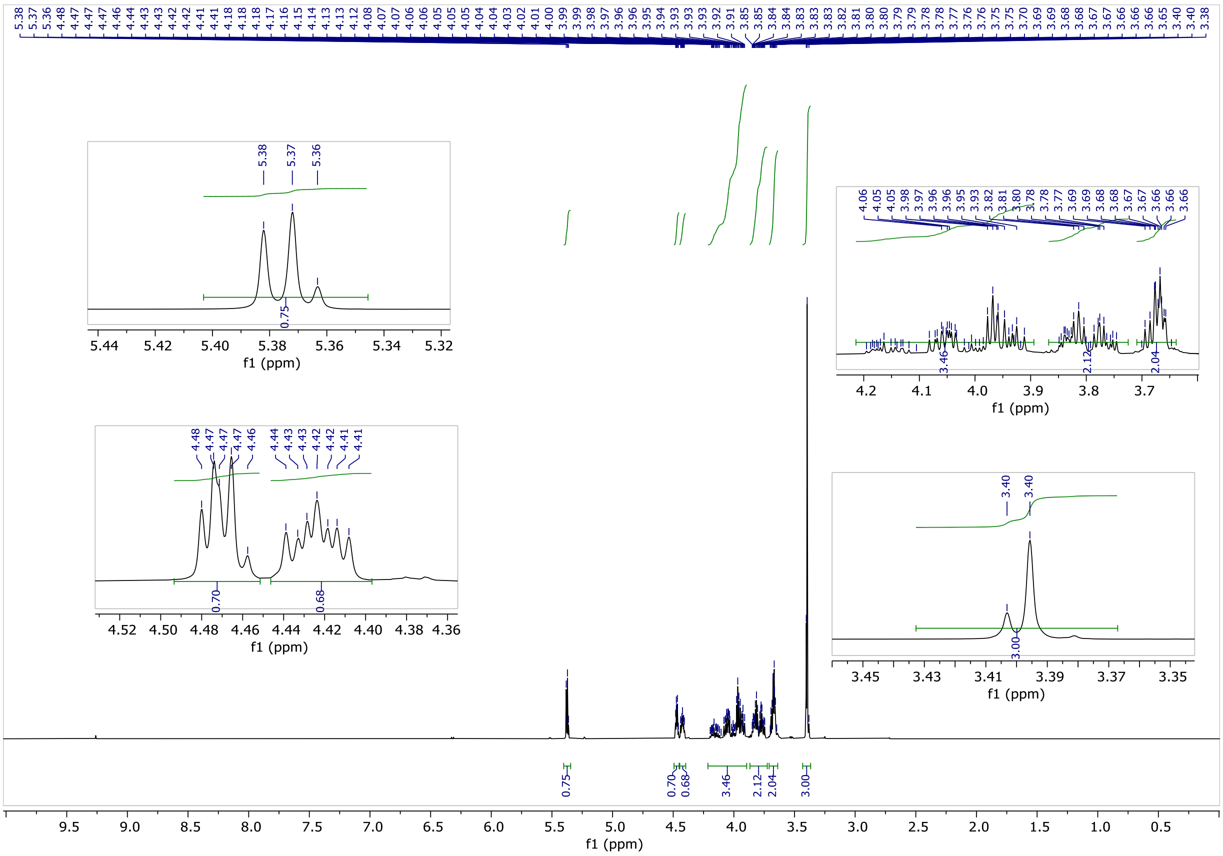

Figure 52: ^{1}H NMR (500 MHz) of 2-deoxy-2-methoxy(ethoxy)-L-lyxose-5-phosphate ***12a***

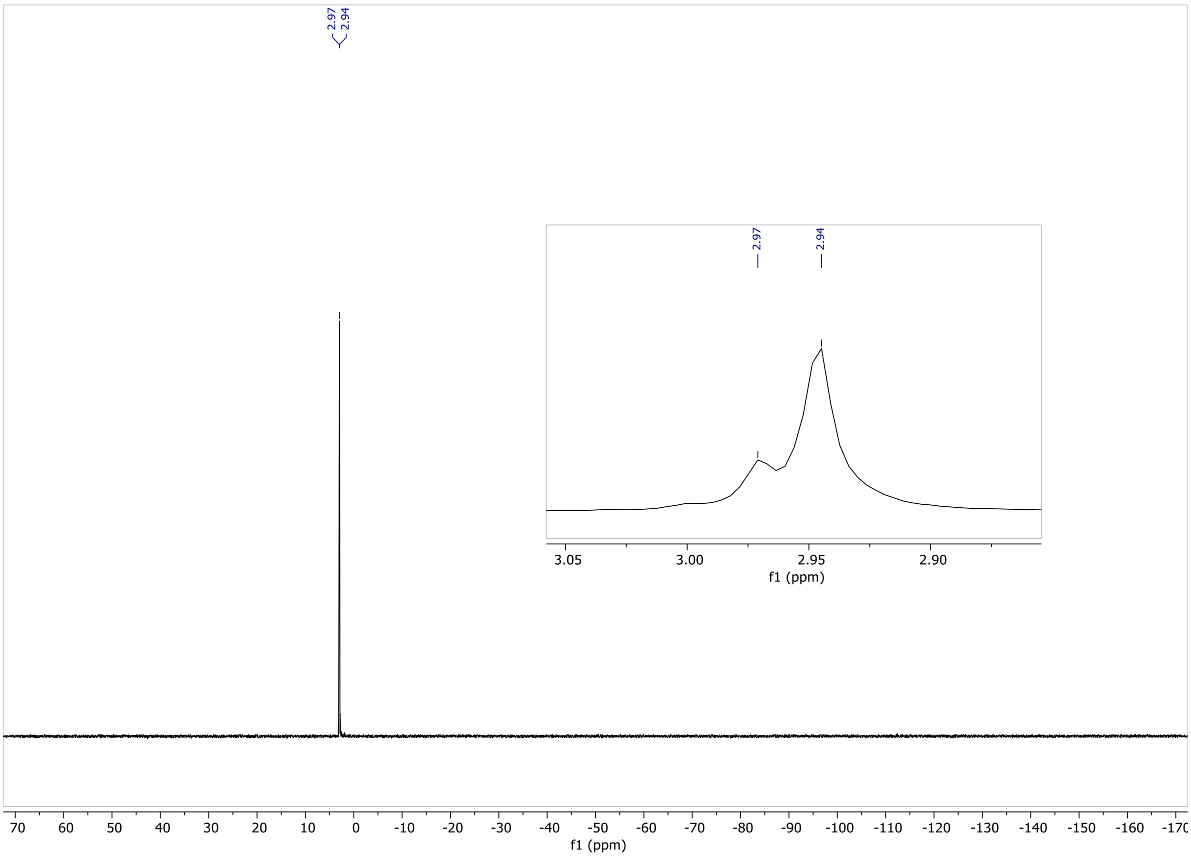

Figure 53: ^{31}P NMR (400 MHz) of 2-deoxy-2-methoxy(ethoxy)-L-lyxose-5-phosphate ***12a***

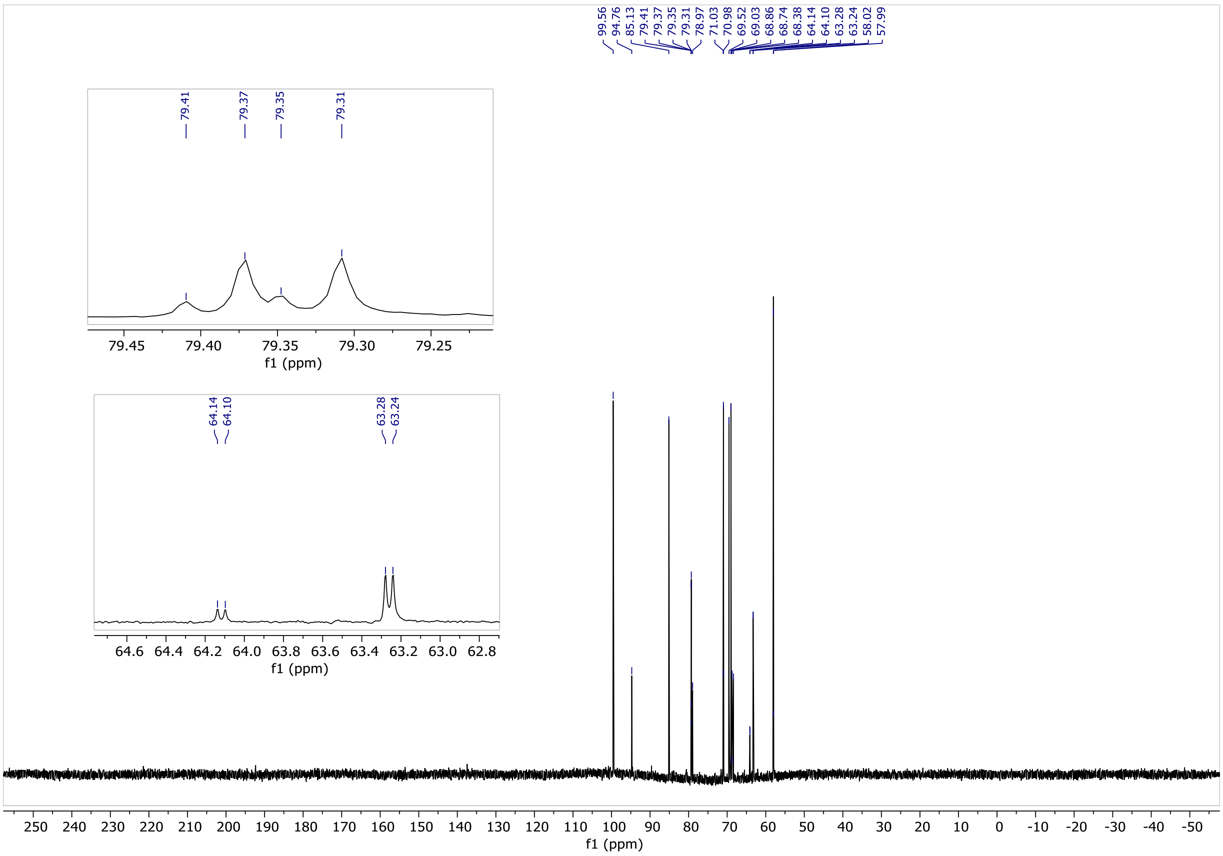

*Figure 54: ^{13}C NMR of 2-deoxy-2-methoxy(ethoxy)-L-lyxose-5-phosphate **12a***

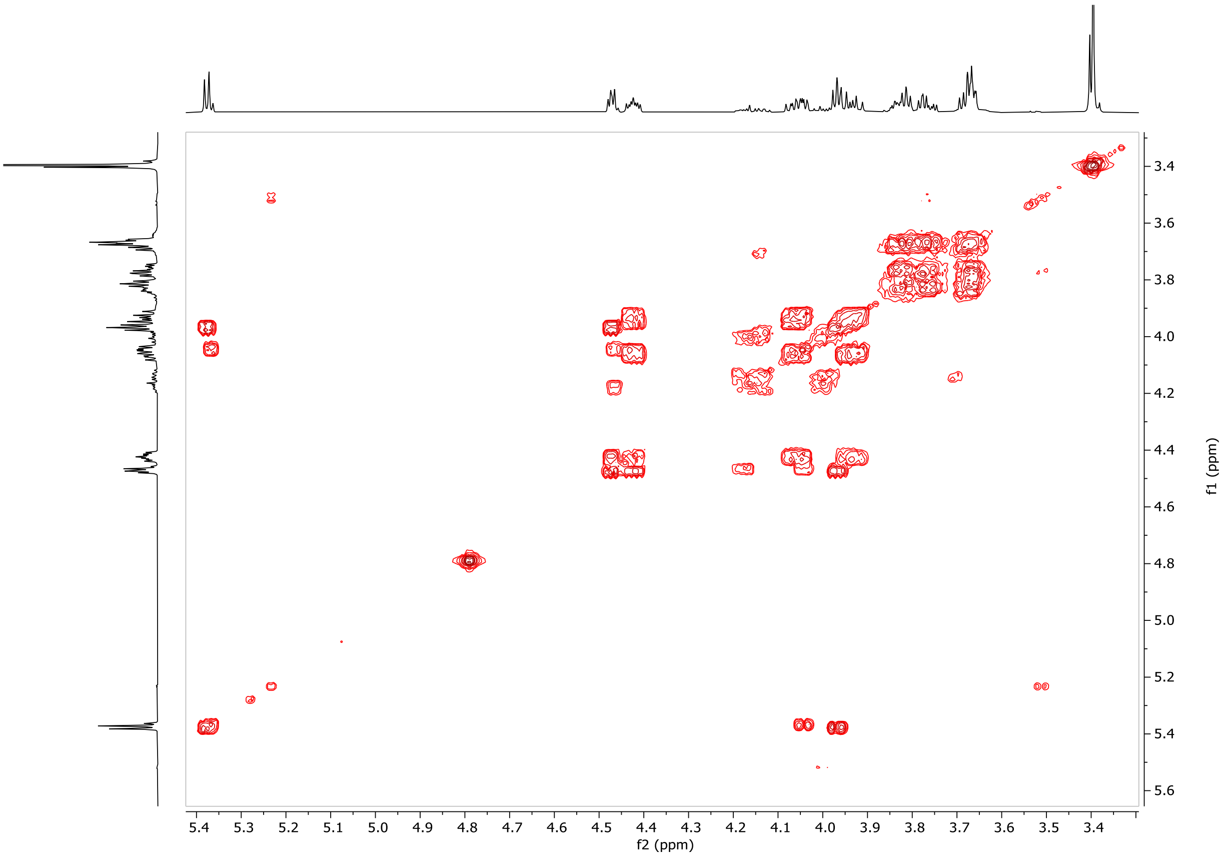

*Figure 55: ^{1}H-^{1}H COSY NMR (500 MHz) of 2-deoxy-2-methoxy(ethoxy)-L-lyxose-5-phosphate **12a***

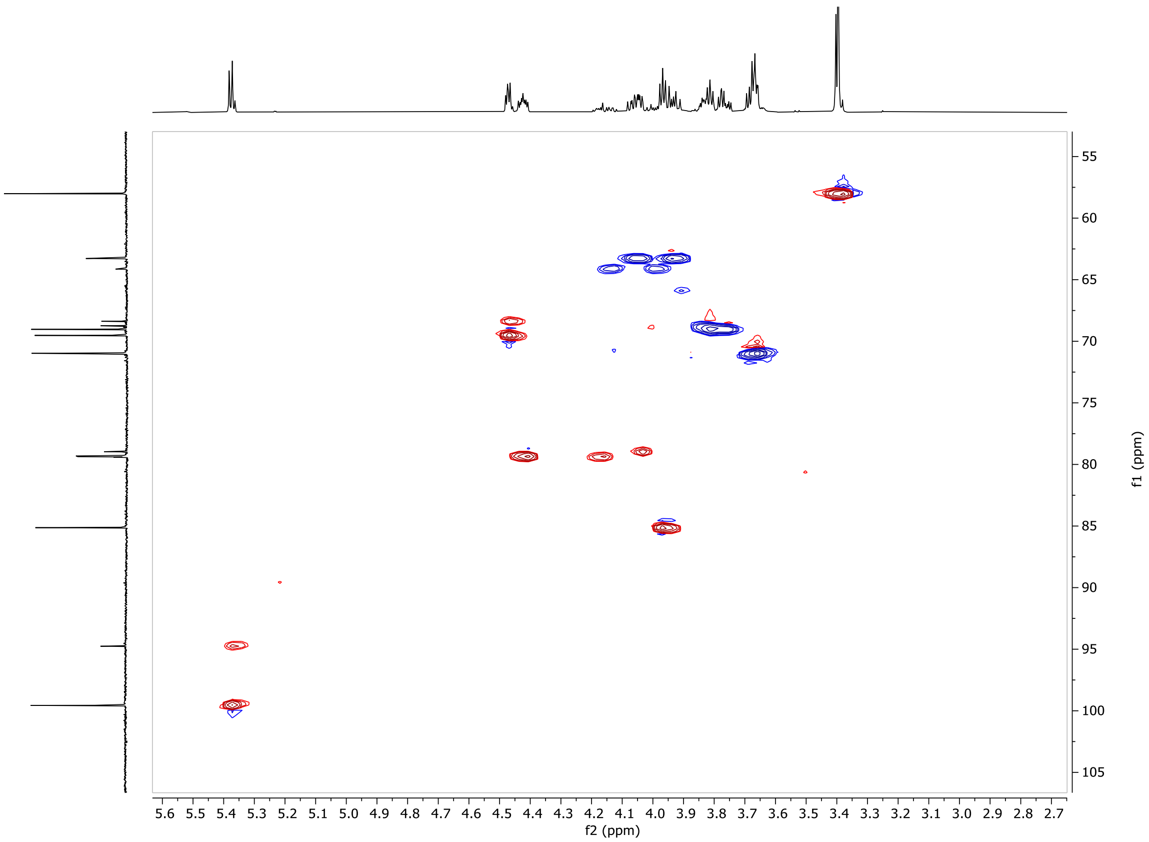

*Figure 56: ^{1}H-^{13}C NMR (500 MHz) of 2-deoxy-2-methoxy(ethoxy)-*L*-lyxose-5-phosphate* ***12a***

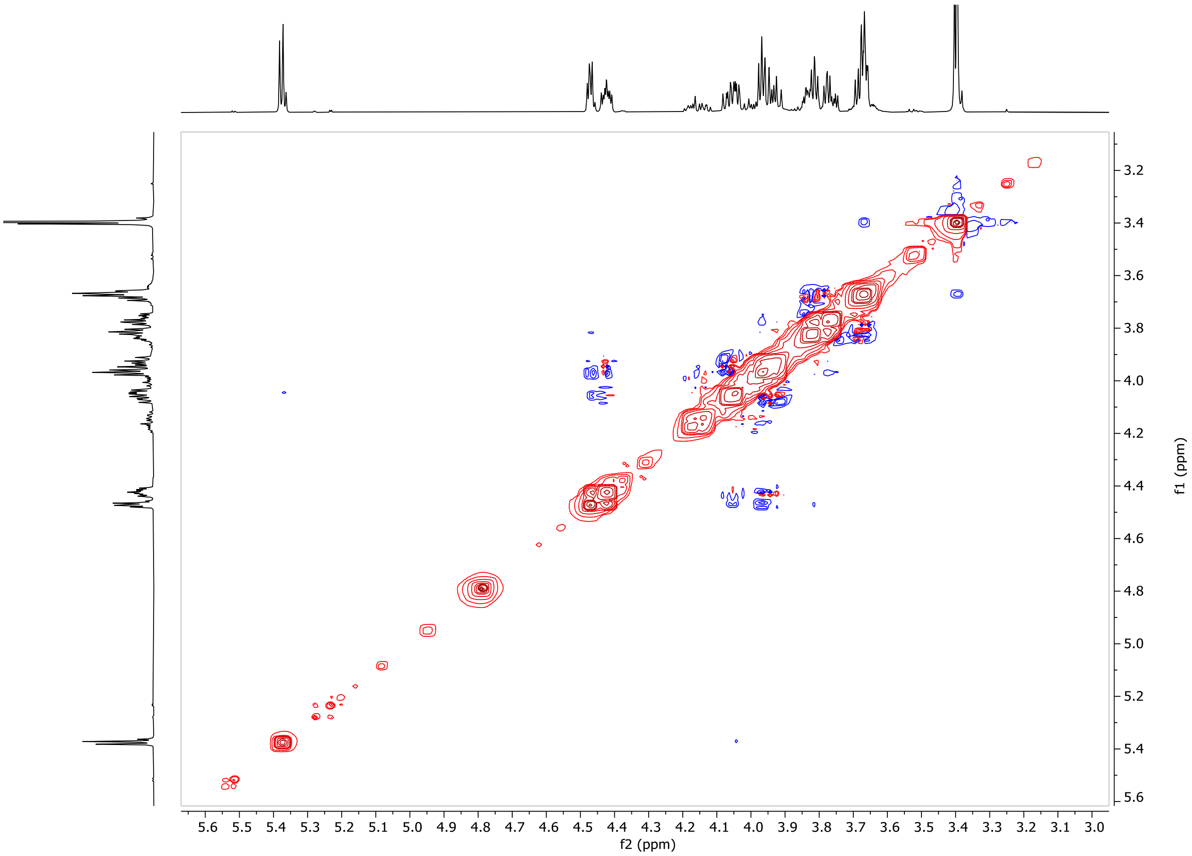

*Figure 57: ^{1}H-^{1}H NOESY NMR (500 MHz) of 2-deoxy-2-methoxy(ethoxy)-*L*-lyxose-5-phosphate*

11.2.4 2-deoxy-2-benzoxyl-L-Lyxose-5-phosphate (**13a**)

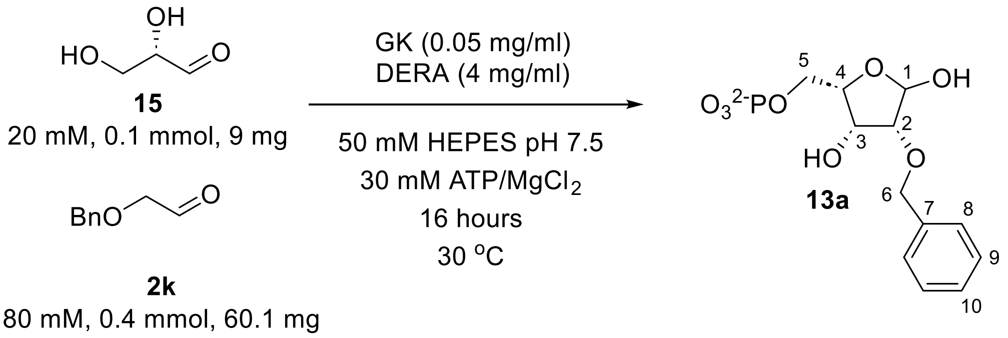

Title compound **13a** was prepared accord to the general procedure using L-glyceraldehyde **15** (20 mM, 0.1 mmol, 9 mg), benzyloxyacetaldehyde **2k** (80 mM, 0.4 mmol, 60.1 mg), ATP (30 mM) and $MgCl_2$ (30 mM). 9.2 mgs of product was isolated as a white solid, representing a 25% isolated yield.

^{31}P NMR indicates the presence of a small amount of ADP alongside an unknown phosphate contaminant. A lack of interaction with the ^{1}H NMR, using ^{1}H-^{31}P HMBC, for this unknown peak suggests it may be due to free phosphate, which can be formed by the degradation of L-G3P into methylglyoxal at neutral pH. Due to the nature of ^{31}P NMR being only semi-quantitative the exact ratio of impurities to product is difficult to accurately quantify.

Major Anomer:

^{1}H NMR (500 MHz) 3.91 (m, 1H, H5), 4.01 (m, 1H, H2), 4.03 (m, 1H, H5), 4.38 (ddd, J = 7.8, 5.5, 3.0 Hz, 1H, H4), 4.43 (dd, *J* = 4.4, 2.9 Hz, 1H, H3), 4.64 (d, J = 11.6, 1H, H6), 4.73 (d, J = 11.7, 1H, H6), 5.38 (d, J = 5.1, 1H, H1), 7.46 (m , 5H, H8, H9, H10, overlapping with minor anomer).

^{13}C NMR (125 MHz) 63.14 (d, J = 4.8 Hz, C5), 69.42 (C3), 72.15 (C6), 79.46 (d, J = 7.8 Hz, C4), 84.03 (C2), 99.85 (C1), 128.21-128.69 (C7,C8, C9, C10 overlapping with minor).

^{31}P (400 MHz) 1.57 (s, P5).

Minor Anomer: ,

^{1}H NMR (500 MHz) 3.97 (m, 1H, H5), 4.05 (m, 1H H2), 4.11 (m, 1H, H5), 4.11 (m, 1H, H4), 4.40 (m, 1H, H3), 4.72 (m, 2H, H6, overlapping with major anomer/water), 5.29 (d, J = 4.9 Hz, 1H, H1), 7.46 (m , 5H, H8, H9, H10, overlapping with major anomer).

^{13}C NMR (125 MHz) 63.94 (d, J = 4.9 Hz, C5), 68.37 (C3), 71.91 (C6), 79.40 (d, J = 7.8 Hz, C4), 77.62 (C2), 94.72 (C1), 128.21-128.69 (C7,C8, C9, C10 overlapping with major).

^{31}P (400 MHz) 1.60 (s, P5).

Known impurities have been annotated on the NMR spectra. Peaks not annotated correspond to unknown impurities.

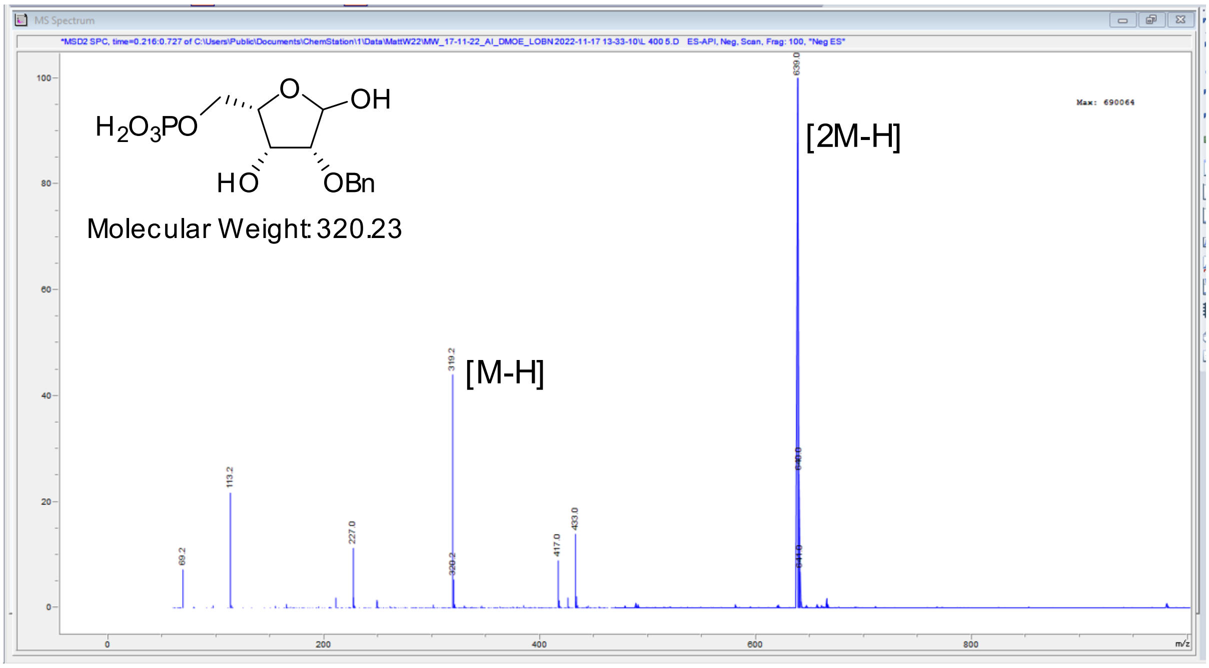

*Figure 58: ESI- mass spectrum of 2-deoxy-2-benzoxyl-L-lyxose-5-phosphate (**13a**), [M-H] = 319.2*

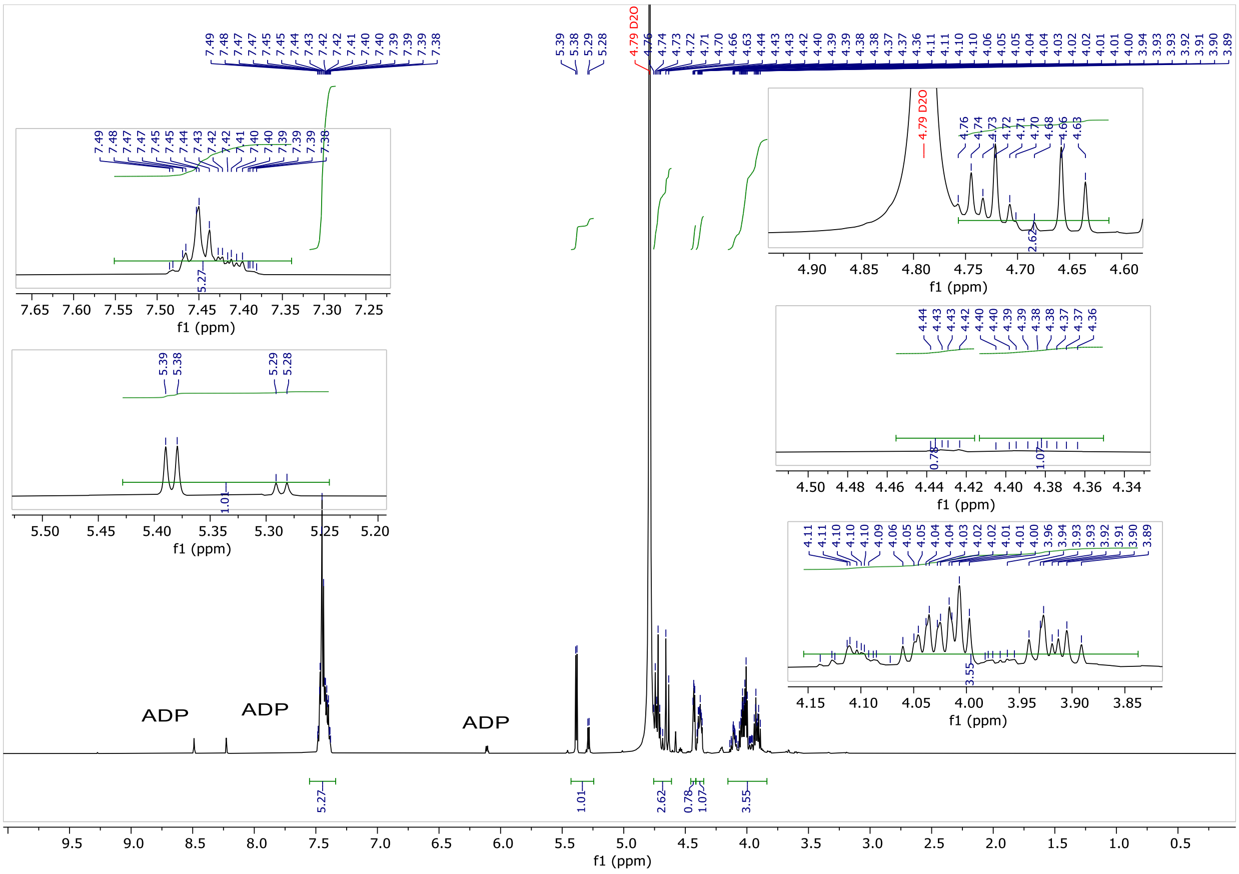

*Figure 59: ^{1}H NMR (500 MHz) of 2-deoxy-2-benzoxyl-L-lyxose-5-phosphate **13a***

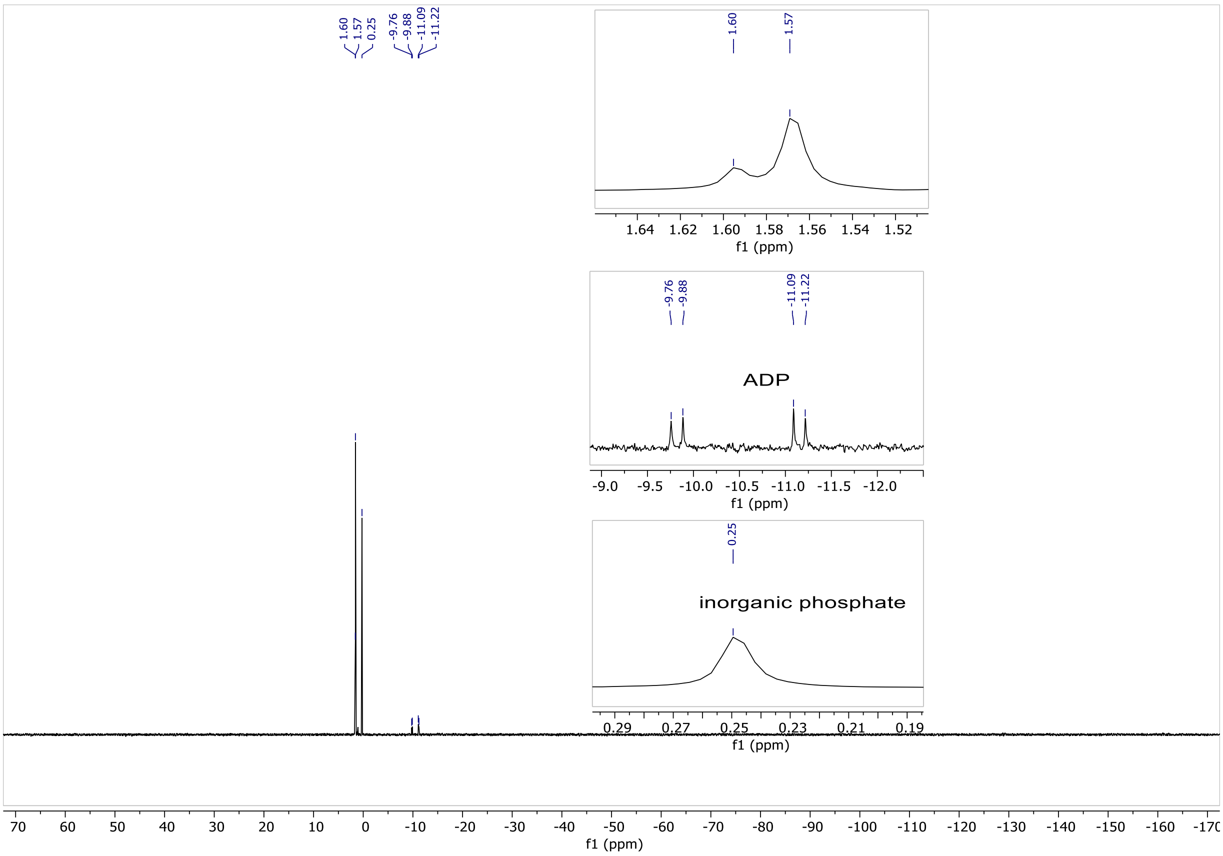

*Figure 60: ^{31}P NMR (400 MHz) of 2-deoxy-2-benzoxyl-L-lyxose-5-phosphate **13a***

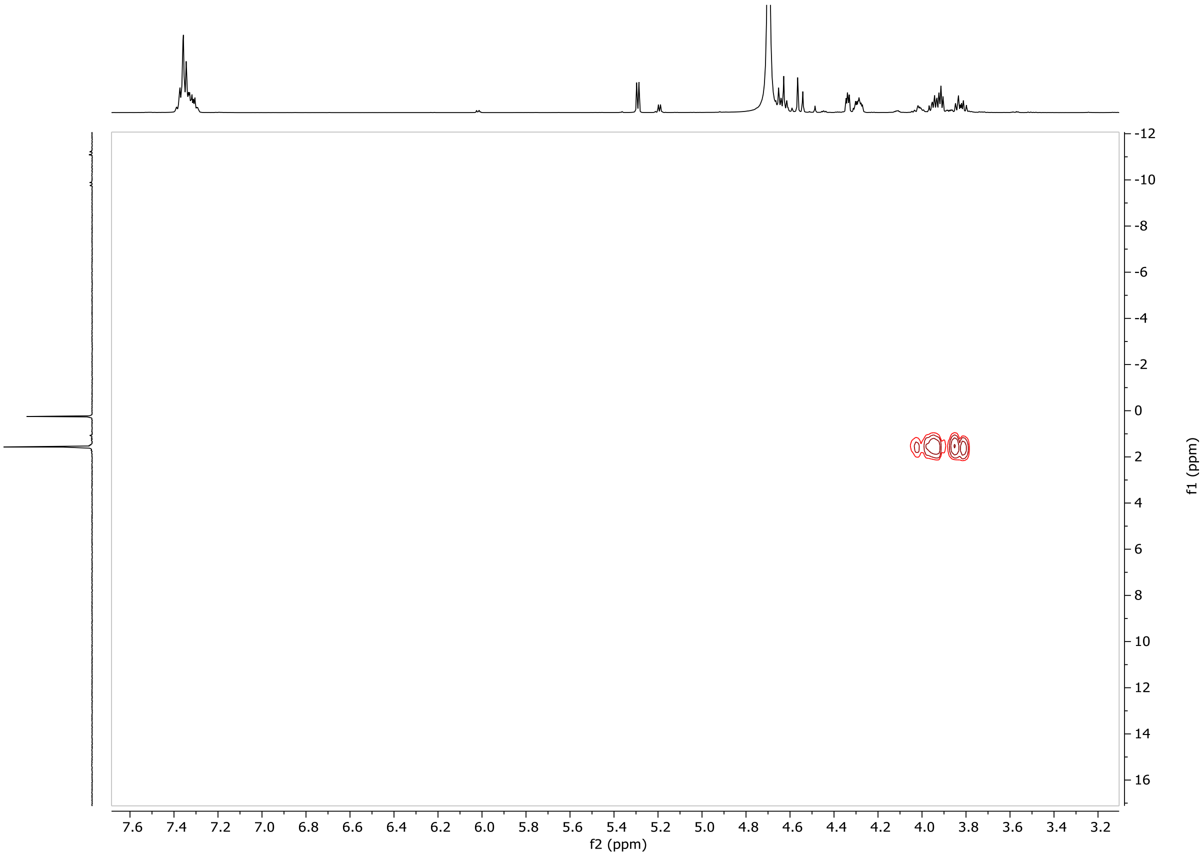

*Figure 61: ^{31}P-^{1}H HMBC NMR (400MHz) of 2-deoxy-2-benzoxyl-L-lyxose-5-phosphate **13a***

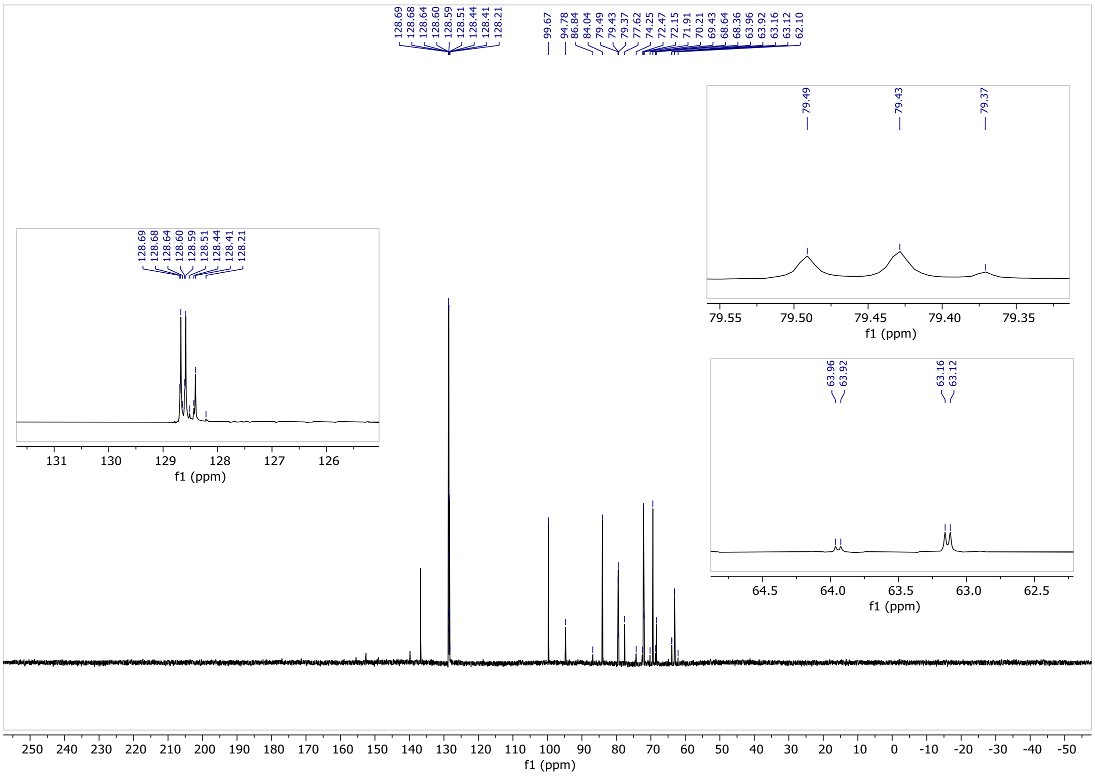

Figure 62: ^{13}C NMR (500 MHz) spectrum of 2-deoxy-2-benzoxyl-L-lyxose-5-phosphate **13a**

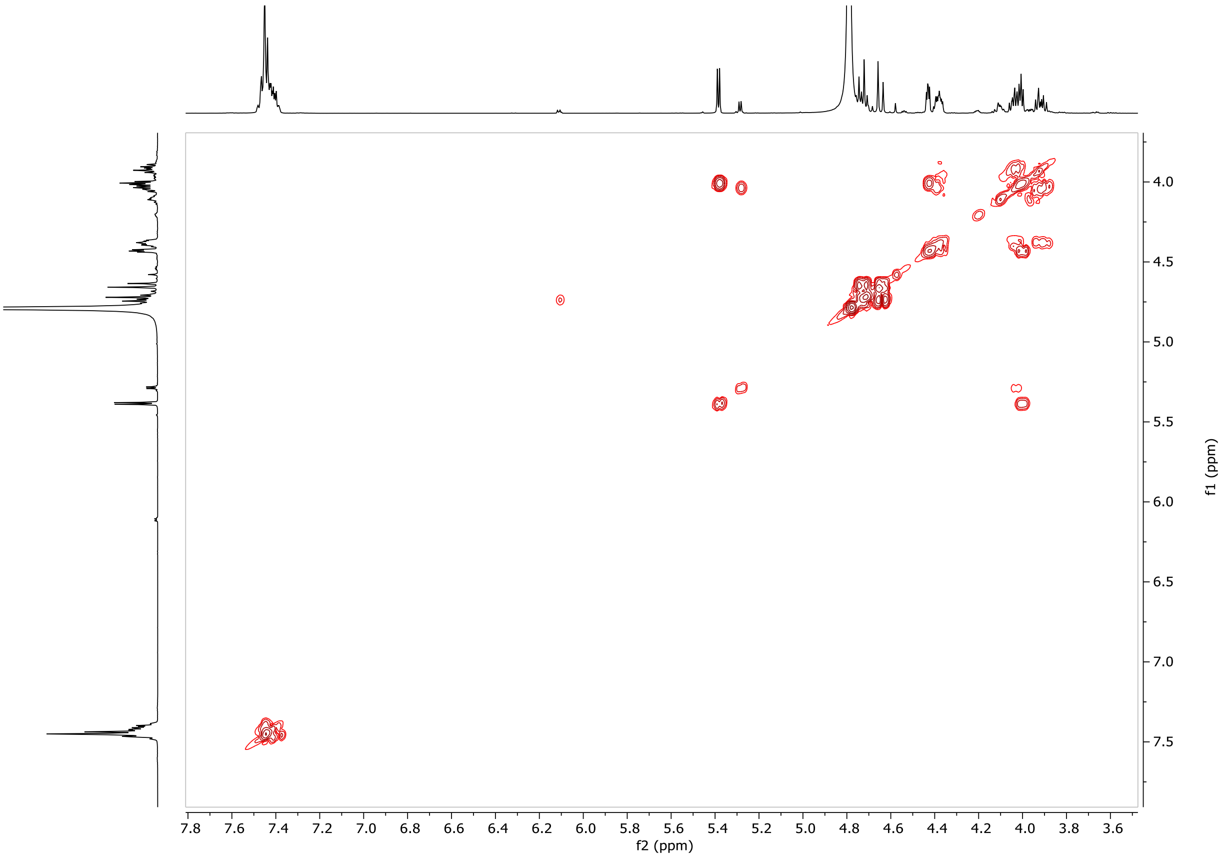

Figure 63: ^{1}H-^{1}H NMR (500 MHz) of 2-deoxy-2-benzoxyl-L-lyxose-5-phosphate **13a**

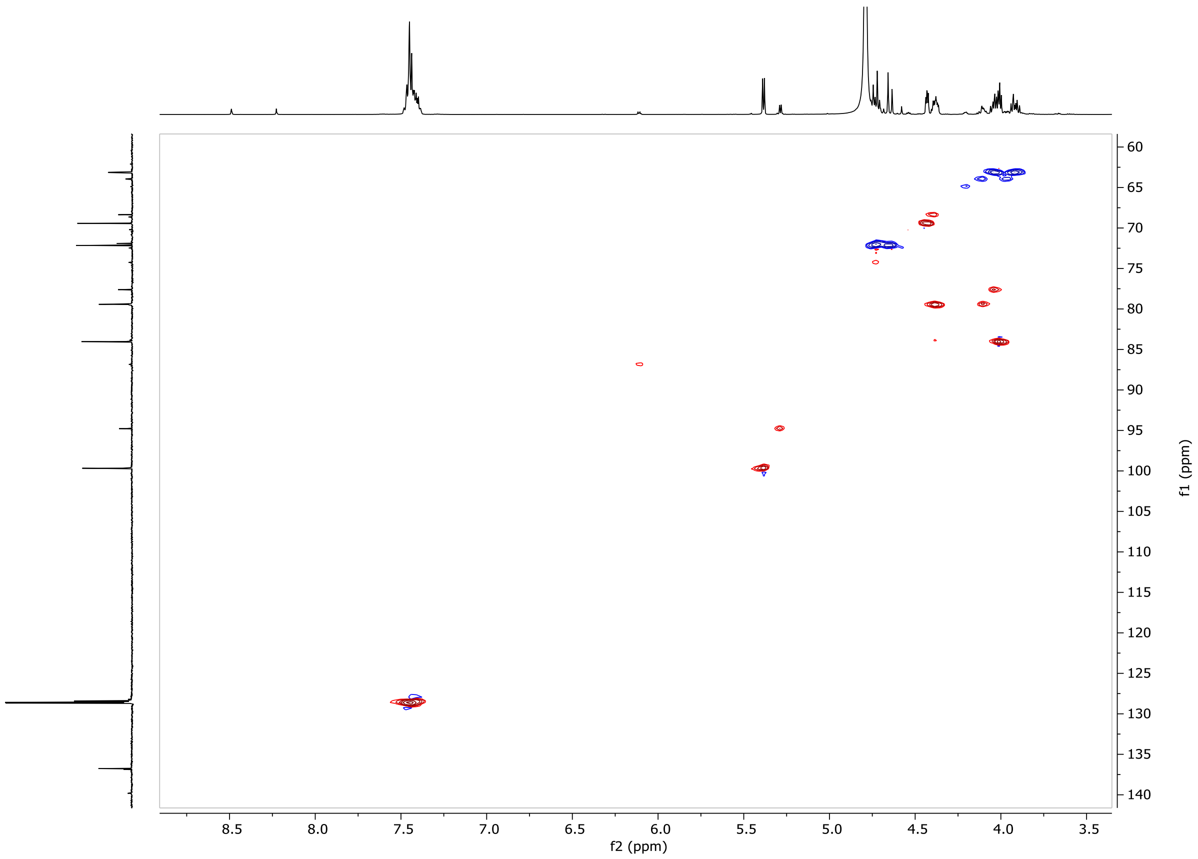

*Figure 64: ^{1}H-^{13}C HSQC (500 MHz) of 2-deoxy-2-benzoxyl-L-lyxose-5-phosphate **13a***

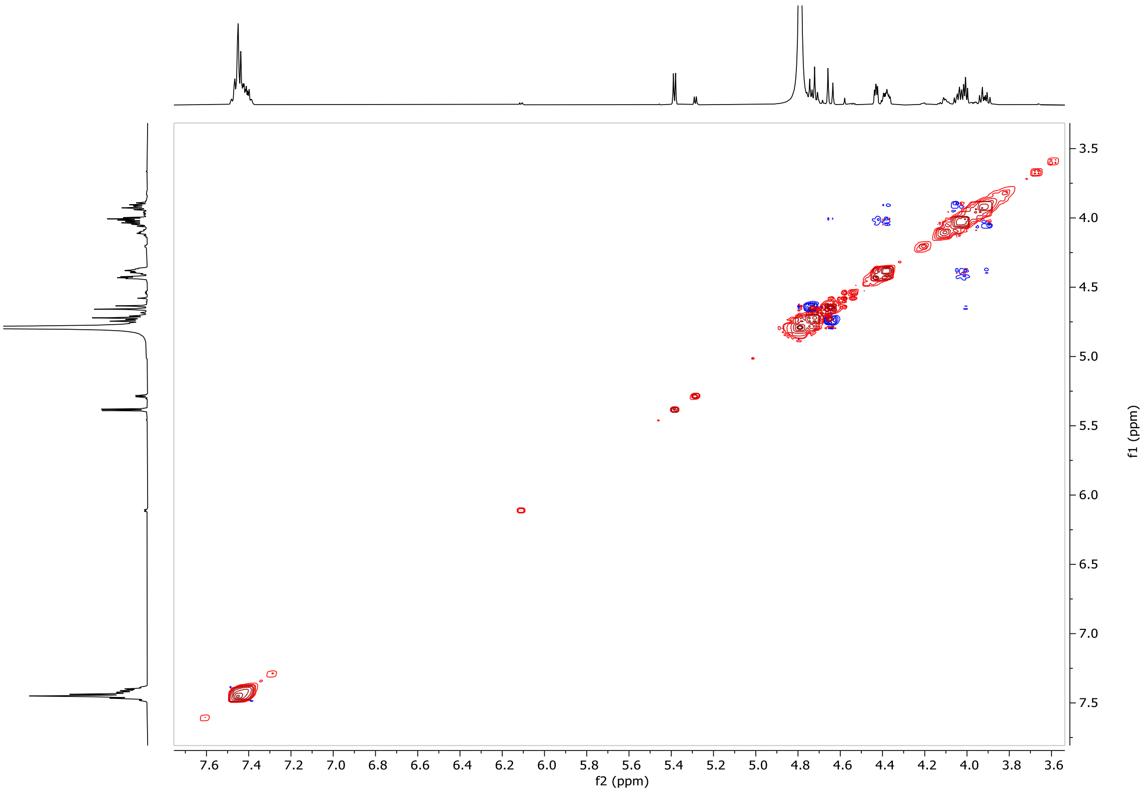

*Figure 65: ^{1}H-^{1}H NOESY NMR (500 MHz) of 2-deoxy-2-benzoxyl-L-lyxose-5-phosphate **13a***

11.2.5 2-Deoxy-2-fluoro-L-lyxose-5-phosphate (19a)

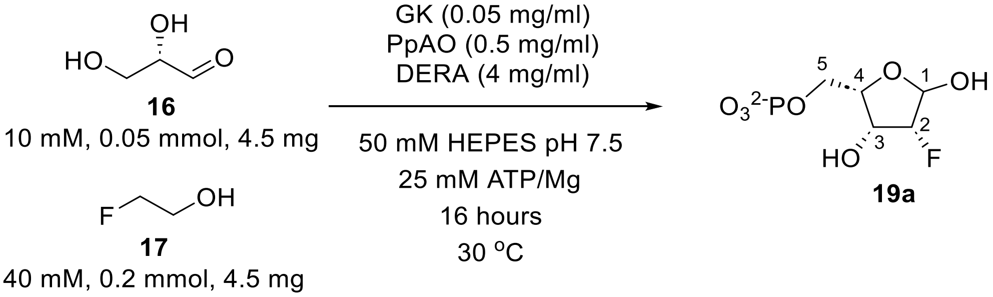

To a 15 ml falcon tube, L-Glyceraldehyde **16** (10 mM, 0.05 mmol, 9 mg), fluoroethanol **17** (40 mM, 0.2 mmol, 4.5 mg), ATP (15 mM) and $MgCl_2$ (15 mM) were added. HEPES buffer (pH 8.2), and milliQ water were added, so as to give a final buffer concentration of 50 mM and a final reaction volume of 5 ml. The reaction was initiated by addition of $DERA_{WT}$ (4 mg/ml) glycerokinase (0.1 mg/ml) and *Pp*AO (0.5 mg/ml). The reaction mixture was split into 10 x 500 μL reactions. These were incubated in a thermoshaker at 30 °C 750 RPM for 18 hours. After this time, the enzyme was removed using a 10k MWCO filter. The phosphorylated product was then purified using anion exchange chromatography, as previously described, giving 7.9 mgs of **19a** as a white solid. (Due to the presence of multiple unknown contaminants in the NMR, isolated yield has not been determined)

Mass spec shows the desired product mass and ^{1}H NMR shows the presence of desired product alongside a small amount of ADP impurity. However, alongside product peaks 1H and ^{19}F NMR show a complex mixture of products, these include the four expected diastereomers (two sugar anomers at H1, two stereocenters at H2) alongside some unknown impurities.

Multiple overlapping product peaks, alongside several unknown peaks has made the ^{1}H/^{13}C NMR difficult to assign. Tentative assignments have been made by comparison to the previous L-lyxose-5-phosphates.

Four product diastereomers are present in the ^{1}H NMR have been labelled a-d, stereochemical assignment of these has not been carried out.

Diastereomer A

^{1}H NMR (500 MHz, D2O) 3.80-4.16 (H5), 4.50 (m, 1H, H4), 4.53, 4.60 (m, 1H, H3), 4.94, 5.04 (m, 1H, H2), 5.59, 5.60 (1H, H1).

^{13}C NMR (125 MHz, D2O) 62.9-66.2 (C5), 72.2 (C4), 72.80 (C3), 88.55, 90.07 (C2), 94.88 (C1).

^{31}P NMR (400 MHz) 0.75 (s, P1)

Diastereomer B

^{1}H NMR (500 MHz, D2O) 3.80-4.16 (H5), 4.47 (m, 1H H4), 4.65, 4.81 (m,1H, H3), 4.98, 4.88 (m, 1H, H2), 5.53, 5.56 (m, 1H, H1).

^{13}C NMR (125 MHz, D2O) 62.9-66.2 (C5), 70.41 (C4), 69.55 (C3), 94.30, 95.85 (C2), 98.34 (C1).

^{31}P NMR (400 MHz) 0.69 (s, P1)

Diastereomer C

^{1}H NMR (500 MHz, D2O) 3.80-4.16 (m, H3, H4, H5), 4.46, 4.56 (m, 1H, H2), 5.26, 5.30(1H, H1).

^{13}C NMR (125 MHz, D2O) 62.9-66.2 (C5), 67.33-71.05 (C3, C4), 91.01, 92.46 (C2), 87.84 (C1).

^{31}P NMR (400 MHz) 0.63 (s, P1)

Diastereomer D

^{1}H NMR (500 MHz, D2O) 3.80-4.16 (m, H3, H4, H5), 3.91 (m, 1H, H3), 4.44, 4.52 (m, 1H, H2), 5.19, 5.18 (m, 1H, H1).

^{13}C NMR (125 MHz, D2O) 62.9-66.2 (C5), 67.33-71.05 (C3, C4), 93.00, 94.41 (C2), 87.60 (C1).

^{31}P NMR (400 MHz) 0.54 (s, P1)

Known impurities have been annotated on NMR spectra. Peaks not annotated correspond to unknown impurities.

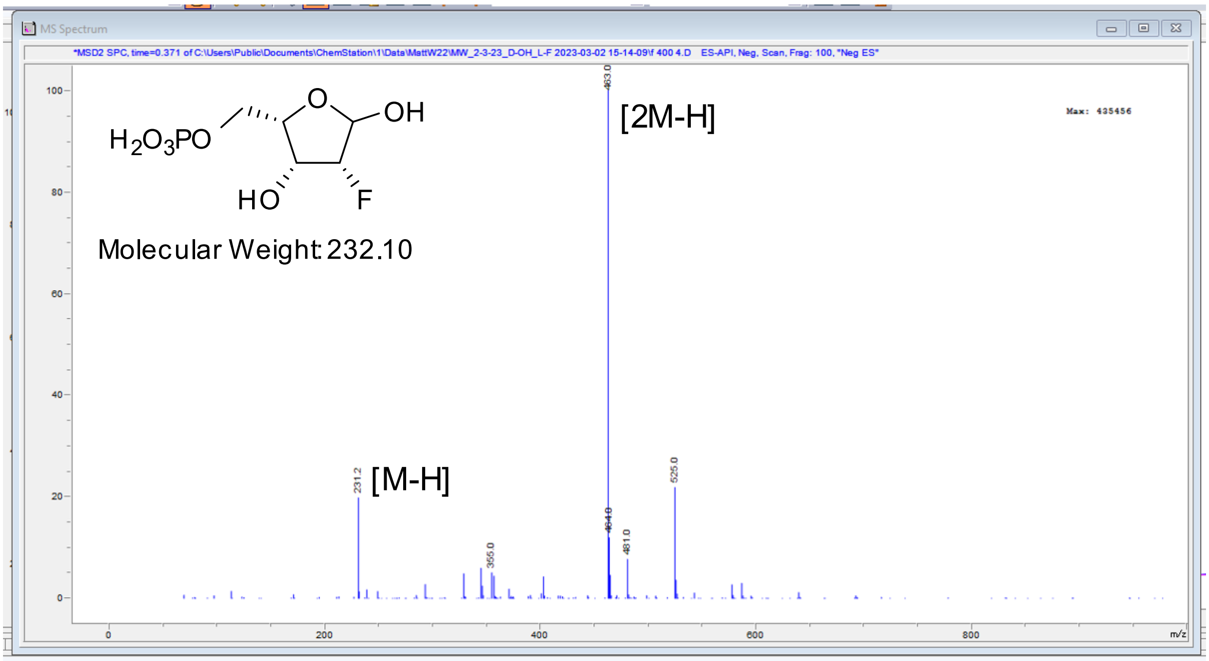

*Figure 66: ESI- mass spectrum of 2-deoxy-2-fluoro-L-lyxose-5-Phosphate (**19a**), [M-H] = 231.2*

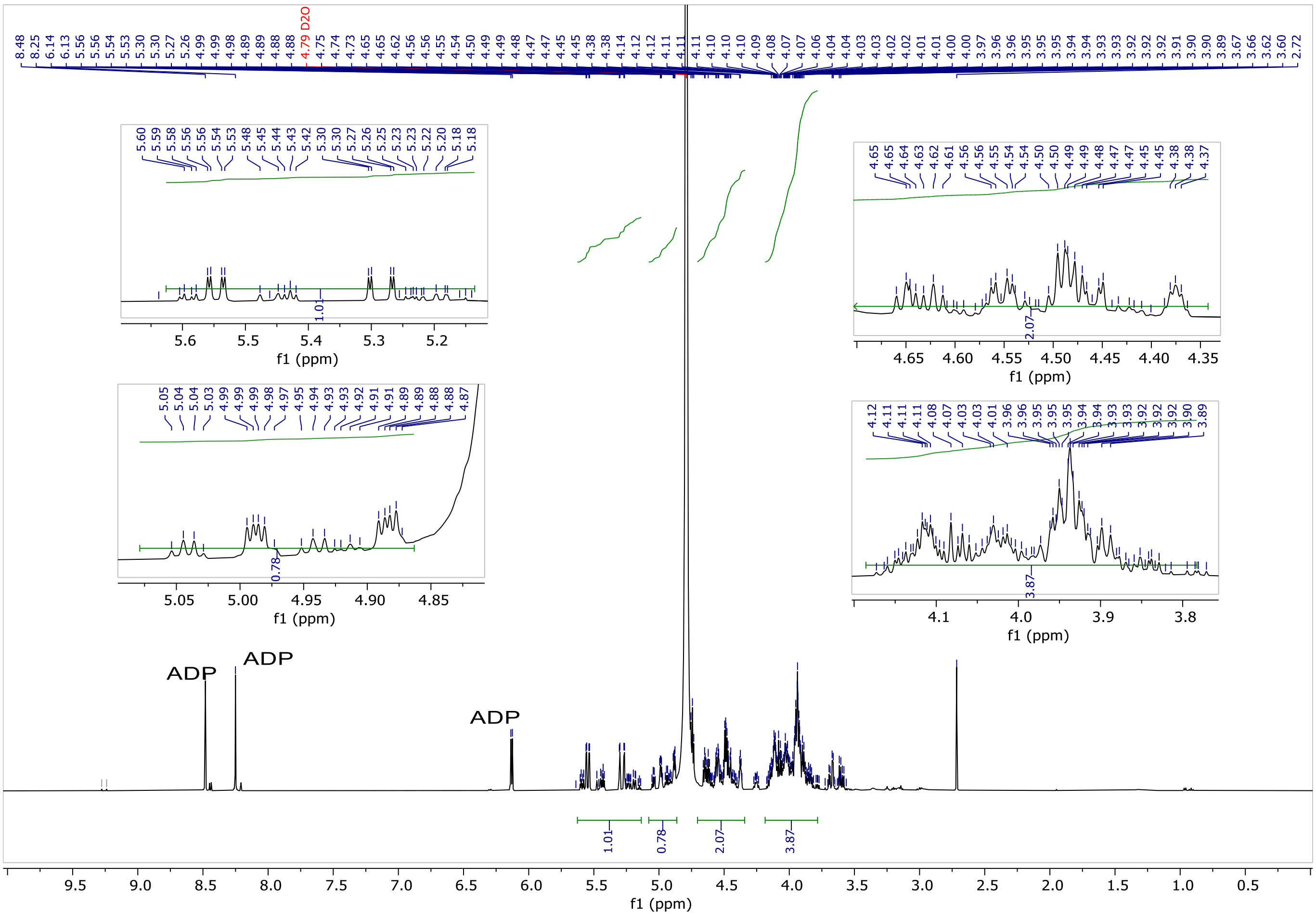

*Figure 67: ^{1}H NMR (500 MHz) of 2-deoxy-2-fluoro-L-lyxose-5-phosphate **19a***

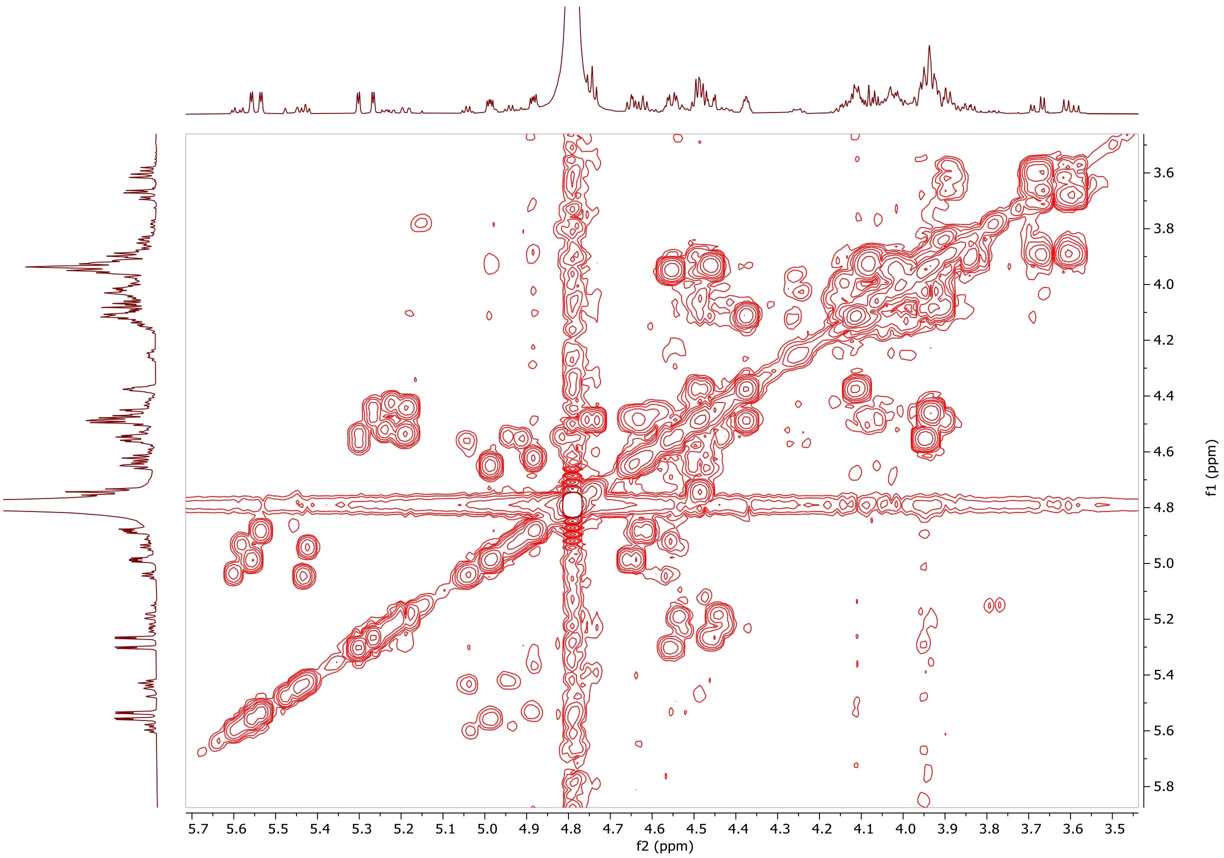

Figure 68: ^{1}H-^{1}H NMR (500MHz) of 2-deoxy-2-fluoro-L-lyxose-5-phosphate ***19a***

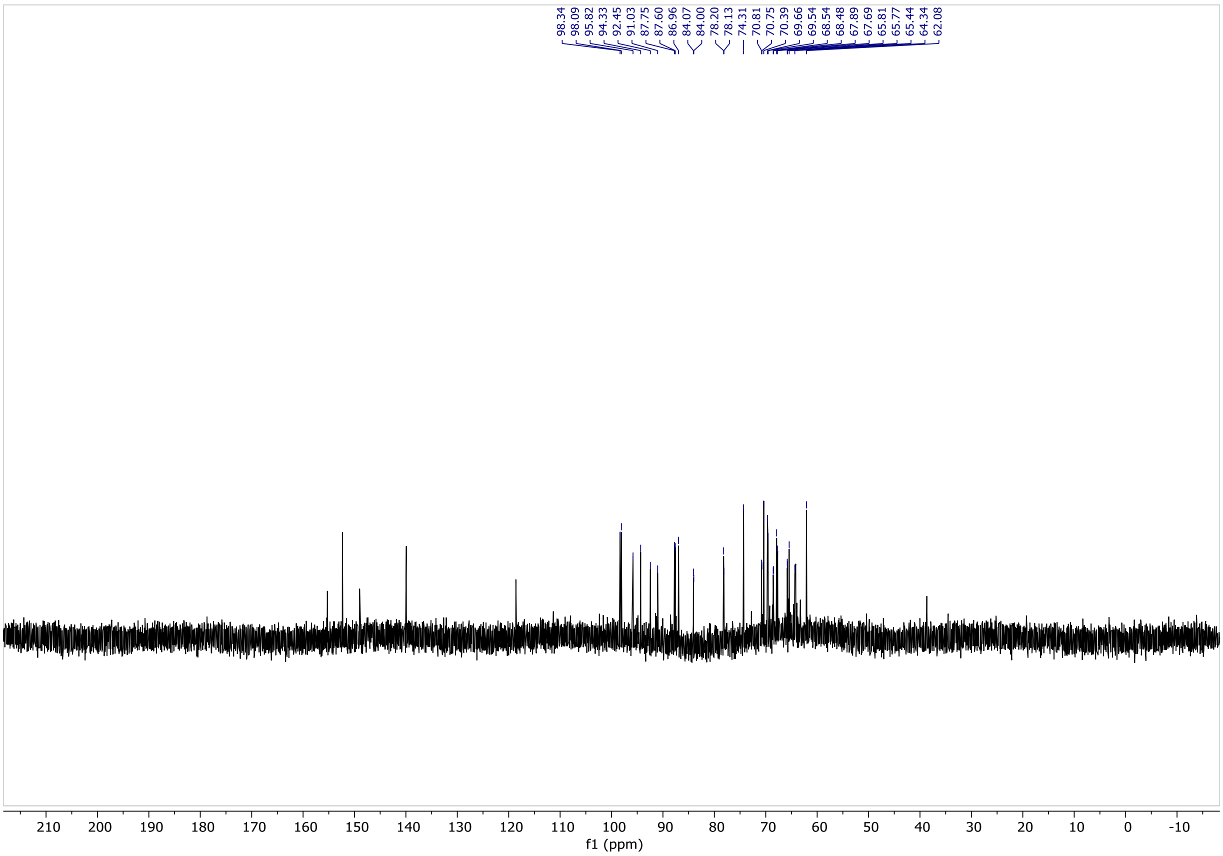

Figure 69: ^{13}C NMR (500 MHz) of 2-deoxy-2-fluoro-L-lyxose-5-Phosphate ***19a***

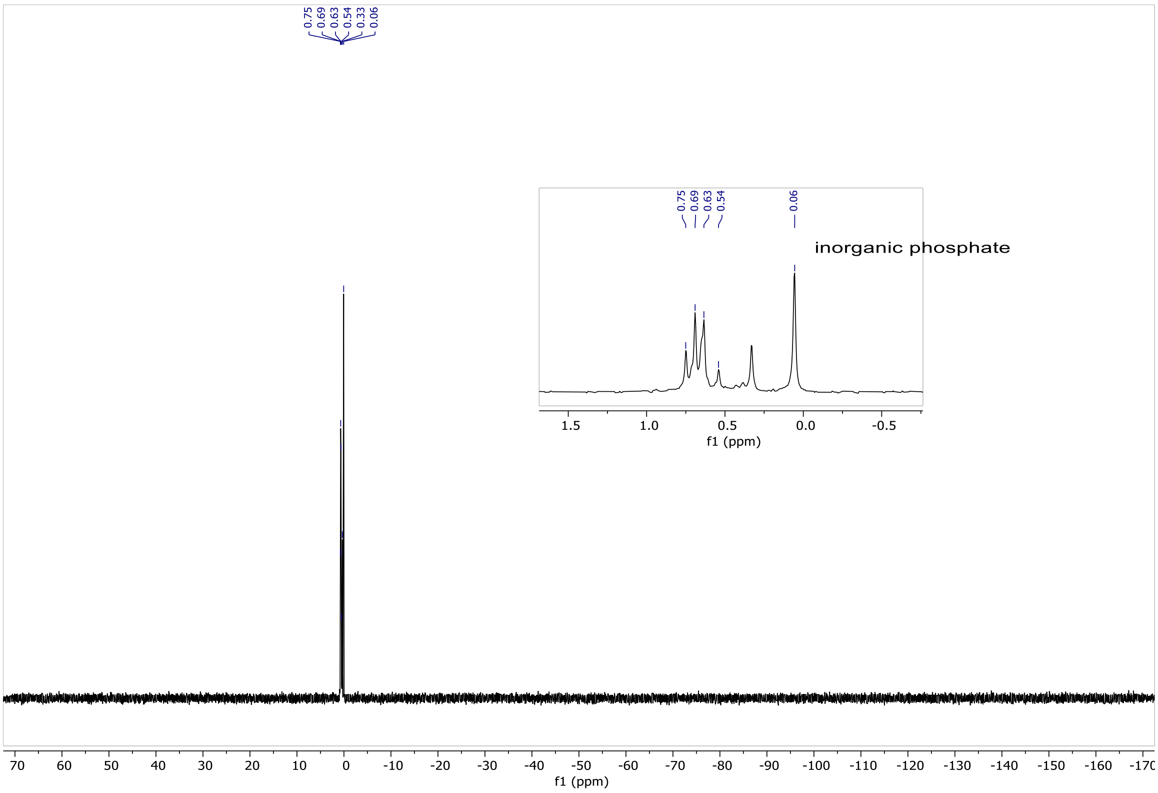

*Figure 70: ^{31}P NMR (400 MHz) of 2-deoxy-2-fluoro-L-lyxose-5-Phosphate **19a***

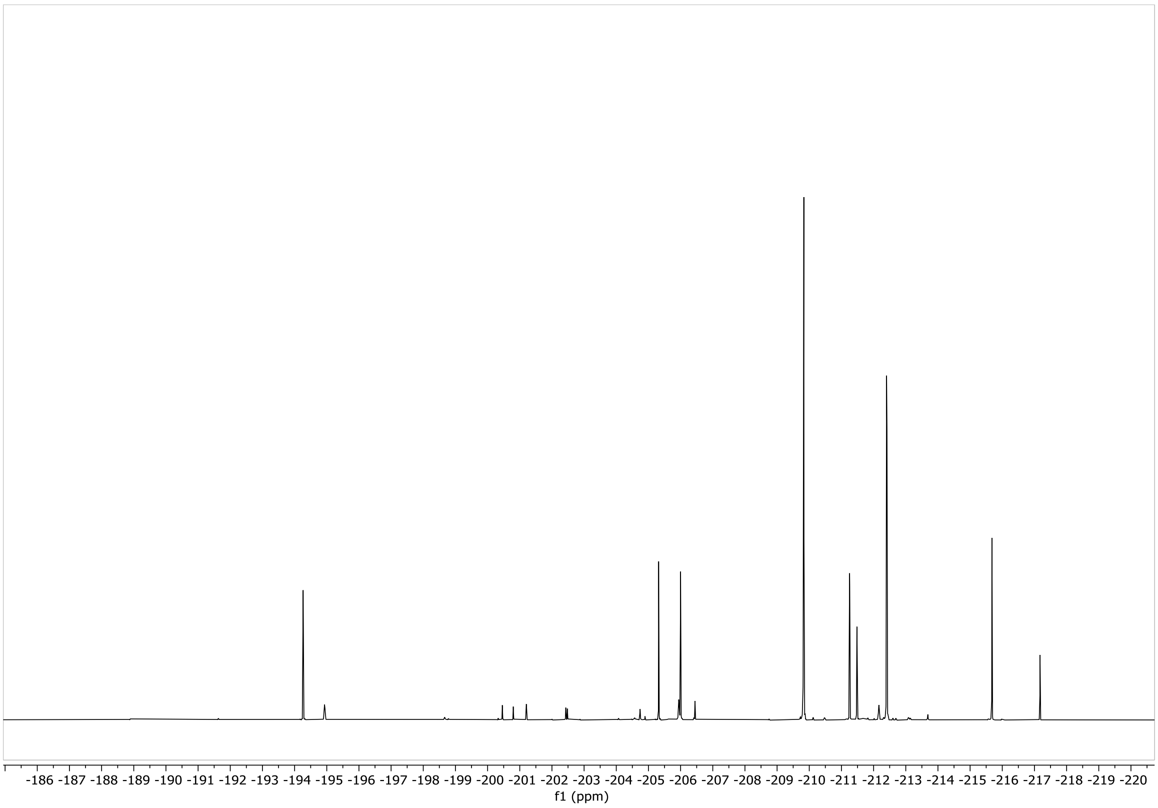

*Figure 71: ^{19}F NMR (500 MHz) of 2-deoxy-2-fluoro-L-lyxose-5-Phosphate **19a***

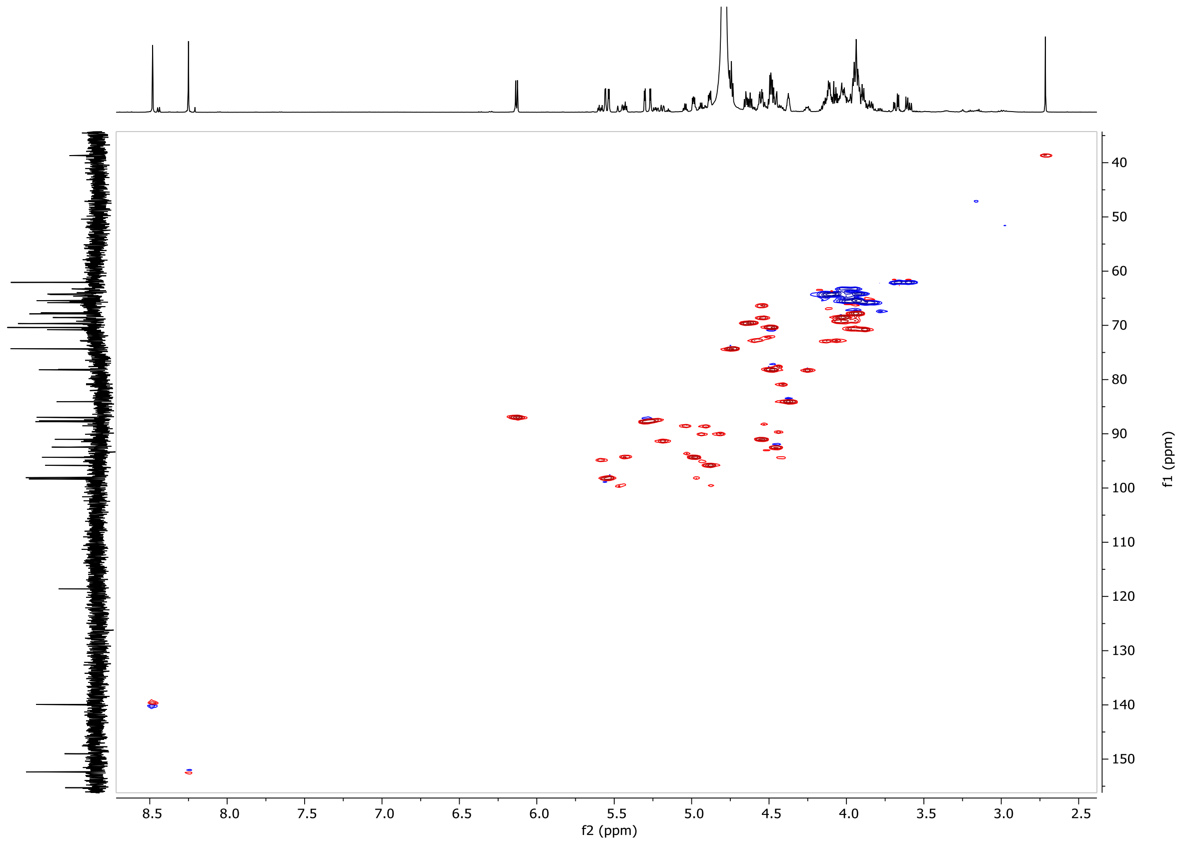

Figure 72: ^{13}C-^{1}H HSQC NMR (500 MHz) of 2-deoxy-2-fluoro-L-lyxose-5-phosphate ***19a***

11.3 Semi-preparative Scale Syntheses of D-Ribose-5-Phosphate Analogues

11.3.1 General Procedure for the Synthesis of D-Ribose-5-Phosphate Analogues

To a 15 ml falcon tube, D-glyceraldehyde **16** (20mM), aldehyde donor **2** (80 mM), ATP (5 mol%), PEP (40 mM) and $MgCl_2$ (20 mM) were added. HEPES buffer (pH 8.2), and milliQ water were added, so as to give a final buffer concentration of 50 mM and a final reaction volume of 5 ml. The reaction was initiated by addition of $DERA_{F76A}$ (4 mg/ml), dihydroxyacetone kinase (0.1 mg/ml), and pyruvate kinase (10 U/ml). The reaction mixture was left shaking in an orbital incubator at 30 °C 200 RPM for 18 hours. After this time the enzyme was removed using a 10k MWCO filter viva spin. The phosphorylated products **11b** and **12b** were then purified using anion exchange chromatography as previously described.

11.3.2 2-deoxy-2-methoxy-D-ribose-5-phosphate (**11b**)

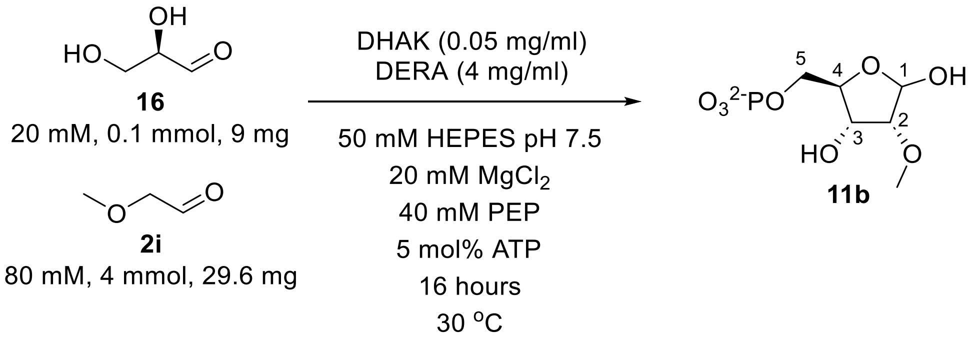

Title compound **11b** was prepared according to the general procedure using D-glyceraldehyde (20 mM, 0.1 mmol, 9 mg), methoxyacetaldehyde (80 mM, 0.4 mmol, 29.6 mg), PEP (40 mM), ATP (5 mol%) and $MgCl_2$ (20 mM). 12.8 mg of product was isolated as a white solid, giving 48% isolated yield.

Major Anomer:

^{1}H NMR (500 MHz) 3.48 (s, 1H, H6), 3.75 (dd, J = 5.0, 2.9 Hz, 1H, H2), 3.83 (m, 2H, H5), 4.04 (dd, J = 9.4, 4.75, 1H, H4), 4.38 (dd, J = 5.05, 5.05), 5.29 (d, *J* = 2.9 Hz, 1H, H1).

^{13}C NMR (125 MHz) 58.07 (C6), 64.27 (d, J = 4.7 Hz, C5), 69.72 (C3), 82.64 (d, J = 8 Hz, C4), 84.48 (C2), 99.00 (C1).

^{31}P NMR (400 MHz) 3.65 (s, P5, overlapping with minor)

Minor Anomer:

^{1}H NMR (500 MHz) 3.49 (s, 1H, H6), 3.88 (m, 1H, H2), 3.79 (m, 2H, H5), 4.22 (dd, J = 8.2, 4.15), 4.29 (dd, J = 5.6, 3.6, 1H, H3), 5.44 (d, *J* = 4.4 Hz, 1H, H1).

^{13}C NMR (125 MHz) 58.38 (C6), 63.65 (d, J = 3.8 Hz, C5), 69.13 (C3), 79.67 (C2), 83.35 (d, J = 8 Hz, C4), 95.36 (C1).

^{31}P NMR (400 MHz) 3.65 (s, P5, overlapping with major)

Known impurities have been annotated on the NMR spectra. Peaks not annotated correspond to unknown impurities.

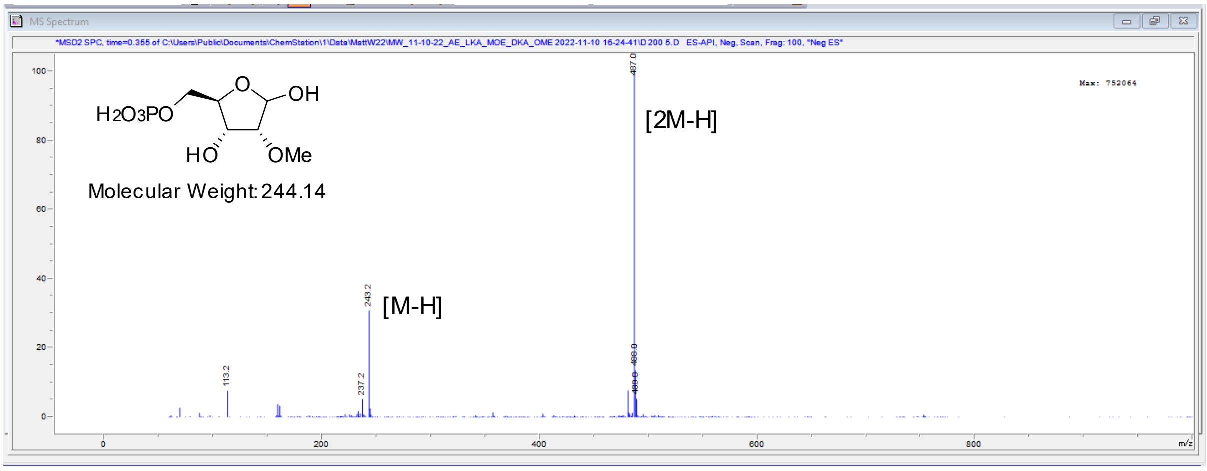

*Figure 73: ESI- mass spectrum of 2-deoxy-2-methoxy-D-ribose-5-phosphate (**11b**) [M-H] = 243.2*

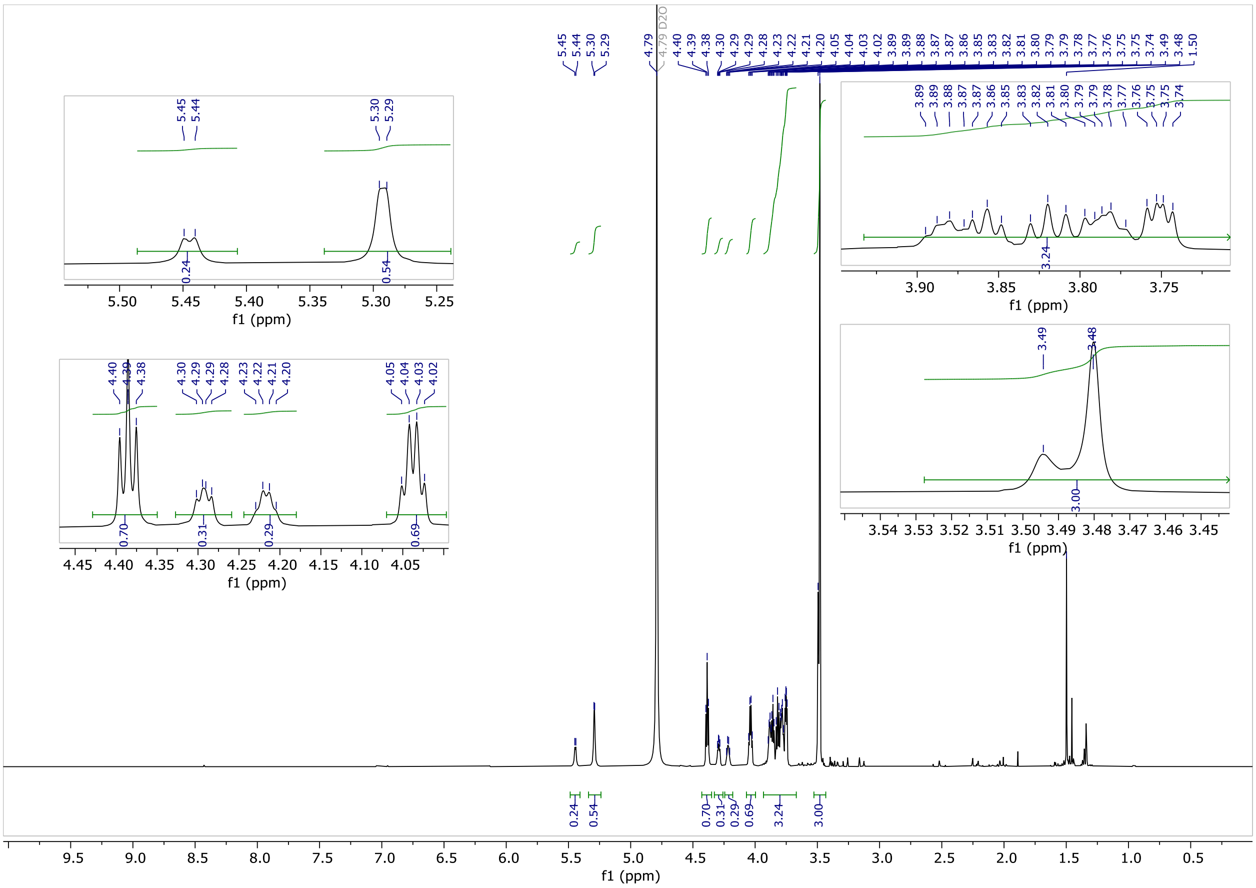

*Figure 74: ^{1}H NMR (500MHz) of 2-deoxy-2-methoxy-D-ribose-5-phosphate **11b***

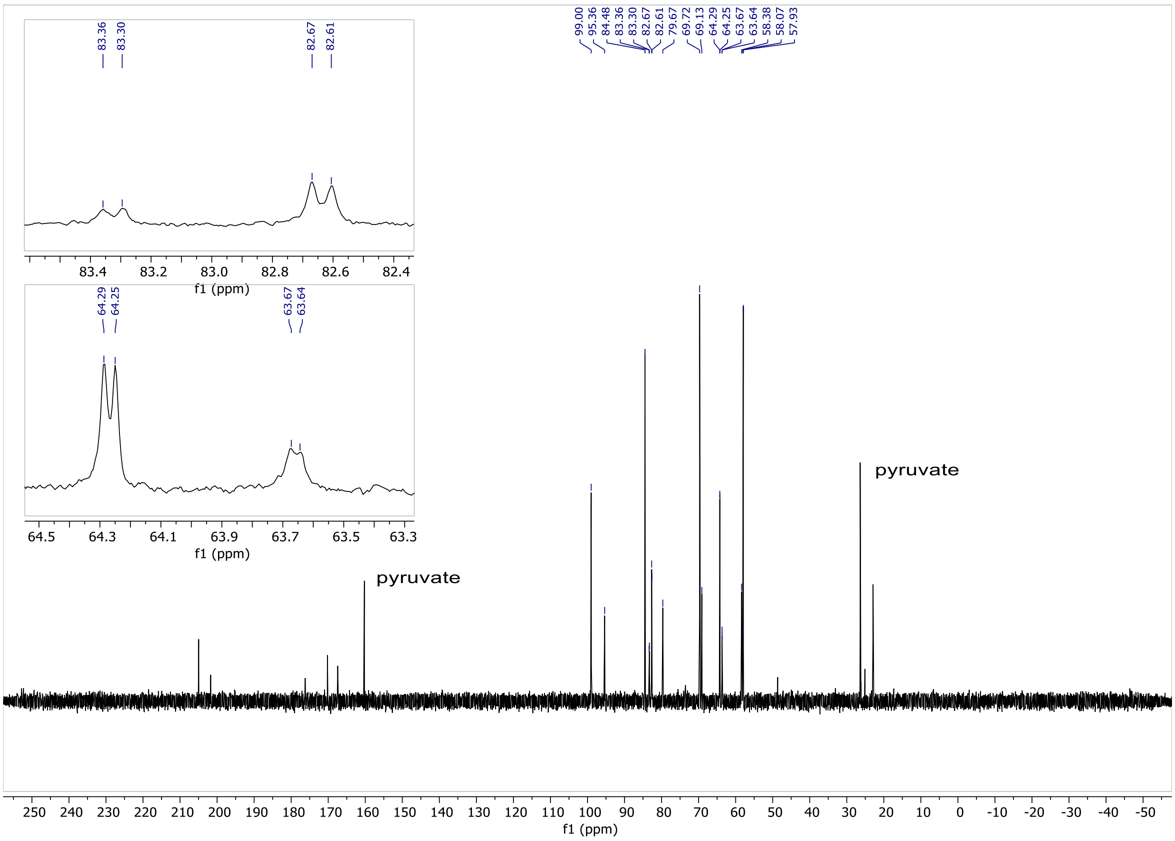

Figure 75: *^{13}C NMR (500 MHz) of 2-deoxy-2-methoxy-D-ribose-5-phosphate* ***11b***

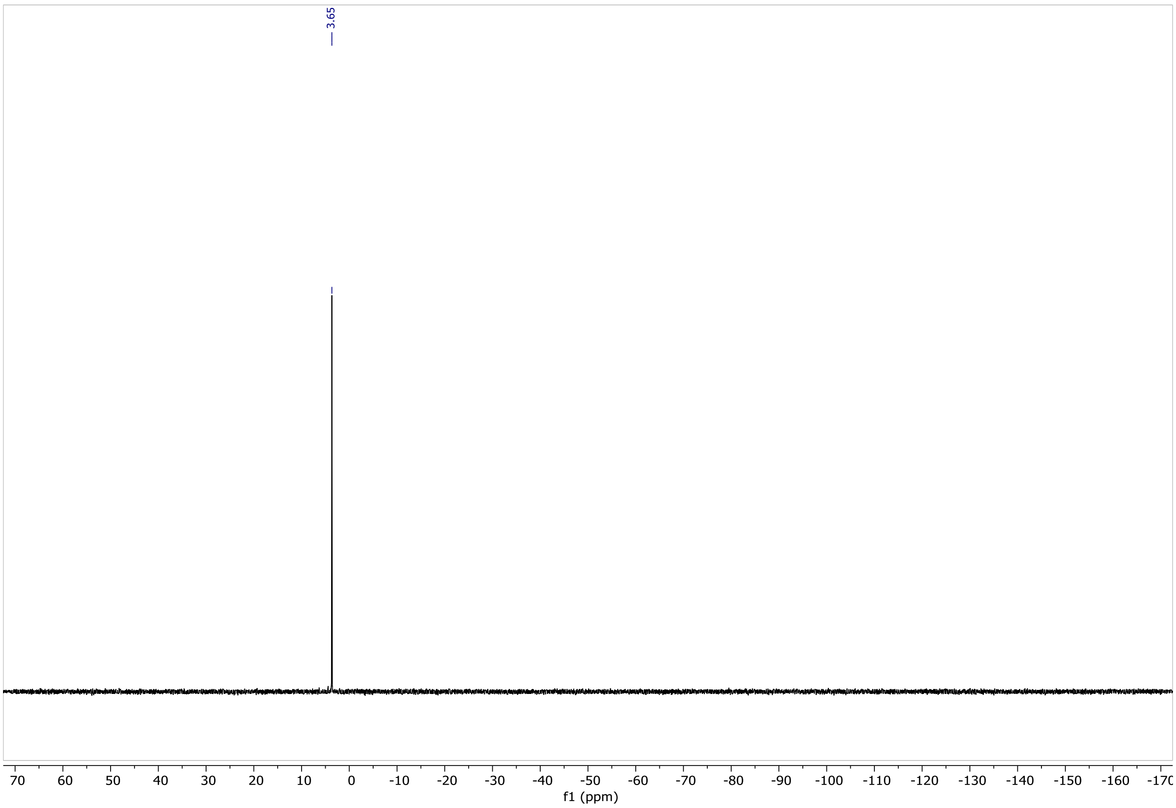

Figure 76: *^{31}P NMR (400 MHz) of 2-deoxy-2-methoxy-D-ribose-5-phosphate* ***11b***

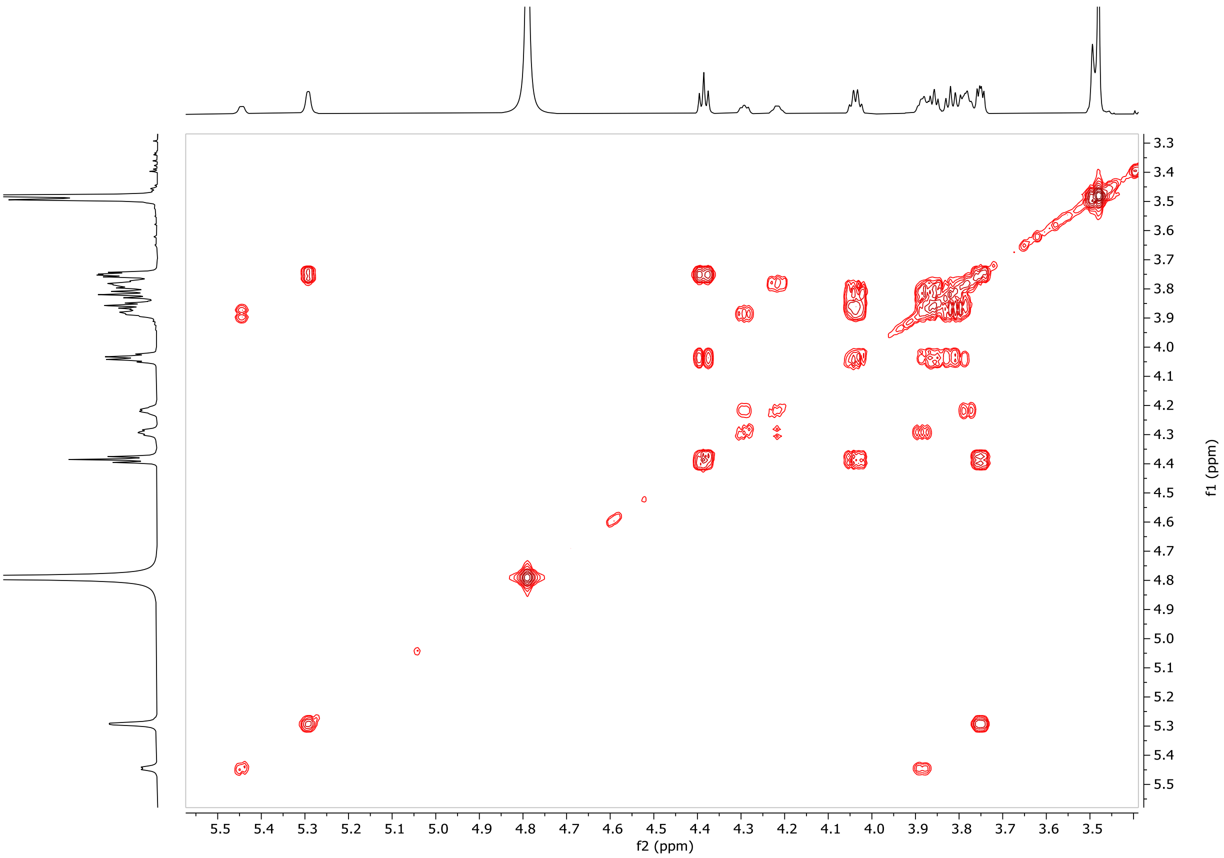

*Figure 77: ^{1}H-^{1}H COSY NMR (500 MHz) of 2-deoxy-2-methoxy-D-ribose-5-phosphate **11b***

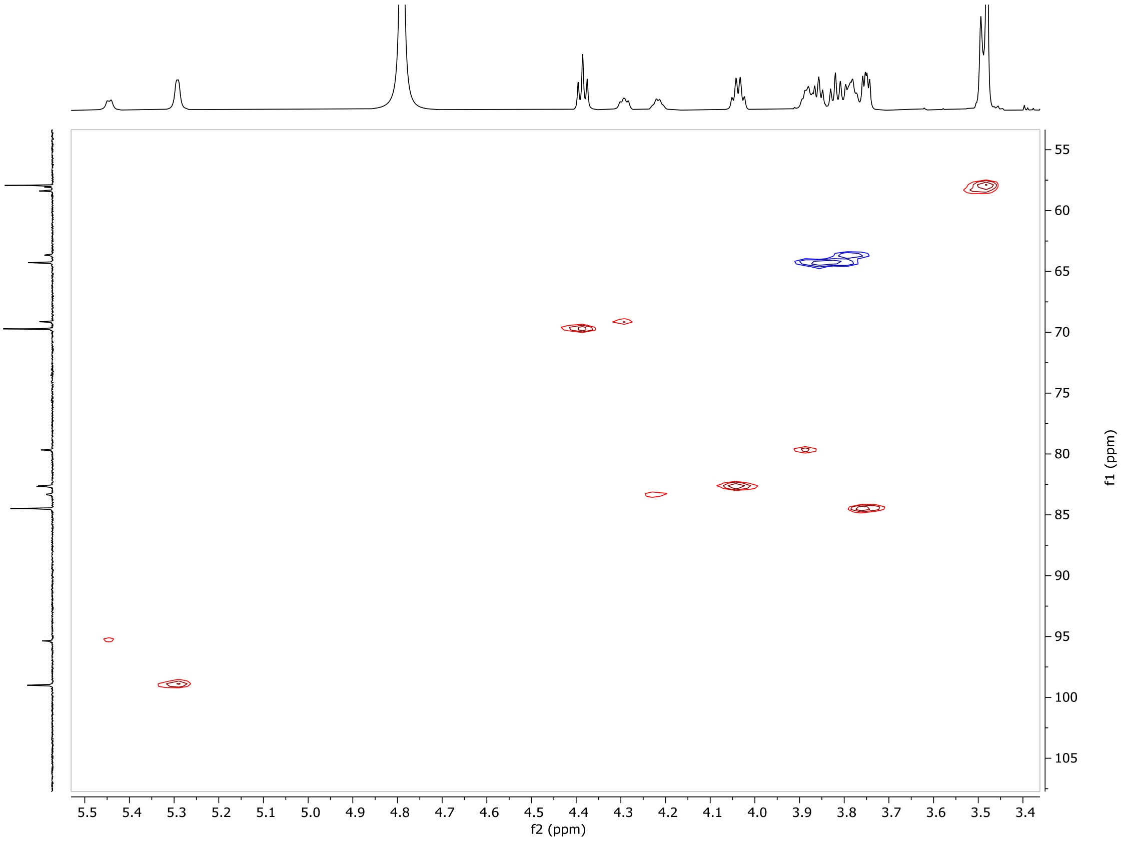

*Figure 78: ^{13}C-^{1}H HQSC NMR (500 MHz) of 2-deoxy-2-methoxy-D-ribose-5-phosphate **11b***

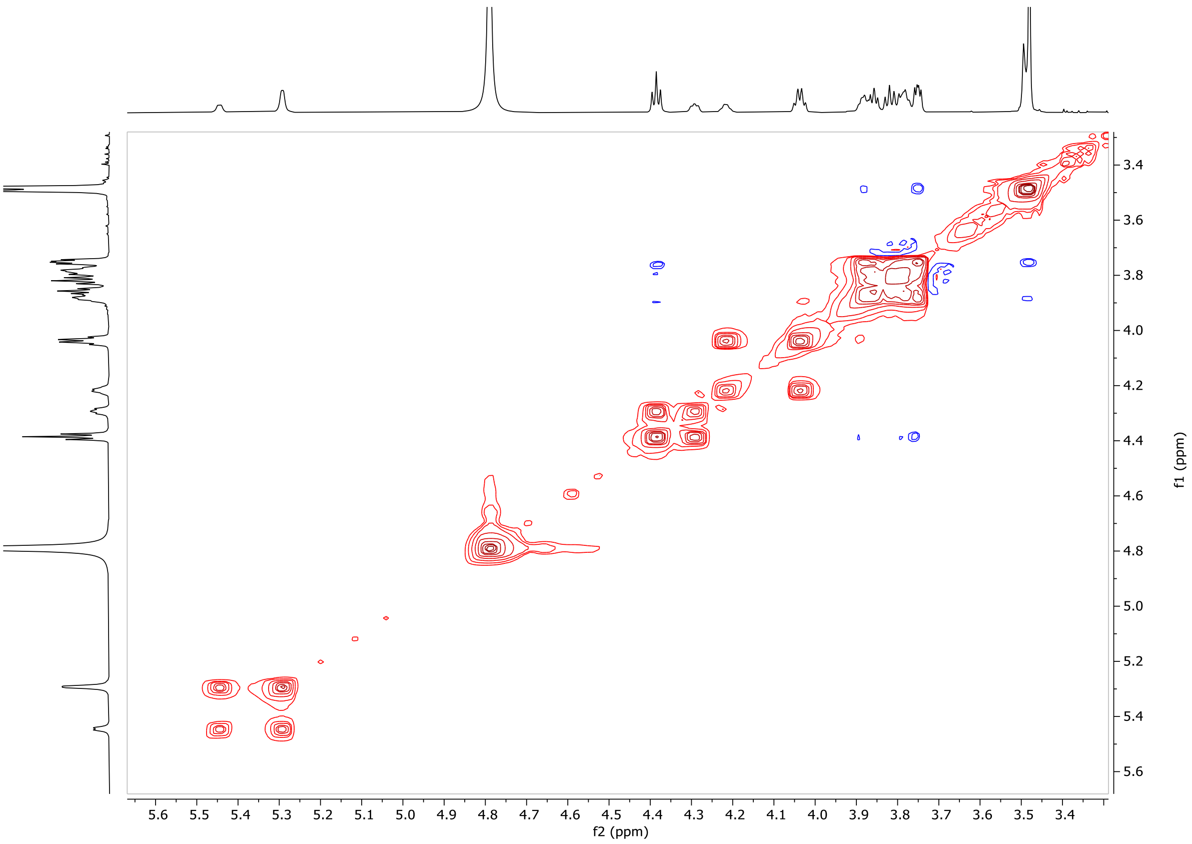

*Figure 79: ^{1}H-^{1}H NOESY NMR (500 MHz) of 2-deoxy-2-methoxy-D-ribose-5-phosphate **11b***

11.3.3 2-deoxy-2-methoxy(ethoxy)-D-ribose-5-phosphate (12b)

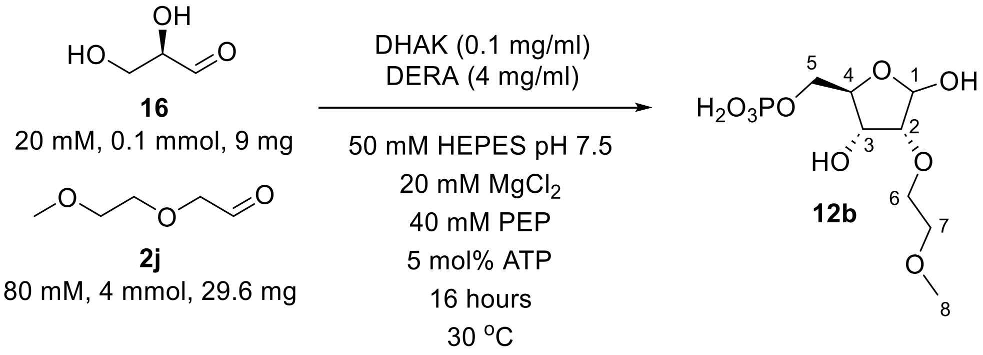

Title compound 12 was prepared according to the general procedure using D-glyceraldehyde (20 mM, 0.1 mmol, 9 mg), methoxy(ethoxy)acetaldehyde (80 mM, 0.4 mmol, 47.2 mg) PEP (40 mM), ATP (5 mol%) and $MgCl_2$ (20 mM). 15.2 mg of product was isolated as a white solid, corresponding to a 47% isolated yield.

Major Anomer:

^{1}H NMR (500 MHz) 3.39 (s, 3H, H8, overlapping with minor anomer), 3.66 (m, 2H, H7, overlapping with minor anomer), 3.83 (m, 2H, H6 overlapping with minor anomer), 3.85 (m, 2H , H5, overlapping with H6, H2), 3.88 (m, 1H H2, overlapping with H5), 4.07 (dd, J = 9.2, 4.7 Hz, 1H, H4), 4.38 (dd, J = 5.1, 5.1 Hz, 1H, H3), 5.30 (d, J = 3.5 Hz, 1H, H1).

^{13}C NMR (125 MHz) 58.01 (C8, overlapping with minor anomer), 64.22 (d, J = 4.8 Hz, C5), 69.37 (C6), 70.06 (C3), 71.17 (C7), 82.65 (C4), 83. 44 (C2), 99.45 (C1).

^{31}P NMR (400 MHz) 3.74 (s, P5, overlapping with minor)

Minor Anomer:

^{1}H NMR (500 MHz) 3.40 (s, 3H, H8, overlapping with major anomer) 3.66 (m, 2H, H7 overlapping with major anomer) 3.79 (m,2H, H5, overlapping with major anomer) 3.83 (m, 2H, H6 overlapping with major anomer) 4.02 (dd, J = 5.0, 5.0 Hz, 1H, H2) 4.24 (dd, J = 8.3, 4.2 Hz, 1H, H3) 5.45 (d, J = 4.3 Hz, 1H, H1)

^{13}C NMR (125 MHz) 58.01 (C8, overlapping with major anomer) 63.64 (C5) 69.49 (C6) 69.94 (C3) 71.17 (C7) 78.62 (C2) 83.25 (d, J = 8 Hz, C4) 95.53 (C1)

^{31}P NMR (400 MHz) 3.74 (s, P5, overlapping with major)

Known impurities have been annotated on the NMR spectra, peaks not annotated correspond to unknown impurities.

Mass Spec

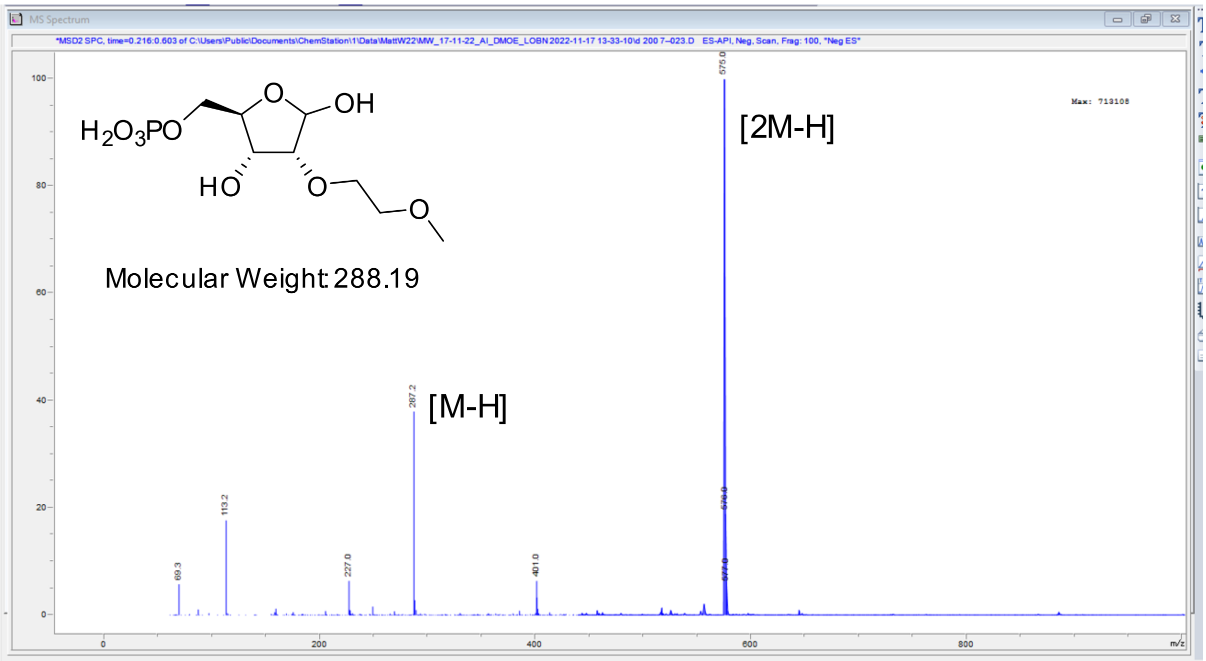

*Figure 80: ESI- mass spectrum of 2-deoxy-2-methoxy(ethoxy)-D-ribose-5-phosphate (**12b**), [M-H] = 287.2*

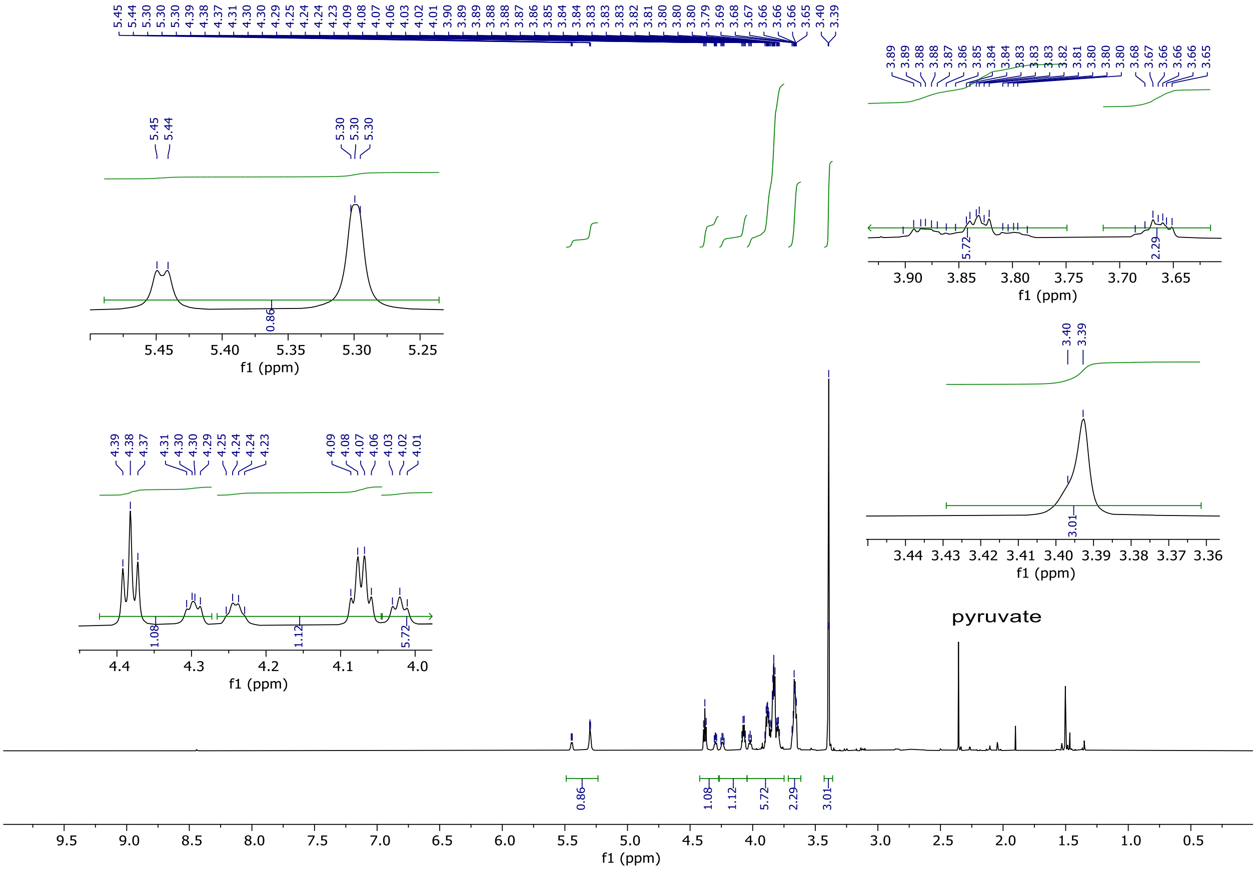

*Figure 81: ^{1}H NMR (500 MHz) of 2-deoxy-2-methoxy(ethoxy)-D-ribose-5-phosphate **12b***

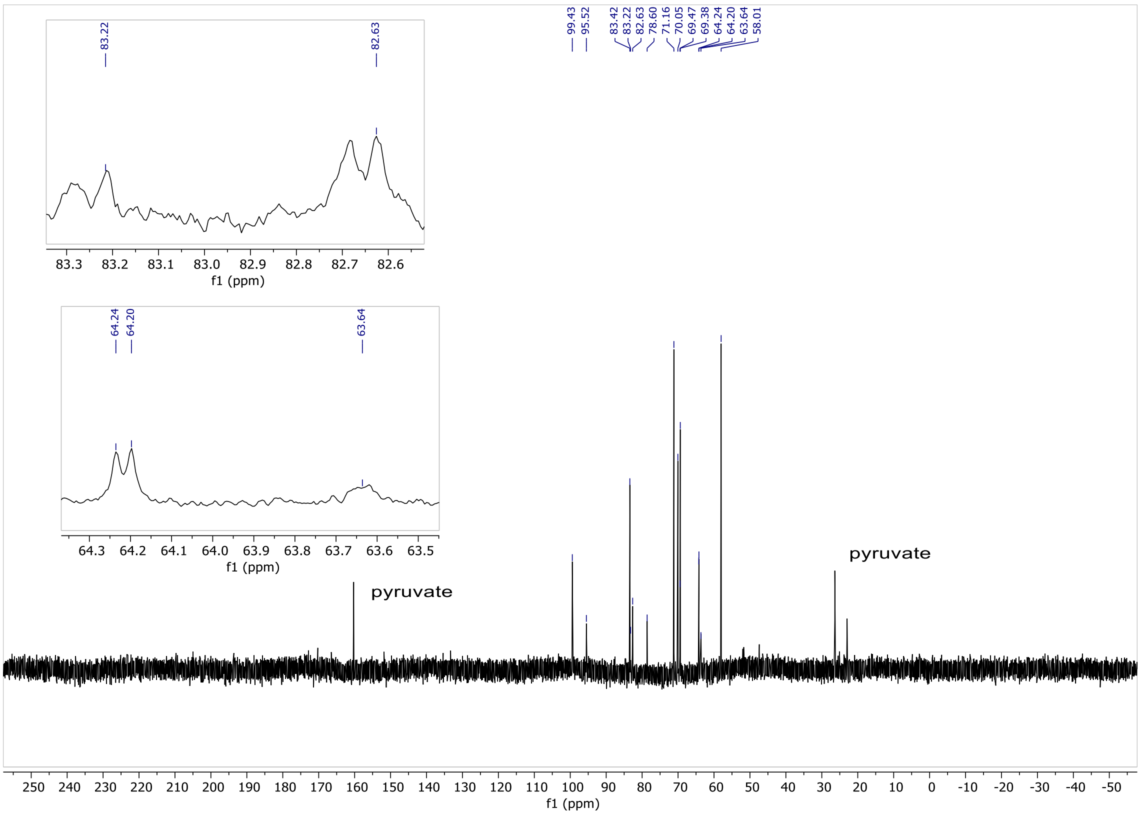

Figure 82:^{13}*C NMR (500 MHz) of 2-deoxy-2-methoxy(ethoxy)-D-ribose-5-phosphate* ***12b***

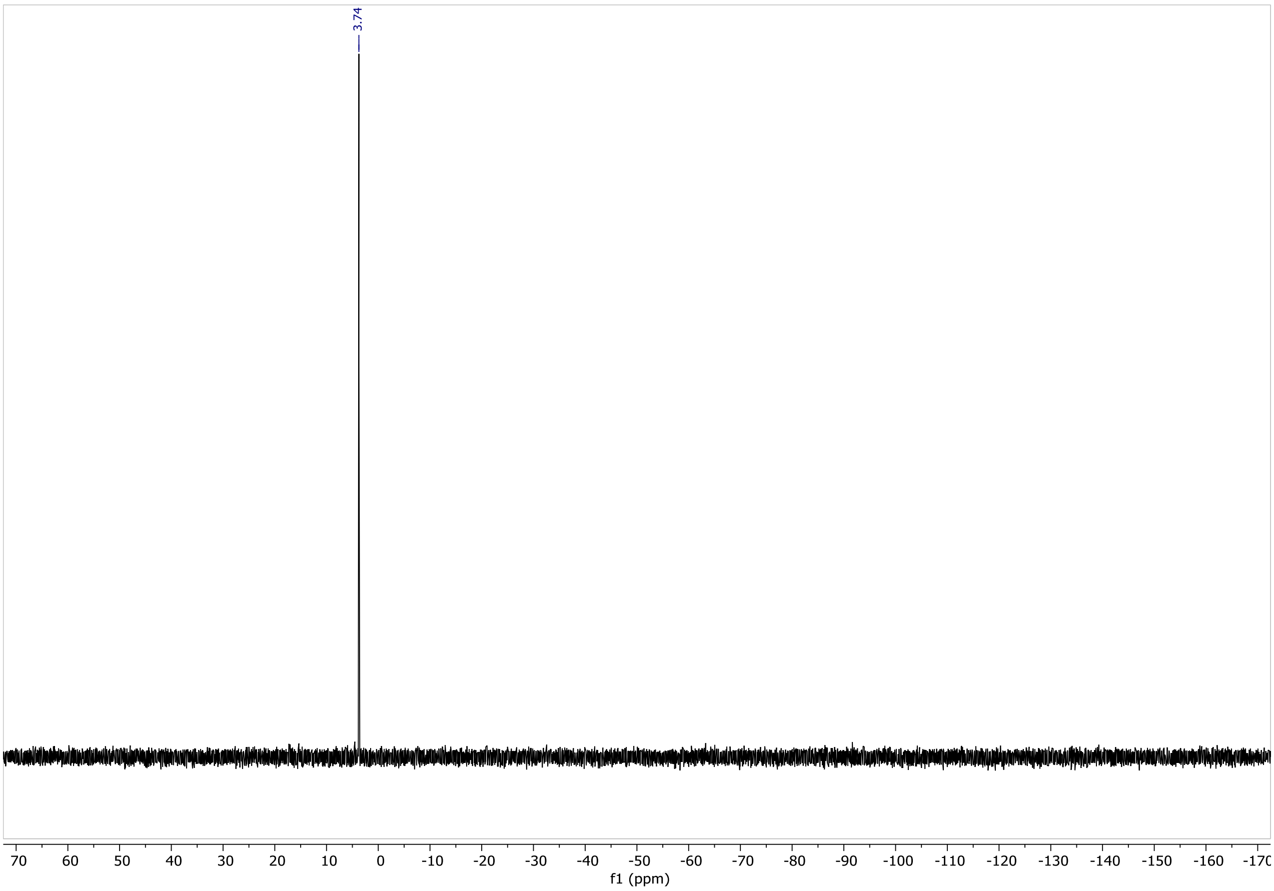

Figure 83: ^{31}*P NMR (400 MHz) of 2-deoxy-2-methoxy(ethoxy)-D-ribose-5-phosphate* ***12b***

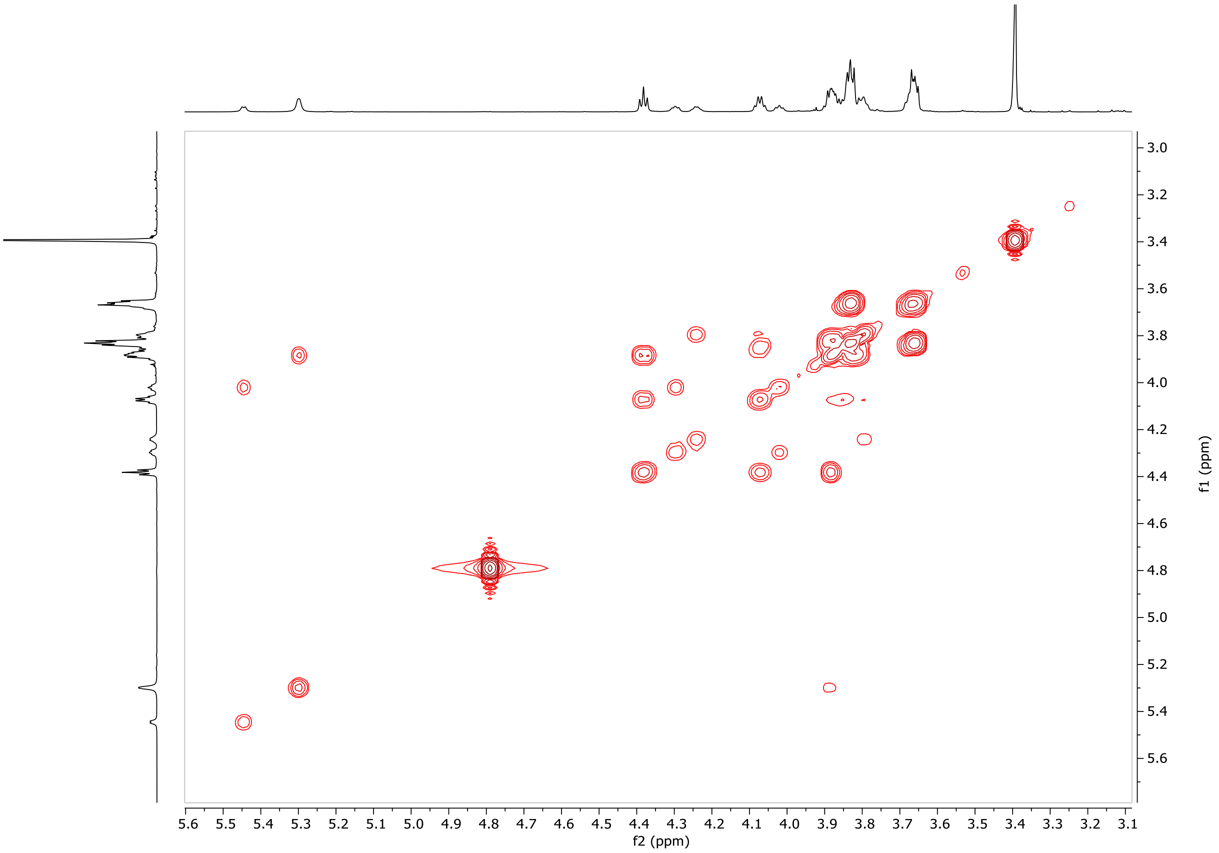

Figure 84: ^{1}H-^{1}H COSY NMR (500 MHz) of 2-deoxy-2-methoxy(ethoxy)-D-ribose-5-phosphate ***12b***

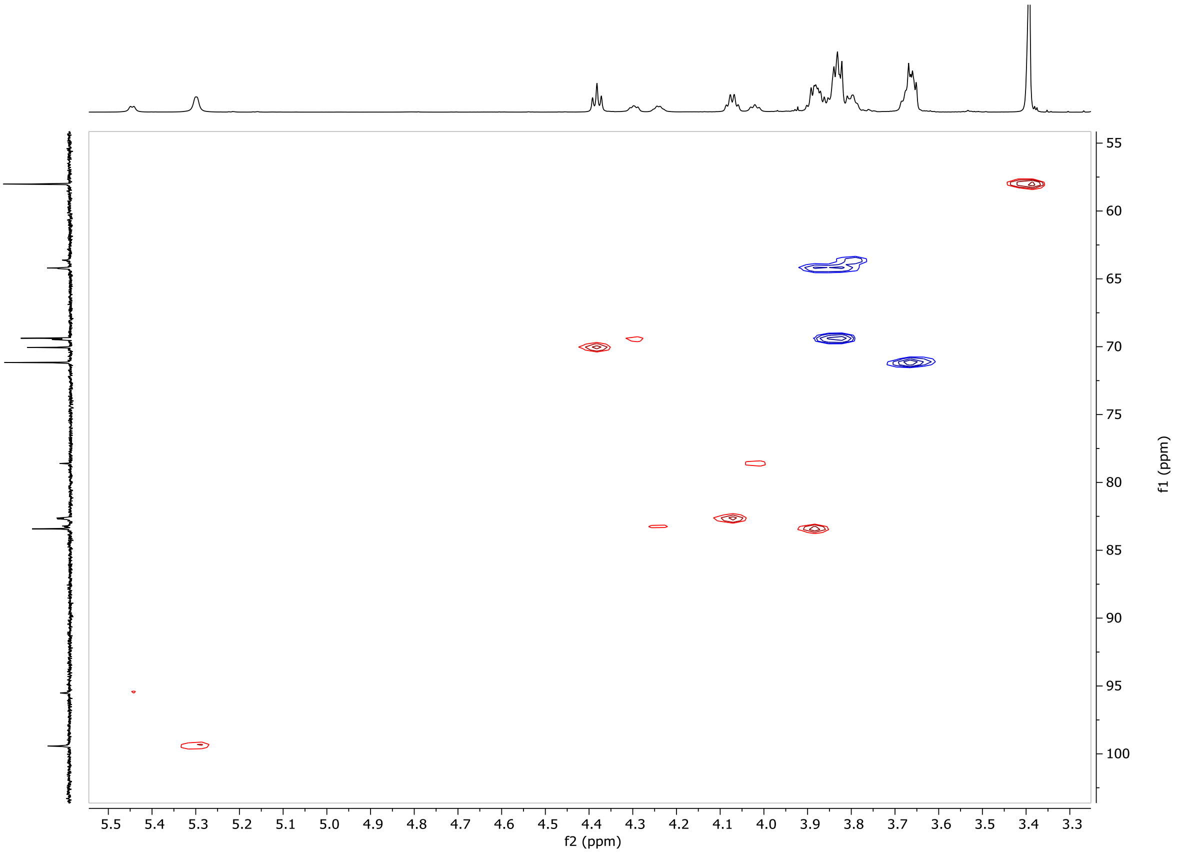

Figure 85: ^{13}C-^{1}H HSQC NMR (500 MHz) of 2-deoxy-2-methoxy(ethoxy)-D-ribose-5-phosphate ***12b***

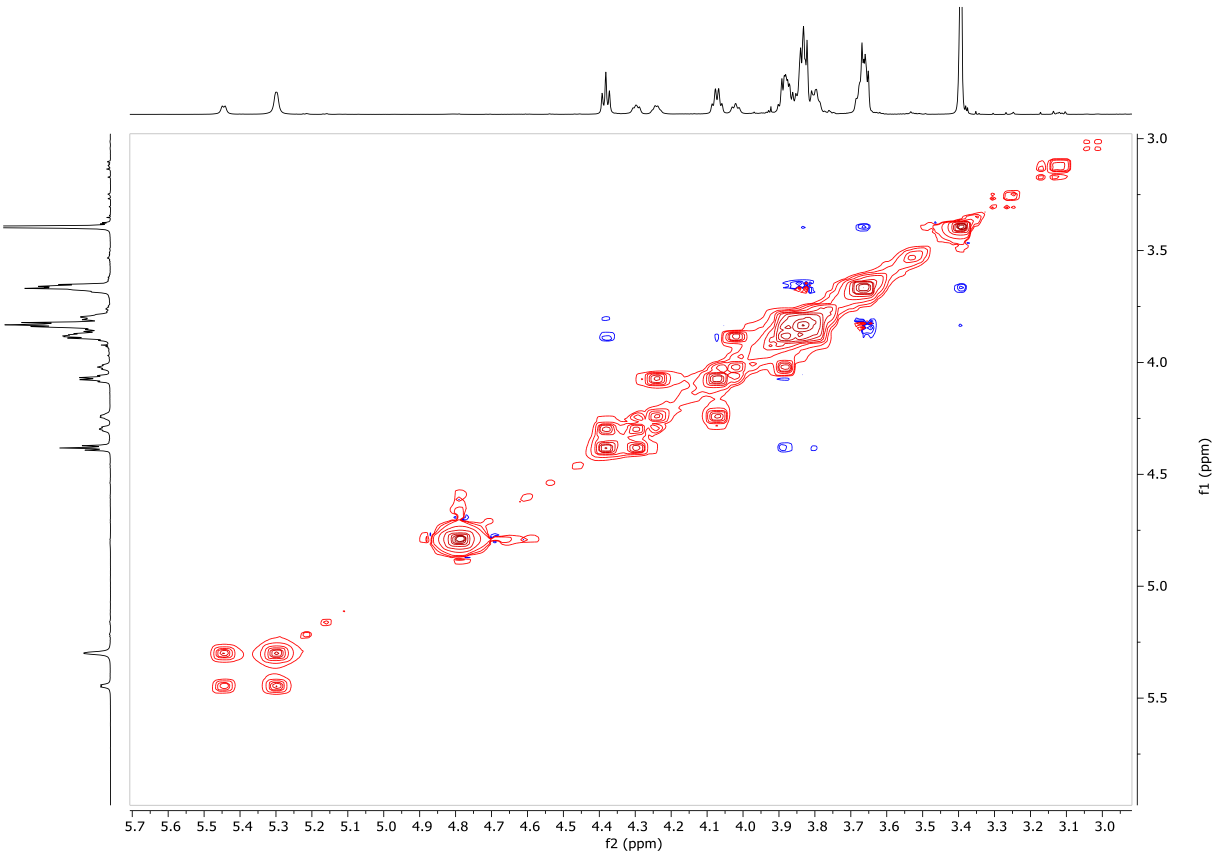

Figure 86: ^{1}H-^{1}H NOESY NMR (500 MHz) of 2-deoxy-2-methoxy(ethoxy)-D-ribose-5-phosphate ***12b***

11.3.4 2-deoxy-2-fluoro-D-ribose-5-phosphate (19b)

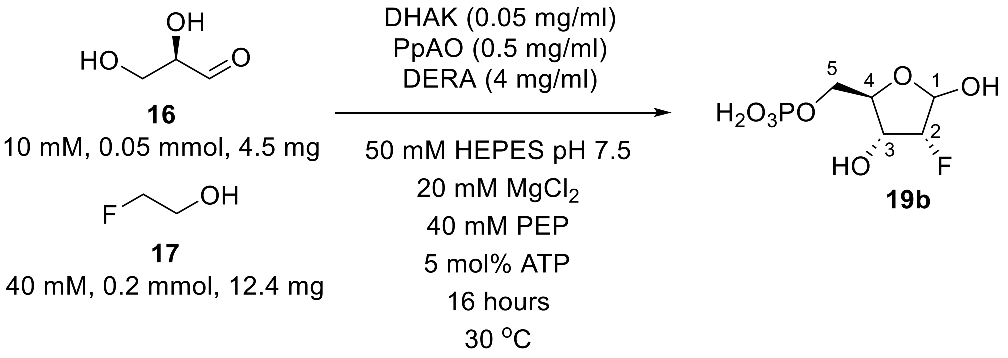

To a 15 ml falcon tube, D-glyceraldehyde **16** (10 mM, 0.05 mmol, 4.5 mg), fluoroethanol **17** (40 mM, 0.2 mmol, 12.4 mg), PEP (20 mM), ATP (5 mol%), and $MgCl_2$ (10 mM) were added. HEPES buffer (pH 8.2), and milliQ water were added, so as to give a final buffer concentration of 50 mM and a final reaction volume of 5 ml. The reaction was initiated by addition of $DERA_{WT}$ (4 mg/ml), *Cf*DHAK (0.1 mg/), PK (10 U/ml), and *Pp*AO (0.5 mg/ml). The reaction mixture was split into 10 x 500 µL reactions, these fractions were incubated in a thermoshaker at 30 °C 750 RPM for 18 hours. After this time, the fractions were recombined, and the enzyme was removed using a 10k MWCO filter. The phosphorylated product was then purified using anion exchange chromatography as previously described giving 5.8 mgs of **19b** as a white solid.

(Due to the presence of multiple unknown contaminants in the NMR, isolated yield has not been determined)

As with previous fluorinated product, expected product mass is present. ^{19}F NMR shows presence of expected four diastereomers but again shows multiple unknown fluorine peaks.

Multiple overlapping product peaks alongside several unknown peaks has made the ^{1}H/^{13}C NMR difficult to assign. Tentative assignments have been made by comparison to the other D-ribose-5-phosphate analogues.

Four diastereomers appear to be present in ^{1}H NMR, these have been assigned as diastereomers A-D, the stereochemistry of these has not been assigned.

Diastereomer A

^{1}H NMR (800 MHz) 3.86-4.04 (m, all H5 peaks overlapping - unassigned), 4.22 (m, 1H, H4A overlapping with H4C), 4.33 (m, 1H, H3A, overlapping with H3C), 4.98 (m, 1H, H2), 5.53 (m, 1H, H1, overlapping with H1B).

^{13}C NMR (200 MHz) 95.43 (C1), 89.99 (C2), 69.14 (C3), 80.53 (C4, overlapping with all other isomers), 62.69 – 65.79 (all C5 diastereomers overlapping).

^{31}P NMR (400 MHz) 2.06 (P5)

Diastereomer B

^{1}H NMR (800 MHz) 3.86-4.04 (m, all H5 peaks overlapping - unassigned), 4.10 (m, 1H, H4, overlapping with H4D), 4.44 (m, 1H, H3), 4.82 (m, 1H, H2, overlapping with H2O peak), 5.53 (m, 1H, H1, overlapping with H1A).

^{13}C NMR (200 MHz) 99.05 (C1), 90.90 (C2), 69.41 (C3), 80.53 (C4, overlapping with all other isomers), 62.69 – 65.79 (all C5 diastereomers overlapping).

^{31}P NMR (400 MHz) 2.06 (P5)

Diastereomer C

^{1}H NMR (800 MHz) 3.86-4.04 (m, all H5 peaks overlapping - unassigned), 4.22 (m, 1H, H4 overlapping with H4A), 4.33 (m, 1H, H3, overlapping with H3A), 4.89 (m, 1H, H2), 5.44 (m, 1H, H1, overlapping with H1D).

^{13}C NMR (200 MHz) 98.60 (C1), 94.16 (C2), 69.22 (C3), 80.53 (C4, overlapping with all other isomers), 62.69 – 65.79 (all C5 diastereomers overlapping).

^{31}P NMR (400 MHz) 0.50 (P5)

Diastereomer D

^{1}H NMR (800 MHz) 3.86-4.04 (m, all H5 peaks overlapping - unassigned), 4.10 (m, 1H, H4, overlapping with H4B), 4.41 (m, 1H, H3), 4.92 (m, 1H, H2), 5.44 (m, 1H, H1, overlapping with H1A).

^{13}C NMR (200 MHz) 94.01 (C1), 95.07 (C2), 69.34 (C3), 80.53 (C4, overlapping with all other isomers), 62.69 – 65.79 (all C5 diastereomers overlapping).

^{31}P NMR (400 MHz) 0.50 (P5)

Peaks not annotated correspond to unknown impurities.

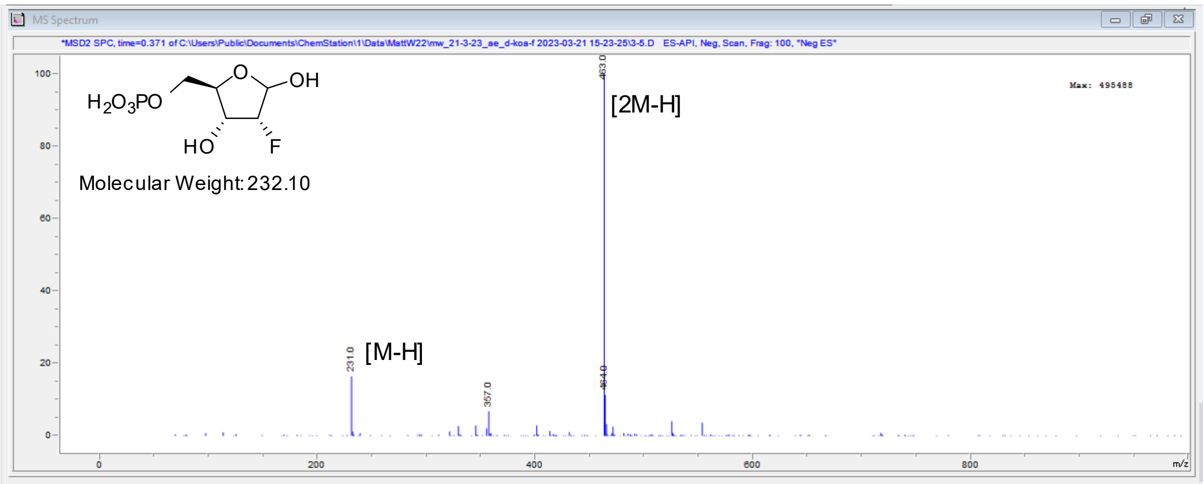

*Figure 87: ESI- mass spectrum of 2-deoxy-2-fluoro-D-ribose-5-phosphate (**19b**), {M-H] = 231.0.*

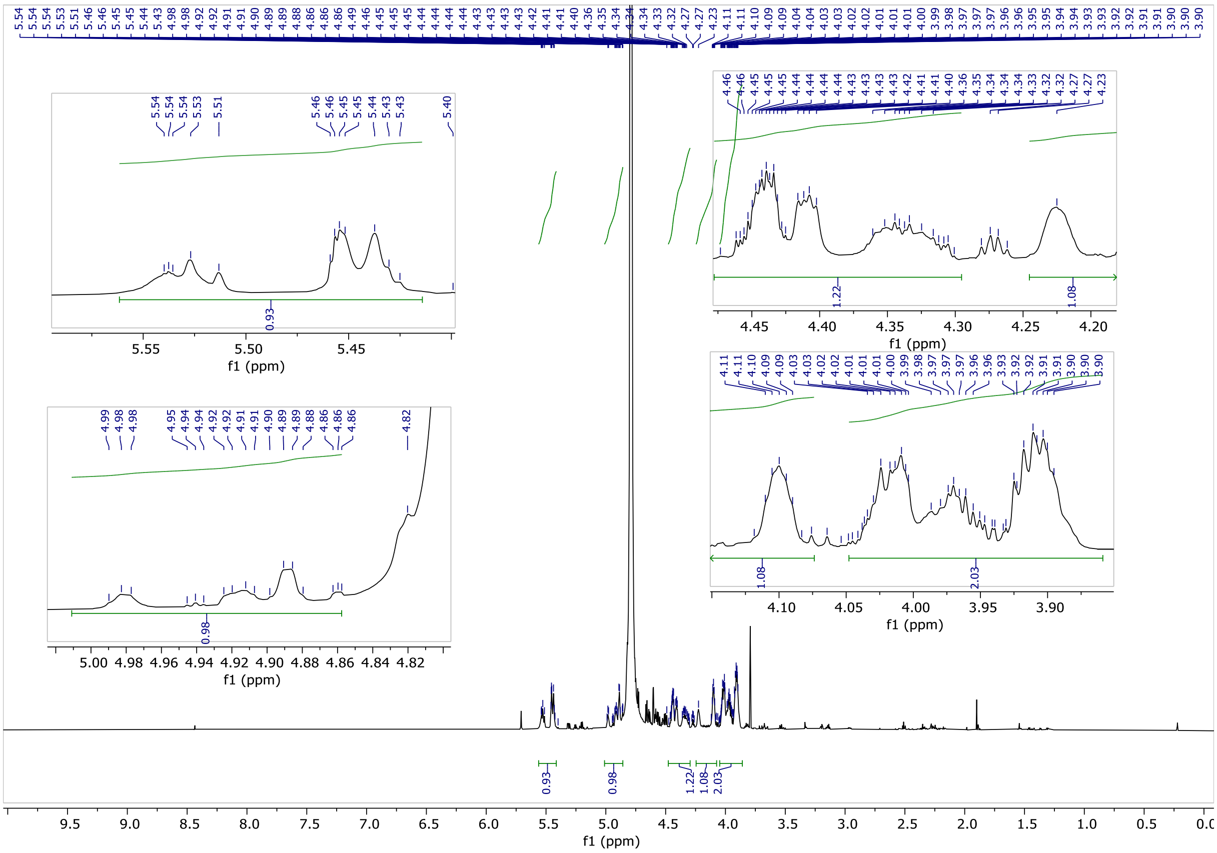

*Figure 88: ^{1}H NMR (500 MHz) of 2-deoxy-2-fluoro-D-ribose-5-phosphate **19b***

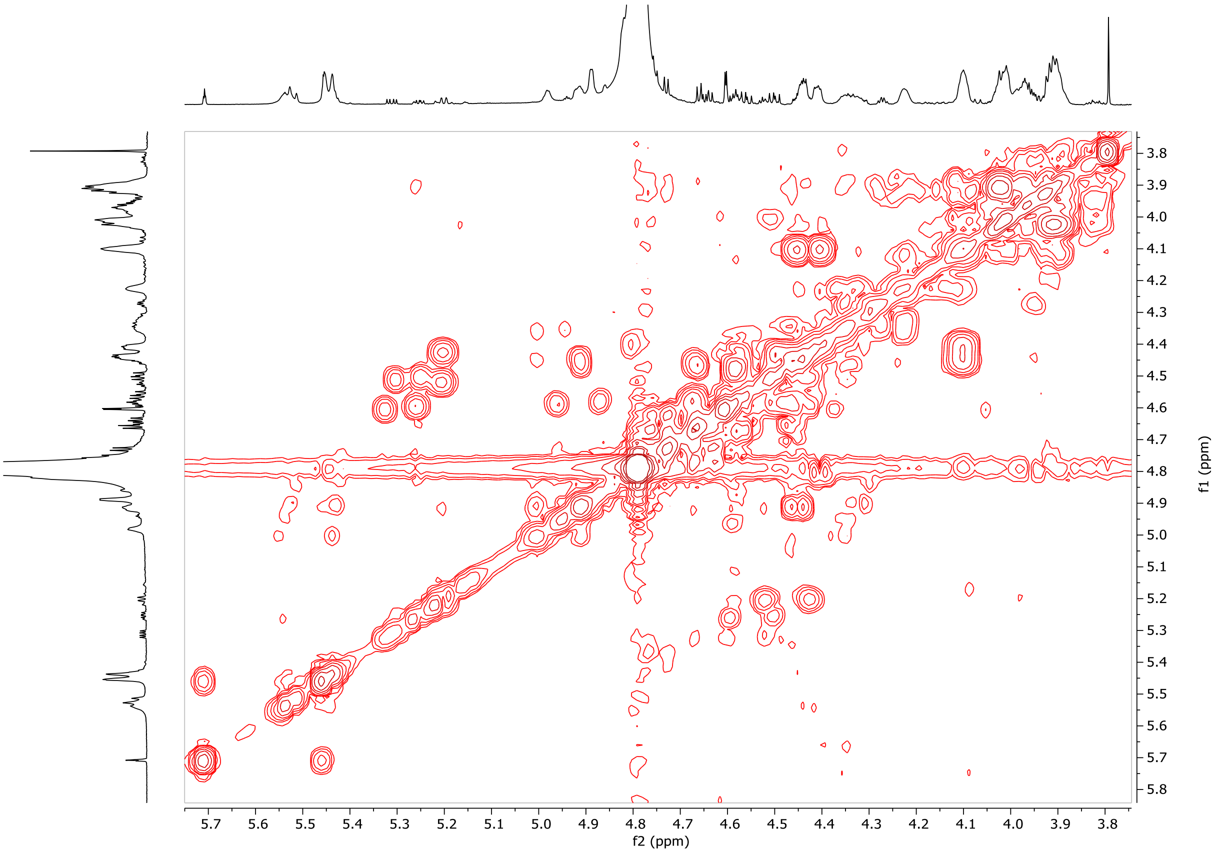

*Figure 89: ^{1}H-^{1}H COSY NMR (500 MHz) of 2-deoxy-2-fluoro-D-ribose-5-phosphate **19b***

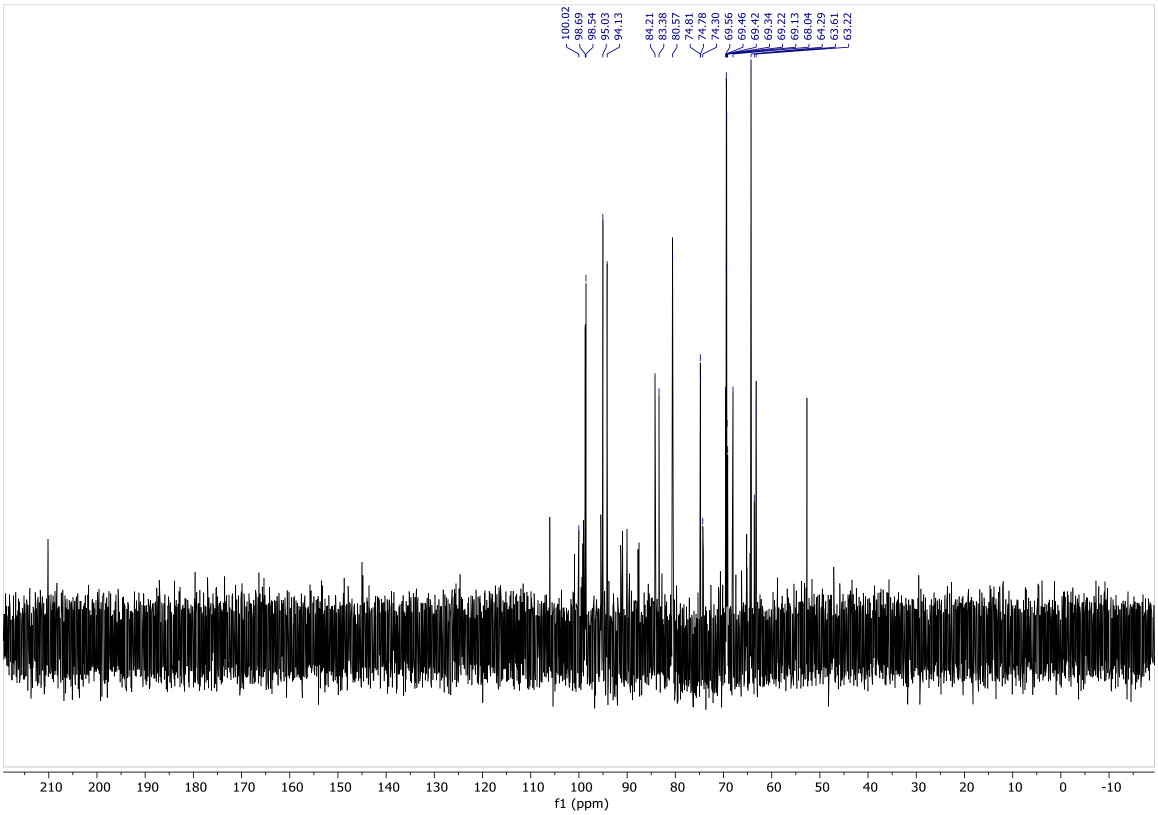

Figure 90: ^{13}C NMR (500 MHz) of 2-deoxy-2-fluoro-D-ribose-5-phosphate

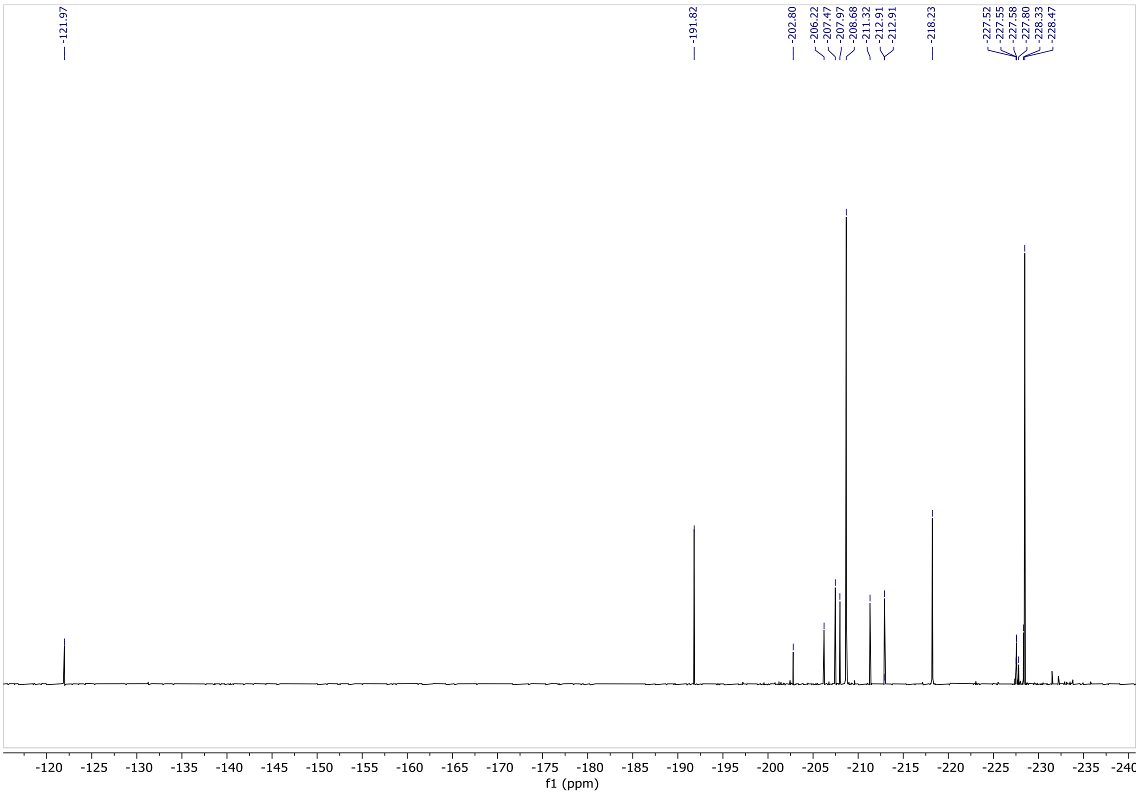

*Figure 91: ^{19}F NMR (500 MHz) of 2-deoxy-2-fluoro-D-ribose-5-phosphate **19b***

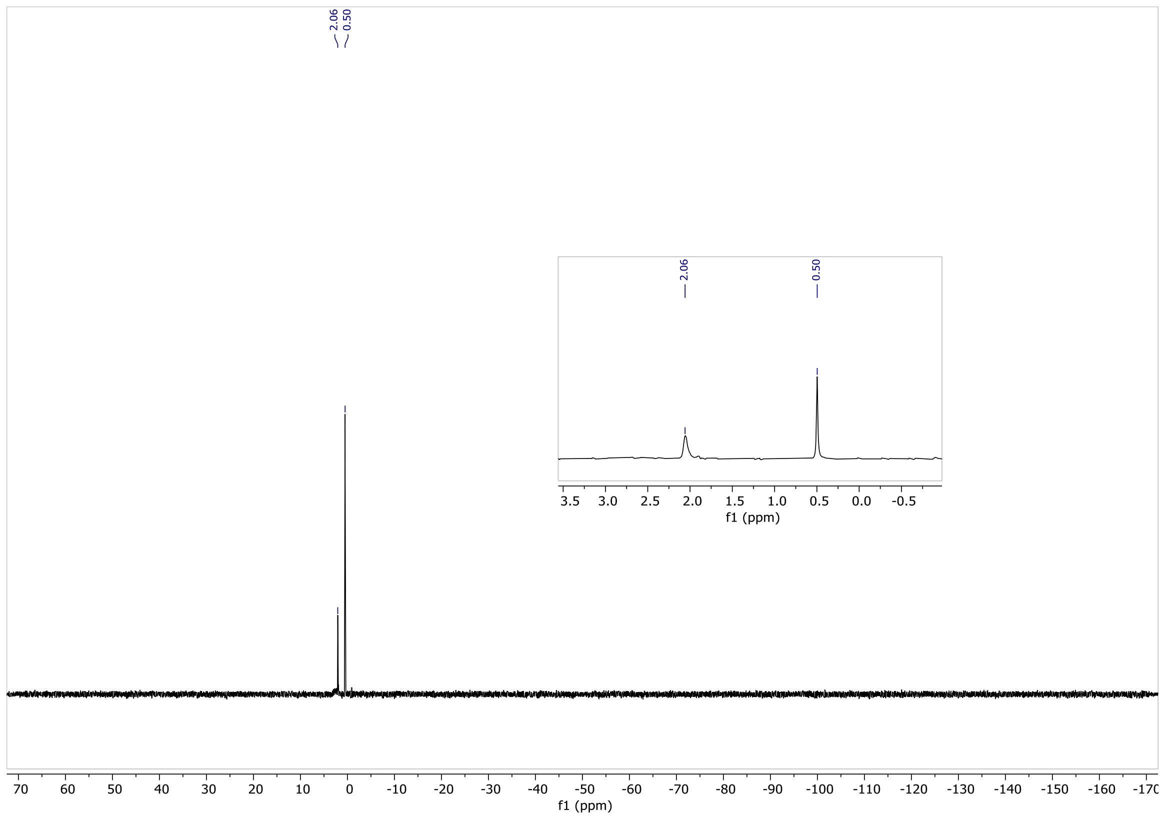

Figure 92: ^{31}P NMR (500 MHz) of 2-deoxy-2-fluoro-D-ribose-5-phosphate ***19b***

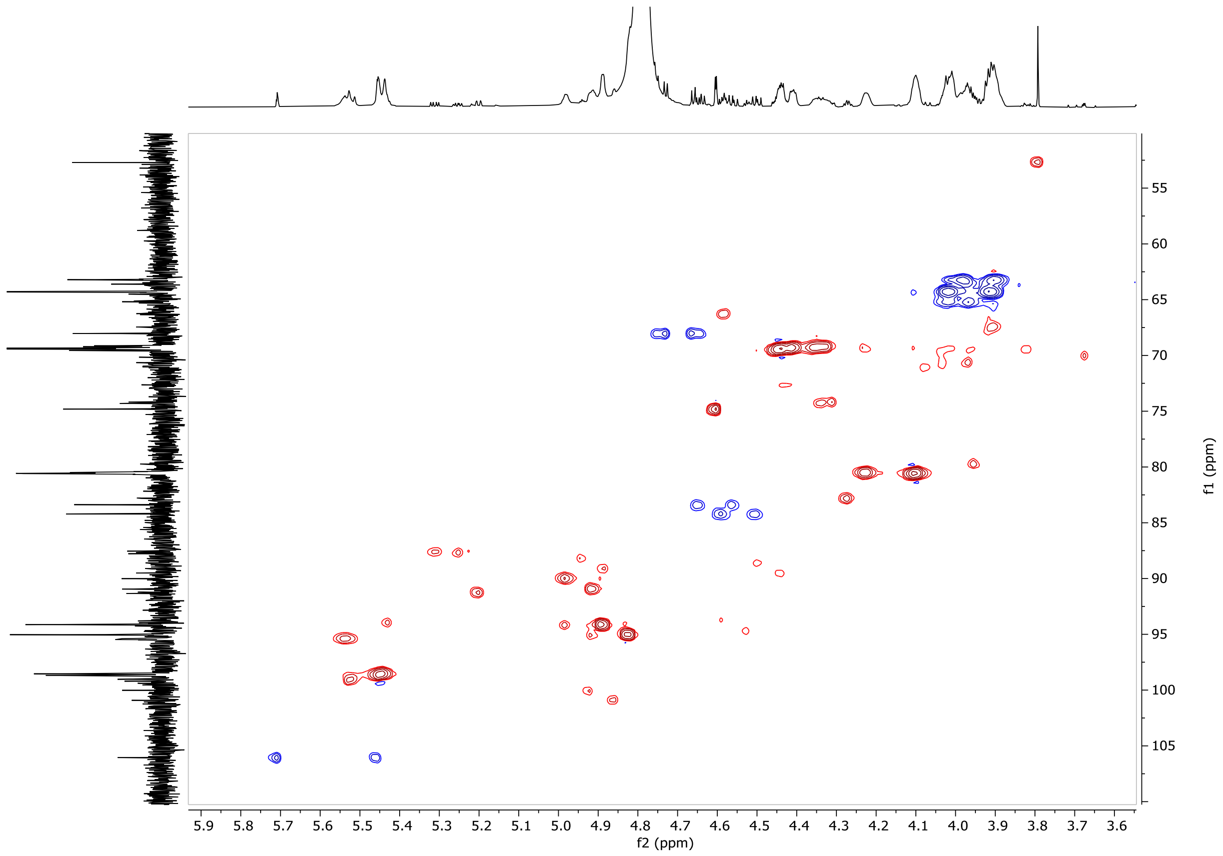

Figure 93: ^{1}H-^{13}C HSQC NMR (500 MHz) of 2-deoxy-2-fluoro-D-ribose-5-phosphate

11.3.5 Scale Up Reaction Conversions

Table 34: peak areas for scale up biotransformations

	Peak Area (samples diluted down to 5 mM)	Concentration (by calibration curve)	Conversion
LKA OMe	3266	3.170873786	63.41747573
LKA MOE	3197	3.103883495	62.0776699
LKA OBn	4495	2.722592368	54.45184737
DKA OMe	3917	3.802912621	76.05825243
DKA MOE	3312	3.215533981	64.31067961
LKOA F (5 ml)	860	0.834951456	16.69902913
LKOA F (10 x 500 uL)	3171	3.078640777	61.57281553
DKOA F (5 ml)	608	0.590291262	11.80582524
DKOA F (10 x 500 uL)	2351	2.282524272	45.65048544

11.4 50 ml scale synthesis of 2-deoxy-2-methoxy-D-ribose-5-phosphate (**11b**)

To a 50 ml falcon tube, D-Glyceraldehyde **16** (20 mM, 1 mmol, 90 mg), methoxyacetaldehyde **2i** (80 mM, 4 mmol, 296 mg), ATP (5 mol%), PEP (40 mM final concentration), and $MgCl_2$ (20 mM). HEPES buffer (pH 8.2) and milliQ water were added, so as to give a final buffer concentration of 50 mM and a final reaction volume of 50 ml. The pH of the reaction mixture was readjusted to 8.2 by dropwise addition of 0.5 M NaOH. After pH adjustment, the reaction was initiated by addition of $DERA_{F76A}$ (4 mg/ml), DHAK (0.1 mg/ml), and PK (10 U/ml). The reaction mixture was left shaking in an orbital incubator at 30 °C 200 RPM for 18 hours. After this time the enzyme was removed using a 10k MWCO filter viva spin.

The phosphorylated products **11b** was purified again using anion exchange chromatography. Due to the binding capacity of the prepacked columns, and the higher amounts of product present in this reaction, after initial purification through 1 x 5 ml column, the initial flowthrough was collected, readded to the washed column and purified again. Five total purification cycles were required to remove all of the desired product from the reaction mixture.

Product **11b** was isolated as a yellow/white solid (158.5 mg, 62% yield)

Diagnostic peaks, such as the anomeric protons (H1) and methoxy protons (H6) are almost identical to that of the previous scale up. A small shift for the C**H₂**OPO₃²⁻ protons (H5) is seen, as expected, due to differences in pH and concentration between different samples.

Major:

^{1}H NMR (500 MHz): 3.49 (s, 1H H6), 3.73 (dd, J = 4.9, 2.9 Hz, 1H, H2), 3.85-3.99 (m, 2H, H5), 4.06 (dd, J = 9.4, 4.8 Hz, 1H, H3), 4.39 (dd, J = 5.1, 5.1 Hz, 1H, H3), 5.32 (d, J = 2.9 Hz, 1H, H1).

^{13}C NMR (125 MHz) 57.97 (C6), 65.07 (d, J = 5 Hz, C5), 69.57 (C3), 82.12 (d, J = 8.2 Hz, C4), 84.32 (C2), 98.94 (C1).

^{31}P NMR (400 MHz): 3.61 (s, P5)

Minor

^{1}H NMR (500 MHz): 3.50 (s, 1H, H6), 3.87 (m, 1H, H2, overlapping with H5 major/minor), 3.88 (m, 2H, H5, overlapping with H5 major), 4.06 (dd, J = 9.6, 4.8, 1H, H4), 4.24 (m 1H, H3), 5.46 (d, J = 4.3 Hz, 1H).

^{13}C NMR (125 MHz) 58.39 (C6), 64.48 (d, *J* = 5.0 Hz) 69.02 (C3), 79.68 (C2), 82.94 (d, *J* = 8.6 Hz), 95.43 (C1).

^{31}P NMR (400 MHz): 3.61 (s, P5)

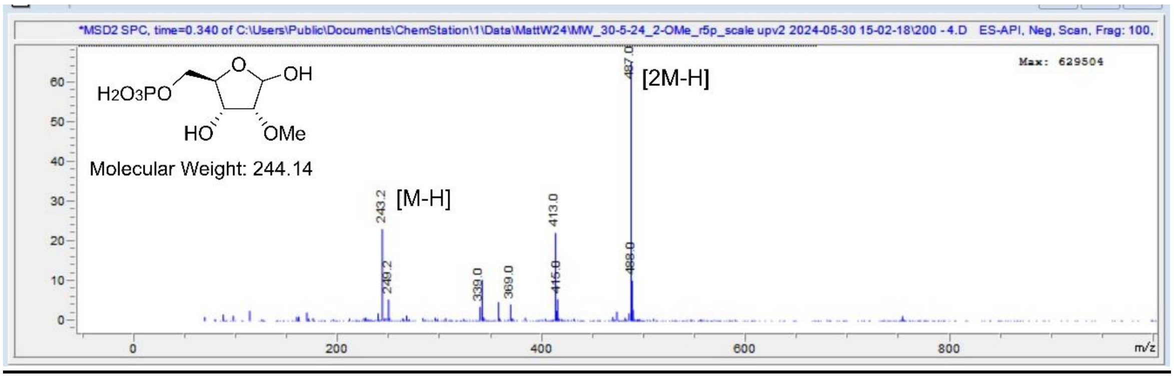

Figure 94: ESI- mass spectrum of 50 ml scale up product ***11b***

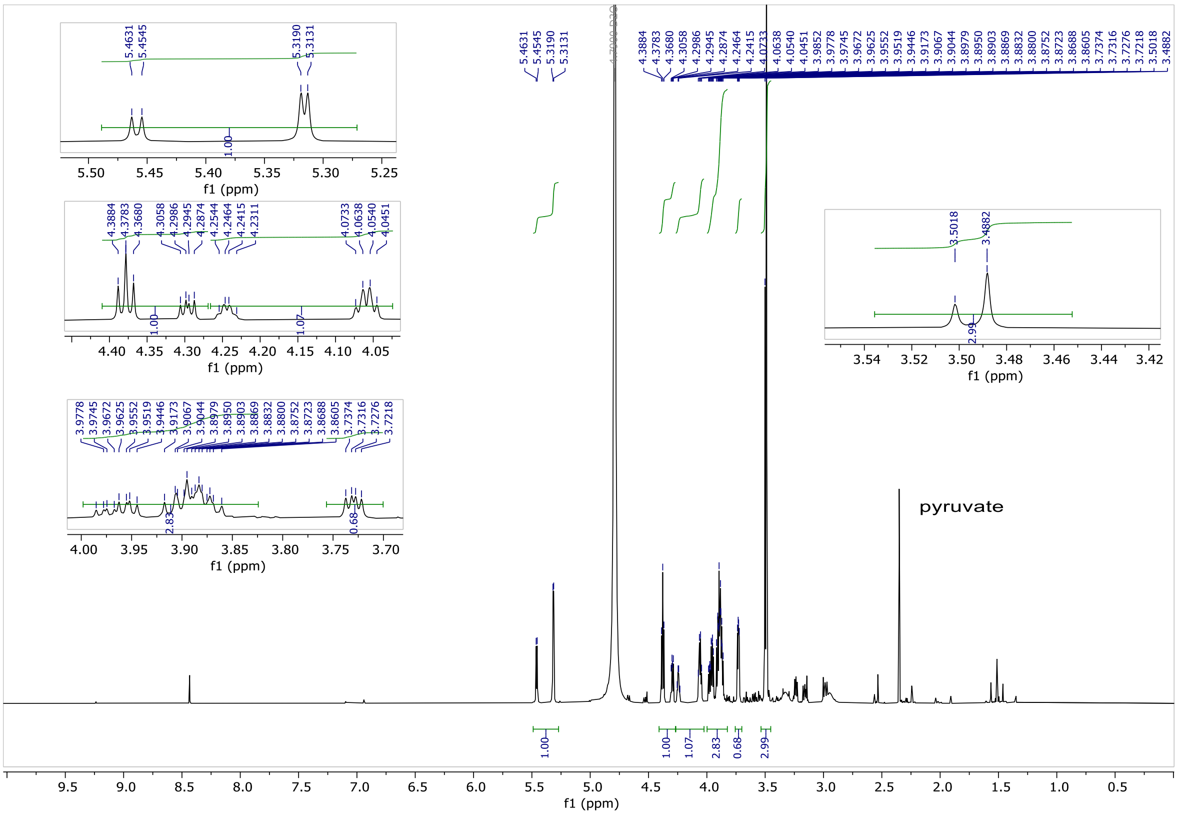

*Figure 95: ^{1}H NMR (500 MHz) of 50 ml scale up product **11b***

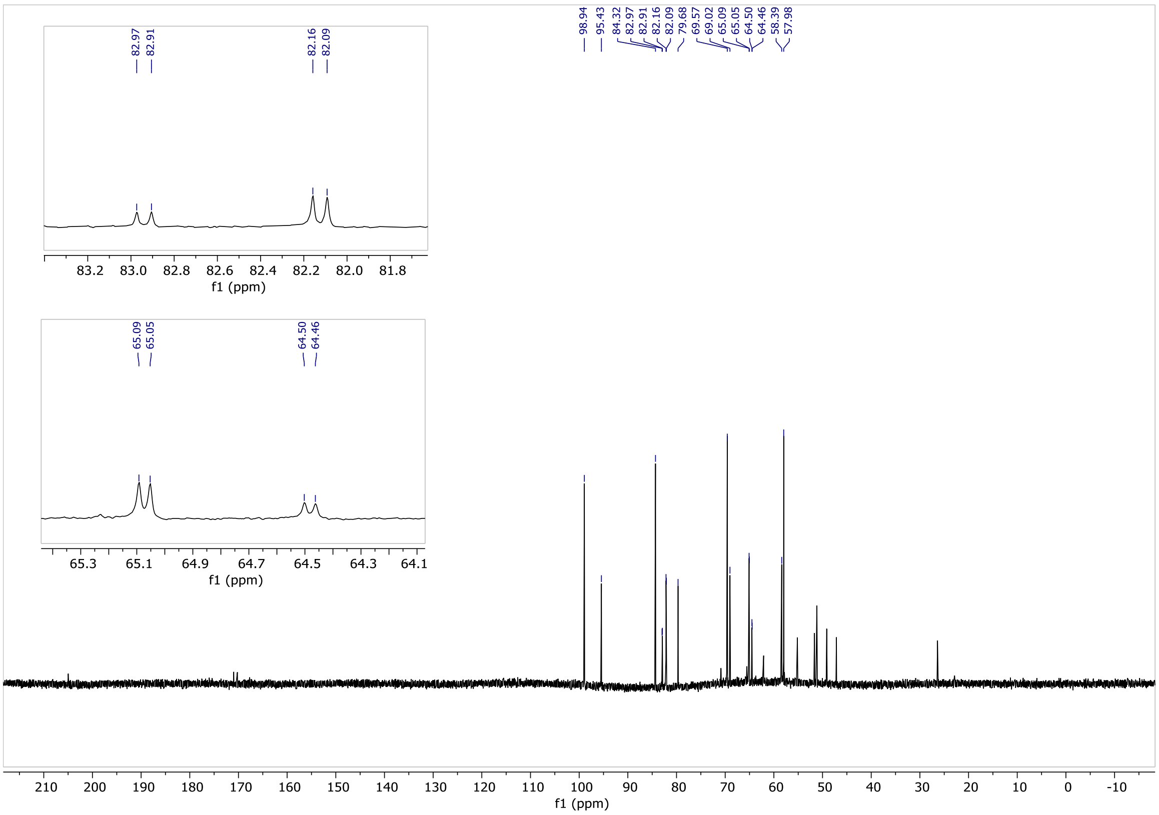

*Figure 96: ^{13}C NMR (500 MHz) of 50 ml scale up product **11b***

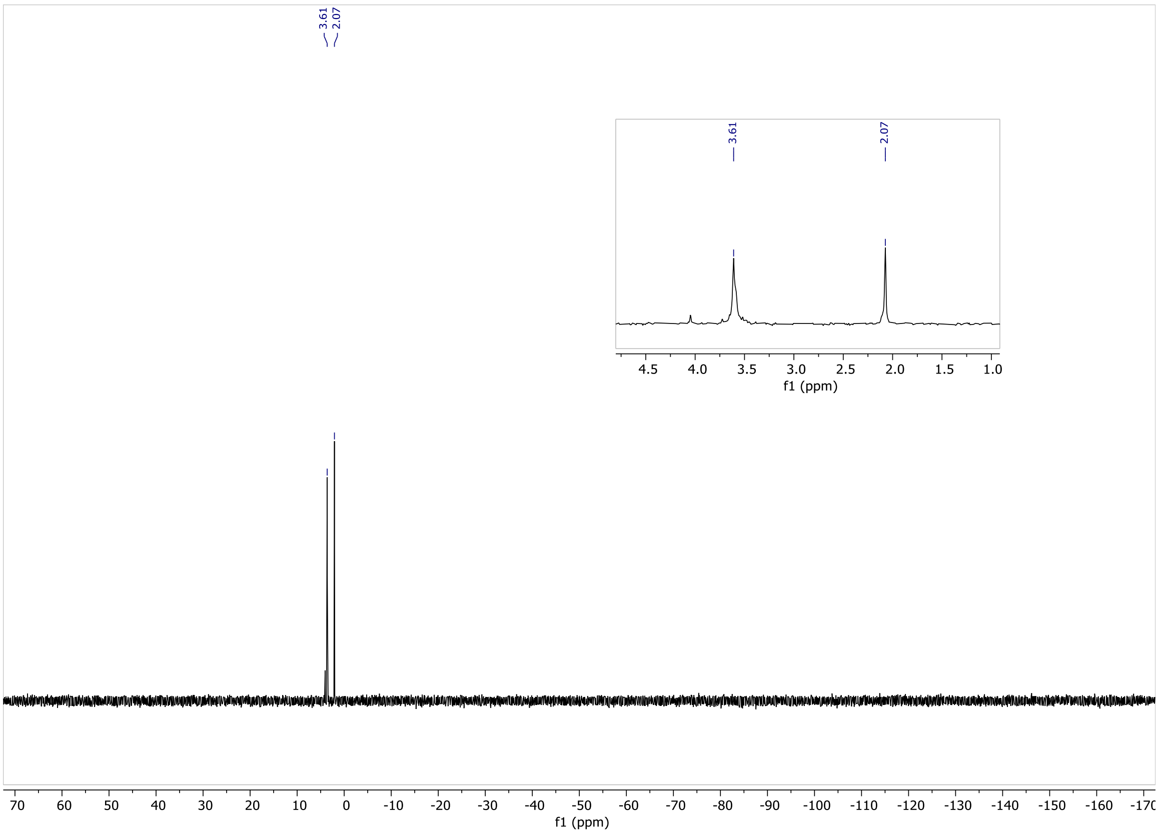

Figure 97: ^{31}P NMR (500 MHz) of 50 ml scale up product **11b**

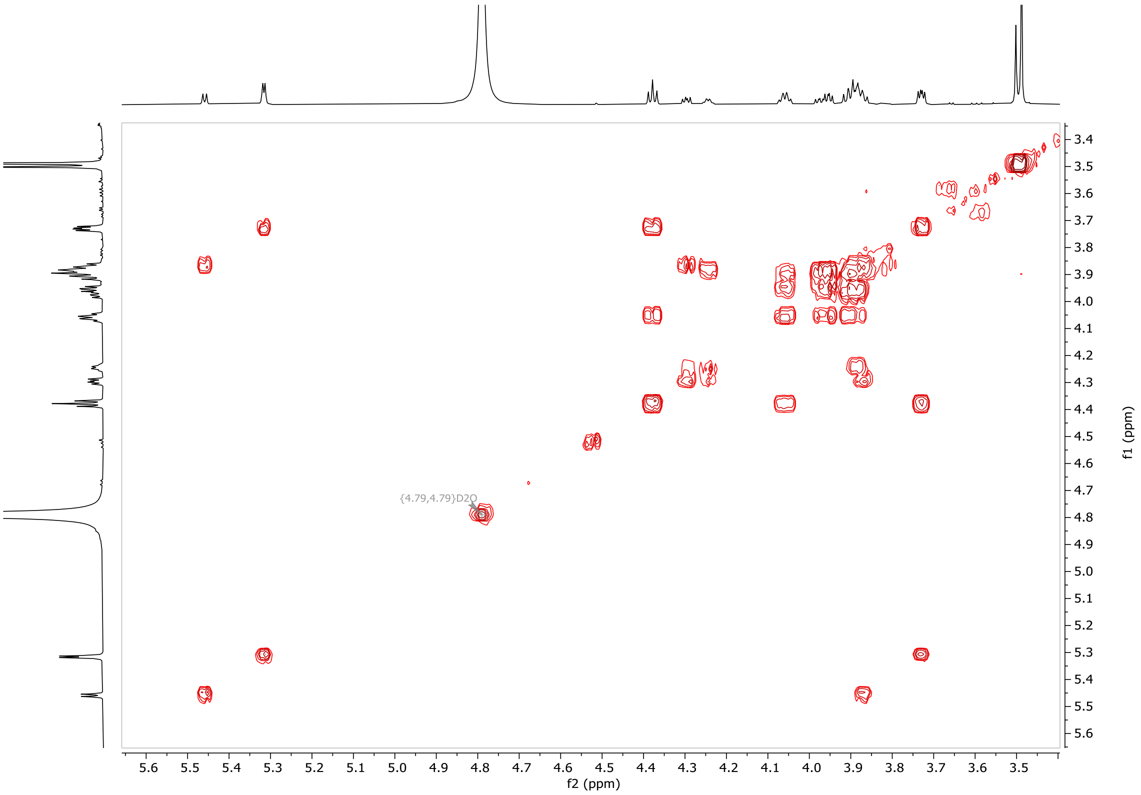

Figure 98: ^{1}H-^{1}H COSY NMR (500 MHz) of 50 ml scale up product **11b**

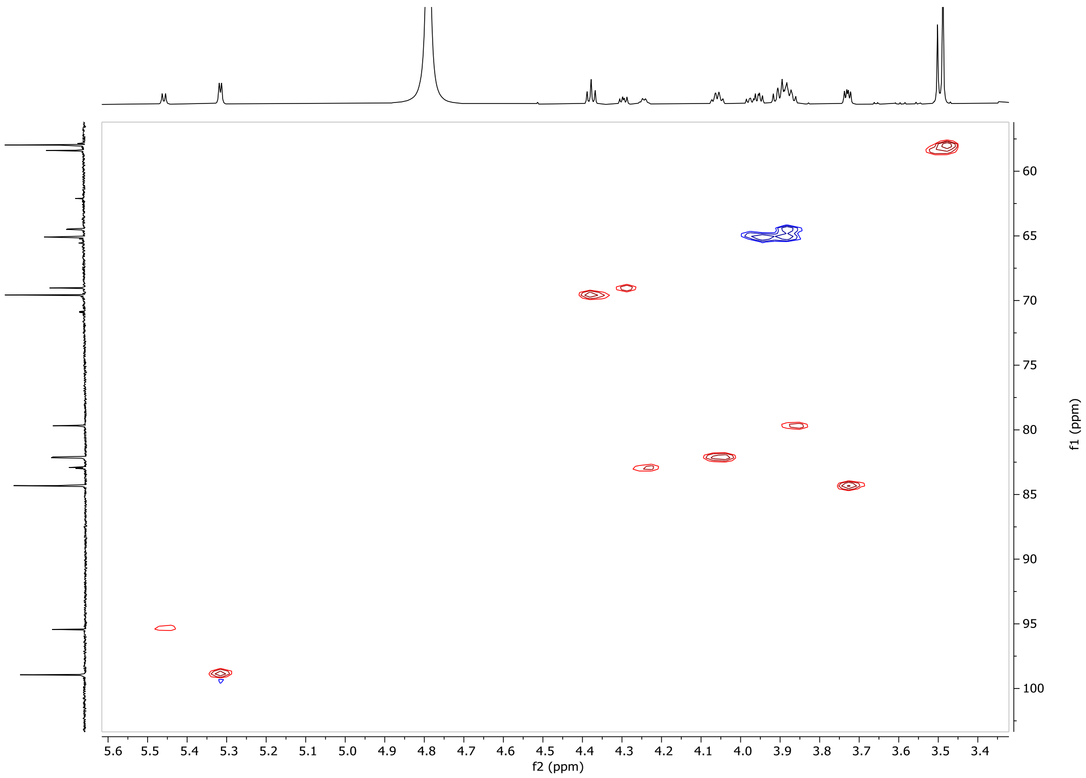

*Figure 99: ^{1}H-^{13}C HSQC NMR (500 MHz) of 50 ml product **11b***

11.5 Assignment of Stereochemistry

11.5.1 Analytical HPLC

For analytical HPLC, use of racemic DL-glyceraldeyde-3-phosphate **1** as a substrate generated two peaks by HPLC. These correspond to the two diastereomers subsequently generated at C4. When generating glyceraldehyde-3-phosphate stereoselectively via kinase enzymes only a single product peak is observed for both D and L Glyceraldehyde (**1b** and **1a**). This suggests that only a single diasteromer of product is present. As the stereocentre at C4 is set by the stereochemistry of the glyceraldehyde starting material, there must be only a single diastereomer at C2 and C3 present. Based on previous work these are likely both set “down” by the aldolase.

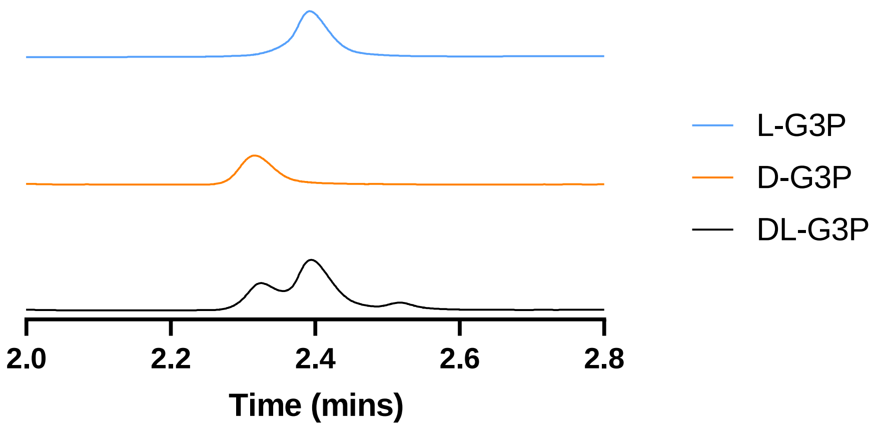

Figure 100: Example HPLC peaks for DL, D and L products

11.5.2 NOESY NMR

To further confirm the assignment of stereochemistry NOESY NMR was used. To carry out these assignments the assumption was made that the stereocentre from D or L glyceraldehyde (**16**/**15**) will not change throughout the reaction and can therefore be used to assign the remaining stereocentres. For 2-deoxy-2-methoxy-L-lyxose-5-phosphate (**11a**) the H4 proton is set on the top face of the sugar (S) by the L-G3P acceptor **15**. When looking at the NOESY NMR strong interactions between H4-H3 and H3-H2 are present and indicate that both the H3 proton and the H2 proton can interact with each other through space. As the H4 proton is set on the top face of the sugar the H3 proton must also be set on the top face of the sugar, making the stereochemistry at positions 2 and 3 (R, R).

In addition, a strong interaction is seen between then H2 and H4 protons which would not be present if these protons were on different faces. This NMR, combined with the presence of only a single diastereomer of derivatised product seen in the HPLC traces, alongside the previously published stereoselectivity of the DERA[5], confirms the enzyme sets the stereocenters at 2 and 3 as (R, R). This is further confirmed by the presence of only two diastereomers for the anomeric and methoxy protons, corresponding to the two anomers in solution, and implying the presence of only a single diastereomer at the remaining positions.

When considering 2-deoxy-2-methoxy-D-ribose-5-phosphate (**11b**) the proton at the 4 position will instead be set on the bottom face of the sugar (R). When comparing to the spectra from **11a**, a strong interaction is observed between H2 and H3. no strong interactions are present

between H4 and H3, or between H4 and H2. These interactions, alongside the single peak observed by the HPLC, and the previously published stereoselectivity of DERA[5], again suggest that H2 and H3 are on the same face as each other, this time on the opposite face to H4. This is again supported by the presence of only two diastereomers in the 1H NMR, corresponding to the two anomers in solution, and implying the presence of a single diastereomer at the remaining positions.

These spectra demonstrate DERA is able to the two stereocenters at the 2 and 3 positions as (R, R), independently of the stereochemistry at the 4-position. This is in agreement with previous literature[5,7].

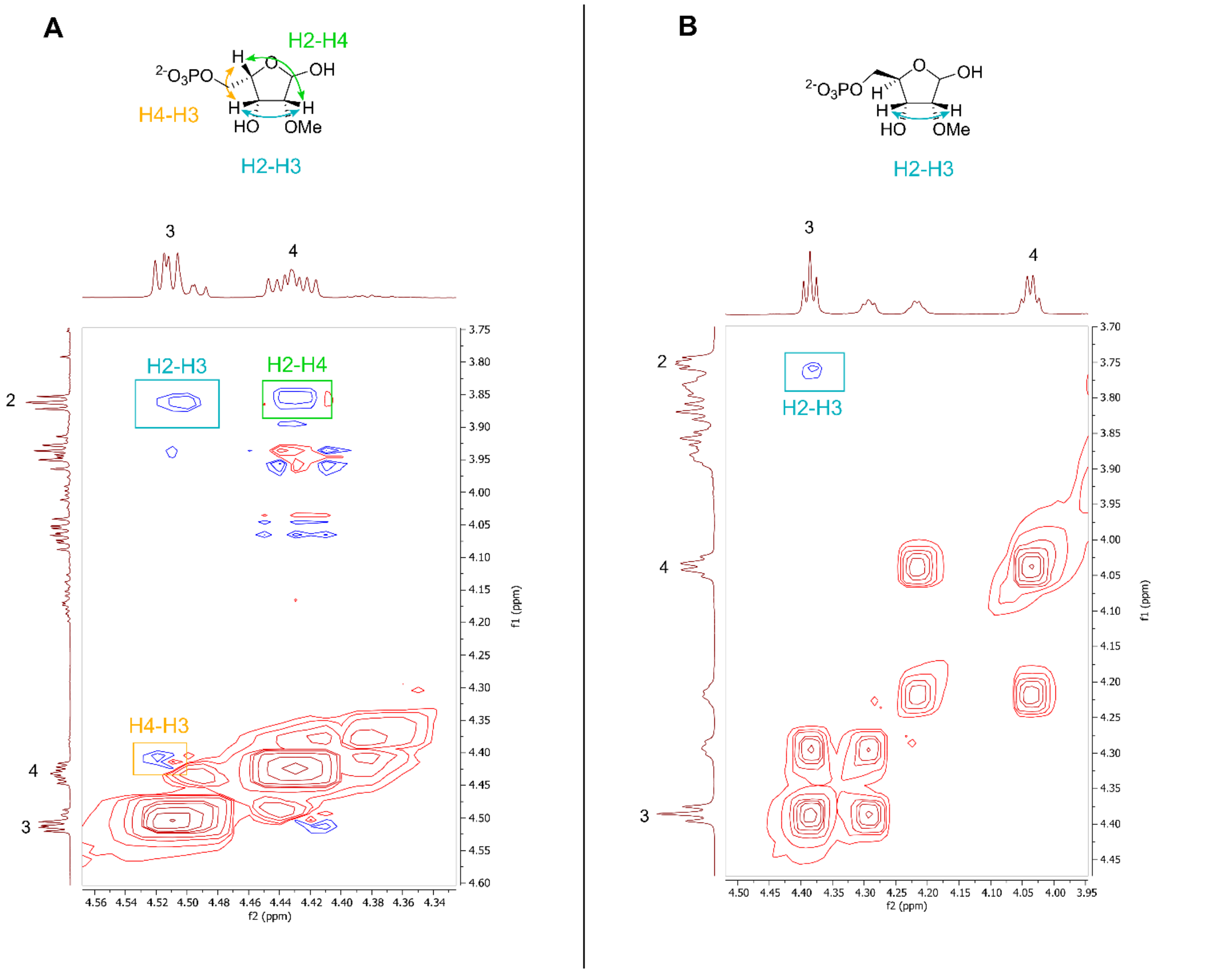

*Figure 101: ^{1}H-^{1}H NOESY NMR assignment of stereochemistry for 2'-OMe products **11a/11b***

In addition to the HPLC and the NOESY NMR, the previously published mechanism for DERA[5] is in agreement with the stereoselectivity observed for these products. Furthermore, only a single diastereomer of product is observed by NMR for the later syntheses of 2'-OH and 2'-

Me adenosine implying only a single diastereomer of the corresponding 5-phosphates are formed.

12. Full Cascade Reactions

12.1 Adenosine (**23**)

A 2x reaction mixture was made up containing D-glyceraldehyde (2 mM), glycolaldehyde (8 mM), ATP (10 mol%), phosphoenolpyruvate (4 mM), adenine (2 mM) $MgCl_2$ (2 mM), $MnCl_2$(2 mM), glucose bisphosphate (0.02 mM), and HEPES buffer (50 mM, pH 8). A 2x enzyme mixture was made up containing DHAK (0.2 mg/ml), DERA (4 mg/ml), PPM (0.2 mg/ml), PNP (0.2 mg/ml) and pyruvate kinase (20 U/ml). To initiate the reaction, 100 µL of enzyme mix was added to 100 µL of reaction mixture, giving final reaction concentrations half those stated above. The reactions were left shaking in an orbital incubator at 30 °C, 200 rpm for 4 hours. The reactions were quenched by additions of equal volumes methanol. Samples were filtered and then analysed directly by HPLC.

12.1.1 Biotransformation Protocol

12.1.2 Example HPLCs

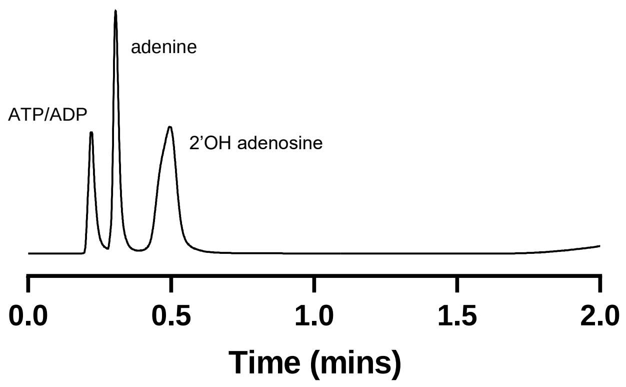

*Figure 102: Example HPLC for biocatalytic synthesis of adenosine **25***

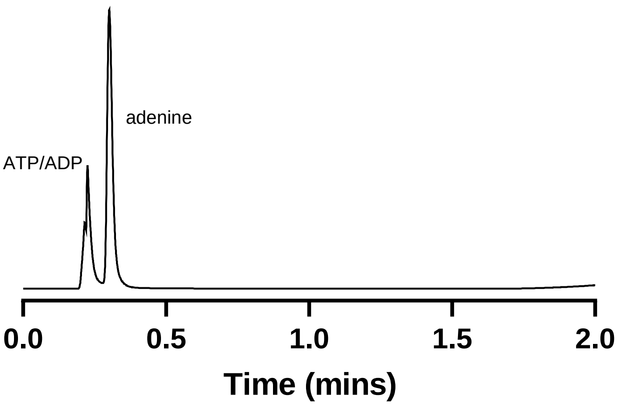

Figure 103: Example HPLC no enzyme control

12.1.3 Mass Spectra

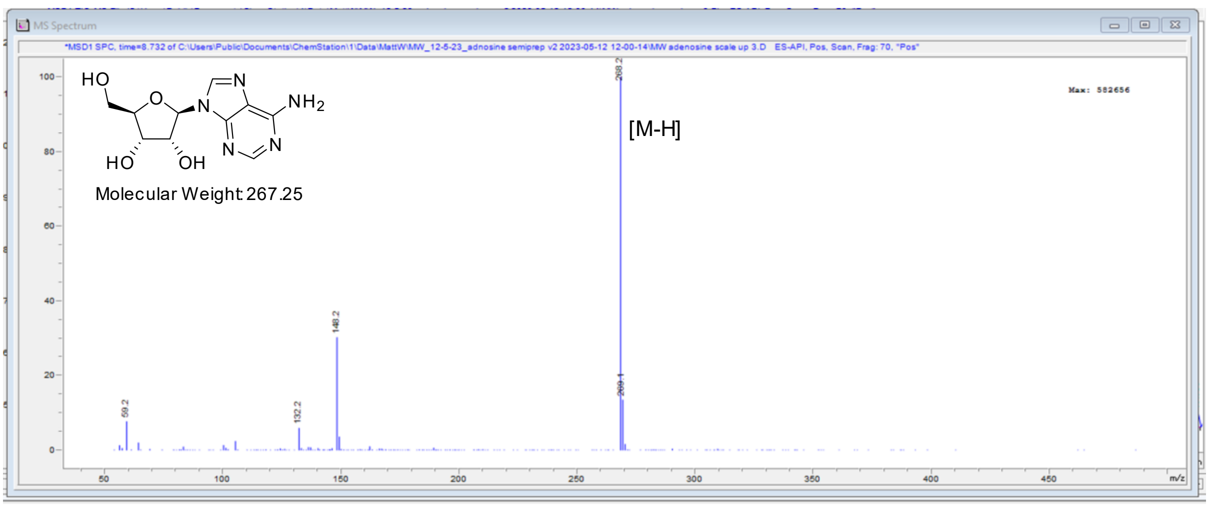

*Figure 104: ESI-+ mass spectrum of adenosine product (**25**), [M+H] = 268.2*

12.1.4 Table of Conversions

*Table 35: peak areas for biocatalytic synthesis of adenosine **25***

DERA	PPM	PNP	Adenine Peak Area	Adenosine Peak Area	Conversion
WT (2mg/ml)	0.1	0.1	1160 ± 28	1037 ± 18	47 ± 1.0
F76A (2mg/ml)	0.1	0.1	817 ± 20	1276 ± 17	61 ± 0.3

12.2 2'-Me Adenosine (**25**)

12.2.1 Biotransformation Protocol

A 2x reaction mixture was made up containing D-glyceraldehyde (20 mM), propanal (40 mM), ATP (10 mol%), PEP (80 mM), adenine (2 mM), $MgCl_2$ (20 mM), $MnCl_2$ (20 mM), glucose bisphosphate (0.02 mM), and HEPES buffer (50 mM, pH 8). A 2x enzyme mixture was made up containing DHAK (0.2 mg/ml), DERA (4 mg/ml) PPM (2 mg/ml) PNP (8 mg/ml) and pyruvate kinase (20 U/ml) in HEPES buffer (50 mM, pH 8). To initiate reaction, 100 µL of enzyme mix was added to 100 µL of reaction mixture. Reactions were left shaking at 30 °C for 18 hours. Reactions were quenched by addition of equal volumes methanol. Samples were filtered and then analysed directly by HPLC.

12.2.2 Example HPLCs

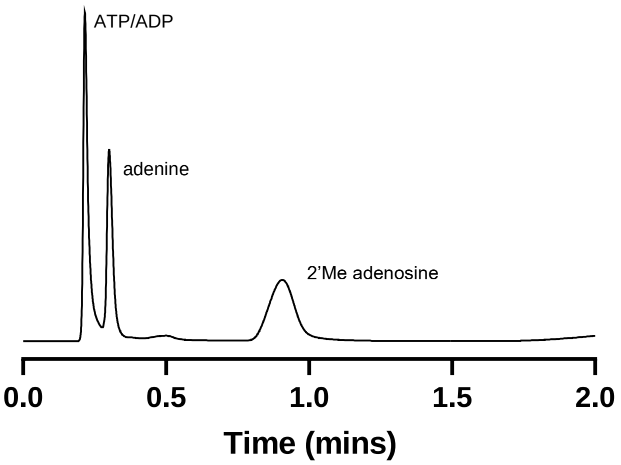

*Figure 105: Example HPLC biotransformation for the synthesis of 2-Me adenosine **27***

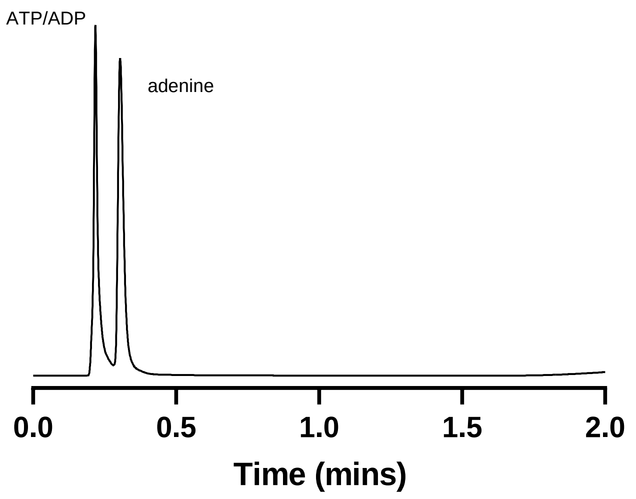

Figure 106: Example HPLC no enzyme control

12.2.3 Mass Spec

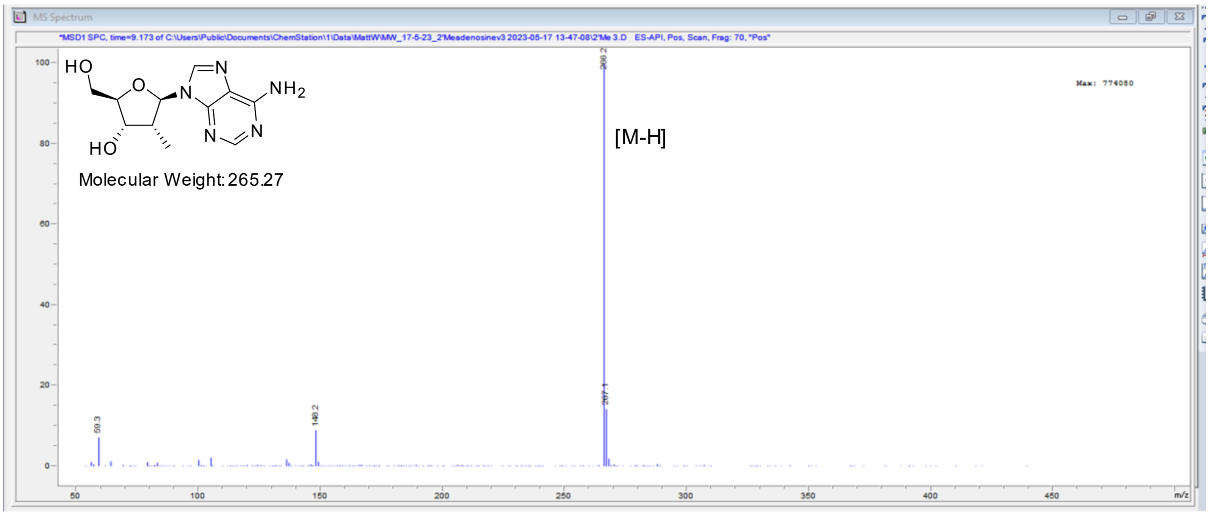

*Figure 107: ESI+ mass spectrum of 2' Me adenosine (**27**), [M+H] = 266.2*

12.2.4 Table of Conversions

Sugar Excess	DERA	PPM (mg/ml)	PNP (mg/ml)	Adenine Peak Area	2'Me-Adenosine Peak Area	Conversion (%)
1x	WT (2 mg/ml)	1	2	2070 ± 24	n.d	n.d
10x	WT (2 mg/ml)	1	2	1549 ± 11	335 ± 3.3	17 ± 0.1
10x	F76A (2 mg/ml))	1	2	1179 ± 15	739 ± 7	38 ± 0.5

10x	F76A (2 mg/ml)	1	4	675 ± 137	1401 ± 216	65 ± 7.8

12.3 2'-F Adenosine (**26**)

12.3.1 Biotransformation Protocol

A 2x reaction mixture was made up containing D-glyceraldehyde (20 mM), fluoroethanol (40 mM), ATP (1 mM), PEP (80 mM), adenine (2 mM), $MgCl_2$ (20 mM), $MnCl_2$ (20 mM), glucose bisphosphate (0.02 mM), and HEPES buffer (50 mM, pH 8). A 2x enzyme mixture was made up containing dihydroxyacetone kinase (0.2 mg/ml), DERA (4 mg/ml), *Pp*AO (1 mg/ml), PPM (2 mg/ml), PNP (8 mg/ml), and pyruvate kinase (20 U/ml) in HEPES buffer (50 mM, pH 8). To initiate the reaction, 100 µL of enzyme mix was added to 100 µL of reaction mixture, giving a final reaction concentrations half those stated above. Reactions were left shaking at 30 °C for 18 hours and then quenched by additions of equal volumes methanol. Samples were filtered and then analysed directly by HPLC.

12.3.2 Example HPLCs

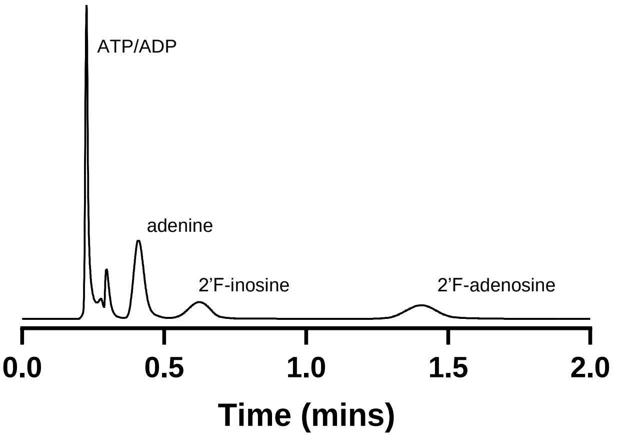

*Figure 108: Example HPLC Biotransformation for the synthesis of 2'-F adenosine **28***

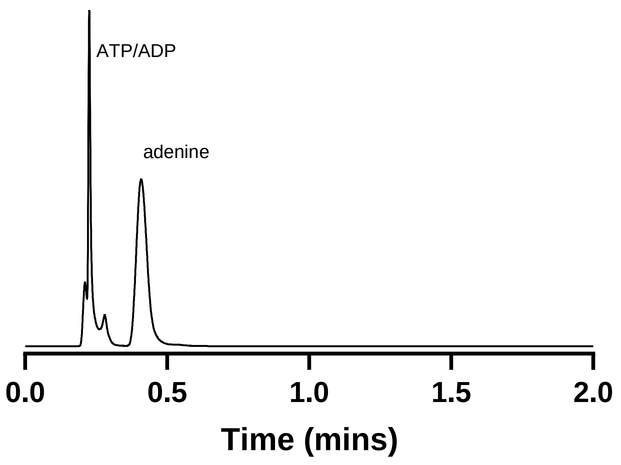

Figure 109: Example HPLC no enzyme control

12.3.3 Mass Spec

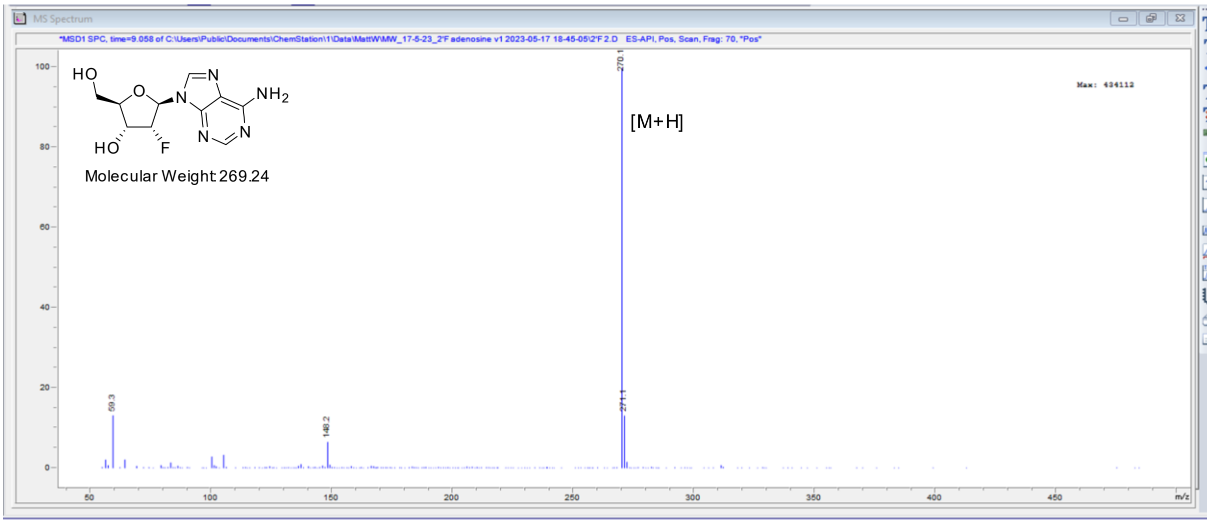

*Figure 110: ESI+ mass spectrum of 2'-F -adenosine (**27**), [M+H] = 270.1*

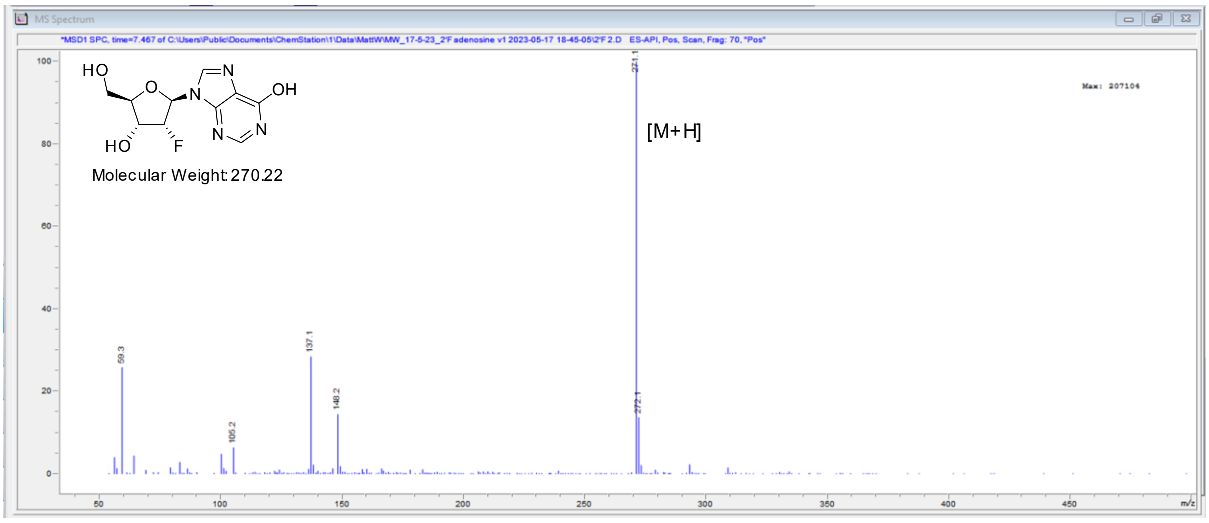

Figure 111: ESI+ mass spectrum of 2'-F -inosine, [M+H] = 271.1

12.3.4 Table of Conversions

Table 36:

Sugar Excess	DERA	PPM	PNP	Adenine Peak Area	2'F Adenosine Peak Area	2'F Inosine Peak Area	Conversion (%)
1x	WT (2 mg/ml)	WT (1 mg/ml)	WT (2 mg/ml)	2211 ± 68	0	0	0
10x	WT (2 mg/ml)	WT (1 mg/ml)	WT (2 mg/ml)	1326 ± 59	98 ± 6.5	149 ± 1.9	6 ± 0.60
10x	WT (2 mg/ml)	WT (1 mg/ml)	WT (4 mg/ml)	1176 ± 8	397 ± 13	387 ± 5	20 ± 0.5
10x	WT (2 mg/ml)	WT (1 mg/ml)	2M (4 mg/ml)	1087 ± 12	590 ± 18	468 ± 3	28 ± 0.5

12.4 Guanosine (**24**)

12.4.1 Biotransformation Protocol

A 2x reaction mixture was made up containing D-glyceraldehyde (2 mM), glycolaldehyde (8 mM), ATP (10 mol %), PEP (4 mM), guanine (2 mM), $MgCl_2$ (2 mM) $MnCl_2$ (10 mM), glucose bisphosphate (0.02 mM), and HEPES buffer (50 mM, pH 8). A 2x enzyme mixture was made up containing dihydroxyacetone kinase (0.2 mg/ml), DERA (4 mg/ml) PPM (0.2 mg/ml) PNP (0.2 mg/ml) and pyruvate kinase (20 U/ml). To initiate the reaction, 100 µL of enzyme mix was added to 100 µL of reaction mixture giving final reaction concentrations half those stated. Reactions were left shaking at 30 °C for 4 hours. Reactions were quenched by additions of equal volumes of methanol. Samples were filtered and then analysed directly by HPLC.

12.4.2 Calculation of Conversion

As guanine appeared to show poor solubility in water under the reaction conditions, comparison of product and starting material peaks was not a valid method of calculating conversion. Instead of this a calibration curve was generated using guanosine as a standard.

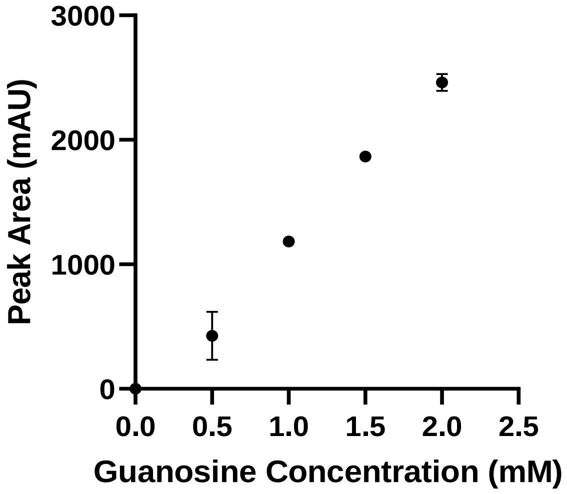

Figure 112: Guanosine calibration curve. Error bars represent standard deviation of triplicate repeat biotransformations, where not visible error bars are hidden behind the points.

Concentration of Guanosine	Peak Area
0	0
0.5	425 ± 158
1	1183 ± 22
1.5	1867 ± 30
2	2461 ± 56

Conversions were calculated using the following formula:

$$\left(\frac{A/_{C}}{S}\right) \times 100\%$$

Where A = peak area from product in HPLC at 200 nm

C = slope of calibration curve, typically using ribose-5-phosphate as a product standard

S = concentration of limiting reagent, typically the aldehyde acceptor at 5 mM concentration.

12.4.3 Example HPLCs

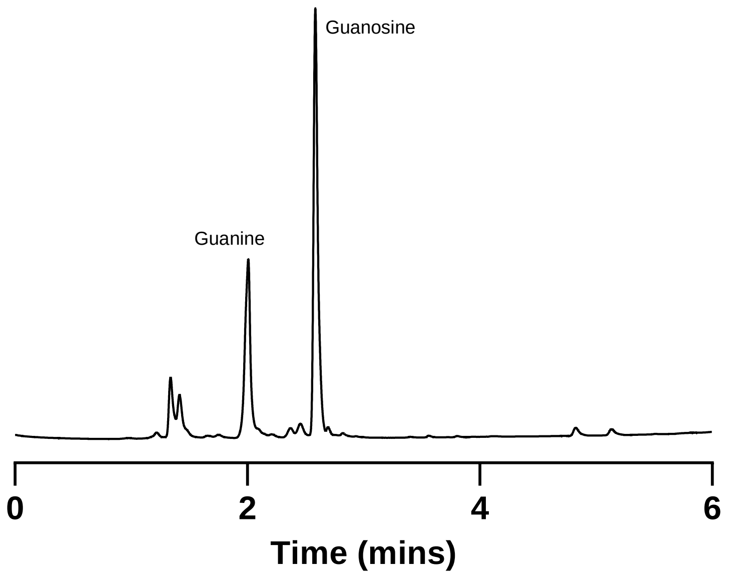

*Figure 113: Example HPLC biotransformation for the synthesis of guanosine **26***

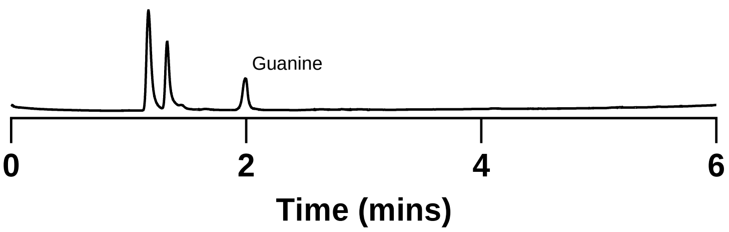

Figure 114: Example HPLC no enzyme control

Interestingly the area of guanine in the starting material is lower than the total amount of guanine and guanosine in the biotransformation. This is likely due to the low solubility of guanine and would suggest that conversion to guanosine seems to help solubilise some of the insoluble guanine starting material.

12.4.4 Mass Spectra

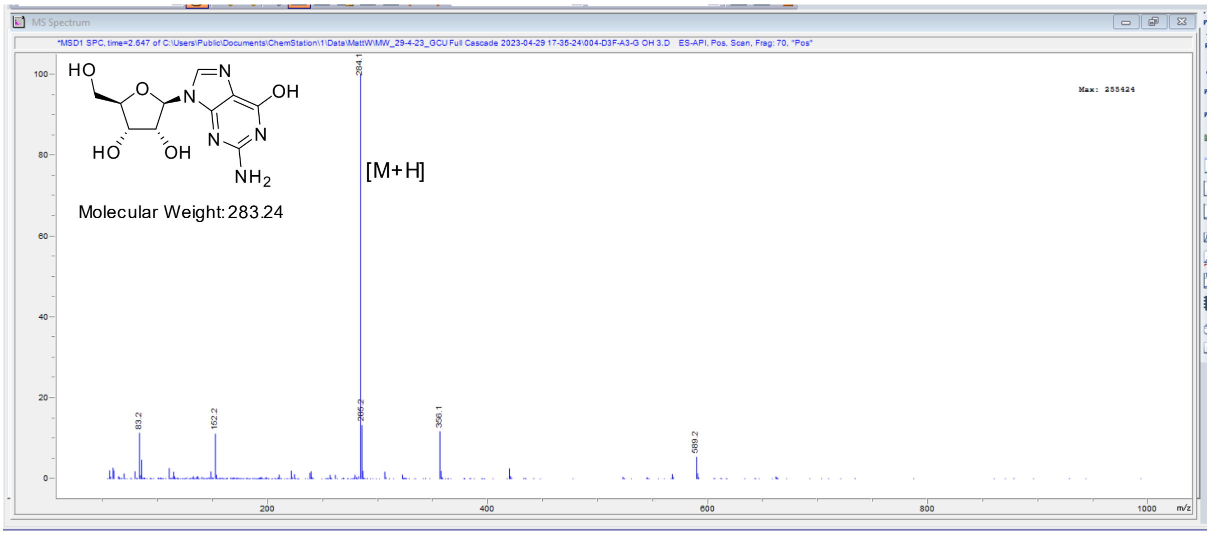

*Figure 115: ESI+ mass spectrum of guanosine biotransformation product (**26**), [M+H] = 284.1*

12.5 Vidarabine (**27**)

12.5.1 Biotransformation Protocol

A 2x reaction mixture was made up containing D-glyceraldehyde (2 mM), glycolaldehyde (8 mM), ATP (10 mol%), PEP (4 mM), adenine (2 mM), $MgCl_2$ (2 mM), $MnCl_2$ (2 mM), glucose bisphosphate (0.02 mM), and HEPES buffer (50 mM, pH 8). A 2x enzyme mixture was made up containing DHAK (0.2 mg/ml), FSA (2 mg/ml) PPM (0.2 mg/ml) PNP (0.2 mg/ml) and pyruvate kinase (20 U/ml). To initiate reaction, 100 µL of enzyme mix was added to 100 µL of reaction mixture, giving final reaction concentrations half those stated above. Reaction were left shaking in an orbital incubator at 30 °C, 200 rpm for 4 hours. Reactions were quenched by additions of equal volumes methanol. Samples were filtered and then analysed directly by HPLC.

12.5.2 Example HPLC

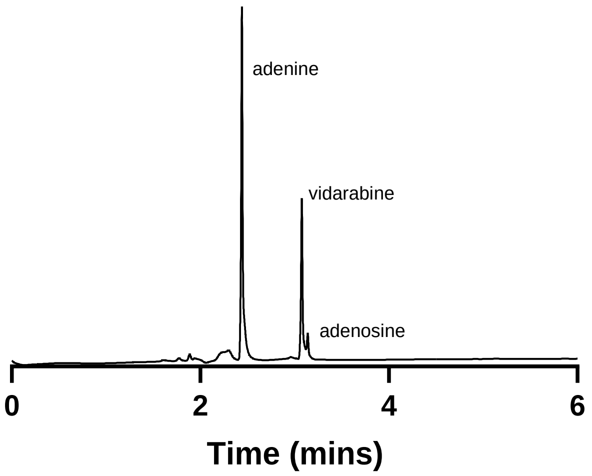

*Figure 116: Example HPLC biotransformation for the synthesis of vidarabine **29***

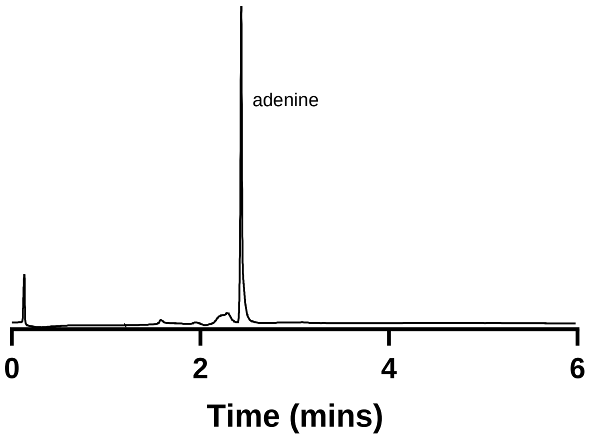

Figure 117: Example HPLC no enzyme control

12.5.3 Stereoselectivity

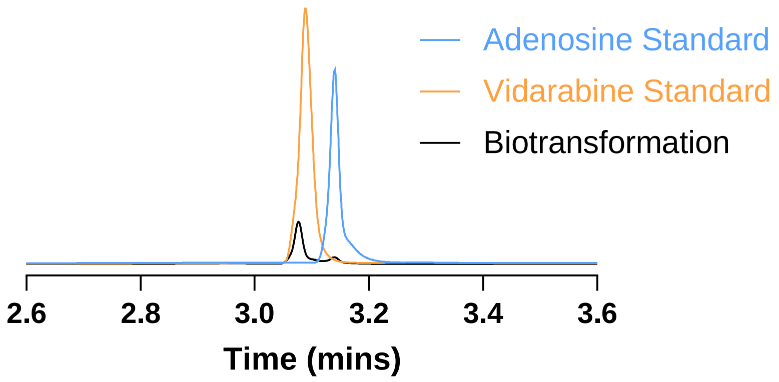

*Figure 118: HPLC trace of biotransformation product peak alongside standards for adenosine **25** and vidarabine **29***

12.5.4 Mass Spectra

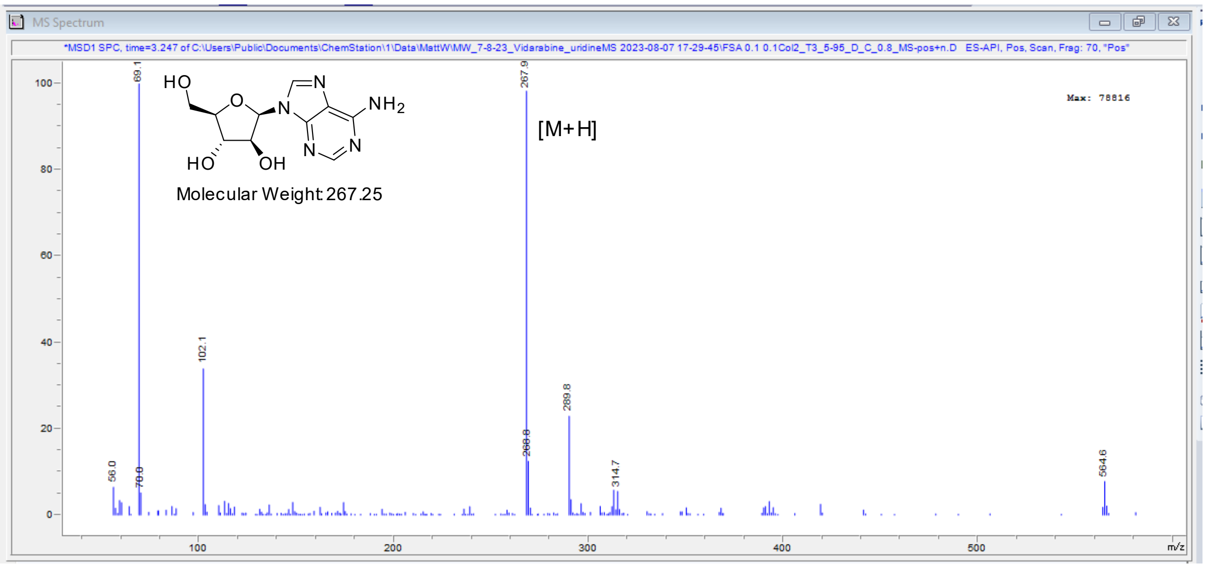

*Figure 119: ESI- mass spectrum of vidarabine (**29**), [M+H] = 267.9*

13. Enzyme Loading DOE

To determine which of the three enzymes has the most significant effect on reaction conversion, design of experiment was used to plot a response surface considering DERA concentration, PPM concentration and PNP concentration.

13.1 Table of Values

Table 37: Reactions conditions for enzyme loading DOE

	Aldolase (mg/ml)	PPM (mg/ml)	PNP (mg/ml)	adenine	2'Me inosine	2'Me adenosine	Conversion (%)
1	1	0.5	0.5	1593	15	173	9.71
2	1	0.5	4	1077	49	833	42.52
3	1	1.25	2.25	1197	61	655	34.24
4	1	2	0.5	1554	60	186	10.33
5	1	2	4	972	71	962	47.98
6	2.5	0.5	2.25	1262	39	631	32.66
7	2.5	0.5	2.25	1235	35	611	32.48
8	2.5	1.25	0.5	1551	37	225	12.41
9	2.5	1.25	0.5	1532	36	232	12.89
10	2.5	1.25	2.25	1161	45	738	37.96
11	2.5	1.25	2.25	1179	44	749	37.98
12	2.5	1.25	2.25	1197	47	731	37.01
13	2.5	1.25	4	853	59	1146	55.69
14	2.515	2	2.25	1084	31	767	40.75
15	4	0.5	0.5	1617	40	184	9.99
16	4	0.5	4	1115	54	867	42.58
17	4	1.25	2.25	1217	55	681	34.87
18	4	1.25	2.25	1219	59	660	34.06
19	4	2	0.5	1575	43	155	8.74
20	4	2	4	868	64	1099	54.11

13.2 Results

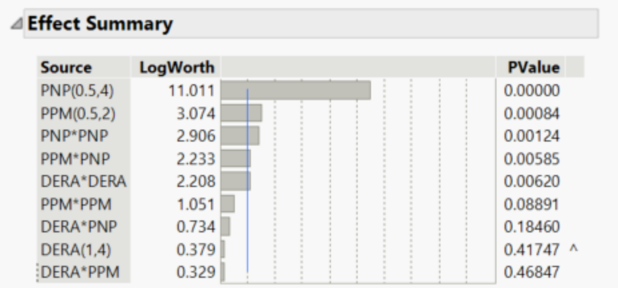

Effect Summary

Source	LogWorth	PValue
PNP(0.5,4)	11.011	0.00000
PPM(0.5,2)	3.074	0.00084
PNP*PNP	2.906	0.00124
PPM*PNP	2.233	0.00585
DERA*DERA	2.208	0.00620
PPM*PPM	1.051	0.08891
DERA*PNP	0.734	0.18460
DERA(1,4)	0.379	0.41747 ^
DERA*PPM	0.329	0.46847

Figure 120: DOE model factors and their significance. P values were calculated using two-sided, sequential T tests, no adjustments were made for multiple comparisons.

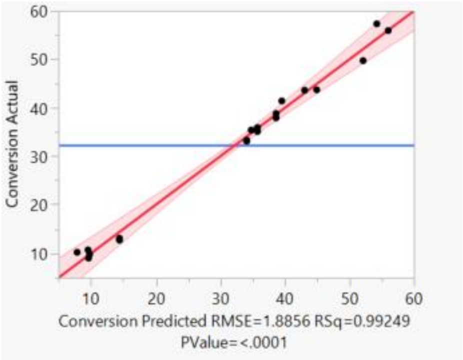

Figure 121: actual conversion vs predicted conversion, upper and lower bands represent 95% confidence intervals. P value was calculated using a one-sided F test. Adjustments were not made for multiple comparisons

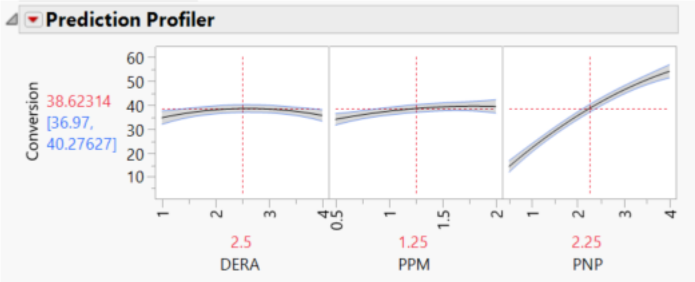

Figure 122:DOE effects of individual factors, the upper and lower bounds indicate 95% confidence intervals.

Predicted conversions from the model gave good correlation with the actual conversions from the experiments. The model generated was dominated by PNP concentration, showing that this enzyme has the lowest activity of the three towards the 2'-Me substrate. For enzyme loading therefore, the optimal conditions were chosen based on maximising the amount of PNP in the solutions.

14. Equilibrium

14.1 Calculated Equilibrium Constants

As with the previous cascades, thermodynamic calculations were carried out using the equilibrator webserver. To calculate the overall equilibrium constant for the cascade, the equilibrium constants for the individual reactions were calculated and then summed together. Known equilibrium constants of phosphorolysis were taken from the literature[8], unknown equilibrium constants were calculated from using the equilibrator webserver[1]. As the initial kinase reaction appears to be functionally irreversible and occurring at a faster rate than the

subsequent aldolase step, the equilibrium constant was instead calculated starting from D-glyceraldehyde-3-phosphate.

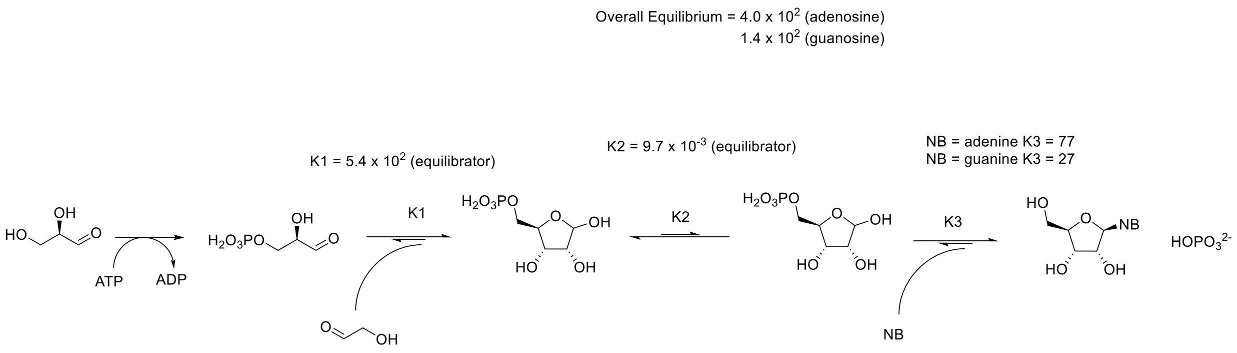

Figure 123: Full cascade reaction scheme with calculated or known equilibrium constants for each of the cascade steps. The overall equilibrium constants for the synthesis of both adenosine and guanosine are shown above.

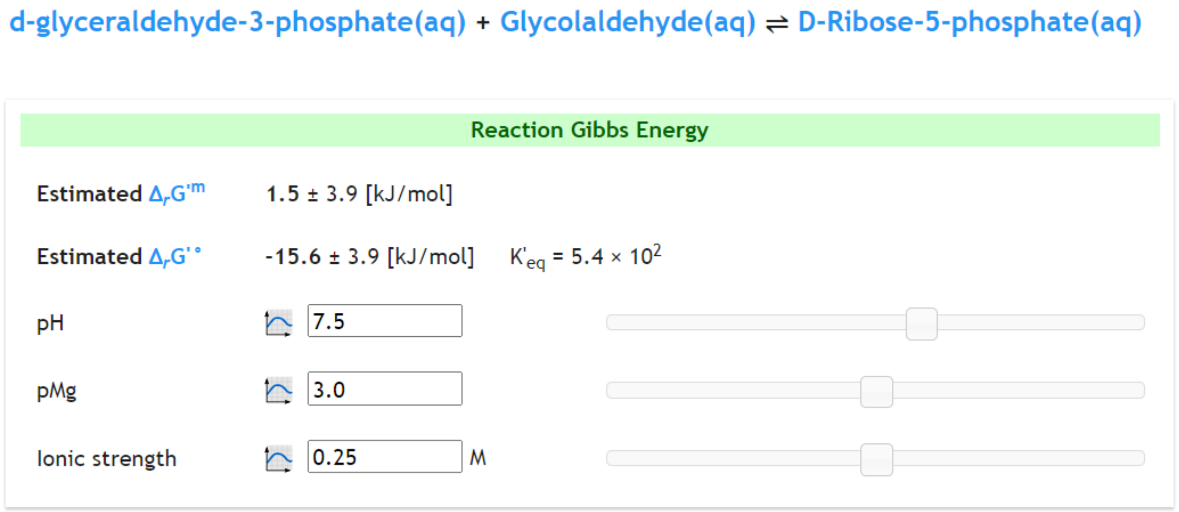

Figure 124: Calculation of the theoretical equilibrium constant for the generation of D-ribose-5-phosphate from G3P and glycolaldehyde. Calculated via equilibrator[1]. Accessed 14/6/22

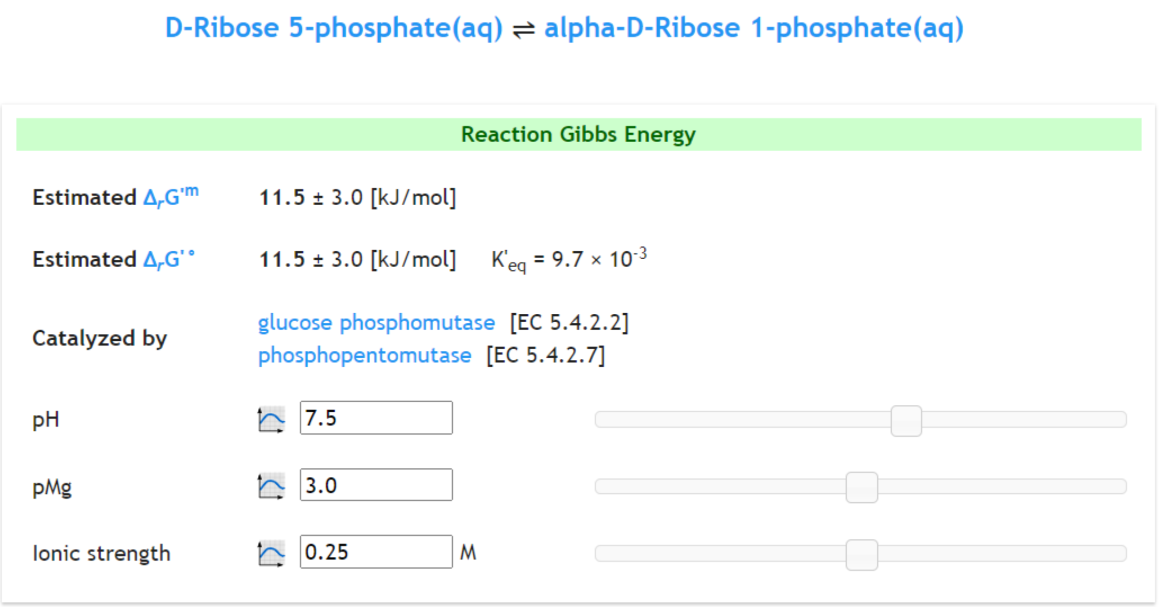

Figure 125: Calculation of the theoretical equilibrium constant for the generation of D-ribose-1-phosphate from D-ribose-5-phosphate. Calculated via equilibrator[1]. Accessed 14/6/22

Phosphorolysis of adenosine K = 0.013[8], therefore for synthesis of adenosine from R1P and adenine, K = 77

Phosphorolysis of guanosine K = 0.037[8], therefore for synthesis of guanosine from R1P and guanine, K = 27

Overall equilibrium for the full cascade synthesis of adenosine, $K = 4.0 \times 10^2$

Overall equilibrium for the full cascade synthesis of guanosine, $K = 1.4 \times 10^2$

14.2 Time Course

To assess the progress of the entire cascade a model reaction was carried out for the synthesis of adenosine and monitored over 5 hours. Under the standard conditions, conversion increased over the first hour but then started to decrease slowly over time. Addition of a 10-fold excess of D-glyceraldehyde both increased the amount of product formed, but also prevented the decrease shift away from product formation at longer reaction times.

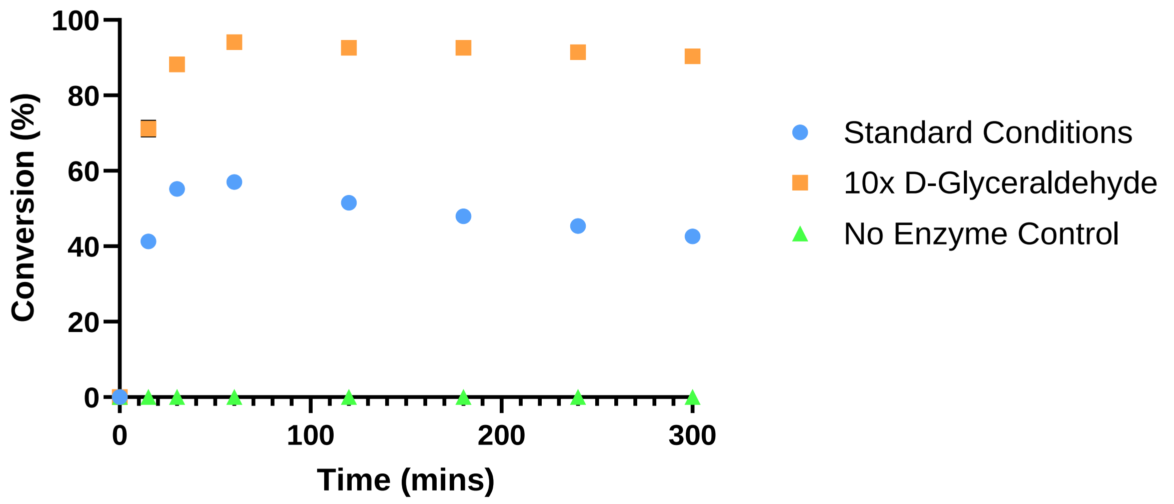

Figure 126: Time course for the biocatalytic synthesis of adenosine, under standard conditions, with a 10x excess of glyceraldehyde starting material and an enzyme-free control. Where not visible error bars for triplicate repeats are hidden under the points.

15. Semi-Preparative Scale Synthesis of Nucleoside Products

To Characterize products by ^{1}H NMR 5 ml scale reactions were carried out and the products were purified by semi-preparative HPLC using the following method:

15.1 HPLC Method

Column = Eclipse XDB C18 5 uM 9.4x250 mM

A = 10 mM ammonium formate pH 4, 95% Water 5% acetonitrile

B = 10 mM ammonium formate pH 4 5% water 95% acetonitrile

Flow = 4 ml/min

Time	A (%)	B (%)
0	100	0
5	100	0
10	50	50
15	50	50
16	100	0
20	100	0

15.2 Adenosine (**23**)

15.2.1 Biotransformation

A 2x reaction mixture was made up containing Adenine **21** (2 mM), D-glyceraldehyde **16** (2 mM), glycolaldehyde **2a** (8 mM), ATP (20 mol%), PEP (4 mM), $MgCl_2$ (2 mM), $MnCl_2$ (2 mM), HEPES buffer (50 mM, pH 8), and water to bring the volume up to 2.5 mls. Alongside this a 2x enzyme mixture was made up containing DHAK (0.2 mg/ml), pyruvate kinase (20 U/ml), $DERA_{F76A}$ (4 mg/ml), PPM (0.2 mg/ml), PNP (0.2 mg/ml), HEPES buffer (50 mM, pH 8) and water to bring the volume up to 2.5 ml. The 2x reaction mix and 2x enzyme mix were combined in a 15 ml falcon tube, giving a final reaction volume of 5 mls and final reaction concentrations half those stated. The reaction was left shaking at 180 rpm at 30 °C for 18 hours (overnight). Enzyme was removed using a 10K MWCO filter and the product was purified via semiprep HPLC. Fractions were collected using mass-based fraction collection for the desired product mass. Fractions were combined, acetonitrile was removed via genevac and then the sample was lyophillsed and isolated as a white solid, resuspended in D_2O and analysed by NMR.

15.2.2 NMR

^{1}H NMR (500 MHz) 3.8 (dd, J = 12.9, 3.6Hz, 1H, H5'), 3.91 (dd, J = 12.9. 2.8 Hz, 1H, H5'), 4.29 (m, 1H, H4'), 4.43 (dd, J = 5.3, 3.3 Hz, 1H, H3'), 4.79 (1H, H2', obscured by water peak), 6.07 (d, J = 6.2 Hz, 1H, H1'), 8.25 (s, 1H, H8), 8.33 (s, 1H, H2).

Known impurities have been annotated on spectrum, peaks not annotated correspond to unknown impurities.

^{1}H NMR for adenosine identical to literature[9]

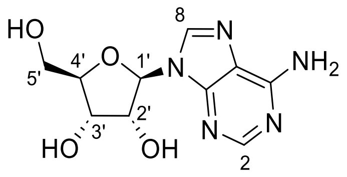

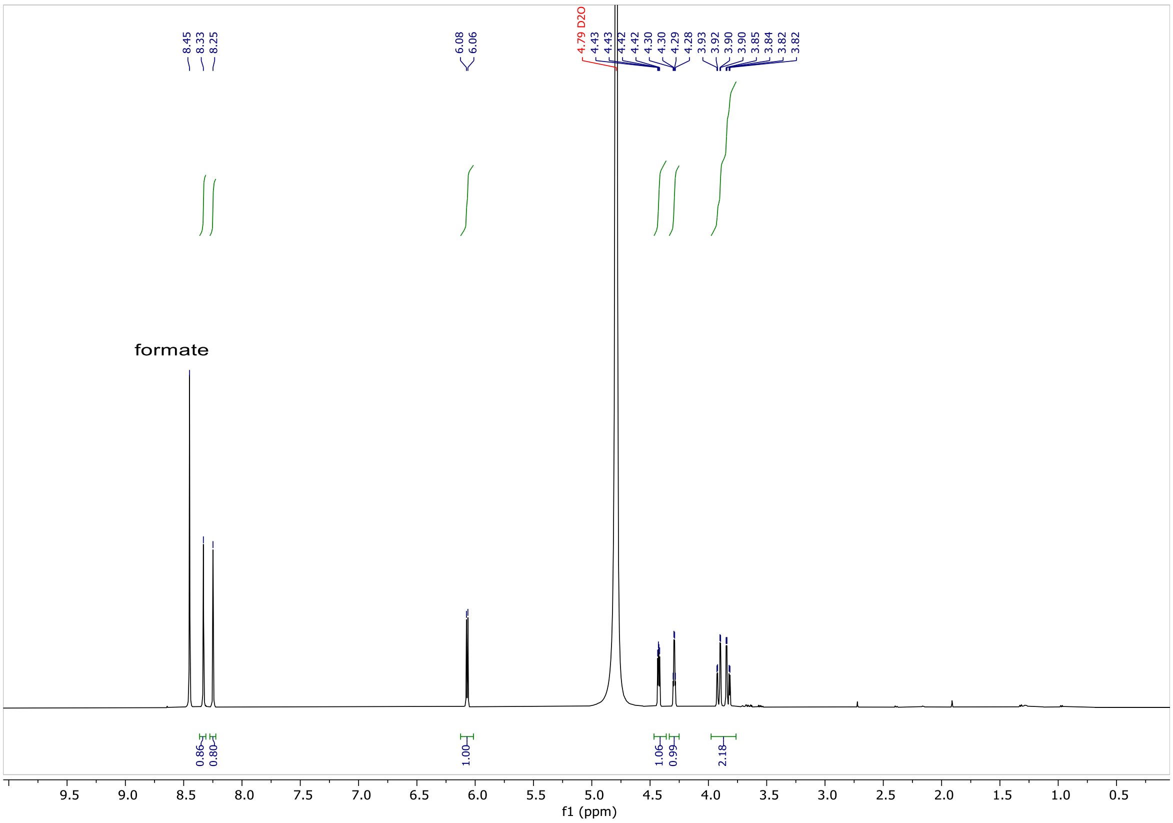

*Figure 127: ¹H NMR (500 MHz) adenosine **25** biotransformation product*

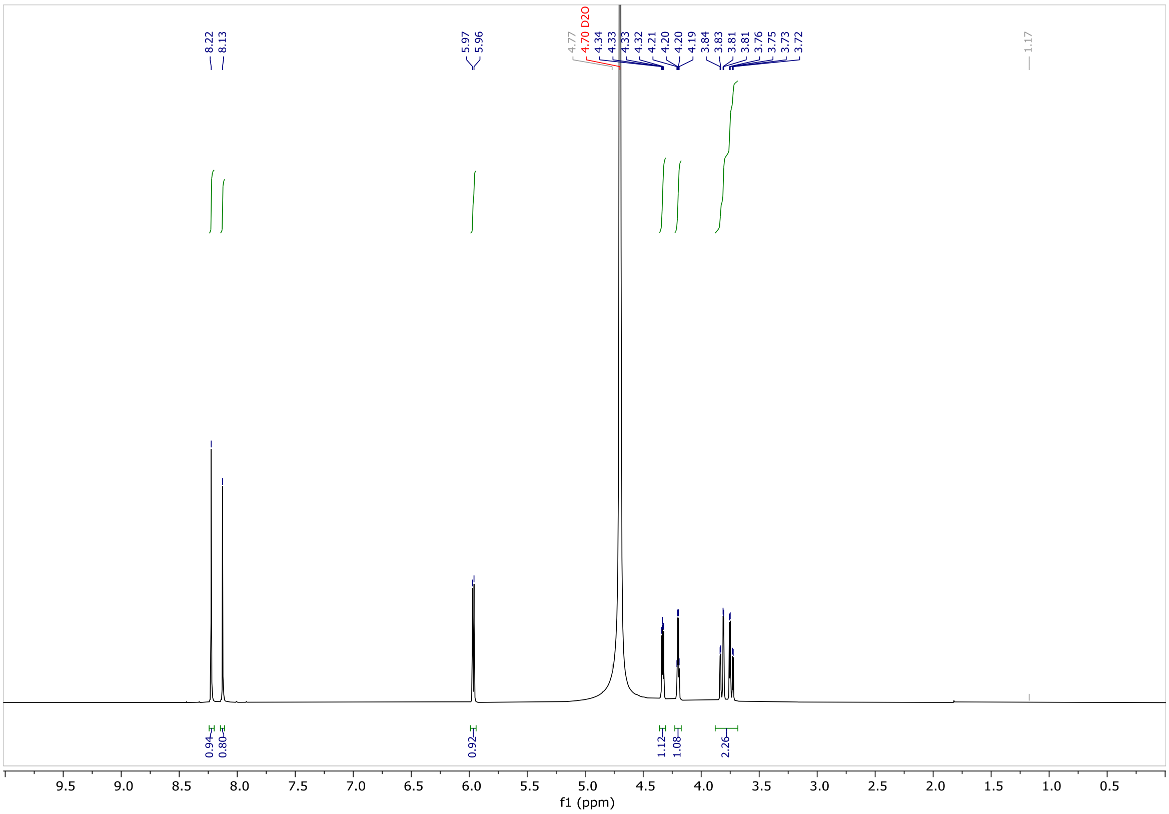

*Figure 128: ¹H NMR (500 MHz) of adenosine **25** standard*

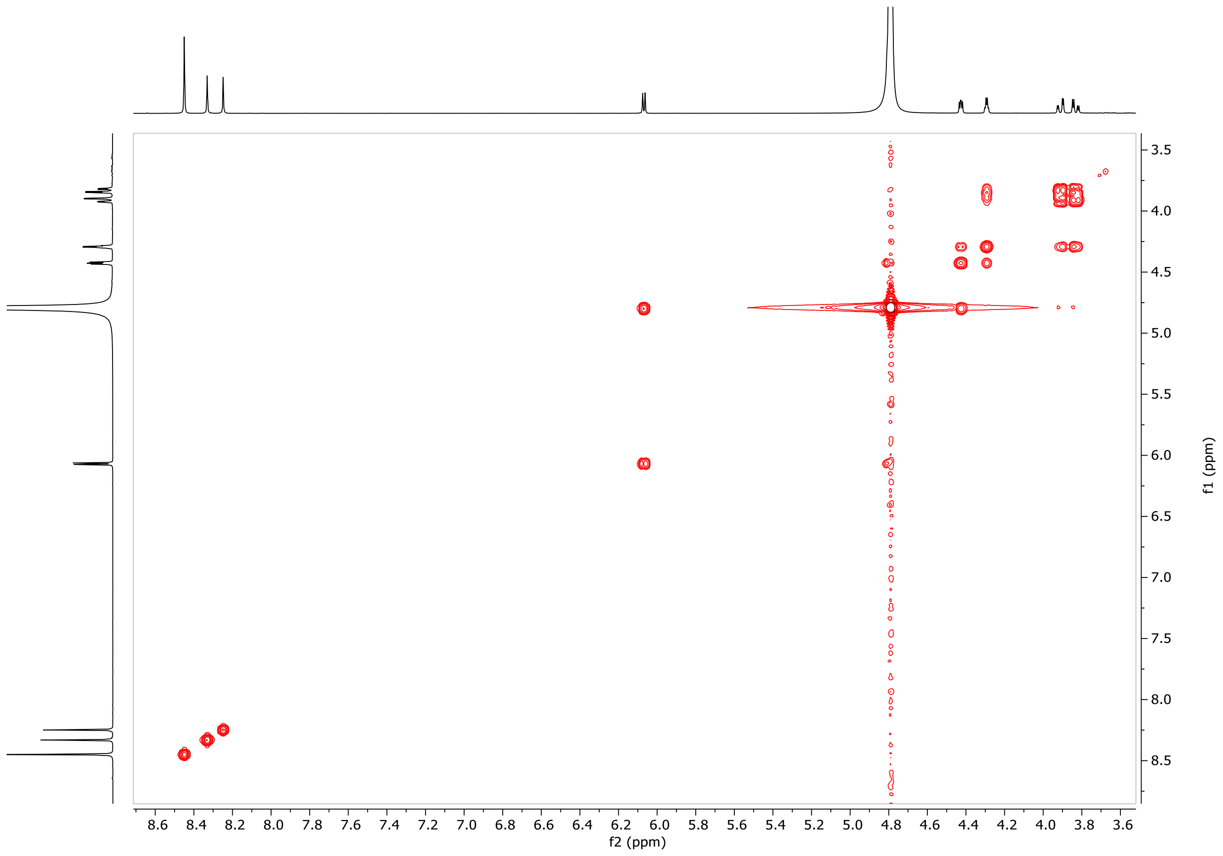

*Figure 129: ^{1}H-^{1}H COSY (500 MHz) of adenosine **25** biotransformation product*

15.3 2'-Me Adenosine (**25**)

15.3.1 Biotransformation

A 2x reaction mixture was made up containing adenine **21** (2 mM), D-glyceraldehyde **16** (20 mM), propanal **2c** (40 mM), ATP (10 mol%), PEP (40 mM), $MgCl_2$ (20 mM), $MnCl_2$ (20 mM), and HEPES buffer (100 mM, pH 8) and water to bring the volume up to 2.5 mls. Alongside this a 2x enzyme mixture was made up containing DHAK (0.2 mg/ml), pyruvate kinase (20 U/ml), $DERA_{F76A}$ (4 mg/ml), PPM (2 mg/ml), PNP (8 mg/ml), HEPES buffer (100 mM, pH 8), and water was added to bring the volume up to 2.5 ml. The 2x reaction mix and 2x enzyme mix were combined in a 15 ml falcon tube giving 5 mls final volume and final reaction concentrations half those stated and left shaking at 180 rpm at 30 °C for 24 hours. Enzyme was removed using a 10K MWCO filter and the product was purified by semiprep HPLC. Fractions were collected using mass-based fraction collection. Fractions were combined, acetonitrile was removed via genevac and then the sample was lyophillsed and isolated as a white solid, resuspended in D_2O and analysed by NMR.

15.3.2 NMR

^{1}H NMR (500 MHz) 1.07 (d, J = 7 Hz, 3H, H6'), 3.0 (m, 1H, H2'), 3.82 (dd, J = 12.6, 4.1 Hz, 1H, H5'), 3.86 (dd, J = 12.6, 3.5, 1H. H5'), 4.24 (dt, J = 3.8,1.45 Hz, 1H, H4'), 4.44 (dd, 5.4, 1.6 Hz, 1H, H3'), 5.98 (d, J = 9.2 Hz, 1H, H1'), 8.24 (s, 1H, H8), 8.34 (s, 1H H2).

Known impurities have been annotated on spectrum, peaks not annotated correspond to unknown impurities.

The ^{1}H NMR peaks from the 2'-Me adenosine product **27** show slight differences in ppm when compared to the literature values[10]. While the peaks are shifted from the literature standard, the coupling constants are virtually identical to that of the published standard, suggesting the same product has been made. The differences in ppm may result from factors such as concentration, pH, or temperature, and without a commercial standard available to run under identical conditions a truly valid comparison is difficult.

It is also worth noting that the NMR of product **27** shows differences in both ppm and coupling constants to the literature values of the 2'-ara-methyl adenosine analogue[11], suggesting that is it not simply the other C2'-diastereomer being formed.

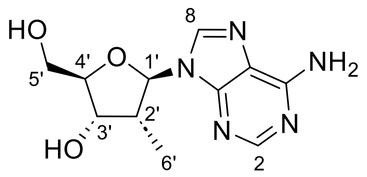

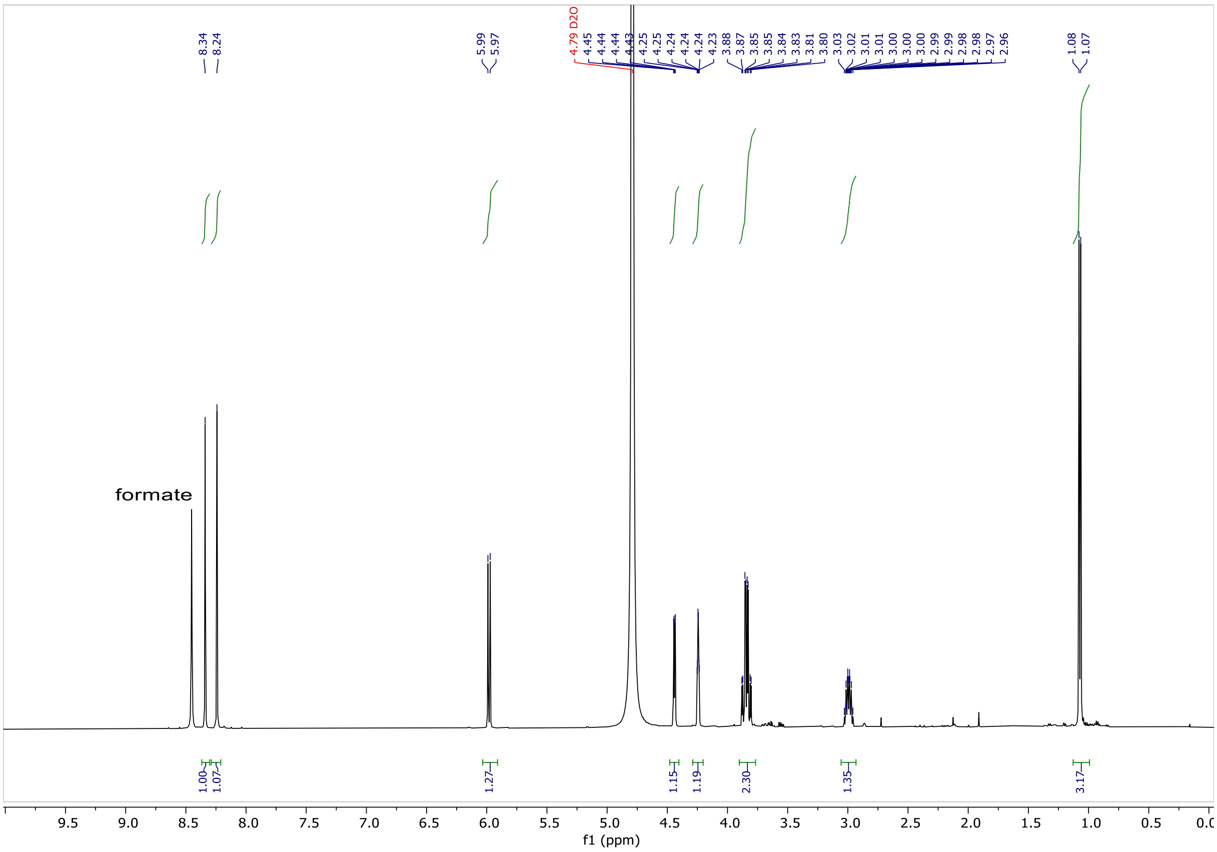

*Figure 130: ^{1}H NMR (500 MHz) of 2’Me-adenosine **27** biotransformation product*

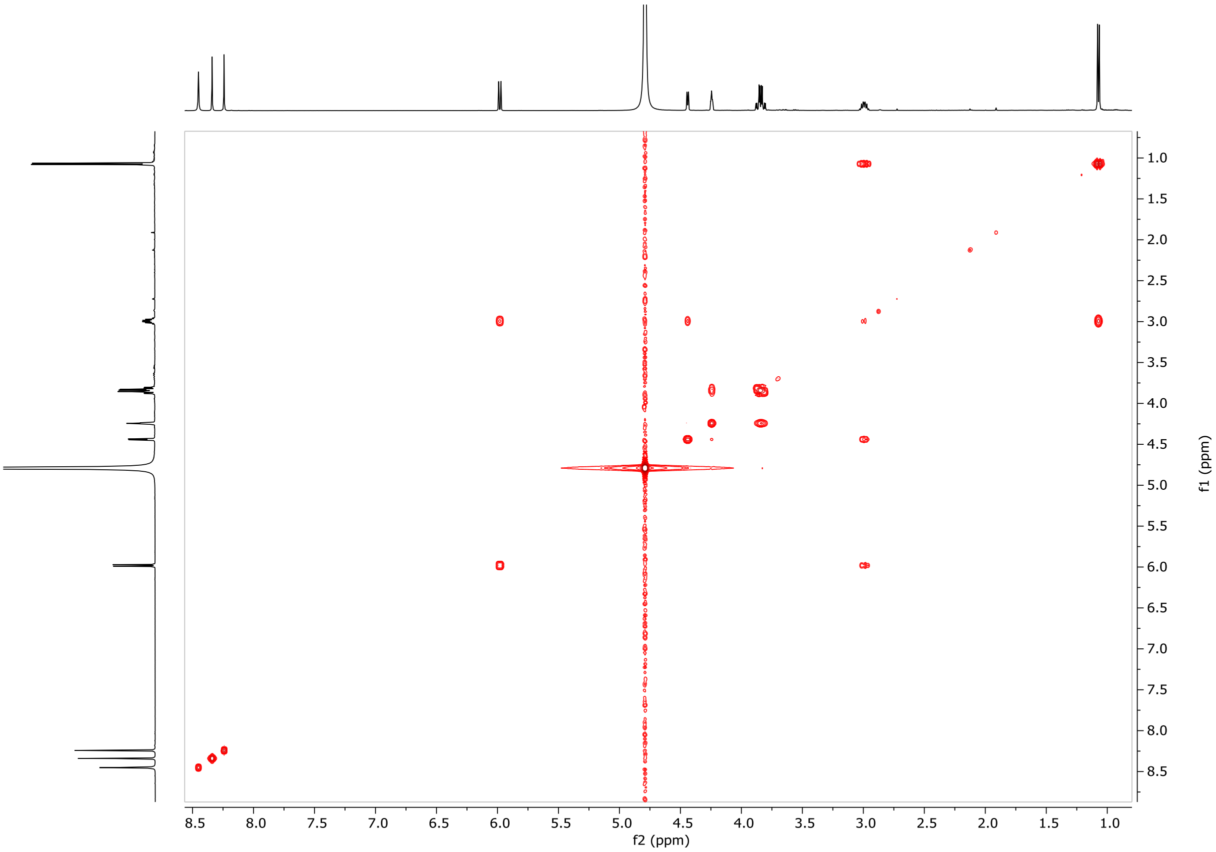

*Figure 131: ^{1}H-^{1}H COSY (500 MHz) of 2'Me-adenosine **27** biotransformation product*

15.4 2'-F Adenosine (**26**)

15.4.1 Biotransformation

A 2x reaction mixture was made up containing adenine **21** (2 mM), D-glyceraldehyde **16** (20 mM), fluoroethanol **17** (40 mM), ATP (10 mol%), PEP (40 mM), $MgCl_2$ (20 mM), $MnCl_2$ (20 mM), HEPES buffer (100 mM, pH 8), and water to bring the volume up to 2.5 mls. Alongside this a 2x enzyme mixture was made up containing DHAK (0.2 mg/ml), PK (20 U/ml), DERA F76A (4 mg/ml), PpAO (1 mg/ml), PPM (2 mg/ml), PNP (8 mg/ml), HEPES buffer (100 mM, pH 8), and water was added to bring the volume up to 2.5 ml. The 2x reaction mix and 2x enzyme mix were combined in a 15 ml falcon tube giving 5 mls final volume and final reaction concentrations half those stated. The reaction was left shaking at 180 rpm at 30 °C for 24 hours. The enzymes were removed using a 10K MWCO filter and the product was then purified by semiprep HPLC. Fractions were collected using mass-based fraction collection. Fractions were combined, acetonitrile was removed via genevac and then the sample was lyophillsed and isolated as a white solid, resuspended in D_2O and analysed by NMR.

15.4.2 NMR

^{1}H (800 MHz) 3.99 (dd, J =13.0, 3.8, 1H, H5'), 3.85 (dd, J = 13.0, 2.4, 1H, H5'), 4.28 (dt, J = 6.1, 2.9 Hz, 1H, H4'), 4.65 (ddd, J = 18.3, 7.1, 4.6 Hz, 1H, H3'), 5.49 (ddd, J = 52.0, 4.6, 2.5 Hz, 1H, H2'), 6.40 (dd, J = 16.9, 2.5 Hz, 1H, H1'), 8.25 (s, 1H, H8) 8.32 (s, 1H, H2).

Known impurities have been annotated on spectrum, peaks not annotated correspond to unknown impurities.

Product peaks in ^{1}H NMR spectra identical to literature[12].

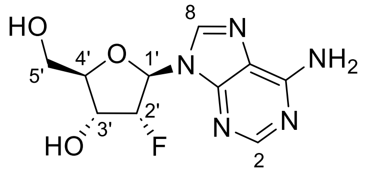

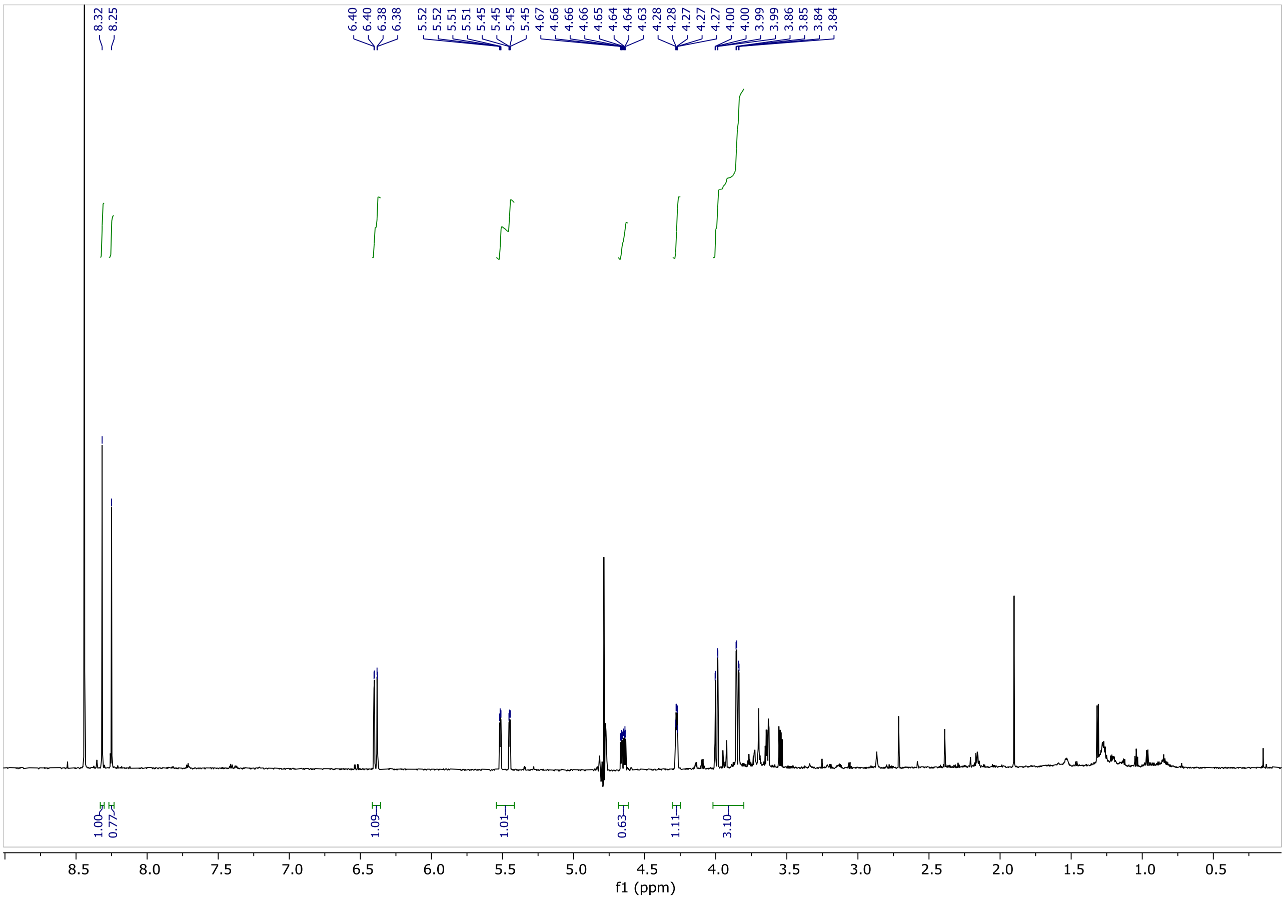

*Figure 132: ¹H NMR (800 MHz) 2'-F-adenosine **28** biotransformation product*

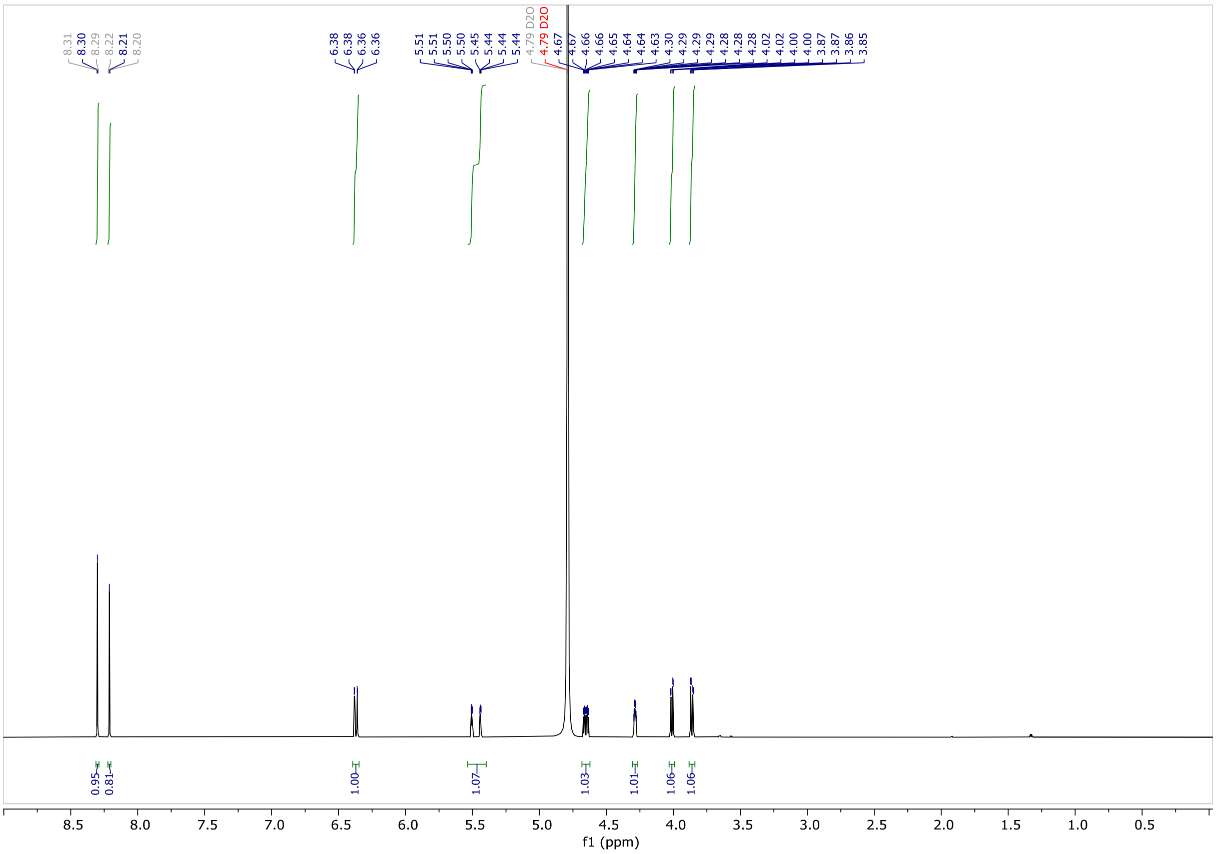

*Figure 133: ^{1}H NMR (800 MHz) 2'-F-adenosine **28** standard*

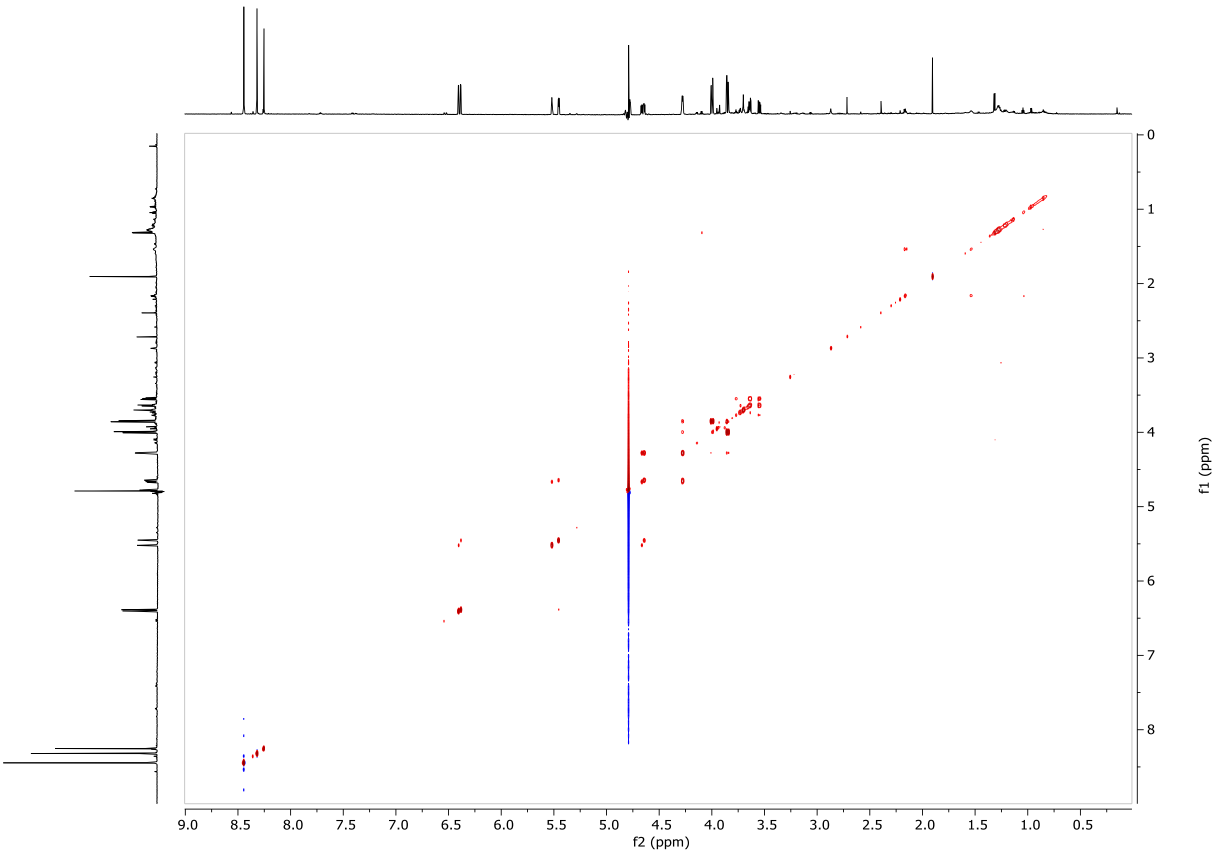

*Figure 134: ^{1}H-^{1}H COSY NMR (800MHz) spectrum of 2-F adenosine **28** biotransformation product*

15.4.3 Stereoselectivity

While 2'-OH and 2'-Me adenosine showed the presence of only one diastereomer at C2' (by NMR), the 2’F substrate showed the presence of both fluorine diastereomers at C2' These were present in a ratio of 98:2 favouring the Fluorine in the D-ribo conformation. The major diastereomer was assigned based on comparison to commercial standard.

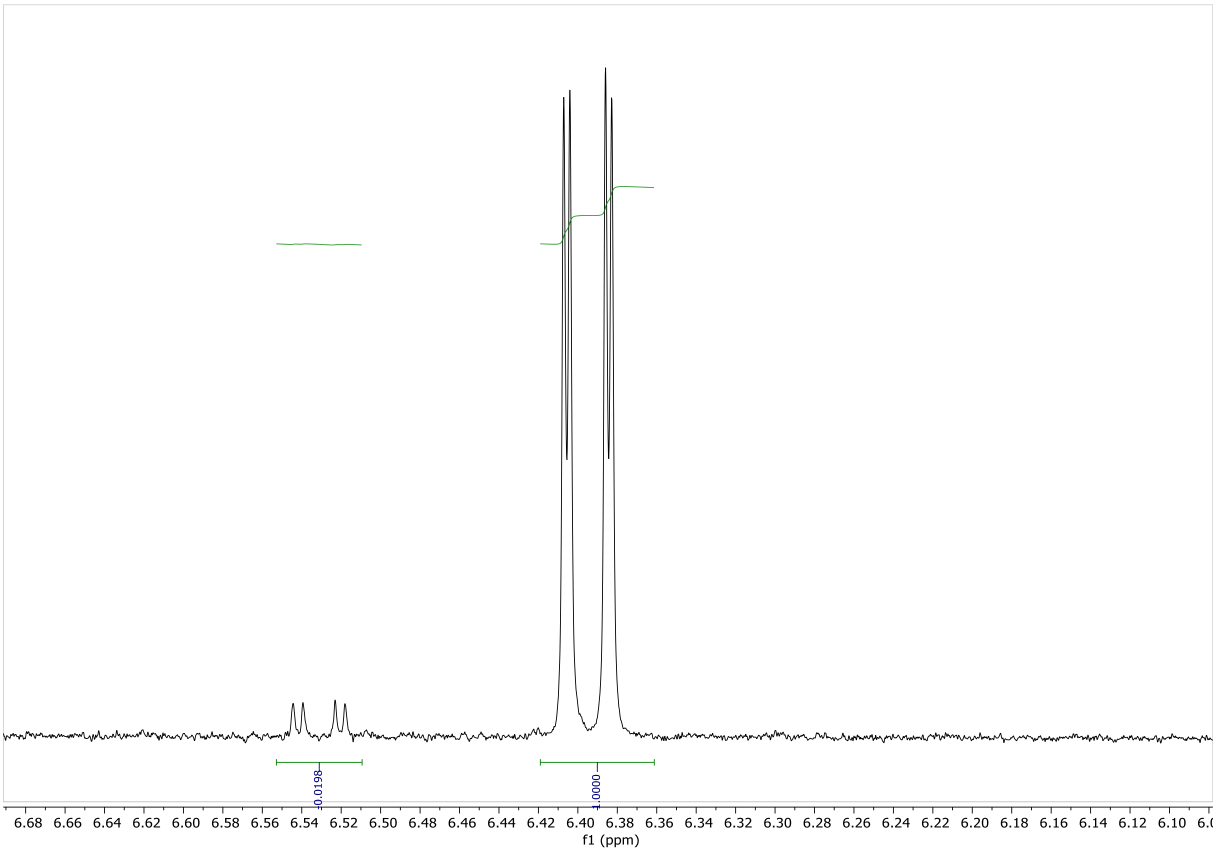

Figure 135: ^{1}H NMR of peak H1 showing the two fluorine diastereomers present in the product

15.5 2'-F-inosine (side product)

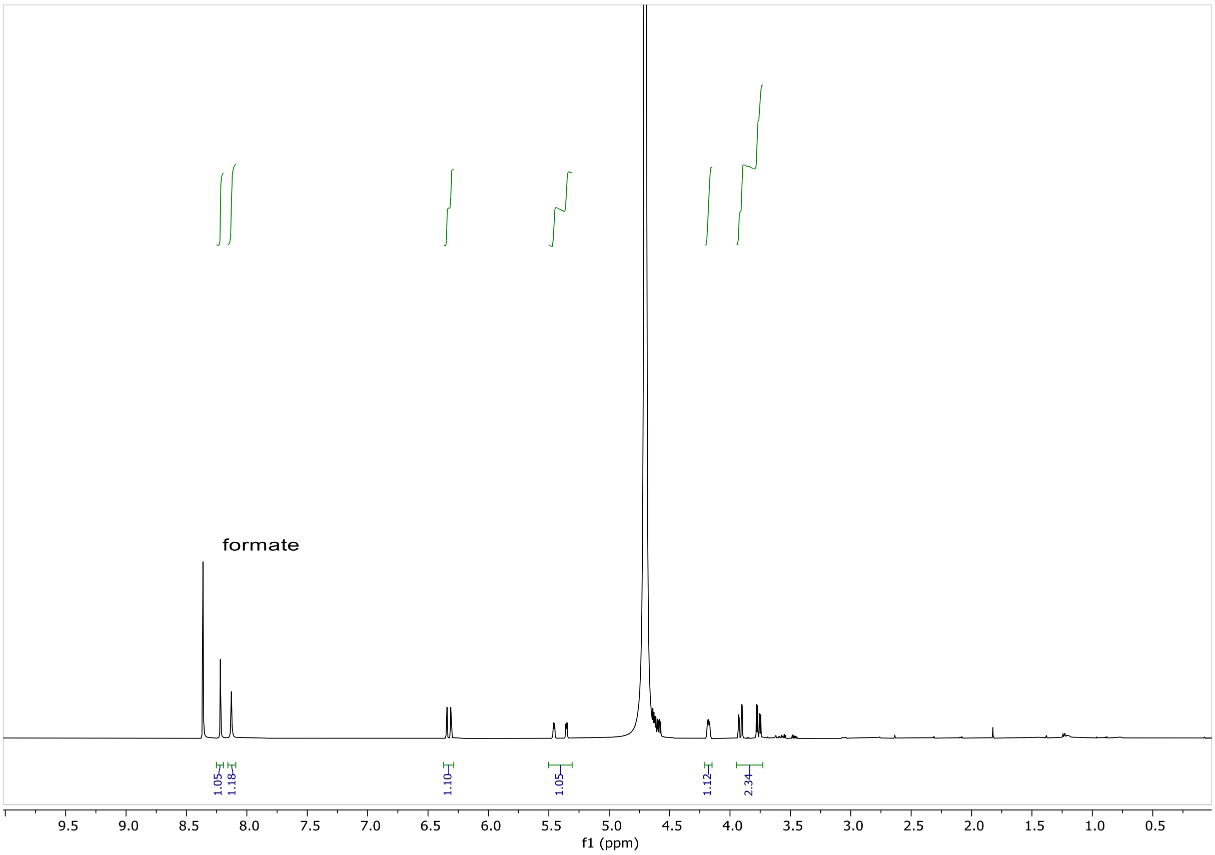

Figure 136: ^{1}H NMR spectrum of 2'-F-inosine side product

15.6 Vidarabine (**27**)

15.6.1 Biotransformation

A 2x reaction mixture was made up contaning adenine **21** (2 mM), D-glyceraldehyde **16** (2 mM), glycolaldehyde **2a** (8 mM), ATP (10 mol%), PEP (4 mM), $MgCl_2$ (2 mM), $MnCl_2$ (2 mM), HEPES buffer (50 mM, pH 8), and water to bring the volume up to 2.5 mls. Alongside this a 2x enzyme mixture was made up containing DHAK (0.2 mg/ml), pyruvate kinase (20 U/ml), FSA WT (4 mg/ml), PPM (0.2 mg/ml), PNP (0.2 mg/ml), HEPES buffer (50 mM, pH 8), and water to bring the volume up to 2.5 ml. The 2x reaction mix and 2x enzyme mix were combined in a 15 ml falcon tube giving 5 mls final volume and final reaction concentrations half those stated. The reaction was left shaking at 180 rpm at 30 °C for 18 hours (overnight). Enzyme was removed using a 10K MWCO filter and the product was purified by semiprep HPLC. Fractions were collected using mass-based fraction collection. Fractions were combined, acetonitrile was removed via genevac and then the sample was lyophillsed, isolated as a white solid, then resuspended in D_2O and analysed by NMR.

15.6.2 NMR

1H NMR (400 MHz) 3.87 (dd, *J* = 12.7, 4.9 Hz, 1H, H5'), 3.95 (dd, *J* = 12.7, 3.0 Hz, 1H, H5'), 4.06 (ddd, J = 6.6, 4.9, 3.1 Hz, 1H, H4'), 4.35 (t, J = 6.1 Hz, 1H, H3'), 4.55 (t, J = 5.7 Hz, 1H, H2') 6.41 (d, J = 5.6 Hz, 1H, H1'), 8.22 (s, 1H, H8), 8.35 (s, 1H, H2).

Known impurities have been annotated on spectrum, peaks not annotated correspond to unknown impurities.

^{1}H NMR identical to literature[9]

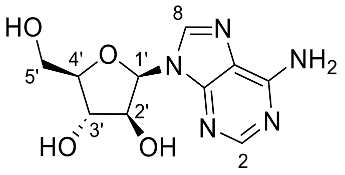

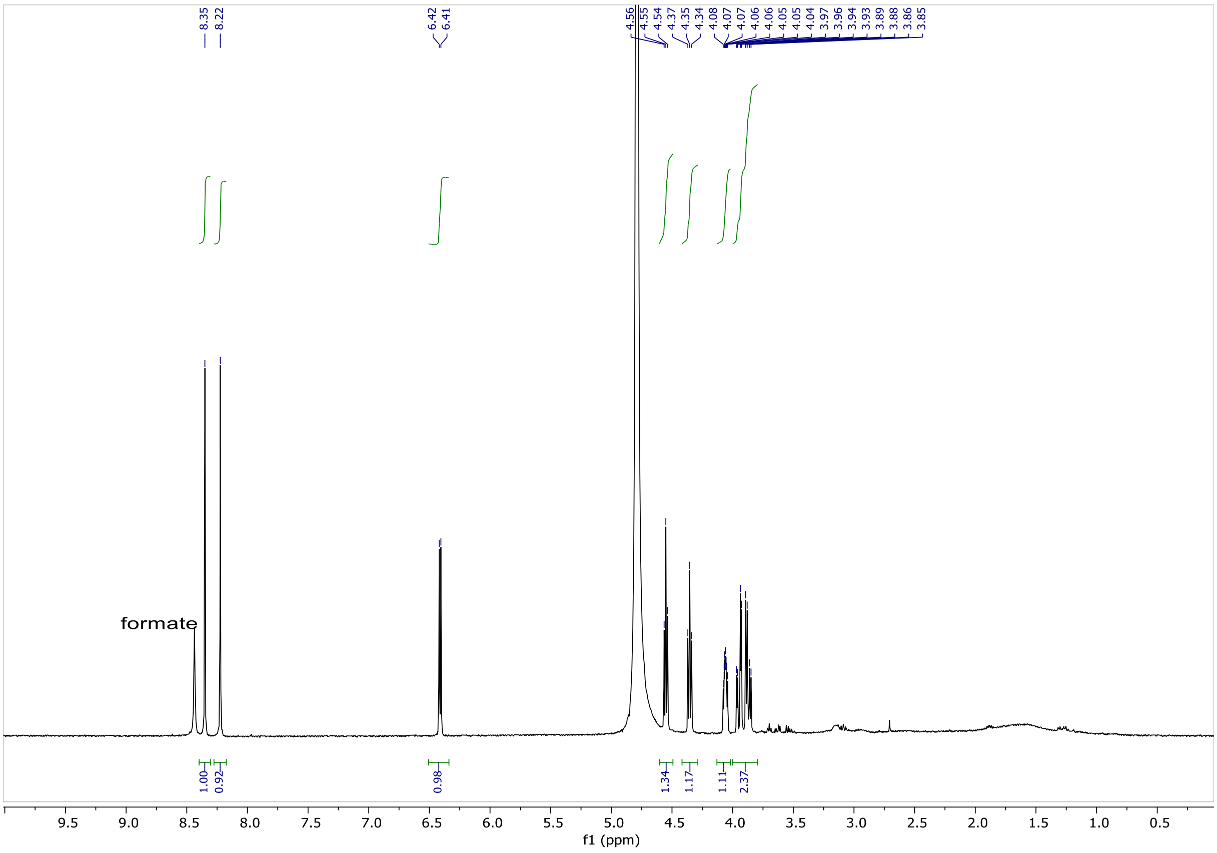

*Figure 137: ^{1}H NMR (500 MHz) of vidarabine **29** biotransformation*

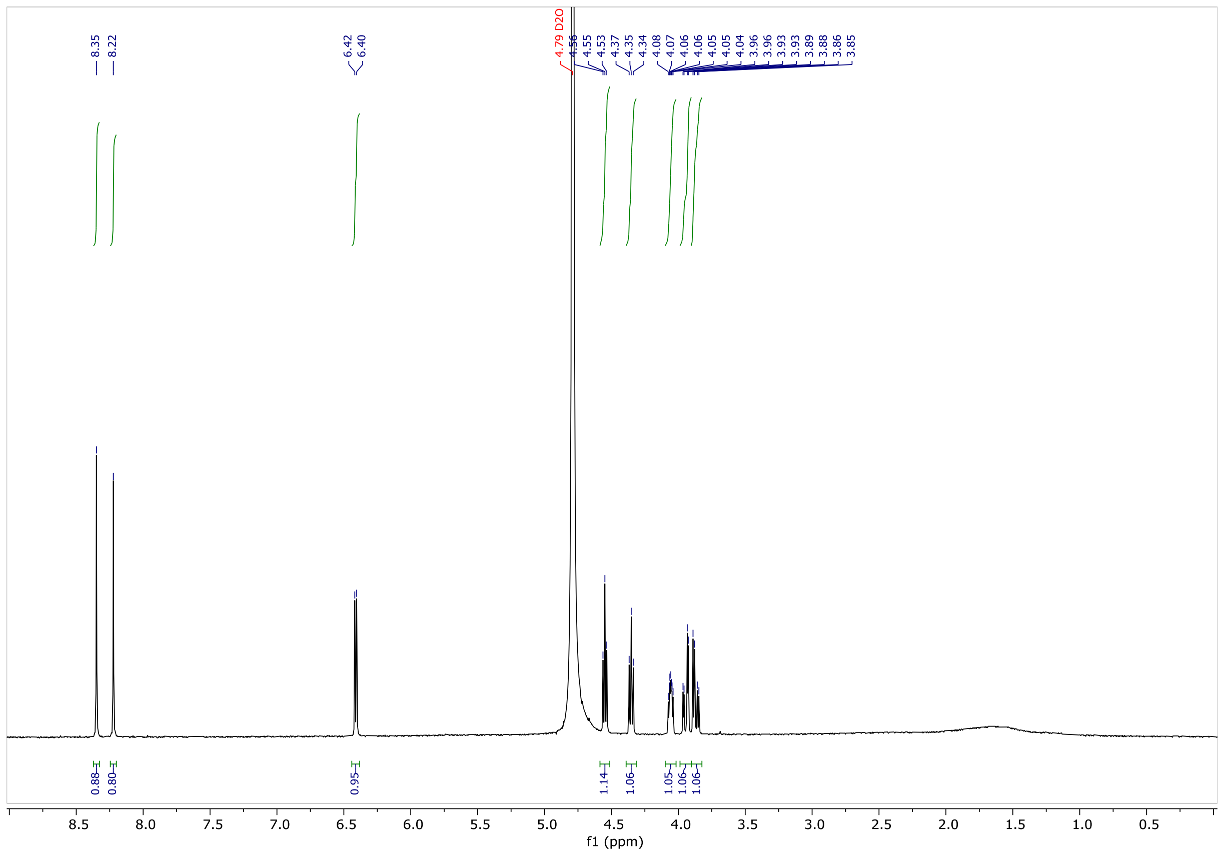

*Figure 138: ^{1}H NMR (500 MHz) of vidarabine **29** standard*

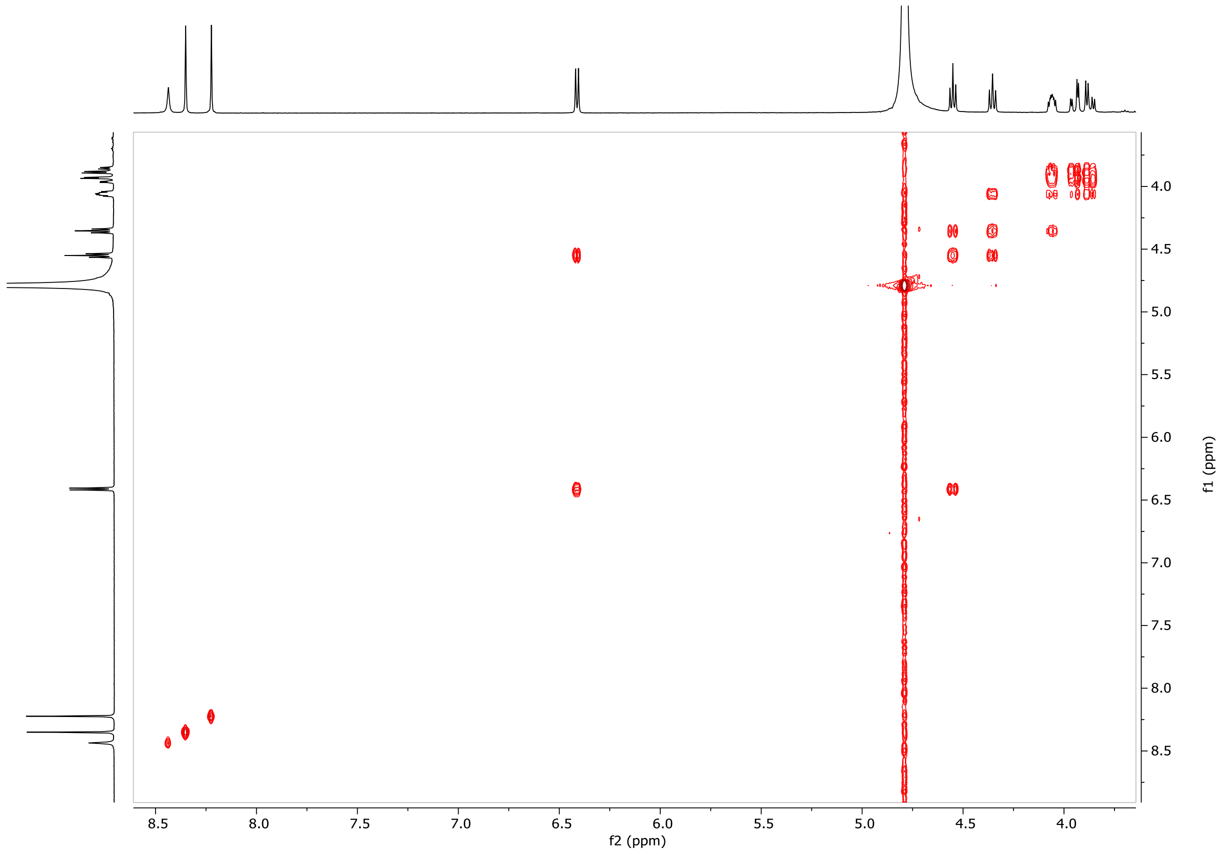

*Figure 139: ^{1}H-^{1}H COSY NMR (500 MHz) of vidarabine **29** biotransformation*

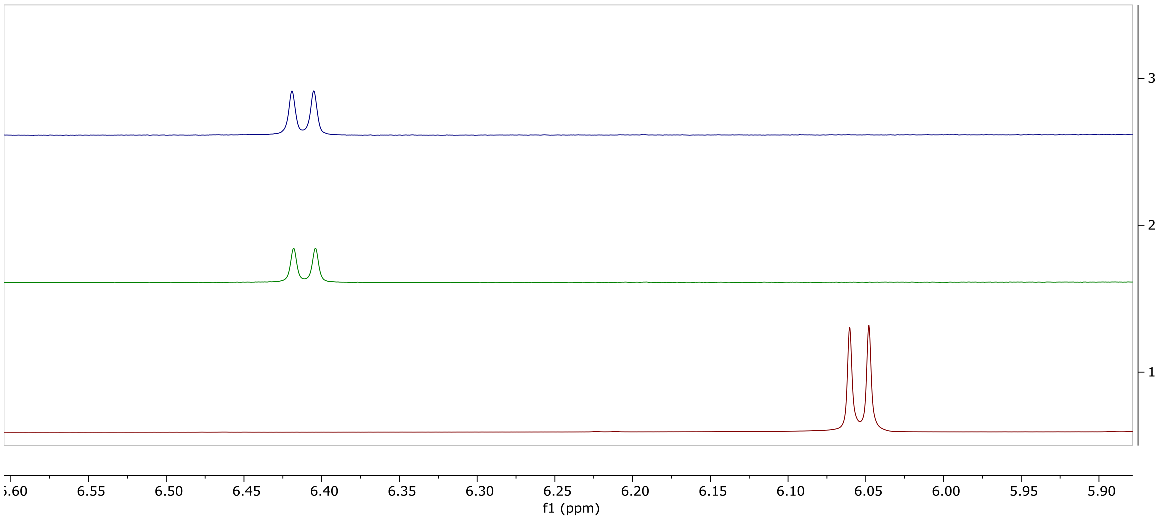

Figure 140: Top – 1H NMR peak H1' vidarabine biotransformation Middle- 1H NMR peak H1' vidarabine standard Bottom - 1H NMR peak H1' adenosine standard

16. Uncropped SDS-PAGE gel

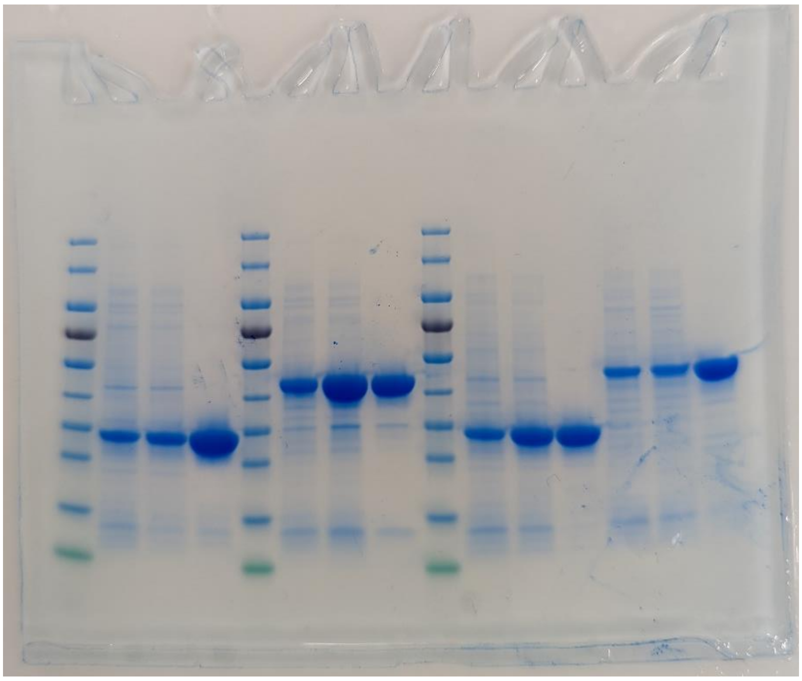

Figure 141: Uncropped SDS-PAGE gel from figure 1, additional lanes correspond to enzymes unused in this work.

References:

(1) Beber, M. E.; Gollub, M. G.; Mozaffari, D.; Shebek, K. M.; Flamholz, A. I.; Milo, R.; Noor, E. EQuilibrator 3.0: A Database Solution for Thermodynamic Constant Estimation. *Nucleic Acids Res* **2022**, *50* (D1), D603–D609. https://doi.org/10.1093/NAR/GKAB1106.

(2) Richard, J. P. Restoring a Metabolic Pathway. *ACS Chem Biol* **2008**, *3* (10), 605–607. https://doi.org/10.1021/cb800238s.

(3) Le Guilloux, V.; Schmidtke, P.; Tuffery, P. Fpocket: An Open Source Platform for Ligand Pocket Detection. *BMC Bioinformatics* **2009**, *10* (1), 168. https://doi.org/10.1186/1471-2105-10-168.

(4) Bianco, G.; Forli, S.; Goodsell, D. S.; Olson, A. J. Covalent Docking Using Autodock: Two-Point Attractor and Flexible Side Chain Methods. *Protein Science* **2016**, *25* (1), 295–301. https://doi.org/10.1002/pro.2733.

(5) Heine, A.; DeSantis, G.; Luz, J. G.; Mitchell, M.; Wong, C. H.; Witson, I. A. Observation of Covalent Intermediates in an Enzyme Mechanism at Atomic Resolution. *Science (1979)* **2001**, *294* (5541), 369–374. https://doi.org/10.1126/science.1063601.

(6) Fesko, K. Threonine Aldolases: Perspectives in Engineering and Screening the Enzymes with Enhanced Substrate and Stereo Specificities. *Appl Microbiol Biotechnol* **2016**, *100* (6), 2579. https://doi.org/10.1007/S00253-015-7218-5.

(7) Chambre, D.; Guérard-Hélaine, C.; Darii, E.; Mariage, A.; Petit, J. L.; Salanoubat, M.; De Berardinis, V.; Lemaire, M.; Hélaine, V. 2-Deoxyribose-5-Phosphate Aldolase, a Remarkably Tolerant Aldolase towards Nucleophile Substrates. *Chemical Communications* **2019**, *55* (52), 7498–7501. https://doi.org/10.1039/c9cc03361k.

(8) Kaspar, F.; Giessmann, R. T.; Neubauer, P.; Wagner, A.; Gimpel, M. Thermodynamic Reaction Control of Nucleoside Phosphorolysis. *Adv Synth Catal* **2020**, *362* (4), 867–876. https://doi.org/10.1002/adsc.201901230.

(9) Xu, J.; Green, N. J.; Russell, D. A.; Liu, Z.; Sutherland, J. D. Prebiotic Photochemical Coproduction of Purine Ribo- and Deoxyribonucleosides. *J Am Chem Soc* **2021**, *143* (36), 14482–14486. https://doi.org/10.1021/jacs.1c07403.

(10) Li, N.-S.; Piccirilli, J. A. Synthesis of 2′-Deoxy-2′- ***C*** -α-Methylpurine Nucleosides. *Synthesis (Stuttg)* **2005**, *2005* (17), 2865–2870. https://doi.org/10.1055/s-2005-872204.

(11) Lee, Y.-H.; Yeh, Y.-C.; Fan, P.-H.; Zhong, A.; Ruszczycky, M. W.; Liu, H. Changing Fates of the Substrate Radicals Generated in the Active Sites of the B $_{12}$ -Dependent Radical SAM Enzymes OxsB and AlsB. *J Am Chem Soc* **2023**, *145* (6), 3656–3664. https://doi.org/10.1021/jacs.2c12953.

(12) O'Reilly, D.; Stein, R. S.; Patrascu, M. B.; Jana, S. K.; Kurian, J.; Moitessier, N.; Damha, M. J. Exploring Atypical Fluorine–Hydrogen Bonds and Their Effects on Nucleoside Conformations. *Chemistry – A European Journal* **2018**, *24* (61), 16432–16439. https://doi.org/10.1002/chem.201803940.
